# The role of human milk oligosaccharides in shaping and restoring infant gut microbiota: population-based cohort study

**DOI:** 10.1016/j.ajcnut.2026.101318

**Published:** 2026-04-16

**Authors:** Minka Ovaska, Manu Tamminen, Mirkka Lahdenperä, Samuli Rautava, Aditya Jeevannavar, Heidi Isokääntä, Lars Bode, Hanna Lagström

**Affiliations:** 1 Department of Public Health, University of Turku and Turku University Hospital, Turku, Finland; 2 Centre for Population Health Research, University of Turku and Turku University Hospital, Turku, Finland; 3 Department of Biology, University of Turku, Turku, Finland; 4 Department of Pediatrics, University of Helsinki and Helsinki University Hospital and New Children’s Hospital, Pediatric Research Center, Helsinki, Finland; 5 Research Center for Infections and Immunity, Institute of Biomedicine, University of Turku, Turku, Finland; 6 Turku Bioscience Centre, University of Turku, Turku, Finland; 7 Department of Pediatrics, Larsson-Rosenquist Foundation Mother-Milk-Infant Center of Research Excellence (LRF MOMI CORE), and the Human Milk Institute (HMI), University of California San Diego, La Jolla, CA, United States; 8 Nutrition and Food Research Center, Faculty of Medicine, University of Turku, Turku, Finland

**Keywords:** gut microbiota, infancy, human milk oligosaccharides, secretor, cesarean section

## Abstract

**Background:**

Infant gut microbiota colonization is important for supporting normal development and long-term health of children. Human milk oligosaccharides (HMOs) influence the composition of the gut microbiota, but their specific effects, particularly after breastfeeding, remain poorly understood.

**Objectives:**

We aimed to deepen the understanding of how HMOs associate with the gut microbiota composition at 3 mo and at 13 mo of age. In addition, we assessed the role of HMOs as microbiome-rebalancing agents in cesarean-delivered infants.

**Methods:**

We analyzed fecal samples from infants at 3 mo (*n* = 517) and 13 mo (*n* = 522), along with human milk samples at 3 mo, from a population-based cohort. Gut microbiota was profiled by 16S rRNA sequencing, and 19 HMOs were quantified by high-performance liquid chromatography with fluorescent detection. Dirichlet multinomial mixtures clustering was used to identify bacterial fecal community types (FCTs) and multinomial logistic regression models to study the association between HMOs and FCTs. Permutational multivariate analysis of variance and linear regression models were used to associate HMOs with gut microbiota diversity measures and Spearman correlation to bacterial genera.

**Results:**

HMOs were associated with gut microbiota FCTs, diversity measures, and bacterial genera at 3 and 13 mo of age. At 3 mo, disialyllacto-N-tetraose and the structurally related lacto-N-sialyllactose b showed notable associations with the gut microbiota, whereas at 13 mo, fucodisialyllacto-N-hexaose was associated with multiple gut microbiota metrics. Maternal secretor status was associated with the gut microbiota beta diversity (*R*^2^ = 0.003, *P* < 0.05) and decreased Shannon diversity (*b* = –0.24, *P* < 0.05) at 3 mo, with diminishing associations at 13 mo (observed richness, *b* = –11, *P* < 0.05). Although no individual HMOs showed microbiome-rebalancing effects in cesarean-born infants, infants fed by nonsecretor mothers exhibited stronger cesarean-related microbiota patterns compared with those fed by secretors.

**Conclusions:**

HMOs exhibit age-dependent and structure-specific associations with infant gut microbiota, extending beyond breastfeeding.

## Introduction

The initial colonization of the infant gut is crucial because it influences future microbiota composition and subsequently impacts child health and development [1]. Supporting the healthy establishment of this ecosystem and mitigating disruptive factors, such as cesarean section delivery, is therefore important [2]. The composition of human milk, particularly the presence of human milk oligosaccharides (HMOs), plays a significant role in shaping the gut microbiota composition as well as rebalancing it after perturbations [3].

HMOs are complex carbohydrates that pass through the gastrointestinal tract intact and are directly accessible for the gut microbiota to use. Only bacteria equipped with genes associated with the degradation of HMOs are capable of metabolizing them, which selectively supports the growth of specific microbial populations, such as a variety of *Bifidobacterium* species, particularly *Bifidobacterium longum subsp. infantis* [4,5]. In addition, certain species of *Bacteroides* and *Lactobacillus* have shown high HMO metabolic capacity [[6], [7], [8], [9]]. HMOs can also influence gut microbiota development and composition through mechanisms such as microbiome cross-feeding, where HMO degradants produced by specific bacteria are shared with other microbes that are unable to degrade HMOs directly [10,11].

Approximately 200 structurally different HMOs are known. The structural and conformational diversity of these oligosaccharides contributes to their different biological functions. The type and concentration of HMOs produced vary between mothers, determined partly by maternal genetic polymorphisms (e.g., secretor status), and vary over the course of lactation [12]. A large part of the variation between individual HMO composition stems from maternal secretor status determined by fucosyltransferase-2 (FUT2) gene activity. Individuals harboring a nonfunctional FUT2 gene are termed nonsecretors, and they have markedly reduced human milk levels of α1-2-fucosylated HMOs [e.g., 2′-fucosyllactose (2ʹFL)] compared with secretor mothers with a functional FUT2 gene [12].

Breastfeeding has been reported to promote the restoration of the infant gut microbiota perturbed after cesarean section delivery, but little is known about the role of specific human milk components, including HMOs, in this modulation [13]. Notably, cesarean section delivery is often associated with decreased abundance of *Bacteroides* species, which are key HMO utilizers. Research highlights differences in the gut microbiota composition and HMO interactions between vaginally and cesarean-delivered infants, but there is currently little evidence regarding whether individual HMOs can mitigate the negative impact of cesarean section on infant gut microbiota [[13], [14], [15]].

Despite the increasing recognition of the critical role that HMO composition plays in shaping the infant gut microbiota, comprehensive research involving large sample sizes remains scarce. Current literature reveals substantial gaps in our understanding of the specific associations between individual HMOs and infant gut microbiota composition, especially in the long term. Here, we investigate the association between HMO composition in human milk and infant gut microbiota composition during breastfeeding, and whether this is reflected in the gut microbiota at a later age. We also investigate the potential of HMOs to act as microbiome-rebalancing agents in infants delivered via cesarean section. We hypothesize that variations in HMO composition are linked to differences in gut microbiota composition during breastfeeding and that these associations may extend beyond the breastfeeding period.

## Methods

### Study subjects and maternal secretor status

**FIGURE 1:**
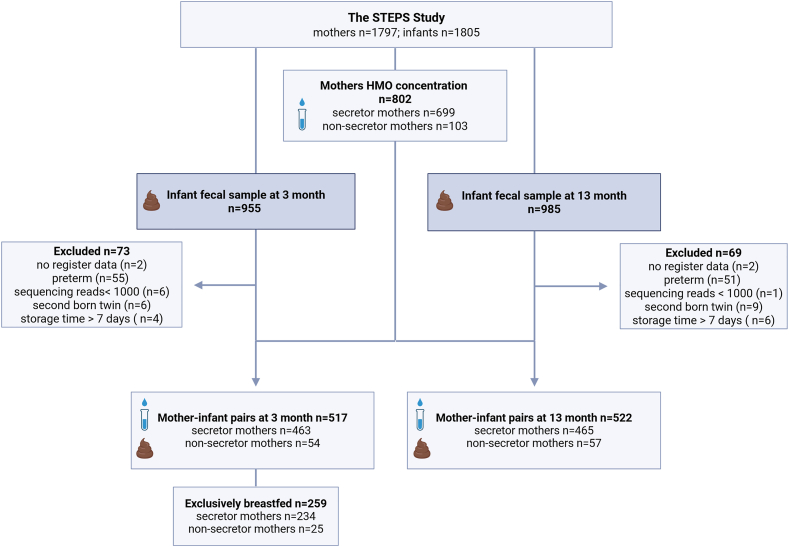
Flow chart of the study. This study focuses on the subcohort of the STEPS Study, comprising mother–infant pairs from whom both human milk samples at 3 mo and infant fecal samples at 3 mo (*n =* 517) and at 13 mo (*n =* 522) were available. Fecal samples were collected at both early and late infancy, representing 2 distinct time points in the study. Created with BioRender.com. HMO, human milk oligosaccharide; STEPS Study, Steps to Healthy Development of Children.

This study was based on the Steps to Healthy Development of Children (the STEPS Study), a longitudinal population-based follow-up study described in detail elsewhere [16]. In brief, the source population included all Finnish- and Swedish-speaking mothers who delivered a child between January 2008 and March 2010 in the Hospital District of Southwest Finland, comprising 13,436 mothers and their 14,946 children. From this cohort, 1797 mothers and 1658 fathers, with 1805 neonates, volunteered for the intensive follow-up group of the STEPS Study. The only inclusion criterion for recruitment was family language (Finnish or Swedish). The ethics committee of the Hospital District of Southwest Finland has approved the STEPS Study (2/2007). The parents gave their written informed consent for the study. The legal basis for processing personal data is public interest and scientific research [European Union General Data Protection Regulation 2016/679, Article 6(1)(e) and Article 9(2)(j); Data Protection Act, Sections 4 and 6]. In this study, we focused on a subcohort of mother–infant pairs from whom both human milk HMO concentrations at 3-mo and infant fecal samples at 3 mo (*n =* 517) and 13 mo (*n =* 522) were available (Figure 1). Maternal secretor status was determined by the high abundance >100 nmol/mL (secretor) or near absence <100 nmol/mL (nonsecretor) of the HMO 2′FL in the respective human milk samples.

### Human milk collection and HMO analysis

Detailed methods for human milk collection and HMO analysis have been described previously [17]. Briefly, mothers collected human milk samples when infants were ∼3 mo old (median: 2.6 mo; range: 0.6–6.0 mo). Written instructions were provided to the mothers who obtained the samples by manual expression in the morning from a single breast, discarding initial drops before collecting 10 mL into plastic containers. Samples were stored at +4°C, transported to the research center the same day, and frozen at −70°C until further analysis. The volume of human milk consumed by the infant was not measured.

HPLC with fluorescent detection was employed to identify HMOs in the human milk samples as previously described [[17], [18], [19]] at the University of California, San Diego. Human milk samples were spiked with raffinose, a non-HMO carbohydrate, as an internal standard to allow absolute quantification. Oligosaccharides were extracted by high-throughput solid-phase extraction over C18 and Carbograph microcolumns and fluorescently labeled with 2-aminobenzamide. Labeled oligosaccharides were separated by HPLC on an amide-80 column (150 × 2 mm inner diameter, 3 μm particle size; Tosoh Bioscience) using a 50 mmol/L ammonium formate–acetonitrile buffer system at 25°C and detected by fluorescence (excitation 360 nm; emission 425 nm). Peak annotation was based on standard retention times and confirmed by mass spectrometry (MS) using a Thermo LCQ Duo ion trap mass spectrometer equipped with a nanoelectrospray ionization source.

Absolute concentrations for 19 HMOs from 802 human milk samples were calculated based on HMO standard response curves and corrected for internal standard recovery (oligosaccharide detection limit: ∼20 pmol, dynamic range between 20 and 5000 pmol; milk samples were diluted accordingly). Quantified HMOs included: 2′FL, 3-fucosyllactose, lacto-N-neotetraose (LNnT), 3′-sialyllactose, difucosyllactose, 6′-sialyllactose, lacto-N-tetraose (LNT), lacto-N-fucopentaose (LNFP) I, LNFP II, LNFP III, sialyl-LNT (LST) b, LSTc, difucosyllacto-LNT, lacto-N-hexaose, disialyllacto-N-tetraose (DSLNT), fucosyllacto-N-hexaose, difucosyllacto-N-hexaose, fucodisialyllacto-N-hexaose (FDSLNH), and disialyllacto-N-hexaose. Using HPLC with fluorescence detection and MS confirmation, these HMOs were quantified at the compositional level; individual linkage and positional isomers were not structurally resolved and therefore represent pooled isomeric compounds. The total concentration of HMOs was calculated as the sum of the 19 oligosaccharides. HMO-bound fucose and HMO-bound sialic acid were calculated on a molar basis. The proportion of each HMO comprising the total HMO concentration was also calculated. HMO diversity was calculated as Simpson’s Reciprocal Index 1/D, which is the reciprocal sum of the square of the relative abundance of each of the measured 19 HMOs [18].

### Fecal sample collection and gut microbiota analysis

Detailed fecal sample collection and gut microbiota analysis methods have been described previously [20]. Briefly, parents collected fecal samples at home into sterile collection tubes without additives when the child was aged 3 and 13 mo, recorded collection times, and delivered or mailed samples to the laboratory at ambient temperature. On average, samples were stored at +4°C or ambient temperature for a mean of 2 d before freezing at −80°C. Only samples with a corresponding human milk sample were included in the study. Of the 1805 infants enrolled in the STEPS Study, 517 provided a fecal sample with a corresponding human milk sample at the 3-mo time point (median: 2.8 mo, range: 0.9–4.4 mo) and 522 at 13-mo time point (median: 13.7 mo, range: 11–17 mo). At 3-mo time point, the median difference between milk and stool sample collection was 3 d. Preterm infants, second-born twins, and fecal samples stored over 7 d before freezing were excluded from the study (Figure 1).

DNA was extracted from 30 to 100 mg of fecal material using the Qiagen DNeasy 96 PowerSoil Pro QIAcube HT kit according to the manufacturer’s protocol. DNA extraction was performed at the Center of Evolutionary Applications, University of Turku. Gut microbiota composition was determined using 16S rRNA gene sequencing targeting the V3–V4 hypervariable regions. Sequencing and the sequencing library preparation were performed at the Finnish Functional Genomics Centre Facility (University of Turku, Åbo Akademi University, and Biocenter Finland). Sequencing libraries were prepared following the Illumina 16S Metagenomic Sequencing Library Preparation protocol using primers Bakt_341F (CCTACGGGNGGCWGCAG) and Bakt_805R (GACTACHVGGGTATCTAATCC) [21]. Sequencing was performed on the MiSeq v3 platform (Illumina). Positive (ZymoBIOMICS Microbial Community DNA Standard D6305/D6306) and negative (water) control samples were included to assess sequencing accuracy and contamination.

Amplicon sequence variants (ASVs) were obtained from the raw data using the nfcore/ampliseq pipeline version 2.1.0 with default parameters [22]. Taxonomic assignments were achieved by aligning reads against the Silva 138.1 prokaryotic SSU taxonomic database [23]. Sequencing read numbers were not equalized by rarefying because this procedure causes a significant and unnecessary loss of data [24], but samples with <1000 reads were excluded (*n =* 7) (Figure 1). After preprocessing, we had a total of 11,306,158 sequencing reads from 517 samples from 3-mo-old infants (on average 21,868 reads per sample, range: 1,034–142,762 reads per sample) and 14,197,146 sequencing reads from 522 samples from 13-mo-old infants (on average 27,197 reads per sample, range 5,720–94,432 reads per sample) (Supplemental Figure 1).

### Background factors

Pre- and perinatal characteristics of the infants and their mothers were extracted from the Medical Birth Register maintained by the Finnish Institute for Health and Welfare. These variables included child birthweight, sex assigned at birth, duration of pregnancy and preterm birth (birth occurring before 37 wk of gestation), maternal age, mode of delivery (vaginal or cesarean section), primiparity (no previous deliveries), maternal prepregnancy BMI, and intrapartum antibiotic treatment, as well as neonatal antibiotic therapy during the first 7 d of life. The use of intrapartum antibiotic treatment was categorized as ever or never received and discussed in the text as a variable “maternal antibiotic.” Neonatal antibiotic therapy during the first 7 d of life was additionally categorized as ever or never received and discussed as “infant antibiotics” in the text.

Information about breastfeeding was obtained via a self-administered follow-up diary. Breastfeeding status was categorized into 3 groups: exclusive breastfeeding (defined as an infant receiving no food other than a human milk, except for water, at the time of fecal sample collection), partial breastfeeding (infants who had been introduced to milk formulas or solid foods and continue breastfeeding at the time of fecal sample collection), and no breastfeeding (infants who were not receiving human milk at the time of fecal sample collection). Infants with missing information on breastfeeding were categorized as unknown. For the 13-mo time point, an additional variable was created for sensitivity analyses. Breastfeeding status and the time since cessation of breastfeeding were categorized as follows: current breastfeeding (infants who had been introduced to milk formulas or solid foods and continue breastfeeding at the time of fecal sample collection), <1 mo, 1–3 mo, 3–6 mo, or >6 mo since cessation of breastfeeding at the time of fecal sample collection. Infants with missing data were classified as unknown.

### Statistical analyses

Statistical analyses were conducted in the R computing environment (version 4.4.0) (Supplemental Methods) [25]. Analyses were conducted separately for early infancy (around 3 mo of age) and late infancy (around 13 mo of age). To maximize the sample size and also control for the breastfeeding status, the analyses were conducted separately for the whole cohort of 3-mo-old infants (*n =* 517) and for those exclusively breastfed (*n =* 259) by the time of fecal sample collection. In the 13-mo time point, the analyses were conducted for the whole cohort (*n =* 522), with additional sensitivity analyses adjusting for breastfeeding status and the time since cessation of breastfeeding. To assess the role of maternal secretor status, stratified analyses were conducted for secretors and nonsecretors. On the basis of previous literature, we selected the following factors, associated with both maternal HMO composition and infant gut microbiota, as covariates: birth mode (vaginal or cesarean section delivery) [[26], [27], [28]], parity (primiparous or multiparous) [29,30], and maternal prepregnancy BMI [17,31] (Supplemental Figure 2). These covariates were used throughout the analysis. HMO concentrations were scaled using a *z*-score transformation before statistical analysis. To support interpretation of the *z*-score standardized HMO concentration results, Supplemental Tables 1–3 report the median, mean, and SD values for each HMO. All *P* values were adjusted for multiple comparisons using the Benjamin–Hochberg procedure. Both corrected and uncorrected *P* values are discussed in the text.

### Fecal community types and association with HMO concentration

Fecal community types (FCTs) were identified using Dirichlet multinomial mixtures (DMMs) clustering on genus-level data using the functions from the mia package (version 1.15.6) [32]. The appropriate number of community types was determined based on the Laplace criteria. To evaluate the association between HMOs and FCTs, covariate-adjusted multinomial logistic regression models were performed using the nnet R package (version 7.3-19). To summarize and compare other background factors across FCTs, we used the tableone R package (version 0.13.2), applying the Kruskal–Wallis test for continuous variables and the Chi-squared test for categorical variables.

### Gut microbiota diversity and association with HMO concentration

Beta diversity (Bray–Curtis) index was calculated using the vegdist function from the vegan package (version 2.6-8) [33]. ASV counts were transformed to relative abundance before diversity analysis. We applied stepwise variable selection to study the association between scaled HMO concentrations and gut microbiota beta diversity using the ordistep function from the vegan package, followed by covariate (birth mode, parity, and maternal prepregnancy BMI) adjusted permutational multivariate analysis of variance (PERMANOVA) with 999 permutations using adonis2 function from vegan package. The homogeneity of the group dispersion was assessed using the permdist function from vegan package.

Alpha diversity, measured as observed richness and Shannon diversity, was calculated on the ASV-level data using the functions from the mia package. Linear regression models were used to study the relationships between scaled HMO concentrations and alpha diversity using the generalized linear model (glm) function from the stats package [25]. Models were adjusted with covariates (birth mode, parity, maternal prepregnancy BMI), and model assumptions were tested using the DHARMa R package (version 0.4.6) [34].

### Gut microbiota genera and association with HMO concentration

Associations between the top 20 microbial genera and scaled HMO concentrations were analyzed using nonparametric Spearman correlation, using functions from the mia R package. The ComplexHeatmap (version 2.20.0) package [35] was used for the visualizations, with rows hierarchically clustered using Euclidean distance and complete linkage. Differential abundance analysis was performed using the LinDA package version 0.1.0 [36], which fits a linear regression model, and the ALDEx2 package version 1.30.0 [37], which fits a regression model on the centered log-ratio–transformed data. The analysis was conducted on genus-level data, focusing on genera present in >10% of the samples.

### Cesarean delivery and associations with HMO concentration

To investigate the relationship between HMO composition and FCTs in a subcohort of cesarean-born infants (*n =* 55), a covariate-adjusted (parity, maternal prepregnancy BMI) logistic regression model was used with the glm function from the stats package. A binary outcome variable was created to compare infants assigned to 3M-FCT3 with those assigned to 3M-FCT1 and 3M-FCT2 combined, based on prior evidence suggesting that FCT3 represents an atypical microbiota profile for 3-mo-old infants [3,[38], [39], [40]]. To summarize and compare other background factors across FCTs, we used the tableone R package applying the Kruskal–Wallis test for continuous variables and the Fisher’s exact test for categorical variables.

## Results

### Participant characteristics

The clinical characteristics of the infants and their mothers are presented in Table 1. On the basis of the abundance of the HMO 2′FL in the milk samples, 90% of the infants received milk from secretor mothers. During early infancy, 50% of the infants were exclusively breastfed by the time of fecal sampling. During late infancy, most of the infants (66%) did not receive human milk anymore.

**TABLE 1:** Descriptive characteristics of infants (at 3 and 13 mo) and their mothers in the STEPS Study subcohort, stratified by maternal secretor status

Variable, *n* (%)/mean (SD)^1^	3 mo			13 mo		
	Total	Nonsecretor	Secretor	Total	Nonsecretor	Secretor
*n*	517	54	463	522	57	465
Sex						
Boy	281 (54.4%)	30 (55.6%)	251 (54.2%)	279 (53.4%)	33 (57.9%)	246 (52.9%)
Girl	236 (45.6%)	24 (44.4%)	212 (45.8%)	243 (46.6%)	24 (42.1%)	219 (47.1%)
Infant age (mo)	2.83 (0.45)	2.77 (0.37)	2.84 (0.45)	13.67 (0.66)	13.77 (0.55)	13.66 (0.67)
Birth weight (kg)	3.58 (0.46)	3.64 (0.47)	3.57 (0.46)	3.58 (0.46)	3.65 (0.47)	3.57 (0.46)
Duration of pregnancy (wk)	40.08 (1.23)	40.17 (1.30)	40.07 (1.22)	40.05 (1.22)	40.10 (1.24)	40.04 (1.21)
Birth mode						
Vaginal	462 (89.4%)	44 (81.5%)	418 (90.3%)	462 (88.5%)	45 (78.9%)	417 (89.7%)
C-section	55 (10.6%)	10 (18.5%)	45 (9.7%)	60 (11.5%)	12 (21.1%)	48 (10.3%)
Breastfeeding status						
Exclusive	259 (50.1%)	25 (46.3%)	234 (50.5%)	—	—	—
No	17 (3.3%)	3 (5.6%)	14 (3.0%)	343 (65.7%)	39 (68.4%)	304 (65.4%)
Partial	154 (29.8%)	18 (33.3%)	136 (29.4%)	70 (13.4%)	11 (19.3%)	59 (12.7%)
Unknown	87 (16.8%)	8 (14.8%)	79 (17.1%)	109 (20.9%)	7 (12.3%)	102 (21.9%)
Maternal age (y)	31.25 (4.32)	30.53 (4.12)	31.34 (4.34)	31.34 (4.44)	30.88 (4.36)	31.40 (4.45)
Prepregnancy BMI (kg/m^2^)	24.23 (4.69)	24.17 (4.99)	24.24 (4.66)	24.17 (4.60)	24.49 (5.36)	24.13 (4.50)
Previous deliveries						
No	318 (61.5%)	36 (66.7%)	282 (60.9%)	321 (61.5%)	38 (66.7%)	283 (60.9%)
>1	199 (38.5%)	18 (33.3%)	181 (39.1%)	201 (38.5%)	19 (33.3%)	182 (39.1%)
Maternal antibiotics^2^						
No	454 (87.8%)	47 (87.0%)	407 (87.9%)	459 (87.9%)	52 (91.2%)	407 (87.5%)
Yes	63 (12.2%)	7 (13.0%)	56 (12.1%)	63 (12.1%)	5 (8.8%)	58 (12.5%)
Infant antibiotics^3^						
No	455 (88.0%)	48 (88.9%)	407 (87.9%)	462 (88.5%)	51 (89.5%)	411 (88.4%)
Yes	62 (12.0%)	6 (11.1%)	56 (12.1%)	60 (11.5%)	6 (10.5%)	54 (11.6%)

Abbreviation: STEPS Study, Steps to Healthy Development of Children.

1 Sample sizes and percentages are given for categorical variables, and means with SDs for continuous variables.

2 Maternal antibiotics refer to intrapartum antibiotic treatment ever (yes) or never (no) received.

3 Infant antibiotics refer to neonatal antibiotic therapy during the first 7 d of life ever (yes) or never (no) received.

### Gut microbiota composition and FCTs

**FIGURE 2:**
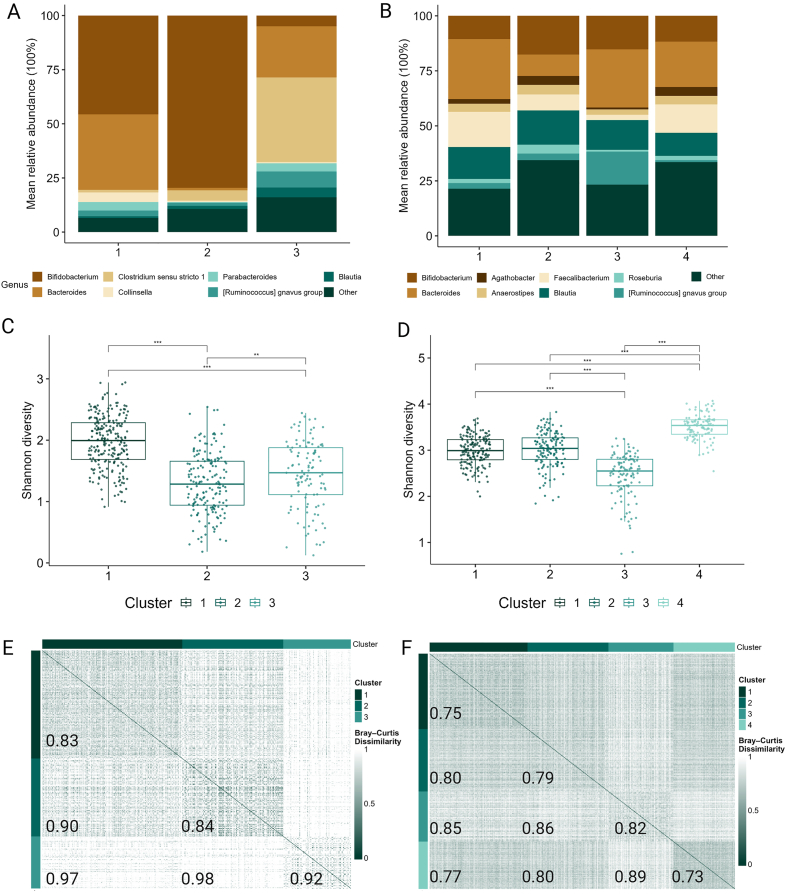
Fecal community types (FCTs) and associated HMO concentrations. FCTs were identified using Dirichlet multinomial mixtures clustering based on genus-level data. Bar plots showing the relative abundance of dominant genera in FCTs of (A) 3-mo-old and (B) 13-mo-old infants. Shannon diversity and the Wilcoxon rank-sum test were applied to compare the alpha diversity between FCTs. Statistical significance is denoted as follows (∗∗∗ for BH adjusted *P* value < 0.001, ∗∗ for BH adjusted *P* value < 0.01) for (C) 3-mo-old and (D) 13-mo-old infants. Bray–Curtis dissimilarity index was applied to compare the community compositions (beta diversity) within and between FCTs for (E) 3-mo-old and (F) 13-mo-old infants. The higher the dissimilarity index, the more different the gut microbiotas are between the FCTs. BH, Benjamin–Hochberg; HMO, human milk oligosaccharide.

Infant gut microbiota formed 3 distinct FCTs at 3 mo, and 4 at 13 mo (Figure 2), identified using DMM clustering based on genus-level data (Supplemental Figures 3 and 4). At 3 mo, 3M-FCT1 (*n =* 235) was characterized by *Bifidobacterium* and *Bacteroides*, 3M-FCT2 (*n =* 169 infants) with *Bifidobacterium*, and 3M-FCT3 (*n =* 113 infants) with *Clostridium sensu stricto 1*. 3M-FCT3 significantly differed from the other FCTs by both alpha diversity and beta diversity (Figure 2C and E). Birth mode (vaginal or cesarean section delivery), previous deliveries, number of sequencing reads, mother’s age, maternal antibiotics, and maternal secretor status (Supplemental Table 4), as well as HMO composition (Table 2), were found to be associated with the FCTs at the 3-mo time point.

**TABLE 2:** Significant association of *z*-score transformed HMO concentrations^1^ with FCTs in 3-mo-old (*n =* 517) infants assessed with covariate-adjusted multinomial logistic regression models

	FCT types 3-mo OR (95% CI)^2^		
	3M-FCT2 vs. 3M-FCT1^3^	3M-FCT3 vs. 3M-FCT1	3M-FCT3 vs. 3M-FCT2
Secretor (yes)	1.91 (0.86, 4.23)	0.57 (0.28, 1.14)	0.30 (0.13, 0.66)∗^4^
Total HMO concentration	1.22 (0.97, 1.53)	0.88 (0.7, 1.09)	**0.72 (0.56, 0.91)∗**
HMO-bound fucose	1.19 (0.95, 1.48)	0.87 (0.70, 1.09)	**0.74 (0.58, 0.94)∗**
2′FL	1.20 (0.97, 1.48)	0.92 (0.73, 1.17)	0.77(0.60, 0.98)∗
DFLac	0.97 (0.79, 1.20)	0.72 (0.54, 0.96)∗	0.74 (0.56, 0.98)∗
6′SL	0.85 (0.68, 1.06)	1.09 (0.87, 1.36)	1.29 (1, 1.65)∗
LNFP_II	0.86 (0.69, 1.06)	1.14 (0.91, 1.43)	1.33 (1.05, 1.69)∗
LNFP_III	0.53 (0.25, 1.14)	1.86 (0.86, 4.02)	**3.53 (1.47, 8.45)**∗
FDSLNH	0.83 (0.66, 1.04)	1.15 (0.92, 1.44)	**1.39 (1.09, 1.78)∗**

Note: complete table presented in Supplemental Table 8.

Abbreviations: 2′FL, 2′-fucosyllactose; 6′SL, 6′-sialyllactose; BH, Benjamin–Hochberg; CI, confidence interval; DFLac, difucosyllactose; FCT, fecal community type; FDSLNH, fucodisialyllacto-N-hexaose; HMO, human milk oligosaccharide; LNFP II, lacto-N-fucopentaose II; LNFP III, lacto-N-fucopentaose III; OR, odds ratio.

1 To support interpretation of the z-score standardized HMO concentration results, Supplemental Table 1 reports the median, mean, and SD values for each HMO.

2 ORs and corresponding 95% CIs are reported per 1 SD increase in *z*-score standardized HMO concentrations. For maternal secretor status, ORs (95% CIs) are reported based on unstandardized values.

3 OR >1 signifies increased odds of being classified in the first FCT category relative to the reference category listed second.

4 Asterisk indicates statistical significance (*P* < 0.05), and bold values represent BH-corrected statistical significance (*P* < 0.05).

At 13 mo, 13M-FCT1 (*n =* 167 infants) and 13M-FCT4 (*n =* 105 infants) were characterized by *Bacteroides* and *Faecalibacterium*, 13M-FCT2 (*n =* 139 infants) by *Blautia* and *Bifidobacterium*, and 13M-FCT3 (*n =* 111 infants) by *Ruminococcus gnavus group* and *Bacteroides* (Figure 2). 13M-FCT3 was significantly different compared with the other FCTs, showing the lowest alpha diversity and distinct beta diversity characterized by the greatest dissimilarity from the other groups (Figure 2D and F). Birth mode and birth weight, previous deliveries, number of sequencing reads, maternal prepregnancy BMI, and neonatal antibiotic exposure (Supplemental Table 5), as well as HMO composition (Table 3), were associated with the FCTs at the 13-mo time point. The FCTs were more internally uniform at the 13-mo time point compared with the 3-mo time point (Figure 2E and F; median Bray–Curtis dissimilarities 0.77 and 0.87, respectively).

**TABLE 3:** Significant association of *z*-score transformed HMO concentrations^1^ with FCTs in 13-mo-old (*n =* 522) infants assessed with covariate-adjusted multinomial logistic regression models

	FCT types 13-mo OR (95% CI)^2^					
	13M-FCT2 vs. 13M-FCT1^3^	13M-FCT3 vs. 13M-FCT1	13M-FCT4 vs. 13M-FCT1	13M-FCT3 vs. 13M-FCT2	13M-FCT4 vs. 13M-FCT2	13M-FCT4 vs. 13M-FCT3
Total HMO concentration	1.14 (0.9, 1.45)	1.28 (0.98, 1.68)	0.95 (0.75, 1.20)	1.12 (0.84, 1.49)	0.83 (0.64, 1.08)	0.74 (0.5, 1.00)∗^4^
HMO-bound fucose	1.09 (0.86, 1.38)	1.19 (0.91, 1.56)	0.89 (0.70, 1.12)	1.10 (0.83, 1.46)	0.82 (0.63, 1.06)	0.74 (0.56, 0.99)∗
3FL	1.09 (0.87, 1.38)	1.14 (0.89, 1.46)	0.81 (0.61, 1.07)	1.04 (0.82, 1.33)	0.74 (0.55, 0.99)∗	0.71 (0.52, 0.96)∗
LNFP I	1.11 (0.87, 1.42)	1.38 (1.08, 1.76)	1.31 (1.01, 1.68)∗	1.24 (0.96, 1.59)	1.17 (0.9, 1.54)	0.95 (0.73, 1.23)
LNFP II	0.91 (0.72, 1.14)	0.74 (0.57, 0.95)∗	0.87 (0.68, 1.11)	0.81 (0.62, 1.06)	0.96 (0.73, 1.25)	1.18 (0.88, 1.57)
LNFP III	1.33 (0.65, 2.74)	0.51 (0.21, 1.23)	0.77 (0.34, 1.74)	0.39 (0.16, 0.95)∗	0.58 (0.24, 1.38)	1.50 (0.56, 4.02)
LSTb	0.86 (0.67, 1.1)	0.99 (0.78, 1.26)	1.20 (0.95, 1.52)	1.15 (0.88, 1.51)	1.40 (1.07, 1.84)∗	1.22 (0.94, 1.57)
LNH	1.18 (0.89, 1.57)	0.76 (0.53, 1.09)	0.78 (0.54, 1.13)	0.64 (0.44, 0.93)∗	0.66 (0.45, 0.97)∗	1.03 (0.67, 1.58)
FDSLNH	0.90 (0.72, 1.13)	0.70 (0.52, 0.93)∗	0.97 (0.76, 1.23)	0.78 (0.58, 1.05)	1.08 (0.83, 1.41)	1.38 (1.01, 1.9)∗

Note: complete table presented in Supplemental Table 8.

Abbreviations: 3FL, 3-fucosyllactose; BH, Benjamin–Hochberg; CI, confidence interval; FCT, fecal community type; FDSLNH, fucodisialyllacto-N-hexaose; HMO, human milk oligosaccharide; LNH, lacto-N-hexaose; LNFP I, lacto-N-fucopentaose I; LNFP II, lacto-N-fucopentaose II; LNFP III, lacto-N-fucopentaose III; LSTb, sialyl-LNT b; OR, odds ratio.

1 To support interpretation of the *z*-score standardized HMO concentration results, Supplemental Table 3 reports the median, mean, and SD values for each HMO.

2 ORs and corresponding 95% CIs are reported per 1 SD increase in *z*-score standardized HMO concentrations.

3 An OR >1 signifies increased odds of being classified in the first FCT category relative to the reference category listed second.

4 Asterisk indicates statistical significance (*P* < 0.05) and bold values represent BH-corrected statistical significance (*P* < 0.05).

### Associations of maternal secretor status and HMO summary measures with infant gut microbiota

**FIGURE 3:**
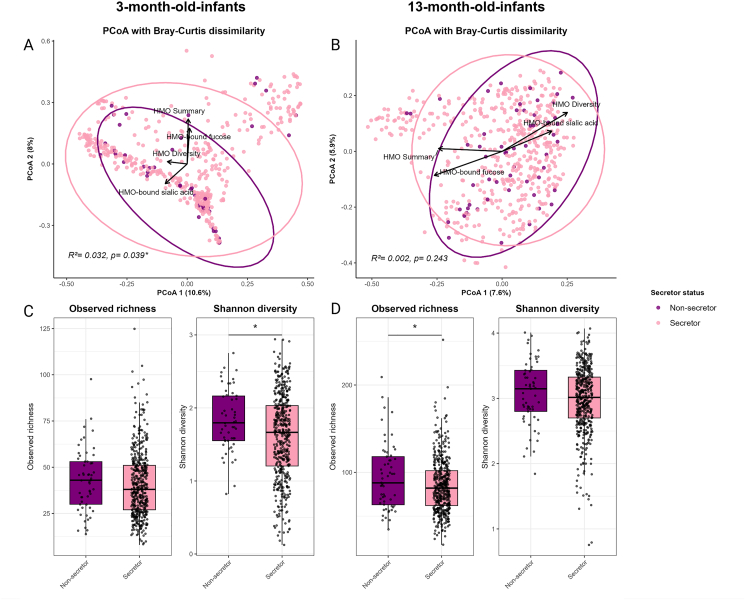
Association between maternal secretor status and infant gut microbiota diversity measures in 3- and 13-mo-old infants. Beta diversity visualized with Principal Coordinates Analysis (PCoA) for ASV-level data using Bray–Curtis distances. The *R*^2^ and *P* values are derived from covariate-adjusted PERMANOVA models. The ellipses represent maternal secretor status, and arrows represent the direction and strength of the correlation between HMO summary measures and microbiota composition (scaled 3× for visualization) in (A) 3-mo-old and (B) 13-mo-old infants. Alpha diversity calculated using Shannon diversity index and observed species richness between infants receiving secretor and nonsecretor milk in (C) 3-mo-old and (D) 13-mo-old infants. The stars indicate statistical significance (*P* value < 0.05) in covariate-adjusted linear model (Table 4). The covariates used in the models were birth mode (vaginal or cesarean section delivery), parity (primiparous or multiparous), and maternal prepregnancy BMI. ASV, amplicon sequence variant; HMO, human milk oligosaccharide; PERMANOVA, permutational multivariate analysis of variance.

Maternal secretor status was associated with gut microbiota beta diversity (Figure 3, *R*^2^ = 0.0032, *P* < 0.05, covariate-adjusted PERMANOVA, Bray–Curtis distance on ASV level) and FCTs at 3 mo. Infants receiving secretor milk were more likely to cluster in 3M-FCT2 compared with 3M-FCT3 (Table 2) and exhibited lower Shannon diversity (Table 4) and higher *Bifidobacterium* abundance (LinDA and ALDEx2, adjusted *P* value < 0.05) compared with infants receiving nonsecretor milk. By 13 mo, the associations were no longer observed, with the exception of a reduction in species richness observed in infants receiving secretor milk (Table 4).

**TABLE 4:** Significant association of secretor status and *z*-score transformed HMO concentrations^1^ with Shannon diversity and observed richness in 3-mo-old (*n =* 517) and 13-mo-old (*n =* 522) infants assessed with covariate-adjusted linear model

	Alpha diversity 3-mo-old (*n =* 517)		Alpha diversity 13-mo-old (*n =* 522)	
	Shannon diversity *b* (95% CI)^2^	Observed richness *b* (95% CI)	Shannon diversity *b* (95% CI)	Observed richness *b* (95% CI)
Secretor (yes)	**–0.239 (–0.397, –0.081)**^3^	–2.186 (–7.155, 2.783)	–0.111 (–0.248, 0.026)	**–11.103 (–19.737,–2.469)**
HMO diversity	0.033 (0.000, 0.066)	0.766 (–0.269, 1.801)	0.020 (–0.009, 0.048)	1.144 (–0.659, 2.946)
Total HMO concentration	**–0.074 (–0.122, –0.026)**	–0.606 (–2.124, 0.912)	–0.047 (–0.090, –0.005)	**–4.108 (–6.786, –1.429)**
HMO-bound sialic acid	**0.094 (0.046, 0.142)**	**2.688 (1.185, 4.192)**	0.040 (–0.003, 0.083)	**4.148 (1.466, 6.831)**
HMO-bound fucose	**–0.068 (–0.117, –0.020)**	–0.606 (–2.123, 0.912)	–0.040 (–0.082, 0.003)	**–3.573 (–6.258, –0.888)**
2′FL	–**0.086 (–0.134, –0.038)**	–1.286 (–2.801, 0.229)	–0.049 (–0.091, –0.006)	**–3.693 (–6.373, –1.012)**
3FL	–0.002 (–0.051, 0.047)	1.372 (–0.143, 2.886)	–0.014 (–0.056, 0.029)	–2.749 (–5.436, –0.061)
3′SL	0.050 (0.001, 0.098)	**2.601 (1.097, 4.105)**	–0.002 (–0.045, 0.041)	–0.611 (–3.314, 2.092)
DFLac	0.034 (–0.015, 0.082)	**2.475 (0.971, 3.979)**	0.017 (–0.026, 0.060)	0.957 (–1.740, 3.655)
6′SL	**0.067 (0.019, 0.116)**	1.503 (–0.011, 3.016)	0.012 (–0.030, 0.055)	2.258 (–0.430, 4.947)
LNFP II	**0.064 (0.016, 0.112)**	0.358 (–1.162, 1.877)	0.036 (–0.006, 0.079)	2.162 (–0.528, 4.853)
LSTb	**0.101 (0.053, 0.149)**	**3.096 (1.600,4.592)**	0.025 (–0.017, 0.068)	2.196 (–0.494, 4.885)
DSLNT	0.049 (0.001, 0.098)	1.737 (0.225, 3.250)	0.032 (–0.010, 0.075)	2.620 (–0.075, 5.314)
FDSLNH	0.027 (–0.021, 0.076)	–0.216 (–1.734, 1.302)	0.045 (0.002, 0.087)	**3.858 (1.178, 6.537)**

Note: complete table presented in Supplemental Table 9.

Abbreviations: 2′FL, 2′-fucosyllactose; 3FL, 3-fucosyllactose; 3′SL, 3′-sialyllactose; 4DFLac, difucosyllactose; 6′SL, 6′-sialyllactose; BH, Benjamin–Hochberg; CI, confidence interval; DSLNT, disialyllacto-N-tetraose; FDSLNH, fucodisialyllacto-N-hexaose; HMO, human milk oligosaccharide; LNFP II, lacto-N-fucopentaose II; LSTb, sialyl-LNT b.

1 To support interpretation of the *z*-score standardized HMO concentration results, Supplemental Tables 1 and 3 report the median, mean, and SD values for each HMO.

2 The effect estimates were expressed as (*b*) and the corresponding 95% CI per 1 SD increase in *z*-score transformed HMO concentration. For maternal secretor status and HMO diversity, the *b* (95% CI) values were expressed for nonstandardized values.

3 Bold values represent BH-corrected statistical significance.

Both total HMO and HMO-bound fucose concentration displayed similar associations with gut microbiota metrics. At 3 mo of age, a 1 SD increase in z-score transformed total HMO and HMO-bound fucose concentrations associated with decreased odds of the infant belonging to 3M-FCT3 compared with 3M-FCT2 (Table 2). Infants receiving higher total HMO and HMO-bound fucose concentrations showed a decrease in Shannon diversity at 3 mo (Table 4). At 13 mo, these HMO measures were associated with decreased odds of belonging to 13M-FCT4 compared with 13M-FCT3 (Table 3) and decreased observed species richness (Table 4). Notably, HMO-bound fucose was also associated with the gut microbiota community composition at 13-mo time point (*R* = 0.003, *P <* 0.05, covariate-adjusted PERMANOVA, Bray–Curtis distance on ASV level). After stratifying by maternal secretor status, the associations did not remain significant in infants fed secretor milk (Supplemental Tables 6 and 7). In contrast, HMO-bound sialic acid showed a positive relationship with Shannon diversity at 3 mo and observed species richness at 13 mo (Table 4). Associations at 3 mo remained significant but diminished at 13 mo, when the analyses were stratified by maternal secretor status (Supplemental Tables 6 and 7).

### Associations between individual HMOs and infant gut microbiota

**FIGURE 4:**
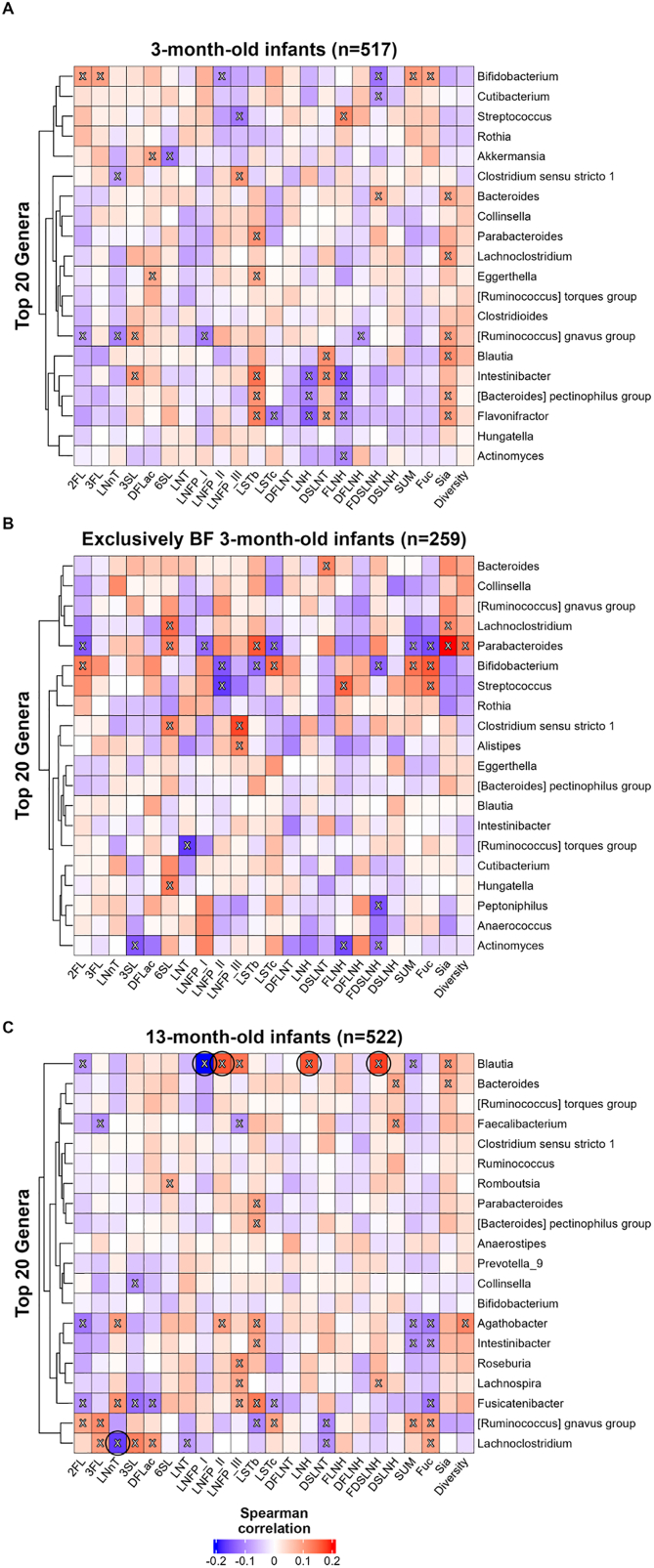
Heatmap showing Spearman correlation between top 20 bacterial genera and *z*-score transformed HMO concentrations measured at the infant age of 3 mo in (A) 3-mo-old infants (*n =* 517), (B) exclusively breastfed (BF) 3-mo-old infants (*n =* 259), and (C) 13-mo-old infants (*n =* 522). Rows are ordered using hierarchical clustering (Euclidean distance, complete linkage). Cells marked with “X” indicate statistically significant (*P* value < 0.05) and circled “X” BH-corrected statistically significant (*P* value < 0.05) Spearman correlations. 2′FL, 2′-fucosyllactose; 3FL, 3-fucosyllactose; 3′SL, 3′-sialyllactose; 6′SL, 6′-sialyllactose; BH, Benjamin–Hochberg; DFLac, difucosyllactose; DFLNH, difucosyl-lacto-N-hexaose; DFLNT, difucosyl-lacto-N-tetraose; Diversity, HMO diversity; DSLNH, disialyl-lacto-N-hexaose; DSLNT, disialyl-lacto-N-tetraose; FDSLNH, fucodisialyl-lacto-N-hexaose; FLNH, fucosyl-lacto-N-hexaose; Fuc, HMO-bound fucose; HMO, human milk oligosaccharide; LNH, lacto-N-hexaose; LNFP I, lacto-N-fucopentaose I; LNFP II, lacto-N-fucopentaose II; LNFP III, lacto-N-fucopentaose III; LNnT, lacto-N-neotetraose; LNT, lacto-N-tetraose; LST b, sialyl-lacto-N-tetraose b; LSTc, sialyl-lacto-N-tetraose c; Sia, HMO-bound sialic acid; SUM, total concentration of HMOs.

LSTb and DSLNT, both belonging to the same HMO functional group internal α-2-6-sialylated HMOs, showed positive correlations with each other in human milk (Supplemental Figure 5) and displayed similar associations with infant gut microbiota. No significant association was observed between the gut microbiota beta diversity and individual HMOs using both variable selection (ordistep, *P <* 0.05) and subsequent covariate-adjusted PERMANOVA analysis in the whole cohort of 3-mo-old infants. However, DSLNT concentration in human milk was associated with gut microbiota beta diversity (*R*^2^ = 0.0063, *P <* 0.05, covariate-adjusted PERMANOVA, Bray–Curtis distance on ASV level) in exclusively breastfed infants at 3 mo. In addition, exclusively breastfed infants whose mothers’ milk contained higher DSLNT concentrations had reduced odds of clustering in 3M-FCT2 [odds ratio (OR): 0.68, 95% confidence interval (CI): 0.48, 0.94] and 3M-FCT3 (OR: 0.65, 95% CI: 0.44, 0.96) compared with 3M-FCT1 dominated by both *Bifidobacterium* and *Bacteroides* (Supplemental Table 10). These associations remained significant in infants fed secretor milk after stratifying by maternal secretor status (Supplemental Table 11). Among exclusively breastfed infants, higher LSTb concentration was associated with decreased odds of clustering to 3M-FCT2 compared with 3M-FCT1 (OR: 0.69, 95% CI: 0.49, 0.97) (Supplemental Table 10), with a significant association remaining in infants fed secretor milk (Supplemental Table 11). Notably, LSTb associated positively with both Shannon diversity and observed species richness in 3-mo-old infants (Table 4) and correlated positively with *Parabacteroides, Bacteroides pectinophilus group*, and *Intestinibacter* at both the 3-mo time point and in the 13-mo time point (Figure 4, Spearman correlation, *P <* 0.05).

Concentrations of LNFP II, LNFP III, and FDSLNH in human milk exhibited a positive correlation with each other, in contrast to a negative correlation with LNFP I (Supplemental Figure 5). A similar trend in the associations was observed between these HMOs and the gut microbiota metrics. Specifically, LNFP II and FDSLNH showed similar associations with gut microbiota at both time points. At 3 mo, higher concentrations of LNFP II and FDSLNH demonstrated a negative correlation with *Bifidobacterium* (Figure 4, Spearman correlation, *P <* 0.05) and increased odds of infants clustering in 3M-FCT3 compared with 3M-FCT2, characterized by the high levels of *Bifidobacterium* (Table 2). At 13 mo, higher concentrations of LNFP II and FDSLNH were associated with decreased odds of clustering in 13M-FCT3 compared with 13M-FCT1 (Table 3). In stratified analysis, the associations remained significant with the infants receiving secretor milk in 13-mo time point (Supplemental Table 7). Similar to LNFP II and FDSLNH, a higher concentration of LNFP III was associated with increased odds of infant clustering into 3M-FCT3 compared with 3M-FCT2 at 3 mo (Table 2). LNFP I exhibited associations with gut microbiota only at 13 mo, showing increased odds of infant clustering in 13M-FCT3 compared with 13M-FCT1 (Table 3). LNFP II, FDSLNH, and LNFP III were positively correlated with the genus *Blautia* at 13 mo, whereas LNFP I showed a negative correlation (Figure 4, Spearman correlation, adjusted *P <* 0.05). Notably, only FDSLNH demonstrated a significant association with gut microbiota beta diversity (*R*^2^ = 0.0032, *P* < 0.05, covariate-adjusted PERMANOVA, Bray–Curtis distance on ASV level) together with LNnT (*R*^2^ = 0.0029, *P* < 0.05, covariate-adjusted PERMANOVA, Bray–Curtis distance on ASV level) at 13 mo.

Associations between maternal HMO composition and infant gut microbiota differed between exclusively breastfed infants (Supplemental Table 11) and with the whole cohort on 3-mo-old infants (Supplemental Table 6). As illustrated in Figure 4, exclusively breastfed infants exhibited stronger Spearman correlation coefficients between HMOs and the top 20 bacterial genera when compared with the entire cohort of 3-mo-olds. Notably, these correlations were primarily observed with the genera *Parabacteroides* and *Bifidobacterium*. In addition, HMOs showed a more pronounced association with the FCTs in exclusively breastfed infants, also showing a greater number of associations compared with the entire cohort of 3-mo-olds (Supplemental Table 10). Furthermore, in exclusively breastfed infants, the significant associations between HMO concentrations and bacterial alpha diversity were mainly detected with the Shannon diversity index (Supplemental Table 12), whereas in the broader 3-mo cohort, HMOs associated with both Shannon diversity and species richness (Table 4). However, many of the associations observed in the full 3-mo cohort were also observed among exclusively breastfed infants, providing a sensitivity analysis for feeding mode at this time point (Supplemental Tables 10 and 12). At 13 mo, sensitivity analyses adjusting for a variable capturing both current breastfeeding status and time since breastfeeding cessation produced results consistent with the unadjusted models (Supplemental Tables 10 and 12). The beta diversity findings were likewise unchanged (data not shown).

### HMO and cesarean section delivery

To assess the role of HMOs in restoring the gut microbiota of infants after cesarean section delivery, we analyzed the subcohort of cesarean-born infants (*n =* 55) and examined their distribution across FCTs at the 3-mo time point. Among cesarean-born infants, the highest proportions were observed in 3M-FCT3 (25/55 = 46%) and 3M-FCT2 (27/55 = 49%), with only 5% in 3M-FCT1. This distribution differed significantly from vaginally born infants (Supplemental Table 4, Chi-squared test, *P* < 0.05). We compared the HMO composition of mothers whose infants were classified into 3M-FCT3 with those whose infants were classified into 3M-FCT1 and 3M-FCT2 combined (Supplemental Figure 6 and Supplemental Table 13). Among cesarean-born infants, 42% were exclusively breastfed. Infants classified into 3M-FCT1 and 3M-FCT2 combined were more likely to be exclusively breastfed compared with those in 3M-FCT3 (Supplemental Table 13).

No individual HMO was significantly associated with FCTs in covariate-adjusted logistic regression models at 3 mo (Supplemental Table 14). However, we did see an association with maternal secretor status and FCTs. Cesarean-born infants receiving secretor milk had 80% lower odds of belonging to 3M-FCT3 compared with the combined 3M-FCT1 and 3M-FCT2 group (Supplemental Table 14, OR: 0.20, 95% CI: 0.027, 0.98). Notably, a similar but less pronounced association was observed in the entire cohort of 3-mo-old infants (logistic regression, OR: 0.41, 95% CI: 0.22, 0.73). The association between maternal secretor status and FCTs in cesarean-born infants diminished by the 13-mo time point (Supplemental Table 15, Fisher’s exact test *P* < 0.05). At this time point, FCTs among cesarean-born infants were primarily influenced by the breastfeeding status (Supplemental Table 15, Fisher’s exact test *P* < 0.05), a trend that was not observed in the full cohort of 13-mo-old infants (Supplemental Table 5, Chi-squared test *P* < 0.05).

## Discussion

We identified multiple associations between HMOs and the infant gut microbiota during breastfeeding at 3 mo, and found that early-life exposure to HMOs is associated with gut microbiota composition even at 13 mo. Notably, HMO-bound sialic acid, as well as individual sialylated HMOs like DSLNT and LSTb, exhibited multiple associations with gut microbiota metrics at 3 mo, whereas HMO-bound fucose demonstrated more lasting associations observed at 13 mo. HMO-bound sialic acid associated with increased bacterial alpha diversity and *Bacteroides* abundance, whereas HMO-bound fucose associated with decreased alpha diversity and increased *Bifidobacterium* abundance. Maternal secretor status is associated with the gut microbiota composition in 3-mo-old infants and with patterns reflecting microbiota restoration in cesarean-born infants.

To comprehensively study the associations between maternal HMO composition and infant gut microbiota, we applied multiple complementary metrics to describe the microbiota composition, diversity, and taxon abundances. In addition to alpha diversity (within-sample diversity), beta diversity (between-sample diversity), and individual taxa, we used FCTs to get a more biologically interpretable view of HMO–microbiota associations. At 3 mo, 2 typical FCTs emerged, dominated by *Bifidobacterium* and *Bacteroides*. Both profiles are commonly linked to breastfeeding and healthy gut microbiota development in infancy [41,42]. In contrast, the third FCT reflected a more atypical profile, characterized by minimal *Bifidobacterium* abundance and elevated levels of *Clostridium sensu stricto* 1 [43]. By 13 mo, FCTs converged toward more similar community structures, consistent with microbiota maturation [42]. The gut microbiota community composition was most similar between FCTs defined by the abundance of the genera *Bacteroides* and *Faecalibacterium*. Notably, we observed 1 FCT that significantly differed from others, displaying the lowest alpha diversity and highest *Ruminococcus gnavus group* abundance.

Our results highlighted the association between maternal secretor status and infant gut microbiota at 3-mo-old infants. Consistent with previous studies, we observed that infants breastfed by secretor mothers harbor higher levels of *Bifidobacterium*, along with decreased alpha diversity and shifts in community composition, compared with those breastfed by nonsecretor mothers [44,45]. This aligns with the established understanding that specific *Bifidobacterium* strains can efficiently use HMOs [46], particularly 2′FL, which is high in secretor mothers [47,48]. Such metabolic advantages allow *Bifidobacterium* to dominate the intestinal microbiota of infants breastfed by secretor mothers, elucidating the associations observed in our study and others. Notably, several associations between maternal secretor status and infant gut microbiota diminished by 13 mo of age. Although some studies suggest that maternal secretor status may influence the gut microbiota composition for up to 3 y [49], our findings indicate a weakening relationship at 13 mo of age, when the infant diet is typically more diverse. It is important to acknowledge that the associations between maternal secretor status and infant gut microbiota may vary across populations [50,51] or under specific conditions like cesarean section [14]. In addition, some studies report no association between maternal secretor status and infant gut microbiota [15,52,53].

We observed patterns in the associations between HMO summary measures and infant gut microbiota that corroborate and extend prior research. First, we found that associations between HMO-bound sialic acid and infant gut microbiota tended to persist in infants receiving milk from secretor mothers in stratified analyses, whereas associations with HMO-bound fucose were more frequently secretor-status dependent. This is biologically plausible because maternal secretor status (FUT2 genotype) primarily influences the presence and abundance of fucosylated HMOs, and HMO sialylation is relatively independent of the secretor trait [12,45]. Second, we observed a positive association between HMO-bound sialic acid and negative association between HMO-bound fucose with infant gut microbiota alpha diversity. This observation is not directly supported by previous literature, but is in line with earlier studies showing that fucosylated HMOs often promote the growth of *Bifidobacterium* [47,48], which can outcompete other bacteria and become dominant in the gut community, thereby reducing overall diversity. Recent study in piglets also demonstrated how degradation of sialylated HMOs can deliver sialic acid to commensal microbes and contribute to the establishment of cross-feeding networks, which may support microbial diversity and stability over time [54]. Finally, we observed an association between total HMO concentration and HMO-bound fucose with *Bifidobacterium* in early infancy and with *Ruminococcus gnavus group* (*R. gnavus* group) in late infancy. During breastfeeding, *Bifidobacterium* efficiently uses fucosylated HMOs, limiting access for other microbes. With the changing diet and increasing microbial diversification, the competitive landscape shifts, creating opportunities for other fucose-metabolizing taxa such as *R. gnavus* to expand [55]. Previous literature highlights the importance of the timely colonization of *R. gnavus* group in relation to allergic diseases. Premature colonization, as a result of early cessation of breastfeeding, has been linked to increased risk of allergic diseases, whereas later colonization may confer protection [41,56,57]. Our observations suggest that total HMO concentration and fucosylated HMOs may contribute to the ecological transition of the infant gut, supporting *Bifidobacterium* dominance in early infancy and enabling selective expansion of taxa like *R. gnavus* as the microbiota matures.

Individual HMOs, including LSTb and DSLNT, both belonging to the same HMO functional group with sialic acid bound to the internal N-acetylglucosamine (GlcNAc) in α-2-6-linkage, displayed similar associations with infant gut microbiota. Among these HMOs, DSLNT has received particular attention due to its reported protective role against necrotizing enterocolitis (NEC); higher DSLNT concentrations in human milk have been associated with a lower incidence of NEC in preterm infants [[58], [59], [60]]. In our study, DSLNT was the only HMO associated with gut microbiota community composition at 3 mo, detectable only in exclusively breastfed infants. This pattern suggests that the microbiota-related associations of DSLNT may be most evident when other feeding-related confounders are minimized. Higher DSLNT concentrations were linked to FCT enriched in *Bacteroides*, consistent with its positive correlation with this genus among exclusively breastfed infants. This finding is in line with our broader observations for HMO-bound sialic acid, which correlated positively with the genus *Bacteroides*. Previous work similarly shows that infants harboring bacteria capable of degrading sialylated HMOs often display *Bacteroides*-dominated FCTs [54]. The known ability of Bacteroides spp. to use sialylated HMOs through mucus-degradation pathways further supports our findings [61].

Compared with DSLNT, LSTb has received less attention, yet our findings suggest that it may play an interesting and potentially biologically meaningful role during early infancy. We observed consistent associations between LSTb and specific gut microbiota genera, *Bacteroides pectinophilus group* and *Parabacteroides*, through early and late infancy. Both genera are known primary degraders within microbial cross‑feeding networks, important for maintaining a diverse and balanced gut microbiota [62,63]. Notably, LSTb displayed the strongest association with microbiota alpha diversity among all HMOs examined, with higher LSTb concentrations linked to increased bacterial diversity in early infancy. These findings suggest that LSTb may support microbial interactions that contribute to a more diverse gut ecosystem, particularly during early infancy. Further mechanistic work is needed to build upon and confirm these observations.

Associations between HMOs and the gut microbiota were strongest during breastfeeding, particularly among exclusively breastfed infants. At the 13-mo time point, associations were still detectable, but few remained significant after correcting for multiple comparions. Notably, associations between HMOs and overall community composition were more pronounced in late infancy than in early infancy. The individual HMOs associated with community composition in late infancy were FDSLNH and LNnT, as well as the total HMO-bound fucose levels. There is little to no evidence about the association between FDSLNH and infant gut microbiota in previous literature, but our findings highlight its associations with various gut microbiota metrics, especially during late infancy. LNnT, on the one hand, is among the abundant HMOs found in human milk and often added to the infant formula together with 2′FL to support the growth of beneficial gut bacteria [64].

Previous literature shows that breastfeeding may help mitigate microbiota alterations associated with cesarean birth [65,66]. Cesarean-born infants appear to particularly benefit from fucosylated oligosaccharides produced by secretor mothers [14] and from HMO supplementation with 2′FL and LNnT [39]. Although we found that receiving milk from secretor mothers was associated with a gut microbiota composition resembling that of vaginally delivered infants, no individual HMOs demonstrated significant microbiome-rebalancing effects on their own. By 13 mo, the impact of maternal secretor status had also diminished, with duration of breastfeeding playing a more important role in shaping the gut microbiota profiles. Consistent with our findings, prolonged breastfeeding has previously been shown to associate with the gut microbiota composition of cesarean-born infants, but not that of vaginally born infants at 6 mo of age, highlighting its importance particularly in cesarean-born infants [67].

Although we did not assess any health implications in our study, it is essential to recognize the potential lasting ramifications of these early microbial imbalances on immune system development and, consequently, other health outcomes. The early months of life are critical for microbiota maturation, which in turn plays a pivotal role in immune development [68]. Previous studies outline a possible microbiota-mediated association between cesarean section delivery and elevated risks of immune-related disorders [69,70]. Our findings, as well as those of others [14], demonstrate that cesarean-born infants receiving milk from nonsecretors show more cesarean-related microbiota patterns compared with infants breastfed by secretor mothers. This highlights a potential avenue for interventions aimed at supporting microbiota development in cesarean-born infants, particularly in relation to maternal secretor status. Our findings demonstrate that possible interventions should not solely focus on HMO supplementation, because cesarean-born infants often lack beneficial HMO-using bacteria such as *Bacteroides* and *Bifidobacterium*. Instead, supplementation should incorporate live bacteria, like *Bifidobacterium* species, alongside bifidogenic HMOs. Further studies with larger sample sizes are needed to confirm this observation.

Key strengths of this study include the large STEPS Study cohort with detailed metadata and paired maternal milk and infant fecal samples. Assessing 2 time points allowed us to examine HMO–microbiota associations both during breastfeeding (3 mo) and later in infancy (13 mo). Stratified analyses by maternal secretor status and infant feeding mode strengthened the validity of our findings. In addition, the exclusion of preterm infants, population-based design, and homogeneity of the study cohort enhance the generalizability of our findings to other industrialized Western populations. Limitations of this study include the lack of validated dietary data beyond breastfeeding, cross-sectional study design, and potential selection bias in the study population, introduced by selection based on sample availability. In addition, only 1 milk sample per mother was available, and the maternal Lewis status was not determined. The small number of cesarean-born infants limited statistical power in subgroup analyses, and the use of 16S rRNA sequencing restricted strain level and functional analysis that would have given more insight into the metabolic capabilities for HMO use.

In conclusion, our study demonstrates age-dependent and structure-specific associations between HMOs and infant gut microbiota, extending beyond breastfeeding. Specifically, we demonstrated how the associations between HMO-bound sialic acid and HMO-bound fucose and infant gut microbiota differ from each other, with HMO-bound sialic acid increasing the bacterial diversity and HMO-bound fucose increasing the abundance of genus *Bifidobacterium* in the infant gut. Specifically, we highlighted LSTb contributing to increased alpha diversity and an increased number of primary degraders in the infant gut. Our findings also reveal that maternal secretor status is associated with multiple gut microbiota metrics at 3-mo-old infants, with diminishing associations in late infancy.

## Author contributions

The authors’ responsibilities were as follows – MO, HL, MT, ML, SR: designed research; HL, MO: conducted research; MO, MT, AJ: analyzed data; MO: wrote the paper; and all authors: contributed to writing and critically revising the manuscript and read and approved the final manuscript.

## Data availability

Because of Finnish federal legislation, the research data cannot be made available online, but data can potentially be shared with a Material Transfer Agreement as part of a research collaboration. Requests for collaboration can be sent to the Executive Committee of the STEPS Study. Please contact principal investigator Hanna Lagström (hanlag@utu.fi).

## Declaration of generative AI and AI-assisted technologies in the writing process

During the preparation of this work, the author Minka Ovaska used ChatGPT for R code correction and text processing for possible grammatical errors according to the guidelines on the responsible use of generative AI in research (European Commission, 2024). After using this tool, the author reviewed and edited the content as needed and took full responsibility for the content of the publication.

## Funding

MO was supported by grant 230052 from the Päivikki and Sakari Sohlberg Foundation. HL was supported by grant 321409 and ML by grant 371390 from the Research Council of Finland. Additionally, HL was supported by Special Governmental grants for Health Sciences Research and ML by Human Diversity consortium, Profi7 program by Research Council of Finland, grant 352727 and NetResilience consortium funded by the Strategic Research Council within the Research Council of Finland (grant numbers 364385 and 364371). The funders had no role in design, execution, or interpretation of the research.

## Conflict of interest

The authors report no conflicts of interest.
