# Supplementary material for: The role of human milk oligosaccharides in shaping and restoring infant gut microbiota: population-based cohort study
Source: Am J Clin Nutr. 2026 Apr 16;123(6):101318. doi: 10.1016/j.ajcnut.2026.101318 (PMC13269344; doi:10.1016/j.ajcnut.2026.101318)
Supplement: Multimedia component 1 [file mmc1.zip › mmc1.html]

Supplementary methods: The role of human milk oligosaccharides in shaping and restoring infant gut microbiota


Code 

- Show All Code
- Hide All Code

# Supplementary methods: The role of human milk oligosaccharides in shaping and restoring infant gut microbiota

#### Minka Ovaska

#### 2025-10-21

# Load the data and the packages

```
setwd("C:/HMO_1")


packages <- c("mia", "miaViz","ape","readxl","scuttle","ggplot2", "vegan", "tidyverse","dplyr","RColorBrewer", "lubridate", "lavaan", "scater", "patchwork", "DHARMa", "ComplexHeatmap", "shadowtext", "colorRamp2", "bluster", "kableExtra", "emmeans", "ggpubr", "cowplot", "gridGraphics", "tableone", "nnet", "broom","tidyr", "purrr", "PerformanceAnalytics", "pairwiseAdonis", "writexl", "forcats", "ggrepel")


is_installed <- function(pkg) {
  new_pkg <- pkg[!(pkg %in% installed.packages()[, "Package"])]
  if (length(new_pkg)) {
    BiocManager::install(new_pkg, ask = FALSE)
  }
  sapply(pkg, require, character.only = TRUE)
}
is_installed(packages)
```

```
##                  mia               miaViz                  ape 
##                 TRUE                 TRUE                 TRUE 
##               readxl              scuttle              ggplot2 
##                 TRUE                 TRUE                 TRUE 
##                vegan            tidyverse                dplyr 
##                 TRUE                 TRUE                 TRUE 
##         RColorBrewer            lubridate               lavaan 
##                 TRUE                 TRUE                 TRUE 
##               scater            patchwork               DHARMa 
##                 TRUE                 TRUE                 TRUE 
##       ComplexHeatmap           shadowtext           colorRamp2 
##                 TRUE                 TRUE                 TRUE 
##              bluster           kableExtra              emmeans 
##                 TRUE                 TRUE                 TRUE 
##               ggpubr              cowplot         gridGraphics 
##                 TRUE                 TRUE                 TRUE 
##             tableone                 nnet                broom 
##                 TRUE                 TRUE                 TRUE 
##                tidyr                purrr PerformanceAnalytics 
##                 TRUE                 TRUE                 TRUE 
##       pairwiseAdonis              writexl              forcats 
##                 TRUE                 TRUE                 TRUE 
##              ggrepel 
##                 TRUE
```

The data is already in tse object, constructed by combining metadata,
ASV table and taxonomy table.

```
tse_subset_by_03kk_ASV <- readRDS(file = "C:/HMO_1/tse_subset_by_03kk_ASV_HMO" )
tse_subset_by_13kk_ASV <- readRDS(file = "C:/HMO_1/tse_subset_by_13kk_ASV_HMO" )

# make data transformations
tse_subset_by_03kk_ASV <- transformAssay(
     tse_subset_by_03kk_ASV, assay.type = "counts", method = "relabundance")

tse_subset_by_03kk_ASV <- transformAssay(
    x = tse_subset_by_03kk_ASV, assay.type = "relabundance", method = "clr",
    name = "clr", pseudocount=1)

tse_subset_by_13kk_ASV <- transformAssay(
     tse_subset_by_13kk_ASV, assay.type = "counts", method = "relabundance")

tse_subset_by_13kk_ASV <- transformAssay(
    x = tse_subset_by_13kk_ASV, assay.type = "relabundance", method = "clr",
    name = "clr",  pseudocount=1)
```

scale the HMO data using z-score transformation

```
# Vector of variable names
vars_to_scale <- c("X_2FL_nmol", "X_3FL_nmol", "LNnT_nmol", "X_3SL_nmol", "DFLac_nmol", 
                 "X_6SL_nmol", "LNT_nmol", "LNFP_I_nmol", "LNFP_II_nmol", "LNFP_III_nmol", 
                 "LSTb_nmol", "LSTc_nmol", "DFLNT_nmol", "LNH_nmol", "DSLNT_nmol", 
                 "FLNH_nmol", "DFLNH_nmol", "FDSLNH_nmol", "DSLNH_nmol", "SUM_nmol", 
                 "Sia", "Fuc")

# Apply log transformation and create new columns 3mo:
for (var in vars_to_scale) {
  colData(tse_subset_by_03kk_ASV)[[paste0(var, "_scale")]] <- scale(colData(tse_subset_by_03kk_ASV)[[var]])
}

# Apply log transformation and create new columns 13mo
for (var in vars_to_scale) {
  colData(tse_subset_by_13kk_ASV)[[paste0(var, "_scale")]] <- scale(colData(tse_subset_by_13kk_ASV)[[var]])
}

meta3kk <- as.data.frame(colData(tse_subset_by_03kk_ASV))
meta13kk <- as.data.frame(colData(tse_subset_by_13kk_ASV))
```

Create a histrogram showing distribution of library sizes at 3 months
and at 13 months

```
p1 <- ggplot(colData(tse_subset_by_03kk_ASV)) +
        geom_histogram(aes(x = sum/1e3), color = "black", fill = "gray") +
        labs(x = "Library size (thousand reads)", y = "Frequency (n)") +
        theme_classic()

# Order data in increasing order
df <- as.data.frame(colData(tse_subset_by_03kk_ASV)) |>
    arrange(sum) |>
    mutate(proportion = (1:n())/n())

# Create a scatter plot showing how library sizes are distributed
p2 <- ggplot(df, aes(y = proportion, x = sum/1e3)) +
    geom_point() +
    labs(x = "Library size (thousand reads)", y = "Proportion of samples") +
    theme_classic()

p1 + p2
```

```
#################### 13mo 

p1b <- ggplot(colData(tse_subset_by_13kk_ASV)) +
        geom_histogram(aes(x = sum/1e3), color = "black", fill = "gray") +
        labs(x = "Library size (thousand reads)", y = "Frequency (n)") +
        theme_classic()

# Order data in increasing order
df <- as.data.frame(colData(tse_subset_by_13kk_ASV)) |>
    arrange(sum) |>
    mutate(proportion = (1:n())/n())

# Create a scatter plot showing how library sizes are distributed
p2b <- ggplot(df, aes(y = proportion, x = sum/1e3)) +
    geom_point() +
    labs(x = "Library size (thousand reads)", y = "Proportion of samples") +
    theme_classic()

p1b + p2b
```

HMO data characteristics

Correlation of the HMO variables (Supplementary 1)

```
meta_df_corr <- dplyr::select(meta3kk, 
                       c("X_2FL_nmol", "X_3FL_nmol", "LNnT_nmol", "X_3SL_nmol", 
                                                "DFLac_nmol", "X_6SL_nmol", "LNT_nmol", "LNFP_I_nmol", 
                                                "LNFP_II_nmol", "LNFP_III_nmol", "LSTb_nmol", "LSTc_nmol", 
                                                "DFLNT_nmol", "LNH_nmol", "DSLNT_nmol", "FLNH_nmol", 
                                                "DFLNH_nmol", "FDSLNH_nmol", "DSLNH_nmol"))

colnames(meta_df_corr) <- gsub("_nmol$", "", colnames(meta_df_corr))  # Remove '_nmol' at the end
colnames(meta_df_corr) <- gsub("^X_", "", colnames(meta_df_corr))      # Remove 'X_' at the start


chart.Correlation(
  meta_df_corr,
  histogram = TRUE,
  method = c("spearman")
)
```

## Table 1

Descriptive characteristics of the subpopulation of the STEPS Study
mothers and their infants in 3 month and 13-month timepoint, in relation
to maternal secretor status. Sample sizes and percentages are given for
categorical variables and means with standard deviations for continuous
variables. Asterisks show significant associations between maternal
secretor status and child or maternal characteristic (p < 0.05).

3 month

```
meta3kk_df <- dplyr::select(meta3kk, 
                       c("SP",
                         "SYNTYMAPAINO_kg",
                         "KESTOVKPV_num", 
                         "SYNNYTYSTAPA_2luok", 
                         "BMI_aiti", 
                         "Aidinika",
                         "aidinab", 
                         "Antib", 
                         "ika_3kk",
                         "season_3kk", 
                         "dmmclust",
                         "lib_size", 
                         "AIEMMATSYNNYTYKSET_cat",
                         "Secretor",
                         "breastfeeding_3kk_category"
                         ))


# Define continuous and categorical variables
continuous_vars <- c("SYNTYMAPAINO_kg", "KESTOVKPV_num", "Aidinika", "ika_3kk", "lib_size", "BMI_aiti")
categorical_vars <- c("SP","SYNNYTYSTAPA_2luok", "aidinab", "Antib",  "season_3kk","AIEMMATSYNNYTYKSET_cat", "dmmclust", "breastfeeding_3kk_category")

# Combine variables into a single list
vars <- c(continuous_vars, categorical_vars)


# Create table
table_one1 <- CreateTableOne(
  vars = vars,
  strata = "Secretor",  # Groups to compare
  data = meta3kk_df,
  factorVars = categorical_vars,  # Categorical variables
  smd = TRUE,  # Add standardized mean differences
  addOverall = TRUE  # Add overall summary
)

#print(table_one1, showAllLevels = TRUE)
# Get printable version (no quotes or spacing, but keep all content)
table_one_printed <- print(table_one1,
                           quote = FALSE,
                           noSpaces = TRUE,
                           printToggle = FALSE)

# Convert row names to a column called "Variable"
table_one_df3kk <- data.frame(Variable = rownames(table_one_printed), table_one_printed, row.names = NULL)

#Write to Excel
#write_xlsx(table_one_df3kk, "table_one_secretor3kk.xlsx")

#Test statistical significance: 
# Grouping variable
# group_var <- "Secretor"
# 
# Define continuous and categorical variables
# continuous_vars <- c("SYNTYMAPAINO_kg", "KESTOVKPV_num", "BMI_aiti", 
#                      "Aidinika", "ika_3kk", "lib_size")
# 
# categorical_vars <- c("SP", "SYNNYTYSTAPA_2luok", "aidinab", "Antib",  
#                       "season_3kk", "AIEMMATSYNNYTYKSET_cat", "breastfeeding_3kk_category")

#for (var in continuous_vars) {
  #cat("\nKruskal-Wallis test for:", var, "\n")
  #print(kruskal.test(as.formula(paste(var, "~", group_var)), data = meta3kk_df))
#}

# for (var in categorical_vars) {
#   # Filter out missing values for the variable and the group
#   df_clean <- meta3kk_df[
#     !is.na(meta3kk_df[[var]]) & 
#     !is.na(meta3kk_df[[group_var]]), ]
#   
#   cat("\nChi-squared test for:", var, "\n")
#   
#   tbl <- table(df_clean[[var]], df_clean[[group_var]])
#   
#   # Skip variables with no valid data
#   if (sum(tbl) == 0) {
#     cat("Skipping", var, "- table has no data.\n")
#     next
#   }
#   
#   # Run chi-squared test
#   #print(chisq.test(tbl))
# }
```

13 month

```
meta13kk_df <- dplyr::select(meta13kk, 
                       c("SP",
                         "SYNTYMAPAINO_kg",
                         "KESTOVKPV_num", 
                         "SYNNYTYSTAPA_2luok", 
                         "BMI_aiti", 
                         "Aidinika",
                         "aidinab", 
                         "Antib", 
                         "ika_13kk",
                         "season_13kk", 
                         "dmmclust",
                         "lib_size", 
                         "AIEMMATSYNNYTYKSET_cat",
                         "Secretor",
                         "breastfeeding_13kk_category"
                         ))


# Define continuous and categorical variables
continuous_vars <- c("SYNTYMAPAINO_kg", "KESTOVKPV_num", "Aidinika", "ika_13kk", "lib_size", "BMI_aiti")
categorical_vars <- c("SP","SYNNYTYSTAPA_2luok", "aidinab", "Antib",  "season_13kk","AIEMMATSYNNYTYKSET_cat", "dmmclust", "breastfeeding_13kk_category")

# Combine variables into a single list
vars <- c(continuous_vars, categorical_vars)

# Create table
table_one2 <- CreateTableOne(
  vars = vars,
  strata = "Secretor",  # Groups to compare
  data = meta13kk_df,
  factorVars = categorical_vars,  # Categorical variables
  smd = TRUE,  # Add standardized mean differences
  addOverall = TRUE  # Add overall summary
)

#table_one
# Print the table with statistical tests
#print(table_one2, showAllLevels = TRUE)


# Get printable version (no quotes or spacing, but keep all content)
table_one_printed <- print(table_one2,
                           quote = FALSE,
                           noSpaces = TRUE,
                           printToggle = FALSE)

# Convert row names to a column called "Variable"
table_one_df2 <- data.frame(Variable = rownames(table_one_printed), table_one_printed, row.names = NULL)

# Write to Excel
#write_xlsx(table_one_df2, "table_one_secretor2.xlsx")

#Test statistical significance: 
# Grouping variable
# group_var <- "Secretor"
# 
# Define continuous and categorical variables
# continuous_vars <- c("SYNTYMAPAINO_kg", "KESTOVKPV_num", "BMI_aiti", 
#                      "Aidinika", "ika_13kk", "lib_size")
# 
# categorical_vars <- c("SP", "SYNNYTYSTAPA_2luok", "aidinab", "Antib",  
#                       "season_13kk", "AIEMMATSYNNYTYKSET_cat", "breastfeeding_13kk_category")
# 
# for (var in continuous_vars) {
#   cat("\nKruskal-Wallis test for:", var, "\n")
#   print(kruskal.test(as.formula(paste(var, "~", group_var)), data = meta13kk_df))
# }
# 
# 
# for (var in categorical_vars) {
#   # Filter out missing values for the variable and the group
#   df_clean <- meta13kk_df[
#     !is.na(meta13kk_df[[var]]) & 
#     !is.na(meta13kk_df[[group_var]]), ]
#   
#   cat("\nChi-squared test for:", var, "\n")
#   
#   tbl <- table(df_clean[[var]], df_clean[[group_var]])
#   
#   # Skip variables with no valid data
#   if (sum(tbl) == 0) {
#     cat("Skipping", var, "- table has no data.\n")
#     next
#   }
#   
#   # Run chi-squared test
#   print(chisq.test(tbl))
# }
```

# Fecal community types 3 month

## DMM clust genus level

FCTs identified using Dirichlet Multinomial Mixtures (DMM)
clustering

```
tse_subset_by_03kk_ASVs_genus <- agglomerateByRank(tse_subset_by_03kk_ASV, rank = "Genus", agglomerateTree = TRUE)


set.seed(123)

tse_subset_by_03kk_ASVs_genus <- addCluster(
    tse_subset_by_03kk_ASVs_genus,
    assay.type = "counts",
    name = "DMM",
    DmmParam(k = 1:7, type = "laplace"),
    MARGIN = "samples",
    full = TRUE,
    clust.col = "dmmclust")
```

```
p <- plotDMNFit(tse_subset_by_03kk_ASVs_genus, type = "laplace", name = "DMM")
a1 <- p + theme_minimal(base_size = 11)

a1
```

```
# Get the estimates on how much each phyla contributes on each cluster
best_model <- metadata(tse_subset_by_03kk_ASVs_genus)$DMM$dmm[3]
drivers <- as.data.frame(best_model[[1]]@fit$Estimate)

b1 <- plotLoadings(as.matrix(drivers), ncomponents = 3)
b1
```

PCoA with bray curtis

```
tse_subset_by_03kk_ASVs_genus <- transformAssay(tse_subset_by_03kk_ASVs_genus, assay.type = "counts", method = "relabundance")


# Add the MDS dimensions for plotting
tse_subset_by_03kk_ASVs_genus <- runMDS(
    tse_subset_by_03kk_ASVs_genus,
    assay.type = "relabundance",
    FUN = vegan::vegdist,
    method = "bray",
    name = "PCoA_bray")

# Calculate explained variance
e <- attr(reducedDim(tse_subset_by_03kk_ASVs_genus, "PCoA_bray"), "eig")
rel_eig <- e / sum(e[e > 0])


# principal coordinate analysis
df <- reducedDim(tse_subset_by_03kk_ASVs_genus, "PCoA_bray")

# Create a data frame from principal coordinates
bray_pcoa_df <- data.frame(pcoa1 = df[, 1], pcoa2 = df[, 2])

# Create a data frame that contains principal coordinates and DMM information
bray_pcoa_df <- cbind(bray_pcoa_df,
                               dmm_component = colData(tse_subset_by_03kk_ASVs_genus)$dmmclust,
                               birth_mde = colData(tse_subset_by_03kk_ASVs_genus)$SYNNYTYSTAPA_2luok,
                               breastfeeding_category = colData(tse_subset_by_03kk_ASVs_genus)$breastfeeding_3kk_category,
                      Secretor = colData(tse_subset_by_03kk_ASVs_genus)$Secretor)

bray_pcoa_df <- bray_pcoa_df %>%
  mutate(Secretor = ifelse(Secretor == "0", "Non-secretor", "Secretor"))
```

```
bray_dmm_plot <- ggplot(data = bray_pcoa_df,
                        aes(x = pcoa1, y = pcoa2, color = dmm_component, shape = Secretor)) +
    geom_point(size = 4, alpha = 0.8) +  # Increased size and added transparency
    labs(x = paste("PCoA 1 (", round(100 * rel_eig[[1]], 1), "%)", sep = ""),
         y = paste("PCoA 2 (", round(100 * rel_eig[[2]], 1), "%)", sep = ""),
         color = "DMMclust", title = "") + 
    stat_ellipse(aes(group = Secretor), show.legend = TRUE) +  # Updated group aesthetic
    scale_color_manual(name = "DMM Component",
                       breaks = c("1", "2", "3"),
                       values = c("#4D9221", "#35978F" , "#DE77AE" )) +
    theme_minimal() +
    theme(plot.title = element_text(size = 12, hjust = 0.5))

bray_dmm_plot
```

```
fig1 <- cowplot::plot_grid(a1, b1, bray_dmm_plot,nrow = 3, labels = c('A', "B", "C"), align = "hv")

#ggsave("3kk_DMM_new.tiff", width = 15, height = 25, dpi = 300)

#Add dmm clusters to the df
colData(tse_subset_by_03kk_ASV)$dmmclust <- as.data.frame(colData(tse_subset_by_03kk_ASVs_genus))$dmmclust

#saveRDS(tse_subset_by_03kk_ASV, "C:/HMO_1/tse_subset_by_03kk_ASV_HMO")
```

### Fig 2 bar plot

```
metadata <- as.data.frame(colData(tse_subset_by_03kk_ASV)) %>%
  select(sample_ID, dmmclust)

taxonomy <- as.data.frame(rowData(tse_subset_by_03kk_ASV)) %>%
  rownames_to_column(var = "ASV") %>%
  select(ASV,Genus)

otu_counts <- as.data.frame(assay(tse_subset_by_03kk_ASV, "counts")) %>%
  rownames_to_column(var = "ASV") %>%
  tidyr:::pivot_longer(cols = 2:518, names_to = "sample_ID", values_to = "count")
```

```
otu_red_abund <- dplyr::inner_join(metadata, otu_counts, by= "sample_ID") %>%
  dplyr::inner_join(., taxonomy, by= "ASV") %>%
  dplyr::group_by(sample_ID) %>%
  mutate(rel_abund = count/ sum(count))

taxon_rel_abund <- otu_red_abund %>%
  group_by(sample_ID, dmmclust, Genus) %>%
  summarise(rel_abund = sum(rel_abund), .groups = "drop") %>%
  dplyr::group_by(dmmclust, Genus) %>%
  summarise(mean_rel_abund = 100*mean(rel_abund, na.rm = TRUE), .groups = "drop")


taxon_pool <- taxon_rel_abund %>%
  group_by(Genus)%>%
  summarize(pool = max(mean_rel_abund) < 3, .groups = "drop")


# Define the desired order of taxa
taxon_order <- c("Bifidobacterium", "Bacteroides", "Clostridium sensu stricto 1", "Collinsella", 
                 "Parabacteroides", "[Ruminococcus] gnavus group","Blautia","Other")


bar_chart <- dplyr::inner_join(taxon_rel_abund, taxon_pool, by = "Genus") %>%
  mutate(Genus = if_else(pool, "Other", Genus),
         Genus = factor(Genus, levels = taxon_order)) %>%
  group_by(dmmclust, Genus) %>%
  summarize(mean_rel_abund = sum(mean_rel_abund), .groups = 'drop') %>%
  ggplot(aes(x = dmmclust, y = mean_rel_abund, fill = Genus)) +
  labs(x = "", y = "Mean relative abundance (100%)") +
  geom_col() +
  scale_fill_manual(values = c("#8C510A", "#BF812D", "#DFC27D", "#F6E8C3",
                               "#80CDC1", "#35978F", "#01665E", "#003C30")) +
  theme_pubr(base_size = 15)

bar_chart
```

```
# save main plot
# bar_chart_no_legend <- bar_chart + theme(legend.position = "none")
# ggsave("bar_chart_3mo_no_legend.png", bar_chart_no_legend, width = 6, height = 5, dpi = 300)
# 
# save legend
# legend <- ggpubr::get_legend(bar_chart)
# legend_plot <- ggpubr::as_ggplot(legend)
# ggsave("bar_chart_legend.png", legend_plot, width = 10, height = 2, dpi = 300)
```

### Fig 2 Alpha diversity (shannon)

```
tse_subset_by_03kk_ASV <- mia::addAlpha(tse_subset_by_03kk_ASV, 
                             assay_name = "counts", 
                             index = "observed", 
                             name="observed_richness")


tse_subset_by_03kk_ASV <- mia::addAlpha(tse_subset_by_03kk_ASV, 
                              assay.type = "counts", # Calculate diversity from "counts" assay 
                              index = "shannon",
                              name = "shannon_diversity") 

index <- "shannon_diversity"
group_var <- "dmmclust"


# Calculate p values
pvals <- pairwise.wilcox.test(
    tse_subset_by_03kk_ASV[[index]], tse_subset_by_03kk_ASV[[group_var]], p.adjust.method = "BH")
# Put them to data.frame format
pvals <- pvals[["p.value"]] |>
    as.data.frame()
varname <- "group1"
pvals[[varname]] <- rownames(pvals)
# To long format
pvals <- reshape(
    pvals,
    direction = "long",
    varying = colnames(pvals)[ !colnames(pvals) %in% varname ],
    times = colnames(pvals)[ !colnames(pvals) %in% varname ],
    v.names = "p",
    timevar = "group2",
    idvar = "group1"
    ) |>
    na.omit()
# Add y-axis position
pvals[["y.position"]] <- apply(pvals, 1, function(x){
    temp1 <- tse_subset_by_03kk_ASV[[index]][ tse_subset_by_03kk_ASV[[group_var]] == x[["group1"]] ]
    temp2 <- tse_subset_by_03kk_ASV[[index]][ tse_subset_by_03kk_ASV[[group_var]] == x[["group2"]] ]
    temp <- max( c(temp1, temp2) )
    return(temp)
})
pvals[["y.position"]] <- max(pvals[["y.position"]]) +
    order(pvals[["y.position"]]) * 0.2
```

```
# extract metadata to a data.frame
df <- as.data.frame(colData(tse_subset_by_03kk_ASV))

# color scheme
annotation_colors <- list(
  Cluster = c("1" = "#003C30", "2" = "#01665E", "3" = "#35978F")
)

pvals$signif <- ifelse(pvals$p < 0.001, "***",
                       ifelse(pvals$p < 0.01, "**",
                              ifelse(pvals$p < 0.05, "*", "ns")))

pvals <- subset(pvals, signif != "ns")

alpha_div_dmm_3mo <- ggplot(df, aes(x = .data[[group_var]], y = .data[[index]], colour = .data[[group_var]])) +
    geom_boxplot(outlier.shape = NA, alpha = 0.4) +
    geom_jitter(width = 0.2, size = 1, alpha = 0.7) +
    scale_color_manual(values = annotation_colors$Cluster,
                       name   = "Cluster") +  # custom colors and rename legend
    labs(
        x = "",
        y = "Shannon diversity"
    ) +
    theme_pubr(base_size = 20,
               legend = "bottom") +
    stat_pvalue_manual(pvals, label = "signif")

alpha_div_dmm_3mo
```

```
#ggsave("alpha_div_dmm_3mo_new.png", width = 8, height = 7, dpi = 300)
```

### Fig 2 Heatmap

Pairwise heat map of Bray-Curtis dissimilarities variation between
and within clusters

```
tse_subset_by_03kk_ASV <- transformAssay(
     tse_subset_by_03kk_ASV, assay.type = "counts", method = "relabundance")


data3kk <- dplyr::select(meta3kk, 
                       c("nro", "LNnT_nmol", "BMI_aiti", "AIEMMATSYNNYTYKSET_cat", "SYNNYTYSTAPA_2luok", "dmmclust")) #remove na values

data3kk1 <- na.omit(data3kk)

tse_subset_PERMANOVA<- tse_subset_by_03kk_ASV[ , tse_subset_by_03kk_ASV$nro %in% c(data3kk1$nro)]

meta3kk_no <- as.data.frame(colData(tse_subset_PERMANOVA))
```

Calculate Bray Curtis distance matrix

```
tse_subset_PERMANOVA <- transformAssay(tse_subset_PERMANOVA, assay.type = "counts", method = "relabundance")

bray_curtis_dist <-vegdist(t(assays(tse_subset_PERMANOVA)$relabundance), method="bray")

# Convert to a matrix
bray_curtis_matrix <- as.matrix(bray_curtis_dist)
```

```
# Assign clusters to samples
clusters <- colData(tse_subset_PERMANOVA)$dmmclust  

# Get order of samples based on cluster assignment
sorted_indices <- order(clusters)

# Reorder distance matrix and metadata
bray_curtis_matrix_sorted <- bray_curtis_matrix[sorted_indices, sorted_indices]

sorted_clusters <- clusters[sorted_indices]  # Sort clusters for annotation

# Create a color annotation for clusters
annotation_df <- data.frame(Cluster = as.factor(sorted_clusters))
rownames(annotation_df) <- rownames(bray_curtis_matrix_sorted)
```

```
# Define the color scheme for the annotations
annotation_colors <- list(
  Cluster = c("1" = "#003C30", "2" = "#01665E", "3" = "#35978F")
)

DMM_heatmap1 <- Heatmap(
  bray_curtis_matrix_sorted,
  col = colorRampPalette(c("#003C30", "white"))(50),
  
  # Top annotation WITH legend
  top_annotation = HeatmapAnnotation(
    df = annotation_df,
    col = annotation_colors,
    show_legend = FALSE
  ),
  
  # Left annotation NO legend
  left_annotation = rowAnnotation(
    df = annotation_df,
    col = annotation_colors,
    show_legend = TRUE,        # show annotation legend
    annotation_legend_param = list(
      Cluster = list(
        title = "Cluster",
        title_gp = gpar(fontsize = 12, fontface = "bold", fontfamily = "sans"),
        labels_gp = gpar(fontsize = 12, fontfamily = "sans"),
        ncol = 1,                # stack vertically
        grid_height = unit(6, "mm")))
  ),
  
  show_row_names = FALSE,
  show_column_names = FALSE,
  cluster_rows = FALSE,
  cluster_columns = FALSE,
  
  heatmap_legend_param = list(
    title = "Bray–Curtis \nDissimilarity",
    title_gp = gpar(fontsize = 12, fontface = "bold", fontfamily = "sans"),
    labels_gp = gpar(fontsize = 12, fontfamily = "sans"),
    legend_height = unit(6, "cm"),
    # background and border to mimic theme_pubr() (clean white, no frame)
    background = "white",
    border = NA)
)

DMM_heatmap1
```

```
# Save using grid graphics (optional for some plots)

#ggsave("DMM_heatmap1_new.png", plot = grid.grabExpr(draw(DMM_heatmap1)), width = 8, height = 6, dpi = 300)
```

Compute Mean Dissimilarities Within and Between Clusters

```
# Create an empty matrix to store mean dissimilarities
cluster_ids <- unique(clusters)
mean_dissimilarity_matrix <- matrix(
  NA, 
  nrow = length(cluster_ids), 
  ncol = length(cluster_ids),
  dimnames = list(cluster_ids, cluster_ids)
)

# Loop through each cluster pair
for (i in cluster_ids) {
  for (j in cluster_ids) {
    # Get indices of samples in each cluster
    cluster_i <- which(clusters == i)
    cluster_j <- which(clusters == j)
    
    # Extract the relevant part of the Bray-Curtis matrix
    submat <- bray_curtis_matrix[cluster_i, cluster_j]
    
    if (i == j) {
      # Within-cluster: exclude diagonal (self-pairs)
      dissimilarities <- submat[lower.tri(submat)]
    } else {
      # Between clusters: use all values
      dissimilarities <- submat
    }
    
    # Compute the mean dissimilarity
    mean_dissimilarity_matrix[i, j] <- mean(dissimilarities, na.rm = TRUE)
  }
}

# Order rows/columns numerically if cluster IDs are numbers stored as characters
mean_dissimilarity_matrix <- mean_dissimilarity_matrix[
  order(as.numeric(rownames(mean_dissimilarity_matrix))),
  order(as.numeric(colnames(mean_dissimilarity_matrix)))
]

# Print the mean dissimilarity matrix
print(mean_dissimilarity_matrix)
```

```
##           1         2         3
## 1 0.8340710 0.8917865 0.9692262
## 2 0.8917865 0.8454192 0.9786764
## 3 0.9692262 0.9786764 0.9264002
```

PERMANOVA

How much variation does the clusters explain in the microbiota.What
is the homogeneity of the group dispersions, or whether the spread of
samples within different groups is similar.

```
method <- "relabundance"
diss_method <- "bray"

set.seed(123)

pairwise.adonis2(t(assay(tse_subset_PERMANOVA, method)) ~ dmmclust + BMI_aiti + AIEMMATSYNNYTYKSET_cat +  SYNNYTYSTAPA_2luok, 
                 data = colData(tse_subset_PERMANOVA), 
                 method = diss_method, 
                 permutations = 999)
```

```
## $parent_call
## [1] "t(assay(tse_subset_PERMANOVA, method)) ~ dmmclust + BMI_aiti + AIEMMATSYNNYTYKSET_cat + SYNNYTYSTAPA_2luok , strata = Null , permutations 999"
## 
## $`2_vs_3`
##           Df SumOfSqs      R2      F Pr(>F)    
## Model      4   11.903 0.09665 7.4094  0.001 ***
## Residual 277  111.251 0.90335                  
## Total    281  123.154 1.00000                  
## ---
## Signif. codes:  0 '***' 0.001 '**' 0.01 '*' 0.05 '.' 0.1 ' ' 1
## 
## $`2_vs_1`
##           Df SumOfSqs      R2      F Pr(>F)    
## Model      4   10.497 0.06767 7.2218  0.001 ***
## Residual 398  144.622 0.93233                  
## Total    402  155.119 1.00000                  
## ---
## Signif. codes:  0 '***' 0.001 '**' 0.01 '*' 0.05 '.' 0.1 ' ' 1
## 
## $`3_vs_1`
##           Df SumOfSqs      R2      F Pr(>F)    
## Model      4   13.524 0.09371 8.8402  0.001 ***
## Residual 342  130.799 0.90629                  
## Total    346  144.323 1.00000                  
## ---
## Signif. codes:  0 '***' 0.001 '**' 0.01 '*' 0.05 '.' 0.1 ' ' 1
## 
## attr(,"class")
## [1] "pwadstrata" "list"
```

```
bd <- betadisper(
  vegdist(t(assay(tse_subset_PERMANOVA, method)), method = diss_method), 
  colData(tse_subset_PERMANOVA)$dmmclust)

permutest(bd)
```

```
## 
## Permutation test for homogeneity of multivariate dispersions
## Permutation: free
## Number of permutations: 999
## 
## Response: Distances
##            Df  Sum Sq  Mean Sq      F N.Perm Pr(>F)    
## Groups      2 0.33583 0.167914 28.249    999  0.001 ***
## Residuals 513 3.04935 0.005944                         
## ---
## Signif. codes:  0 '***' 0.001 '**' 0.01 '*' 0.05 '.' 0.1 ' ' 1
```

```
# Visualize the betadisper result
plot(bd)
```

```
# Add boxplot to compare dispersion visually
boxplot(bd)
```

Pairwise adonis

```
set.seed(123)

pairwise.adonis2(t(assay(tse_subset_PERMANOVA, method)) ~ dmmclust + BMI_aiti + AIEMMATSYNNYTYKSET_cat +  SYNNYTYSTAPA_2luok, 
                 data = colData(tse_subset_PERMANOVA), 
                 method = diss_method, 
                 permutations = 999)
```

```
## $parent_call
## [1] "t(assay(tse_subset_PERMANOVA, method)) ~ dmmclust + BMI_aiti + AIEMMATSYNNYTYKSET_cat + SYNNYTYSTAPA_2luok , strata = Null , permutations 999"
## 
## $`2_vs_3`
##           Df SumOfSqs      R2      F Pr(>F)    
## Model      4   11.903 0.09665 7.4094  0.001 ***
## Residual 277  111.251 0.90335                  
## Total    281  123.154 1.00000                  
## ---
## Signif. codes:  0 '***' 0.001 '**' 0.01 '*' 0.05 '.' 0.1 ' ' 1
## 
## $`2_vs_1`
##           Df SumOfSqs      R2      F Pr(>F)    
## Model      4   10.497 0.06767 7.2218  0.001 ***
## Residual 398  144.622 0.93233                  
## Total    402  155.119 1.00000                  
## ---
## Signif. codes:  0 '***' 0.001 '**' 0.01 '*' 0.05 '.' 0.1 ' ' 1
## 
## $`3_vs_1`
##           Df SumOfSqs      R2      F Pr(>F)    
## Model      4   13.524 0.09371 8.8402  0.001 ***
## Residual 342  130.799 0.90629                  
## Total    346  144.323 1.00000                  
## ---
## Signif. codes:  0 '***' 0.001 '**' 0.01 '*' 0.05 '.' 0.1 ' ' 1
## 
## attr(,"class")
## [1] "pwadstrata" "list"
```

# Fecal community types 13 month

## DMM clust genus level

```
tse_subset_by_13kk_Genus <- agglomerateByRank(tse_subset_by_13kk_ASV, rank = "Genus", agglomerateTree = TRUE)


set.seed(123)
tse_subset_by_13kk_Genus <- addCluster(
    tse_subset_by_13kk_Genus,
    assay.type = "counts",
    name = "DMM",
    DmmParam(k = 1:7, type = "laplace"),
    MARGIN = "samples",
    full = TRUE,
    clust.col = "dmmclust")
```

```
p <- plotDMNFit(tse_subset_by_13kk_Genus, type = "laplace", name = "DMM")

a2 <- p + theme_minimal(base_size = 11)
a2
```

```
#ggsave("13kk_DMM1.tiff", width = 7, height = 5, dpi = 300)
```

```
# Get the estimates on how much each phyla contributes on each cluster
best_model <- metadata(tse_subset_by_13kk_Genus)$DMM$dmm[4]
drivers <- as.data.frame(best_model[[1]]@fit$Estimate)

b2 <- plotLoadings(as.matrix(drivers), ncomponents = 4)
b2
```

PCoA with bray curtis

```
tse_subset_by_13kk_Genus <- transformAssay(tse_subset_by_13kk_Genus, assay.type = "counts", method = "relabundance")


# Add the MDS dimensions for plotting
tse_subset_by_13kk_Genus <- runMDS(
    tse_subset_by_13kk_Genus,
    assay.type = "relabundance",
    FUN = vegan::vegdist,
    method = "bray",
    name = "PCoA_bray")

# Calculate explained variance
e <- attr(reducedDim(tse_subset_by_13kk_Genus, "PCoA_bray"), "eig")
rel_eig <- e / sum(e[e > 0])


# principal coordinate analysis
df <- reducedDim(tse_subset_by_13kk_Genus, "PCoA_bray")

# Create a data frame from principal coordinates
bray_pcoa_df <- data.frame(pcoa1 = df[, 1], pcoa2 = df[, 2])

# Create a data frame that contains principal coordinates and DMM information
bray_pcoa_df <- cbind(bray_pcoa_df,
                               dmm_component = colData(tse_subset_by_13kk_Genus)$dmmclust,
                               birth_mde = colData(tse_subset_by_13kk_Genus)$SYNNYTYSTAPA_2luok,
                               breastfeeding_category = colData(tse_subset_by_13kk_Genus)$breastfeeding_13kk_category,
                      Secretor = colData(tse_subset_by_13kk_Genus)$Secretor)


bray_pcoa_df <- bray_pcoa_df %>%
  mutate(Secretor = ifelse(Secretor == "0", "Non-secretor","Secretor"))
```

```
bray_dmm_plot <- ggplot(data = bray_pcoa_df,
                        aes(x = pcoa1, y = pcoa2, color = dmm_component, shape = Secretor)) +
    geom_point(size = 3, alpha = 0.8) +  # Increased size and added transparency
    labs(x = paste("PCoA 1 (", round(100 * rel_eig[[1]], 1), "%)", sep = ""),
         y = paste("PCoA 2 (", round(100 * rel_eig[[2]], 1), "%)", sep = ""),
         color = "DMMclust", title = "") + 
    stat_ellipse(aes(group = Secretor), show.legend = TRUE) +  # Updated group aesthetic
    scale_color_manual(name = "DMM Component",
                       breaks = c("1", "2", "3", "4"),
                       values = c("#4D9221", "#35978F" , "#DE77AE" , "#BF812D")) +
    theme_minimal() +
    theme(plot.title = element_text(size = 12, hjust = 0.5))

bray_dmm_plot
```

```
fig1 <- cowplot::plot_grid(a2, b2, bray_dmm_plot,nrow = 3, labels = c('A', "B", "C"), align = "hv")

#ggsave("13kk_DMM_new.tiff", width = 15, height = 25, dpi = 300)

#Add dmm clusters to the df
colData(tse_subset_by_13kk_ASV)$dmmclust <- as.data.frame(colData(tse_subset_by_13kk_Genus))$dmmclust

#saveRDS(tse_subset_by_13kk_ASV, "C:/HMO_1/tse_subset_by_13kk_ASV_HMO")
```

### Fig 2 bar plot 13 month

```
metadata <- as.data.frame(colData(tse_subset_by_13kk_ASV)) %>%
  dplyr::select(sample_ID, dmmclust)

taxonomy <- as.data.frame(rowData(tse_subset_by_13kk_ASV)) %>%
  rownames_to_column(var = "ASV") %>%
  select(ASV, Genus)


otu_counts <- as.data.frame(assay(tse_subset_by_13kk_ASV, "counts"))%>% 
  rownames_to_column(var = "ASV") %>%
  tidyr:::pivot_longer(cols = 2:523, names_to = "sample_ID", values_to = "count")
```

```
otu_red_abund <- dplyr::inner_join(metadata, otu_counts, by= "sample_ID") %>%
  dplyr::inner_join(., taxonomy, by= "ASV") %>%
  dplyr::group_by(sample_ID) %>%
  mutate(rel_abund = count/ sum(count))


taxon_rel_abund <- otu_red_abund %>%
  group_by(sample_ID, dmmclust, Genus) %>%
  summarise(rel_abund = sum(rel_abund), .groups = "drop") %>%
  dplyr::group_by(dmmclust, Genus) %>%
  summarise(mean_rel_abund = 100*mean(rel_abund, na.rm = TRUE), .groups = "drop")


taxon_pool <- taxon_rel_abund %>%
  group_by(Genus)%>%
  summarize(pool = max(mean_rel_abund) < 4, .groups = "drop")


# Define the desired order of taxa
taxon_order <- c("Bifidobacterium","Bacteroides", "Agathobacter", "Anaerostipes",  
                 "Faecalibacterium", "Blautia","Roseburia", 
                 "[Ruminococcus] gnavus group", "Other")


bar_chart2 <- dplyr::inner_join(taxon_rel_abund, taxon_pool, by = "Genus") %>%
  mutate(Genus = if_else(pool, "Other", Genus),
         Genus = factor(Genus, levels = taxon_order)) %>%
  group_by(dmmclust, Genus) %>%
  summarize(mean_rel_abund = sum(mean_rel_abund), .groups = 'drop') %>%
  ggplot(aes(x = dmmclust, y = mean_rel_abund, fill = Genus)) +
  labs(x = "", y = "Mean relative abundance (100%)") +
  geom_col() +
  scale_fill_manual(values = c("#8C510A", "#BF812D","#543005" ,"#DFC27D" ,"#F6E8C3" , "#01665E","#80CDC1", "#35978F","#003C30"))+
  theme_pubr(base_size = 15)

bar_chart2
```

```
# # save main plot
# bar_chart_no_legend2 <- bar_chart2 + theme(legend.position = "none")
# ggsave("bar_chart_13mo_no_legend.png", bar_chart_no_legend2, width = 6, height = 5, dpi = 300)
# 
# #save legend
# legend <- ggpubr::get_legend(bar_chart2)
# legend_plot <- ggpubr::as_ggplot(legend)
# ggsave("bar_chart_legend_13.png", legend_plot, width = 10, height = 2, dpi = 300)
```

### Fig 2 Alpha diversity (shannon) 13 month

```
tse_subset_by_13kk_ASV <- mia::addAlpha(tse_subset_by_13kk_ASV, 
                             assay_name = "counts", 
                             index = "observed", 
                             name="observed_richness")


tse_subset_by_13kk_ASV <- mia::addAlpha(tse_subset_by_13kk_ASV, 
                              assay.type = "counts", # Calculate diversity from "counts" assay 
                              index = "shannon",
                              name = "shannon_diversity")


index <- "shannon_diversity"
group_var <- "dmmclust"

# Calculate p values
pvals <- pairwise.wilcox.test(
    tse_subset_by_13kk_ASV[[index]], tse_subset_by_13kk_ASV[[group_var]], p.adjust.method = "BH")
# Put them to data.frame format
pvals <- pvals[["p.value"]] |>
    as.data.frame()
varname <- "group1"
pvals[[varname]] <- rownames(pvals)
# To long format
pvals <- reshape(
    pvals,
    direction = "long",
    varying = colnames(pvals)[ !colnames(pvals) %in% varname ],
    times = colnames(pvals)[ !colnames(pvals) %in% varname ],
    v.names = "p",
    timevar = "group2",
    idvar = "group1"
    ) |>
    na.omit()
# Add y-axis position
pvals[["y.position"]] <- apply(pvals, 1, function(x){
    temp1 <- tse_subset_by_13kk_ASV[[index]][ tse_subset_by_13kk_ASV[[group_var]] == x[["group1"]] ]
    temp2 <- tse_subset_by_13kk_ASV[[index]][ tse_subset_by_13kk_ASV[[group_var]] == x[["group2"]] ]
    temp <- max( c(temp1, temp2) )
    return(temp)
})
pvals[["y.position"]] <- max(pvals[["y.position"]]) +
    order(pvals[["y.position"]]) * 0.2
```

```
# extract metadata to a data.frame
df <- as.data.frame(colData(tse_subset_by_13kk_ASV))

annotation_colors <- list(
  Cluster = c("1" = "#003C30", "2" = "#01665E", "3" = "#35978F", "4" = "#80CDC1")
)

pvals$signif <- ifelse(pvals$p < 0.001, "***",
                       ifelse(pvals$p < 0.01, "**",
                              ifelse(pvals$p < 0.05, "*", "ns")))

pvals <- subset(pvals, signif != "ns")

alpha_div_dmm_13mo <- ggplot(df, aes(x = .data[[group_var]], y = .data[[index]], colour = .data[[group_var]])) +
    geom_boxplot(outlier.shape = NA, alpha = 0.4) +
    geom_jitter(width = 0.2, size = 1, alpha = 0.7) +
    scale_color_manual(values = annotation_colors$Cluster,
                       name   = "Cluster") +  # custom colors and rename legend
    labs(
        x = "",
        y = "Shannon diversity"
    ) +
    theme_pubr(base_size = 20,
               legend = "bottom") +
    stat_pvalue_manual(pvals, label = "signif")

alpha_div_dmm_13mo
```

```
#ggsave("alpha_div_dmm_13mo_new.png", width = 8, height = 7, dpi = 300)
```

### Fig 2 Heatmap 13 month

```
#Late infancy

tse_subset_by_13kk_ASV <- transformAssay(
     tse_subset_by_13kk_ASV, assay.type = "counts", method = "relabundance")


data13kk <- dplyr::select(meta13kk, 
                       c("nro", "LNnT_nmol", "BMI_aiti", "AIEMMATSYNNYTYKSET_cat", "SYNNYTYSTAPA_2luok", "dmmclust"))

data13kk1 <- na.omit(data13kk)

tse_subset_PERMANOVA_13<- tse_subset_by_13kk_ASV[ , tse_subset_by_13kk_ASV$nro %in% c(data13kk1$nro)]
tse_subset_PERMANOVA_13 <- transformAssay(tse_subset_PERMANOVA_13, assay.type = "counts", method = "relabundance")

meta13kk_no <- as.data.frame(colData(tse_subset_PERMANOVA_13))
```

pairwise heatmap of Bray-Curtis dissimilarities

```
bray_curtis_dist_13 <-vegdist(t(assays(tse_subset_PERMANOVA_13)$relabundance), method="bray")

# Convert to a matrix
bray_curtis_matrix_13 <- as.matrix(bray_curtis_dist_13)
```

```
# Assign clusters to samples
clusters <- colData(tse_subset_PERMANOVA_13)$dmmclust   # Make sure metadata has the correct clustering information

# Get order of samples based on cluster assignment
sorted_indices <- order(clusters)

# Reorder distance matrix and metadata
bray_curtis_matrix_sorted_13 <- bray_curtis_matrix_13[sorted_indices, sorted_indices]

sorted_clusters <- clusters[sorted_indices]  # Sort clusters for annotation

#Create a color annotation for clusters
annotation_df <- data.frame(Cluster = as.factor(sorted_clusters))
rownames(annotation_df) <- rownames(bray_curtis_matrix_sorted_13)
```

```
# Define the color scheme for the annotations
annotation_colors <- list(
  Cluster = c("1" = "#003C30", "2" = "#01665E", "3" = "#35978F", "4" = "#80CDC1")
)

DMM_heatmap2 <- Heatmap(
  bray_curtis_matrix_sorted_13,
  col = colorRampPalette(c("#003C30", "white"))(50),
  
  # Top annotation WITH legend
  top_annotation = HeatmapAnnotation(
    df = annotation_df,
    col = annotation_colors,
    show_legend = FALSE
  ),
  
  # Left annotation NO legend
  left_annotation = rowAnnotation(
    df = annotation_df,
    col = annotation_colors,
    show_legend = TRUE,        # show annotation legend
    annotation_legend_param = list(
      Cluster = list(
        title = "Cluster",
        title_gp = gpar(fontsize = 12, fontface = "bold", fontfamily = "sans"),
        labels_gp = gpar(fontsize = 12, fontfamily = "sans"),
        ncol = 1,                # stack vertically
        grid_height = unit(6, "mm")))
  ),
  
  show_row_names = FALSE,
  show_column_names = FALSE,
  cluster_rows = FALSE,
  cluster_columns = FALSE,
  
  heatmap_legend_param = list(
    title = "Bray–Curtis \nDissimilarity",
    title_gp = gpar(fontsize = 12, fontface = "bold", fontfamily = "sans"),
    labels_gp = gpar(fontsize = 12, fontfamily = "sans"),
    legend_height = unit(6, "cm"),
    # background and border to mimic theme_pubr() (clean white, no frame)
    background = "white",
    border = NA)
)

DMM_heatmap2
```

```
# Save using grid graphics (optional for some plots)
#ggsave("DMM_heatmap2_new.png", plot = grid.grabExpr(draw(DMM_heatmap2)), width = 8, height = 6, dpi = 300)
```

Compute Mean Dissimilarities Within and Between Clusters

```
# Create an empty matrix to store mean dissimilarities
cluster_ids <- unique(clusters)
mean_dissimilarity_matrix_13 <- matrix(
  NA, 
  nrow = length(cluster_ids), 
  ncol = length(cluster_ids),
  dimnames = list(cluster_ids, cluster_ids)
)

# Loop through each cluster pair
for (i in cluster_ids) {
  for (j in cluster_ids) {
    # Get sample indices for the clusters
    cluster_i <- which(clusters == i)
    cluster_j <- which(clusters == j)
    
    # Subset Bray-Curtis dissimilarities
    submat <- bray_curtis_matrix_13[cluster_i, cluster_j]
    
    if (i == j) {
      # Within-cluster: exclude diagonal (self comparisons)
      dissimilarities <- submat[lower.tri(submat)]
    } else {
      # Between clusters: use all pairwise dissimilarities
      dissimilarities <- submat
    }
    
    # Compute mean dissimilarity
    mean_dissimilarity_matrix_13[i, j] <- mean(dissimilarities, na.rm = TRUE)
  }
}

# Order rows/columns numerically if cluster IDs are numbers stored as characters
mean_dissimilarity_matrix_13 <- mean_dissimilarity_matrix_13[
  order(as.numeric(rownames(mean_dissimilarity_matrix_13))),
  order(as.numeric(colnames(mean_dissimilarity_matrix_13)))
]

# Print the mean dissimilarity matrix
print(mean_dissimilarity_matrix_13)
```

```
##           1         2         3         4
## 1 0.7597186 0.7980655 0.8519476 0.7717351
## 2 0.7980655 0.7932989 0.8591057 0.8007524
## 3 0.8519476 0.8591057 0.8315360 0.8865323
## 4 0.7717351 0.8007524 0.8865323 0.7280419
```

PERMANOVA

How much variation does the clusters explain in the microbiota.What
is the homogeneity of the group dispersions, or whether the spread of
samples within different groups is similar.

```
tse_subset_PERMANOVA_13 <- transformAssay(tse_subset_PERMANOVA_13, assay.type = "counts", method = "relabundance")

method <- "relabundance"
diss_method <- "bray"

set.seed(123)

permanova <- adonis2(t(assay(tse_subset_PERMANOVA_13, method)) ~ dmmclust + BMI_aiti + AIEMMATSYNNYTYKSET_cat +  SYNNYTYSTAPA_2luok  ,
                     by = "margin",
                     data = colData(tse_subset_PERMANOVA_13),
                     method = diss_method,
                     permutations = 999)

permanova
```

```
## Permutation test for adonis under reduced model
## Marginal effects of terms
## Permutation: free
## Number of permutations: 999
## 
## adonis2(formula = t(assay(tse_subset_PERMANOVA_13, method)) ~ dmmclust + BMI_aiti + AIEMMATSYNNYTYKSET_cat + SYNNYTYSTAPA_2luok, data = colData(tse_subset_PERMANOVA_13), permutations = 999, method = diss_method, by = "margin")
##                         Df SumOfSqs      R2       F Pr(>F)    
## dmmclust                 3   12.855 0.07395 13.8972  0.001 ***
## BMI_aiti                 1    0.264 0.00152  0.8546  0.718    
## AIEMMATSYNNYTYKSET_cat   1    0.783 0.00451  2.5401  0.001 ***
## SYNNYTYSTAPA_2luok       1    0.358 0.00206  1.1615  0.201    
## Residual               514  158.489 0.91170                   
## Total                  520  173.838 1.00000                   
## ---
## Signif. codes:  0 '***' 0.001 '**' 0.01 '*' 0.05 '.' 0.1 ' ' 1
```

```
bd <- betadisper(
  vegdist(t(assay(tse_subset_PERMANOVA_13, method)), method = diss_method), 
  colData(tse_subset_PERMANOVA_13)$dmmclust)

permutest(bd)
```

```
## 
## Permutation test for homogeneity of multivariate dispersions
## Permutation: free
## Number of permutations: 999
## 
## Response: Distances
##            Df Sum Sq  Mean Sq      F N.Perm Pr(>F)    
## Groups      3 0.3177 0.105901 32.766    999  0.001 ***
## Residuals 517 1.6710 0.003232                         
## ---
## Signif. codes:  0 '***' 0.001 '**' 0.01 '*' 0.05 '.' 0.1 ' ' 1
```

```
# Visualize the betadisper result
plot(bd)
```

```
# Add boxplot to compare dispersion visually
boxplot(bd)
```

pairwise adonis 13mo

```
method <- "relabundance"
diss_method <- "bray"

set.seed(123)

pairwise.adonis2(t(assay(tse_subset_PERMANOVA_13, method)) ~ dmmclust + BMI_aiti + AIEMMATSYNNYTYKSET_cat +  SYNNYTYSTAPA_2luok, 
                 data = colData(tse_subset_PERMANOVA_13), 
                 method = diss_method, 
                 permutations = 999)
```

```
## $parent_call
## [1] "t(assay(tse_subset_PERMANOVA_13, method)) ~ dmmclust + BMI_aiti + AIEMMATSYNNYTYKSET_cat + SYNNYTYSTAPA_2luok , strata = Null , permutations 999"
## 
## $`4_vs_3`
##           Df SumOfSqs      R2      F Pr(>F)    
## Model      4     8.82 0.13079 6.8839  0.001 ***
## Residual 183    58.62 0.86921                  
## Total    187    67.44 1.00000                  
## ---
## Signif. codes:  0 '***' 0.001 '**' 0.01 '*' 0.05 '.' 0.1 ' ' 1
## 
## $`4_vs_1`
##           Df SumOfSqs      R2      F Pr(>F)    
## Model      4    3.483 0.04401 3.0495  0.001 ***
## Residual 265   75.677 0.95599                  
## Total    269   79.161 1.00000                  
## ---
## Signif. codes:  0 '***' 0.001 '**' 0.01 '*' 0.05 '.' 0.1 ' ' 1
## 
## $`4_vs_2`
##           Df SumOfSqs      R2      F Pr(>F)    
## Model      4    4.275 0.06786 3.5671  0.001 ***
## Residual 196   58.726 0.93214                  
## Total    200   63.001 1.00000                  
## ---
## Signif. codes:  0 '***' 0.001 '**' 0.01 '*' 0.05 '.' 0.1 ' ' 1
## 
## $`3_vs_1`
##           Df SumOfSqs      R2      F Pr(>F)    
## Model      4    8.326 0.07776 6.6401  0.001 ***
## Residual 315   98.747 0.92224                  
## Total    319  107.073 1.00000                  
## ---
## Signif. codes:  0 '***' 0.001 '**' 0.01 '*' 0.05 '.' 0.1 ' ' 1
## 
## $`3_vs_2`
##           Df SumOfSqs      R2      F Pr(>F)    
## Model      4    6.369 0.07215 4.7823  0.001 ***
## Residual 246   81.907 0.92785                  
## Total    250   88.276 1.00000                  
## ---
## Signif. codes:  0 '***' 0.001 '**' 0.01 '*' 0.05 '.' 0.1 ' ' 1
## 
## $`1_vs_2`
##           Df SumOfSqs      R2      F Pr(>F)    
## Model      4    4.382 0.04246 3.6362  0.001 ***
## Residual 328   98.813 0.95754                  
## Total    332  103.195 1.00000                  
## ---
## Signif. codes:  0 '***' 0.001 '**' 0.01 '*' 0.05 '.' 0.1 ' ' 1
## 
## attr(,"class")
## [1] "pwadstrata" "list"
```

### Table characteristics between DMM clusters

Supplementary 1

Descriptive characteristics of the subpopulation of the STEPS Study
mothers and their infants in 3 month and 13-month timepoint, in relation
to dmm clusters. Sample sizes and percentages are given for categorical
variables and means with standard deviations for continuous variables.
Asterisks show significant associations between maternal secretor status
and child or maternal characteristic (p < 0.05).

3 month

```
meta3kk_df <- dplyr::select(meta3kk, 
                       c("SP",
                         "SYNTYMAPAINO_kg",
                         "KESTOVKPV_num", 
                         "SYNNYTYSTAPA_2luok", 
                         "BMI_aiti", 
                         "Aidinika",
                         "aidinab", 
                         "Antib", 
                         "ika_3kk",
                         "season_3kk", 
                         "dmmclust",
                         "lib_size", 
                         "AIEMMATSYNNYTYKSET_cat",
                         "Secretor",
                         "breastfeeding_3kk_category"
                         ))

# Define continuous and categorical variables
continuous_vars <- c("SYNTYMAPAINO_kg", "KESTOVKPV_num", "Aidinika", "ika_3kk", "lib_size", "BMI_aiti")
categorical_vars <- c("SP","SYNNYTYSTAPA_2luok", "aidinab", "Antib",  "season_3kk","AIEMMATSYNNYTYKSET_cat","Secretor","breastfeeding_3kk_category")

# Combine variables into a single list
vars <- c(continuous_vars, categorical_vars)


# Create table and specify tests
table_one1 <- CreateTableOne(
  vars = vars,
  strata = "dmmclust",  # Groups to compare
  data = meta3kk_df,
  factorVars = categorical_vars,  # Categorical variables
  smd = TRUE,  # Add standardized mean differences
  addOverall = TRUE  # Add overall summary
)

# Step 1: Print with all levels (this works for console and data capture)
table_one_printed <- print(table_one1,
                           quote = FALSE,
                           noSpaces = TRUE,
                           printToggle = FALSE,
                           showAllLevels = TRUE)

# Step 2: Convert properly: row names → "Variable" column
table_one_df <- cbind(Variable = rownames(table_one_printed),
                      as.data.frame.matrix(table_one_printed))  # better than data.frame()


#Write to Excel
#write_xlsx(table_one_df, "table_one_dmm3kk.xlsx")
```

```
#Test statistical significance: 
# # Grouping variable
# group_var <- "dmmclust"
# 
# # Define continuous and categorical variables
# continuous_vars <- c("SYNTYMAPAINO_kg", "KESTOVKPV_num", "BMI_aiti", 
#                      "Aidinika", "ika_3kk", "lib_size")
# 
# categorical_vars <- c("SP", "SYNNYTYSTAPA_2luok", "aidinab", "Antib",  
#                       "season_3kk", "AIEMMATSYNNYTYKSET_cat", 
#                       "Secretor", "breastfeeding_3kk_category")
# 
# 
# for (var in continuous_vars) {
#   cat("\nKruskal-Wallis test for:", var, "\n")
#   print(kruskal.test(as.formula(paste(var, "~", group_var)), data = meta3kk_df))
# }
# 
# for (var in categorical_vars) {
#   # Remove NAs to avoid errors in table()
#   df_clean <- meta3kk_df[!is.na(meta3kk_df[[var]]) & !is.na(meta3kk_df[[group_var]]), ]
#   
#   cat("\nChi-squared test for:", var, "\n")
#   tbl <- table(df_clean[[var]], df_clean[[group_var]])
#   print(chisq.test(tbl))
# }
```

13 month

```
meta13kk_df <- dplyr::select(meta13kk, 
                       c("SP",
                         "SYNTYMAPAINO_kg",
                         "KESTOVKPV_num", 
                         "SYNNYTYSTAPA_2luok", 
                         "BMI_aiti", 
                         "Aidinika",
                         "aidinab", 
                         "Antib", 
                         "ika_13kk",
                         "season_13kk", 
                         "dmmclust",
                         "lib_size", 
                         "AIEMMATSYNNYTYKSET_cat",
                         "Secretor",
                         "breastfeeding_13kk_category"
                         ))


# Define continuous and categorical variables
continuous_vars <- c("SYNTYMAPAINO_kg", "KESTOVKPV_num", "Aidinika", "ika_13kk", "lib_size", "BMI_aiti")
categorical_vars <- c("SP","SYNNYTYSTAPA_2luok", "aidinab", "Antib",  "season_13kk","AIEMMATSYNNYTYKSET_cat", "Secretor", "breastfeeding_13kk_category")

# Combine variables into a single list
vars <- c(continuous_vars, categorical_vars)

# Create table and specify tests
table_one2 <- CreateTableOne(
  vars = vars,
  strata = "dmmclust",  # Groups to compare
  data = meta13kk_df,
  factorVars = categorical_vars,  # Categorical variables
  smd = TRUE,  # Add standardized mean differences
  addOverall = TRUE  # Add overall summary
)

# Step 1: Print with all levels (this works for console and data capture)
table_one_printed2 <- print(table_one2,
                           quote = FALSE,
                           noSpaces = TRUE,
                           printToggle = FALSE,
                           showAllLevels = TRUE)

# Step 2: Convert properly: row names → "Variable" column
table_one_df2 <- cbind(Variable = rownames(table_one_printed2),
                      as.data.frame.matrix(table_one_printed2))  # better than data.frame()
# Write to Excel
#write_xlsx(table_one_df2, "table_one_dmm2.xlsx")
```

```
#Test statistical significance: 
# for (var in continuous_vars) {
#   cat("\nKruskal-Wallis test for:", var, "\n")
#   print(kruskal.test(as.formula(paste(var, "~", group_var)), data = meta13kk_df))
# }
# 
# for (var in categorical_vars) {
#   # Remove NAs to avoid errors in table()
#   df_clean <- meta13kk_df[!is.na(meta13kk_df[[var]]) & !is.na(meta13kk_df[[group_var]]), ]
#   
#   cat("\nChi-squared test for:", var, "\n")
#   tbl <- table(df_clean[[var]], df_clean[[group_var]])
#   print(chisq.test(tbl))
# }
```

# Multinomial logistic regression model

significance betwee HMO concenetrations and FCTs

### HMO vs DMM cluster 3 month

Secretor status

```
# --- Model 1: ref = 1, gives 2 vs 1 and 3 vs 1
meta3kk$dmmclust <- relevel(factor(meta3kk$dmmclust), ref = "1")

mod_ref1 <- multinom(dmmclust ~ Secretor + SYNNYTYSTAPA_2luok + BMI_aiti + AIEMMATSYNNYTYKSET_cat, data = meta3kk)

tidy_ref1 <- broom::tidy(mod_ref1, conf.int = TRUE, exponentiate = TRUE) %>%
  mutate(comparison = paste0(y.level, " vs 1"))

# --- Model 2: ref = 2, gives 3 vs 2
meta3kk$dmmclust <- relevel(factor(meta3kk$dmmclust), ref = "2")

mod_ref2 <- multinom(dmmclust ~ Secretor + SYNNYTYSTAPA_2luok + BMI_aiti + AIEMMATSYNNYTYKSET_cat, data = meta3kk)

tidy_ref2 <- broom::tidy(mod_ref2, conf.int = TRUE, exponentiate = TRUE) %>%
  filter(y.level == "3") %>%  # Only want 3 vs 2 here
  mutate(comparison = "3 vs 2")

# --- Combine both sets of results
all_results <- bind_rows(tidy_ref1, tidy_ref2)

# Format OR (95% CI)
formatted_results <- all_results %>%
  mutate(OR_CI = paste0(round(estimate, 4), " (", 
                        round(conf.low, 4), "–", 
                        round(conf.high, 4), ")")) %>%
  select(comparison, term, OR_CI)

# Pivot wider: comparisons as columns
pretty_table <- formatted_results %>%
  pivot_wider(names_from = comparison, values_from = OR_CI)
```

z-score transformed HMO values

Odds ratios (OR) and 95% confidence intervals (CI) are shown for each
HMO. Significance stars reflect FDR-adjusted p-values: p < 0.05 (), p
< 0.01 (), and p < 0.001 ().

Individual HMOs and HMO summary measures

```
variables <- c(
  "X_2FL_nmol_scale", "X_3FL_nmol_scale", "LNnT_nmol_scale", "X_3SL_nmol_scale", "DFLac_nmol_scale", 
  "X_6SL_nmol_scale", "LNT_nmol_scale", "LNFP_I_nmol_scale", "LNFP_II_nmol_scale", "LNFP_III_nmol_scale", 
  "LSTb_nmol_scale", "LSTc_nmol_scale", "DFLNT_nmol_scale", "LNH_nmol_scale", "DSLNT_nmol_scale", 
  "FLNH_nmol_scale", "DFLNH_nmol_scale", "FDSLNH_nmol_scale", "DSLNH_nmol_scale", "SUM_nmol_scale", 
  "Sia_scale", "Fuc_scale", "Diversity"
)

unique_comparisons <- c("2 vs 1", "3 vs 1", "3 vs 2")

# Significance star helper
get_signif <- function(p) {
  case_when(
    p < 0.001 ~ "***",
    p < 0.01 ~ "**",
    p < 0.05 ~ "*",
    TRUE ~ ""
  )
}

get_or_for_hmo <- function(var, data) {
  refs <- c("1", "2", "3")
  all_comps <- list()
  
  for (ref in refs) {
    data$dmmclust <- relevel(factor(data$dmmclust), ref = ref)
    
    formula <- as.formula(paste("dmmclust ~", var, "+ SYNNYTYSTAPA_2luok + BMI_aiti + AIEMMATSYNNYTYKSET_cat "))
    mod <- multinom(formula, data = data, trace = FALSE)
    
    tidy_mod <- tidy(mod, exponentiate = TRUE, conf.int = TRUE)
    
    tidy_hmo <- tidy_mod %>%
      filter(term == var) %>%
      mutate(comparison = paste0(y.level, " vs ", ref))
    
    all_comps[[ref]] <- tidy_hmo
  }
  
  full_results <- bind_rows(all_comps) %>%
    filter(comparison %in% unique_comparisons) %>%
    mutate(
      HMO = var,
      p.value.adj = p.adjust(p.value, method = "BH"),
      signif_raw = get_signif(p.value),
      signif_adj = get_signif(p.value.adj),
      OR_CI_raw = paste0(
        round(estimate, 2), " (", round(conf.low, 2), "–", round(conf.high, 2), ") ", signif_raw
      ),
      OR_CI_adj = paste0(
        round(estimate, 2), " (", round(conf.low, 2), "–", round(conf.high, 2), ") ", signif_adj
      )
    ) %>%
    select(HMO, comparison, estimate, conf.low, conf.high, p.value, p.value.adj, signif_raw, signif_adj, OR_CI_raw, OR_CI_adj)
  
  return(full_results)
}

# Run for all HMOs
all_hmo_results <- map_dfr(variables, get_or_for_hmo, data = meta3kk)

# Clean HMO variable names: remove "X_" prefix and "_nmol_scale" or "_scale" suffix
all_hmo_results <- all_hmo_results %>%
  mutate(HMO = gsub("^X_", "", HMO),                # remove leading X_
         HMO = gsub("(_nmol_scale|_scale)$", "", HMO))  # remove trailing _nmol_scale or _scale


# Wide tables
pretty_wide_z_raw <- all_hmo_results %>%
  select(HMO, comparison, OR_CI_raw) %>%
  pivot_wider(names_from = comparison, values_from = OR_CI_raw)

pretty_wide_z_adj <- all_hmo_results %>%
  select(HMO, comparison, OR_CI_adj) %>%
  pivot_wider(names_from = comparison, values_from = OR_CI_adj)

# # Print both versions
# cat("\n=== ORs with Raw p-value Stars ===\n")
# #print(pretty_wide_z_raw)
# 
cat("\n=== ORs with FDR-adjusted p-value Stars ===\n")
```

```
## 
## === ORs with FDR-adjusted p-value Stars ===
```

```
print(pretty_wide_z_adj)
```

```
## # A tibble: 23 × 4
##    HMO      `2 vs 1`            `3 vs 1`            `3 vs 2`            
##    <chr>    <chr>               <chr>               <chr>               
##  1 2FL      "1.2 (0.97–1.48) "  "0.92 (0.73–1.17) " "0.77 (0.6–0.98) "  
##  2 3FL      "1.1 (0.89–1.36) "  "0.99 (0.78–1.27) " "0.9 (0.72–1.14) "  
##  3 LNnT     "1.03 (0.84–1.27) " "1.05 (0.83–1.33) " "1.02 (0.8–1.3) "   
##  4 3SL      "0.99 (0.81–1.21) " "0.79 (0.6–1.04) "  "0.8 (0.61–1.04) "  
##  5 DFLac    "0.97 (0.79–1.2) "  "0.72 (0.54–0.96) " "0.74 (0.56–0.98) " 
##  6 6SL      "0.85 (0.68–1.06) " "1.09 (0.87–1.36) " "1.29 (1–1.65) "    
##  7 LNT      "1.01 (0.82–1.24) " "0.99 (0.78–1.25) " "0.98 (0.77–1.24) " 
##  8 LNFP_I   "1.13 (0.92–1.38) " "0.9 (0.71–1.15) "  "0.8 (0.63–1.02) "  
##  9 LNFP_II  "0.86 (0.69–1.06) " "1.14 (0.91–1.43) " "1.33 (1.05–1.69) " 
## 10 LNFP_III "0.53 (0.25–1.14) " "1.86 (0.86–4.02) " "3.53 (1.47–8.45) *"
## # ℹ 13 more rows
```

Secretors and non-secretors 3mo

```
subset_03kk_ASV_Secretor<- tse_subset_by_03kk_ASV[ , tse_subset_by_03kk_ASV$Secretor %in% c("1")] 

meta3kk_se <- as.data.frame(colData(subset_03kk_ASV_Secretor))

variables <- c(
  "X_2FL_nmol_scale", "X_3FL_nmol_scale", "LNnT_nmol_scale", "X_3SL_nmol_scale", "DFLac_nmol_scale", 
  "X_6SL_nmol_scale", "LNT_nmol_scale", "LNFP_I_nmol_scale", "LNFP_II_nmol_scale", "LNFP_III_nmol_scale", 
  "LSTb_nmol_scale", "LSTc_nmol_scale", "DFLNT_nmol_scale", "LNH_nmol_scale", "DSLNT_nmol_scale", 
  "FLNH_nmol_scale", "DFLNH_nmol_scale", "FDSLNH_nmol_scale", "DSLNH_nmol_scale", "SUM_nmol_scale", 
  "Sia_scale", "Fuc_scale", "Diversity"
)

unique_comparisons <- c("2 vs 1", "3 vs 1", "3 vs 2")

# Significance star helper
get_signif <- function(p) {
  case_when(
    p < 0.001 ~ "***",
    p < 0.01 ~ "**",
    p < 0.05 ~ "*",
    TRUE ~ ""
  )
}

get_or_for_hmo <- function(var, data) {
  refs <- c("1", "2", "3")
  all_comps <- list()
  
  for (ref in refs) {
    data$dmmclust <- relevel(factor(data$dmmclust), ref = ref)
    
    formula <- as.formula(paste("dmmclust ~", var, "+ SYNNYTYSTAPA_2luok + BMI_aiti + AIEMMATSYNNYTYKSET_cat"))
    mod <- multinom(formula, data = data, trace = FALSE)
    
    tidy_mod <- tidy(mod, exponentiate = TRUE, conf.int = TRUE)
    
    tidy_hmo <- tidy_mod %>%
      filter(term == var) %>%
      mutate(comparison = paste0(y.level, " vs ", ref))
    
    all_comps[[ref]] <- tidy_hmo
  }
  
  full_results <- bind_rows(all_comps) %>%
    filter(comparison %in% unique_comparisons) %>%
    mutate(
      HMO = var,
      p.value.adj = p.adjust(p.value, method = "fdr"),
      signif_raw = get_signif(p.value),
      signif_adj = get_signif(p.value.adj),
      OR_CI_raw = paste0(
        round(estimate, 4), " (", round(conf.low, 4), "–", round(conf.high, 4), ") ", signif_raw
      ),
      OR_CI_adj = paste0(
        round(estimate, 4), " (", round(conf.low, 4), "–", round(conf.high, 4), ") ", signif_adj
      )
    ) %>%
    select(HMO, comparison, estimate, conf.low, conf.high, p.value, p.value.adj, signif_raw, signif_adj, OR_CI_raw, OR_CI_adj)
  
  return(full_results)
}

# Run for all HMOs
all_hmo_results <- map_dfr(variables, get_or_for_hmo, data = meta3kk_se)

# Clean HMO variable names: remove "X_" prefix and "_nmol_scale" or "_scale" suffix
all_hmo_results <- all_hmo_results %>%
  mutate(HMO = gsub("^X_", "", HMO),                # remove leading X_
         HMO = gsub("(_nmol_scale|_scale)$", "", HMO))  # remove trailing _nmol_scale or _scale


# Wide tables
pretty_wide_z_raw2 <- all_hmo_results %>%
  select(HMO, comparison, OR_CI_raw) %>%
  pivot_wider(names_from = comparison, values_from = OR_CI_raw)

pretty_wide_z_adj2 <- all_hmo_results %>%
  select(HMO, comparison, OR_CI_adj) %>%
  pivot_wider(names_from = comparison, values_from = OR_CI_adj)

# # Print both versions
# cat("\n=== ORs with Raw p-value Stars ===\n")
# #print(pretty_wide_z_raw2)
# 
#cat("\n=== ORs with FDR-adjusted p-value Stars ===\n")
#print(pretty_wide_z_adj2)
```

```
subset_03kk_ASV_nonsecretor<- tse_subset_by_03kk_ASV[ , tse_subset_by_03kk_ASV$Secretor %in% c("0")] 

meta3kk_nse <- as.data.frame(colData(subset_03kk_ASV_nonsecretor))

variables <- c(
  "X_2FL_nmol_scale", "X_3FL_nmol_scale", "LNnT_nmol_scale", "X_3SL_nmol_scale", "DFLac_nmol_scale", 
  "X_6SL_nmol_scale", "LNT_nmol_scale", "LNFP_I_nmol_scale", "LNFP_II_nmol_scale", "LNFP_III_nmol_scale", 
  "LSTb_nmol_scale", "LSTc_nmol_scale", "DFLNT_nmol_scale", "LNH_nmol_scale", "DSLNT_nmol_scale", 
  "FLNH_nmol_scale", "DFLNH_nmol_scale", "FDSLNH_nmol_scale", "DSLNH_nmol_scale", "SUM_nmol_scale", 
  "Sia_scale", "Fuc_scale", "Diversity"
)

unique_comparisons <- c("2 vs 1", "3 vs 1", "3 vs 2")

# Significance star helper
get_signif <- function(p) {
  case_when(
    p < 0.001 ~ "***",
    p < 0.01 ~ "**",
    p < 0.05 ~ "*",
    TRUE ~ ""
  )
}

get_or_for_hmo <- function(var, data) {
  refs <- c("1", "2", "3")
  all_comps <- list()
  
  for (ref in refs) {
    data$dmmclust <- relevel(factor(data$dmmclust), ref = ref)
    
    formula <- as.formula(paste("dmmclust ~", var, "+ SYNNYTYSTAPA_2luok + BMI_aiti + AIEMMATSYNNYTYKSET_cat"))
    mod <- multinom(formula, data = data, trace = FALSE)
    
    tidy_mod <- tidy(mod, exponentiate = TRUE, conf.int = TRUE)
    
    tidy_hmo <- tidy_mod %>%
      filter(term == var) %>%
      mutate(comparison = paste0(y.level, " vs ", ref))
    
    all_comps[[ref]] <- tidy_hmo
  }
  
  full_results <- bind_rows(all_comps) %>%
    filter(comparison %in% unique_comparisons) %>%
    mutate(
      HMO = var,
      p.value.adj = p.adjust(p.value, method = "fdr"),
      signif_raw = get_signif(p.value),
      signif_adj = get_signif(p.value.adj),
      OR_CI_raw = paste0(
        round(estimate, 4), " (", round(conf.low, 4), "–", round(conf.high, 4), ") ", signif_raw
      ),
      OR_CI_adj = paste0(
        round(estimate, 4), " (", round(conf.low, 4), "–", round(conf.high, 4), ") ", signif_adj
      )
    ) %>%
    select(HMO, comparison, estimate, conf.low, conf.high, p.value, p.value.adj, signif_raw, signif_adj, OR_CI_raw, OR_CI_adj)
  
  return(full_results)
}

# Run for all HMOs
all_hmo_results <- map_dfr(variables, get_or_for_hmo, data = meta3kk_nse)

# Clean HMO variable names: remove "X_" prefix and "_nmol_scale" or "_scale" suffix
all_hmo_results <- all_hmo_results %>%
  mutate(HMO = gsub("^X_", "", HMO),                # remove leading X_
         HMO = gsub("(_nmol_scale|_scale)$", "", HMO))  # remove trailing _nmol_scale or _scale


# Wide tables
pretty_wide_z_raw3 <- all_hmo_results %>%
  select(HMO, comparison, OR_CI_raw) %>%
  pivot_wider(names_from = comparison, values_from = OR_CI_raw)

pretty_wide_z_adj3 <- all_hmo_results %>%
  select(HMO, comparison, OR_CI_adj) %>%
  pivot_wider(names_from = comparison, values_from = OR_CI_adj)
```

### Exclusivelly BF

```
tse_subset_03kk_ASV_BF_Exclus <- tse_subset_by_03kk_ASV[ , tse_subset_by_03kk_ASV$breastfeeding_3kk_category %in% c("exclusive")] 
meta3kk_ecx <- as.data.frame(colData(tse_subset_03kk_ASV_BF_Exclus))
```

Secretor status

```
# --- Model 1: ref = 1, gives 2 vs 1 and 3 vs 1
meta3kk_ecx$dmmclust <- relevel(factor(meta3kk_ecx$dmmclust), ref = "1")

mod_ref1 <- multinom(dmmclust ~ Secretor + SYNNYTYSTAPA_2luok + BMI_aiti + AIEMMATSYNNYTYKSET_cat, data = meta3kk_ecx)

tidy_ref1 <- broom::tidy(mod_ref1, conf.int = TRUE, exponentiate = TRUE) %>%
  mutate(comparison = paste0(y.level, " vs 1"))

# --- Model 2: ref = 2, gives 3 vs 2
meta3kk_ecx$dmmclust <- relevel(factor(meta3kk_ecx$dmmclust), ref = "2")

mod_ref2 <- multinom(dmmclust ~ Secretor + SYNNYTYSTAPA_2luok + BMI_aiti + AIEMMATSYNNYTYKSET_cat, data = meta3kk_ecx)

tidy_ref2 <- broom::tidy(mod_ref2, conf.int = TRUE, exponentiate = TRUE) %>%
  filter(y.level == "3") %>%  # Only want 3 vs 2 here
  mutate(comparison = "3 vs 2")

# --- Combine both sets of results
all_results <- bind_rows(tidy_ref1, tidy_ref2)

# Format OR (95% CI)
formatted_results <- all_results %>%
  mutate(OR_CI = paste0(round(estimate, 4), " (", 
                        round(conf.low, 4), "–", 
                        round(conf.high, 4), ")")) %>%
  select(comparison, term, OR_CI)

# Pivot wider: comparisons as columns
pretty_table <- formatted_results %>%
  pivot_wider(names_from = comparison, values_from = OR_CI)
```

Individual HMOs and HMO summary measures

```
variables <- c(
  "X_2FL_nmol_scale", "X_3FL_nmol_scale", "LNnT_nmol_scale", "X_3SL_nmol_scale", "DFLac_nmol_scale", 
  "X_6SL_nmol_scale", "LNT_nmol_scale", "LNFP_I_nmol_scale", "LNFP_II_nmol_scale", "LNFP_III_nmol_scale", 
  "LSTb_nmol_scale", "LSTc_nmol_scale", "DFLNT_nmol_scale", "LNH_nmol_scale", "DSLNT_nmol_scale", 
  "FLNH_nmol_scale", "DFLNH_nmol_scale", "FDSLNH_nmol_scale", "DSLNH_nmol_scale", "SUM_nmol_scale", 
  "Sia_scale", "Fuc_scale", "Diversity"
)

unique_comparisons <- c("2 vs 1", "3 vs 1", "3 vs 2")

# Significance star helper
get_signif <- function(p) {
  case_when(
    p < 0.001 ~ "***",
    p < 0.01 ~ "**",
    p < 0.05 ~ "*",
    TRUE ~ ""
  )
}

get_or_for_hmo <- function(var, data) {
  refs <- c("1", "2", "3")
  all_comps <- list()
  
  for (ref in refs) {
    data$dmmclust <- relevel(factor(data$dmmclust), ref = ref)
    
    formula <- as.formula(paste("dmmclust ~", var, "+ SYNNYTYSTAPA_2luok + BMI_aiti + AIEMMATSYNNYTYKSET_cat"))
    mod <- multinom(formula, data = data, trace = FALSE)
    
    tidy_mod <- tidy(mod, exponentiate = TRUE, conf.int = TRUE)
    
    tidy_hmo <- tidy_mod %>%
      filter(term == var) %>%
      mutate(comparison = paste0(y.level, " vs ", ref))
    
    all_comps[[ref]] <- tidy_hmo
  }
  
  full_results <- bind_rows(all_comps) %>%
    filter(comparison %in% unique_comparisons) %>%
    mutate(
      HMO = var,
      p.value.adj = p.adjust(p.value, method = "fdr"),
      signif_raw = get_signif(p.value),
      signif_adj = get_signif(p.value.adj),
      OR_CI_raw = paste0(
        round(estimate, 4), " (", round(conf.low, 4), "–", round(conf.high, 4), ") ", signif_raw
      ),
      OR_CI_adj = paste0(
        round(estimate, 4), " (", round(conf.low, 4), "–", round(conf.high, 4), ") ", signif_adj
      )
    ) %>%
    select(HMO, comparison, estimate, conf.low, conf.high, p.value, p.value.adj, signif_raw, signif_adj, OR_CI_raw, OR_CI_adj)
  
  return(full_results)
}

# Run for all HMOs
all_hmo_results <- map_dfr(variables, get_or_for_hmo, data = meta3kk_ecx)

# Clean HMO variable names: remove "X_" prefix and "_nmol_scale" or "_scale" suffix
all_hmo_results <- all_hmo_results %>%
  mutate(HMO = gsub("^X_", "", HMO),                # remove leading X_
         HMO = gsub("(_nmol_scale|_scale)$", "", HMO))  # remove trailing _nmol_scale or _scale


# Wide tables
pretty_wide_z_raw4 <- all_hmo_results %>%
  select(HMO, comparison, OR_CI_raw) %>%
  pivot_wider(names_from = comparison, values_from = OR_CI_raw)

pretty_wide_z_adj4 <- all_hmo_results %>%
  select(HMO, comparison, OR_CI_adj) %>%
  pivot_wider(names_from = comparison, values_from = OR_CI_adj)

cat("\n=== ORs with FDR-adjusted p-value Stars ===\n")
```

```
## 
## === ORs with FDR-adjusted p-value Stars ===
```

```
print(pretty_wide_z_adj4)
```

```
## # A tibble: 23 × 4
##    HMO      `2 vs 1`                  `3 vs 1`                   `3 vs 2`       
##    <chr>    <chr>                     <chr>                      <chr>          
##  1 2FL      "1.3343 (0.9816–1.8137) " "0.9848 (0.7–1.3854) "     "0.7381 (0.520…
##  2 3FL      "1.3275 (0.9437–1.8674) " "1.2625 (0.8487–1.8781) "  "0.951 (0.6433…
##  3 LNnT     "0.8315 (0.6191–1.1168) " "0.8531 (0.6024–1.2083) "  "1.026 (0.7071…
##  4 3SL      "0.945 (0.6454–1.3837) "  "0.7291 (0.4373–1.2156) "  "0.7715 (0.454…
##  5 DFLac    "1.0907 (0.7168–1.6596) " "0.4986 (0.2834–0.8772) *" "0.4571 (0.256…
##  6 6SL      "0.8711 (0.6194–1.2252) " "1.2379 (0.8805–1.7404) "  "1.421 (0.9902…
##  7 LNT      "0.9563 (0.7074–1.2927) " "0.9041 (0.6331–1.2911) "  "0.9454 (0.660…
##  8 LNFP_I   "1.062 (0.7872–1.4329) "  "0.8254 (0.5643–1.2074) "  "0.7772 (0.529…
##  9 LNFP_II  "0.7666 (0.5559–1.0572) " "1.2565 (0.901–1.7523) "   "1.639 (1.1444…
## 10 LNFP_III "0.3207 (0.1067–0.9641) " "2.3874 (0.9021–6.3183) "  "7.4457 (2.229…
## # ℹ 13 more rows
```

Secretors and non-secretors

```
subset_03kk_ASV_BF_Exclus_Secretor<- tse_subset_03kk_ASV_BF_Exclus[ , tse_subset_03kk_ASV_BF_Exclus$Secretor %in% c("1")] 

meta3kk_exc_se <- as.data.frame(colData(subset_03kk_ASV_BF_Exclus_Secretor))
```

```
variables <- c(
  "X_2FL_nmol_scale", "X_3FL_nmol_scale", "LNnT_nmol_scale", "X_3SL_nmol_scale", "DFLac_nmol_scale", 
  "X_6SL_nmol_scale", "LNT_nmol_scale", "LNFP_I_nmol_scale", "LNFP_II_nmol_scale", "LNFP_III_nmol_scale", 
  "LSTb_nmol_scale", "LSTc_nmol_scale", "DFLNT_nmol_scale", "LNH_nmol_scale", "DSLNT_nmol_scale", 
  "FLNH_nmol_scale", "DFLNH_nmol_scale", "FDSLNH_nmol_scale", "DSLNH_nmol_scale", "SUM_nmol_scale", 
  "Sia_scale", "Fuc_scale", "Diversity"
)

unique_comparisons <- c("2 vs 1", "3 vs 1", "3 vs 2")

# Significance star helper
get_signif <- function(p) {
  case_when(
    p < 0.001 ~ "***",
    p < 0.01 ~ "**",
    p < 0.05 ~ "*",
    TRUE ~ ""
  )
}

get_or_for_hmo <- function(var, data) {
  refs <- c("1", "2", "3")
  all_comps <- list()
  
  for (ref in refs) {
    data$dmmclust <- relevel(factor(data$dmmclust), ref = ref)
    
    formula <- as.formula(paste("dmmclust ~", var, "+ SYNNYTYSTAPA_2luok + BMI_aiti + AIEMMATSYNNYTYKSET_cat"))
    mod <- multinom(formula, data = data, trace = FALSE)
    
    tidy_mod <- tidy(mod, exponentiate = TRUE, conf.int = TRUE)
    
    tidy_hmo <- tidy_mod %>%
      filter(term == var) %>%
      mutate(comparison = paste0(y.level, " vs ", ref))
    
    all_comps[[ref]] <- tidy_hmo
  }
  
  full_results <- bind_rows(all_comps) %>%
    filter(comparison %in% unique_comparisons) %>%
    mutate(
      HMO = var,
      p.value.adj = p.adjust(p.value, method = "fdr"),
      signif_raw = get_signif(p.value),
      signif_adj = get_signif(p.value.adj),
      OR_CI_raw = paste0(
        round(estimate, 4), " (", round(conf.low, 4), "–", round(conf.high, 4), ") ", signif_raw
      ),
      OR_CI_adj = paste0(
        round(estimate, 4), " (", round(conf.low, 4), "–", round(conf.high, 4), ") ", signif_adj
      )
    ) %>%
    select(HMO, comparison, estimate, conf.low, conf.high, p.value, p.value.adj, signif_raw, signif_adj, OR_CI_raw, OR_CI_adj)
  
  return(full_results)
}

# Run for all HMOs
all_hmo_results <- map_dfr(variables, get_or_for_hmo, data = meta3kk_exc_se)

# Clean HMO variable names: remove "X_" prefix and "_nmol_scale" or "_scale" suffix
all_hmo_results <- all_hmo_results %>%
  mutate(HMO = gsub("^X_", "", HMO),                # remove leading X_
         HMO = gsub("(_nmol_scale|_scale)$", "", HMO))  # remove trailing _nmol_scale or _scale


# Wide tables
pretty_wide_z_raw5 <- all_hmo_results %>%
  select(HMO, comparison, OR_CI_raw) %>%
  pivot_wider(names_from = comparison, values_from = OR_CI_raw)

pretty_wide_z_adj5 <- all_hmo_results %>%
  select(HMO, comparison, OR_CI_adj) %>%
  pivot_wider(names_from = comparison, values_from = OR_CI_adj)
```

### 13 months

Secretor status

```
# Define the outcome levels manually (adjust if needed)
dmm_levels <- c("1", "2", "3", "4")

# Initialize list to store results
all_models_results <- list()

# Loop over reference levels
for (ref_level in dmm_levels) {
  # Relevel the outcome variable
  meta13kk$dmmclust <- relevel(factor(meta13kk$dmmclust), ref = ref_level)
  
  # Fit the multinomial model
  mod <- multinom(dmmclust ~ Secretor + SYNNYTYSTAPA_2luok + BMI_aiti + AIEMMATSYNNYTYKSET_cat, data = meta13kk)
  
  # Extract and tidy results
  tidy_mod <- broom::tidy(mod, conf.int = TRUE, exponentiate = TRUE)
  
  # Label comparisons: each row is "comparison level vs reference"
  tidy_mod <- tidy_mod %>%
    mutate(comparison = paste0(y.level, " vs ", ref_level)) %>%
    select(term, estimate, conf.low, conf.high, comparison)
  
  all_models_results[[ref_level]] <- tidy_mod
}

# Combine all results into one data frame
all_results <- bind_rows(all_models_results)

# Optional: keep only unique comparisons (e.g., not both "2 vs 1" and "1 vs 2")
unique_comparisons <- c("2 vs 1", "3 vs 1", "4 vs 1", "3 vs 2", "4 vs 2", "4 vs 3")
all_results <- all_results %>%
  filter(comparison %in% unique_comparisons)

# Format OR (95% CI)
formatted_results <- all_results %>%
  mutate(OR_CI = paste0(round(estimate, 4), " (", 
                        round(conf.low, 4), "–", 
                        round(conf.high, 4), ")")) %>%
  select(comparison, term, OR_CI)

# Pivot wider: comparisons as columns
pretty_table <- formatted_results %>%
  pivot_wider(names_from = comparison, values_from = OR_CI)
```

Individual HMOs and HMO summary measures

```
variables <- c(
  "X_2FL_nmol_scale", "X_3FL_nmol_scale", "LNnT_nmol_scale", "X_3SL_nmol_scale", "DFLac_nmol_scale", 
  "X_6SL_nmol_scale", "LNT_nmol_scale", "LNFP_I_nmol_scale", "LNFP_II_nmol_scale", "LNFP_III_nmol_scale", 
  "LSTb_nmol_scale", "LSTc_nmol_scale", "DFLNT_nmol_scale", "LNH_nmol_scale", "DSLNT_nmol_scale", 
  "FLNH_nmol_scale", "DFLNH_nmol_scale", "FDSLNH_nmol_scale", "DSLNH_nmol_scale", "SUM_nmol_scale", 
  "Sia_scale", "Fuc_scale", "Diversity"
)

# All pairwise comparisons for 4 groups
unique_comparisons <- c("2 vs 1", "3 vs 1", "4 vs 1", "3 vs 2", "4 vs 2", "4 vs 3")

# Helper to assign significance stars
get_signif <- function(p) {
  case_when(
    p < 0.001 ~ "***",
    p < 0.01 ~ "**",
    p < 0.05 ~ "*",
    TRUE ~ ""
  )
}

# Function to extract ORs and CI per HMO
get_or_for_hmo <- function(var, data) {
  refs <- c("1", "2", "3", "4")
  all_comps <- list()
  
  for (ref in refs) {
    data$dmmclust <- relevel(factor(data$dmmclust), ref = ref)
    
    formula <- as.formula(paste("dmmclust ~", var, "+ SYNNYTYSTAPA_2luok + BMI_aiti + AIEMMATSYNNYTYKSET_cat"))
    mod <- multinom(formula, data = data, trace = FALSE)
    
    tidy_mod <- tidy(mod, exponentiate = TRUE, conf.int = TRUE)
    
    tidy_hmo <- tidy_mod %>%
      filter(term == var) %>%
      mutate(comparison = paste0(y.level, " vs ", ref))
    
    all_comps[[ref]] <- tidy_hmo
  }
  
  full_results <- bind_rows(all_comps) %>%
    filter(comparison %in% unique_comparisons) %>%
    mutate(
      HMO = var,
      p.value.adj = p.adjust(p.value, method = "BH"),
      signif_raw = get_signif(p.value),
      signif_adj = get_signif(p.value.adj),
      OR_CI_raw = paste0(
        round(estimate, 2), " (", round(conf.low, 2), "–", round(conf.high, 2), ") ", signif_raw
      ),
      OR_CI_adj = paste0(
        round(estimate, 2), " (", round(conf.low, 2), "–", round(conf.high, 2), ") ", signif_adj
      )
    ) %>%
    select(HMO, comparison, estimate, conf.low, conf.high, p.value, p.value.adj, signif_raw, signif_adj, OR_CI_raw, OR_CI_adj)
  
  return(full_results)
}

# Run for all HMOs
all_hmo_results <- map_dfr(variables, get_or_for_hmo, data = meta13kk)

# Clean HMO variable names: remove "X_" prefix and "_nmol_scale" or "_scale" suffix
all_hmo_results <- all_hmo_results %>%
  mutate(HMO = gsub("^X_", "", HMO),                # remove leading X_
         HMO = gsub("(_nmol_scale|_scale)$", "", HMO))  # remove trailing _nmol_scale or _scale


# Wide tables
pretty_wide_z_raw9 <- all_hmo_results %>%
  select(HMO, comparison, OR_CI_raw) %>%
  pivot_wider(names_from = comparison, values_from = OR_CI_raw)

pretty_wide_z_adj9 <- all_hmo_results %>%
  select(HMO, comparison, OR_CI_adj) %>%
  pivot_wider(names_from = comparison, values_from = OR_CI_adj)

cat("\n=== ORs with FDR-adjusted p-value Stars ===\n")
```

```
## 
## === ORs with FDR-adjusted p-value Stars ===
```

```
print(pretty_wide_z_adj9)
```

```
## # A tibble: 23 × 7
##    HMO      `4 vs 1`            `3 vs 1`     `2 vs 1` `4 vs 2` `3 vs 2` `4 vs 3`
##    <chr>    <chr>               <chr>        <chr>    <chr>    <chr>    <chr>   
##  1 2FL      "0.95 (0.75–1.22) " "1.26 (0.98… "1.12 (… "0.85 (… "1.12 (… "0.76 (…
##  2 3FL      "0.81 (0.61–1.07) " "1.14 (0.89… "1.09 (… "0.74 (… "1.04 (… "0.71 (…
##  3 LNnT     "1.03 (0.8–1.32) "  "0.96 (0.74… "1.1 (0… "0.94 (… "0.87 (… "1.07 (…
##  4 3SL      "0.86 (0.65–1.12) " "1 (0.79–1.… "0.89 (… "0.96 (… "1.13 (… "0.85 (…
##  5 DFLac    "0.91 (0.7–1.19) "  "0.94 (0.73… "0.94 (… "0.97 (… "1 (0.7… "0.97 (…
##  6 6SL      "1.11 (0.88–1.4) "  "0.88 (0.67… "1.05 (… "1.06 (… "0.84 (… "1.26 (…
##  7 LNT      "1.22 (0.96–1.55) " "1.08 (0.84… "1.08 (… "1.13 (… "1 (0.7… "1.13 (…
##  8 LNFP_I   "1.31 (1.01–1.68) " "1.38 (1.08… "1.11 (… "1.17 (… "1.24 (… "0.95 (…
##  9 LNFP_II  "0.87 (0.68–1.11) " "0.74 (0.57… "0.91 (… "0.96 (… "0.81 (… "1.18 (…
## 10 LNFP_III "0.77 (0.34–1.74) " "0.51 (0.21… "1.33 (… "0.58 (… "0.39 (… "1.5 (0…
## # ℹ 13 more rows
```

Secretors and non-secretors

```
subset_13kk_ASV_Secretor<- tse_subset_by_13kk_ASV[ , tse_subset_by_13kk_ASV$Secretor %in% c("1")] 

meta13kk_se <- as.data.frame(colData(subset_13kk_ASV_Secretor))

variables <- c(
  "X_2FL_nmol_scale", "X_3FL_nmol_scale", "LNnT_nmol_scale", "X_3SL_nmol_scale", "DFLac_nmol_scale", 
  "X_6SL_nmol_scale", "LNT_nmol_scale", "LNFP_I_nmol_scale", "LNFP_II_nmol_scale", "LNFP_III_nmol_scale", 
  "LSTb_nmol_scale", "LSTc_nmol_scale", "DFLNT_nmol_scale", "LNH_nmol_scale", "DSLNT_nmol_scale", 
  "FLNH_nmol_scale", "DFLNH_nmol_scale", "FDSLNH_nmol_scale", "DSLNH_nmol_scale", "SUM_nmol_scale", 
  "Sia_scale", "Fuc_scale", "Diversity"
)

# All pairwise comparisons for 4 groups
unique_comparisons <- c("2 vs 1", "3 vs 1", "4 vs 1", "3 vs 2", "4 vs 2", "4 vs 3")

# Helper to assign significance stars
get_signif <- function(p) {
  case_when(
    p < 0.001 ~ "***",
    p < 0.01 ~ "**",
    p < 0.05 ~ "*",
    TRUE ~ ""
  )
}

# Function to extract ORs and CI per HMO
get_or_for_hmo <- function(var, data) {
  refs <- c("1", "2", "3", "4")
  all_comps <- list()
  
  for (ref in refs) {
    data$dmmclust <- relevel(factor(data$dmmclust), ref = ref)
    
    formula <- as.formula(paste("dmmclust ~", var, "+ SYNNYTYSTAPA_2luok + BMI_aiti + AIEMMATSYNNYTYKSET_cat"))
    mod <- multinom(formula, data = data, trace = FALSE)
    
    tidy_mod <- tidy(mod, exponentiate = TRUE, conf.int = TRUE)
    
    tidy_hmo <- tidy_mod %>%
      filter(term == var) %>%
      mutate(comparison = paste0(y.level, " vs ", ref))
    
    all_comps[[ref]] <- tidy_hmo
  }
  
  full_results <- bind_rows(all_comps) %>%
    filter(comparison %in% unique_comparisons) %>%
    mutate(
      HMO = var,
      p.value.adj = p.adjust(p.value, method = "fdr"),
      signif_raw = get_signif(p.value),
      signif_adj = get_signif(p.value.adj),
      OR_CI_raw = paste0(
        round(estimate, 4), " (", round(conf.low, 4), "–", round(conf.high, 4), ") ", signif_raw
      ),
      OR_CI_adj = paste0(
        round(estimate, 4), " (", round(conf.low, 4), "–", round(conf.high, 4), ") ", signif_adj
      )
    ) %>%
    select(HMO, comparison, estimate, conf.low, conf.high, p.value, p.value.adj, signif_raw, signif_adj, OR_CI_raw, OR_CI_adj)
  
  return(full_results)
}

# Run for all HMOs
all_hmo_results <- map_dfr(variables, get_or_for_hmo, data = meta13kk_se)

# Clean HMO variable names: remove "X_" prefix and "_nmol_scale" or "_scale" suffix
all_hmo_results <- all_hmo_results %>%
  mutate(HMO = gsub("^X_", "", HMO),                # remove leading X_
         HMO = gsub("(_nmol_scale|_scale)$", "", HMO))  # remove trailing _nmol_scale or _scale

# Clean HMO variable names: remove "X_" prefix and "_nmol_scale" or "_scale" suffix
all_hmo_results <- all_hmo_results %>%
  mutate(HMO = gsub("^X_", "", HMO),                # remove leading X_
         HMO = gsub("(_nmol_scale|_scale)$", "", HMO))  # remove trailing _nmol_scale or _scale

# Wide tables
pretty_wide_z_raw6 <- all_hmo_results %>%
  select(HMO, comparison, OR_CI_raw) %>%
  pivot_wider(names_from = comparison, values_from = OR_CI_raw)

pretty_wide_z_adj6 <- all_hmo_results %>%
  select(HMO, comparison, OR_CI_adj) %>%
  pivot_wider(names_from = comparison, values_from = OR_CI_adj)
```

```
subset_13kk_ASV_nonsecretor<- tse_subset_by_13kk_ASV[ , tse_subset_by_13kk_ASV$Secretor %in% c("0")] 

meta13kk_nse <- as.data.frame(colData(subset_13kk_ASV_nonsecretor))

variables <- c(
  "X_2FL_nmol_scale", "X_3FL_nmol_scale", "LNnT_nmol_scale", "X_3SL_nmol_scale", "DFLac_nmol_scale", 
  "X_6SL_nmol_scale", "LNT_nmol_scale", "LNFP_I_nmol_scale", "LNFP_II_nmol_scale", "LNFP_III_nmol_scale", 
  "LSTb_nmol_scale", "LSTc_nmol_scale", "DFLNT_nmol_scale", "LNH_nmol_scale", "DSLNT_nmol_scale", 
  "FLNH_nmol_scale", "DFLNH_nmol_scale", "FDSLNH_nmol_scale", "DSLNH_nmol_scale", "SUM_nmol_scale", 
  "Sia_scale", "Fuc_scale", "Diversity"
)

# All pairwise comparisons for 4 groups
unique_comparisons <- c("2 vs 1", "3 vs 1", "4 vs 1", "3 vs 2", "4 vs 2", "4 vs 3")

# Helper to assign significance stars
get_signif <- function(p) {
  case_when(
    p < 0.001 ~ "***",
    p < 0.01 ~ "**",
    p < 0.05 ~ "*",
    TRUE ~ ""
  )
}

# Function to extract ORs and CI per HMO
get_or_for_hmo <- function(var, data) {
  refs <- c("1", "2", "3", "4")
  all_comps <- list()
  
  for (ref in refs) {
    data$dmmclust <- relevel(factor(data$dmmclust), ref = ref)
    
    formula <- as.formula(paste("dmmclust ~", var, "+ SYNNYTYSTAPA_2luok + BMI_aiti + AIEMMATSYNNYTYKSET_cat"))
    mod <- multinom(formula, data = data, trace = FALSE)
    
    tidy_mod <- tidy(mod, exponentiate = TRUE, conf.int = TRUE)
    
    tidy_hmo <- tidy_mod %>%
      filter(term == var) %>%
      mutate(comparison = paste0(y.level, " vs ", ref))
    
    all_comps[[ref]] <- tidy_hmo
  }
  
  full_results <- bind_rows(all_comps) %>%
    filter(comparison %in% unique_comparisons) %>%
    mutate(
      HMO = var,
      p.value.adj = p.adjust(p.value, method = "fdr"),
      signif_raw = get_signif(p.value),
      signif_adj = get_signif(p.value.adj),
      OR_CI_raw = paste0(
        round(estimate, 4), " (", round(conf.low, 4), "–", round(conf.high, 4), ") ", signif_raw
      ),
      OR_CI_adj = paste0(
        round(estimate, 4), " (", round(conf.low, 4), "–", round(conf.high, 4), ") ", signif_adj
      )
    ) %>%
    select(HMO, comparison, estimate, conf.low, conf.high, p.value, p.value.adj, signif_raw, signif_adj, OR_CI_raw, OR_CI_adj)
  
  return(full_results)
}

# Run for all HMOs
all_hmo_results <- map_dfr(variables, get_or_for_hmo, data = meta13kk_nse)

# Clean HMO variable names: remove "X_" prefix and "_nmol_scale" or "_scale" suffix
all_hmo_results <- all_hmo_results %>%
  mutate(HMO = gsub("^X_", "", HMO),                # remove leading X_
         HMO = gsub("(_nmol_scale|_scale)$", "", HMO))  # remove trailing _nmol_scale or _scale

# Wide tables
pretty_wide_z_raw7 <- all_hmo_results %>%
  select(HMO, comparison, OR_CI_raw) %>%
  pivot_wider(names_from = comparison, values_from = OR_CI_raw)

pretty_wide_z_adj7 <- all_hmo_results %>%
  select(HMO, comparison, OR_CI_adj) %>%
  pivot_wider(names_from = comparison, values_from = OR_CI_adj)
```

```
#raw p-values
all_data <- list(
  meta3kk = pretty_wide_z_raw,
  meta3kk_se = pretty_wide_z_raw2,
  meta3kk_nse = pretty_wide_z_raw3,
  meta3kk_ecx = pretty_wide_z_raw4,
  meta3kk_exc_se = pretty_wide_z_raw5,
  meta13kk = pretty_wide_z_raw9,
  meta13kk_se = pretty_wide_z_raw6,
  meta13kk_nse = pretty_wide_z_raw7
)

# Write to Excel file with each data frame in its own sheet
#write_xlsx(all_data, path = "multinom_log_reg.xlsx")

#adjusted-palues
all_data_adj <- list(
  meta3kk = pretty_wide_z_adj,
  meta3kk_se = pretty_wide_z_adj2,
  meta3kk_nse = pretty_wide_z_adj3,
  meta3kk_ecx = pretty_wide_z_adj4,
  meta3kk_exc_se = pretty_wide_z_adj5,
  meta13kk = pretty_wide_z_adj9,
  meta13kk_se = pretty_wide_z_adj6,
  meta13kk_nse = pretty_wide_z_adj7
)

# Write to Excel file with each data frame in its own sheet
#write_xlsx(all_data_adj, path = "multinom_log_reg_adj.xlsx")
```

# Beta dversity

ordistep + permanova analysis

```
tse_subset_by_03kk_ASV <- transformAssay(
     tse_subset_by_03kk_ASV, assay.type = "counts", method = "relabundance")


#Late infancy

tse_subset_by_13kk_ASV <- transformAssay(
     tse_subset_by_13kk_ASV, assay.type = "counts", method = "relabundance")

data3kk <- dplyr::select(meta3kk, 
                       c("nro", "LNnT_nmol", "BMI_aiti", "AIEMMATSYNNYTYKSET_cat", "SYNNYTYSTAPA_2luok", "dmmclust"))

data3kk1 <- na.omit(data3kk)

tse_subset_PERMANOVA<- tse_subset_by_03kk_ASV[ , tse_subset_by_03kk_ASV$nro %in% c(data3kk1$nro)]
```

Test the significance of the secretor status

```
tse_subset_PERMANOVA <- transformAssay(tse_subset_PERMANOVA, assay.type = "counts", method = "relabundance")

method <- "relabundance"
diss_method <- "bray"

set.seed(123)

permanova <- adonis2(t(assay(tse_subset_PERMANOVA, method)) ~ Secretor + BMI_aiti + AIEMMATSYNNYTYKSET_cat +  SYNNYTYSTAPA_2luok  + lib_size,
                     by = "margin",
                     data = colData(tse_subset_PERMANOVA),
                     method = diss_method,
                     permutations = 999)

permanova
```

```
## Permutation test for adonis under reduced model
## Marginal effects of terms
## Permutation: free
## Number of permutations: 999
## 
## adonis2(formula = t(assay(tse_subset_PERMANOVA, method)) ~ Secretor + BMI_aiti + AIEMMATSYNNYTYKSET_cat + SYNNYTYSTAPA_2luok + lib_size, data = colData(tse_subset_PERMANOVA), permutations = 999, method = diss_method, by = "margin")
##                         Df SumOfSqs      R2      F Pr(>F)    
## Secretor                 1    0.687 0.00318 1.6720  0.039 *  
## BMI_aiti                 1    0.592 0.00274 1.4415  0.102    
## AIEMMATSYNNYTYKSET_cat   1    1.766 0.00817 4.2997  0.001 ***
## SYNNYTYSTAPA_2luok       1    1.410 0.00653 3.4344  0.001 ***
## lib_size                 1    1.715 0.00794 4.1762  0.001 ***
## Residual               510  209.432 0.96962                  
## Total                  515  215.995 1.00000                  
## ---
## Signif. codes:  0 '***' 0.001 '**' 0.01 '*' 0.05 '.' 0.1 ' ' 1
```

Test the same also in genus level

```
tse_genus_3kk <- altExp(tse_subset_by_03kk_ASV, "Genus")

colData(tse_genus_3kk)$dmmclust <- as.data.frame(colData(tse_subset_by_03kk_ASV))$dmmclust

meta_3kk_g <- as.data.frame(colData(tse_genus_3kk))


data3kk <- dplyr::select(meta_3kk_g, 
                       c("nro", "LNnT_nmol", "BMI_aiti", "AIEMMATSYNNYTYKSET_cat", "SYNNYTYSTAPA_2luok", "dmmclust"))

data3kk1 <- na.omit(data3kk)

tse_subset_PERMANOVA<- tse_genus_3kk[ , tse_genus_3kk$nro %in% c(data3kk1$nro)]
```

```
tse_subset_PERMANOVA <- transformAssay(tse_subset_PERMANOVA, assay.type = "counts", method = "relabundance")

method <- "relabundance"
diss_method <- "bray"

set.seed(123)

permanova <- adonis2(t(assay(tse_subset_PERMANOVA, method)) ~ Secretor + BMI_aiti + AIEMMATSYNNYTYKSET_cat +  SYNNYTYSTAPA_2luok  ,
                     by = "margin",
                     data = colData(tse_subset_PERMANOVA),
                     method = diss_method,
                     permutations = 999)

permanova
```

```
## Permutation test for adonis under reduced model
## Marginal effects of terms
## Permutation: free
## Number of permutations: 999
## 
## adonis2(formula = t(assay(tse_subset_PERMANOVA, method)) ~ Secretor + BMI_aiti + AIEMMATSYNNYTYKSET_cat + SYNNYTYSTAPA_2luok, data = colData(tse_subset_PERMANOVA), permutations = 999, method = diss_method, by = "margin")
##                         Df SumOfSqs      R2       F Pr(>F)    
## Secretor                 1    0.779 0.00722  3.9397  0.003 ** 
## BMI_aiti                 1    0.142 0.00131  0.7163  0.537    
## AIEMMATSYNNYTYKSET_cat   1    1.319 0.01222  6.6688  0.001 ***
## SYNNYTYSTAPA_2luok       1    4.296 0.03980 21.7133  0.001 ***
## Residual               511  101.092 0.93666                   
## Total                  515  107.928 1.00000                   
## ---
## Signif. codes:  0 '***' 0.001 '**' 0.01 '*' 0.05 '.' 0.1 ' ' 1
```

```
bd <- betadisper(
  vegdist(t(assay(tse_subset_PERMANOVA, method)), method = diss_method), 
  colData(tse_subset_PERMANOVA)$Secretor)

permutest(bd)
```

```
## 
## Permutation test for homogeneity of multivariate dispersions
## Permutation: free
## Number of permutations: 999
## 
## Response: Distances
##            Df  Sum Sq Mean Sq      F N.Perm Pr(>F)   
## Groups      1  0.3792 0.37917 8.2452    999  0.006 **
## Residuals 514 23.6371 0.04599                        
## ---
## Signif. codes:  0 '***' 0.001 '**' 0.01 '*' 0.05 '.' 0.1 ' ' 1
```

## 3 months

```
#Early infancy

tse_subset_by_03kk_ASV <- transformAssay(
     tse_subset_by_03kk_ASV, assay.type = "counts", method = "relabundance")


#Late infancy

tse_subset_by_13kk_ASV <- transformAssay(
     tse_subset_by_13kk_ASV, assay.type = "counts", method = "relabundance")


data3kk <- dplyr::select(meta3kk, 
                       c("nro", "LNnT_nmol", "BMI_aiti", "AIEMMATSYNNYTYKSET_cat", "SYNNYTYSTAPA_2luok"))

data3kk1 <- na.omit(data3kk)

tse_subset_PERMANOVA<- tse_subset_by_03kk_ASV[ , tse_subset_by_03kk_ASV$nro %in% c(data3kk1$nro)]

meta3kk_All <- as.data.frame(colData(tse_subset_PERMANOVA))
```

OORDISTEP USING DIST MATRIX

```
tse_subset_PERMANOVA <- transformAssay(
     tse_subset_PERMANOVA, assay.type = "counts", method = "relabundance")

set.seed(123)

#Calculate Bray-Curtis dist. matrix
D_relabundance <- vegdist(t(assays(tse_subset_PERMANOVA)$relabundance), method="bray")
```

Scaled HMO data 3 month

```
#select HMO data  
meta3kk_HMO <- dplyr::select(meta3kk_All, 
                       c("sample_ID","X_2FL_nmol_scale", "X_3FL_nmol_scale", "LNnT_nmol_scale", "X_3SL_nmol_scale", "DFLac_nmol_scale", 
                 "X_6SL_nmol_scale", "LNT_nmol_scale", "LNFP_I_nmol_scale", "LNFP_II_nmol_scale", "LNFP_III_nmol_scale", 
                 "LSTb_nmol_scale", "LSTc_nmol_scale", "DFLNT_nmol_scale", "LNH_nmol_scale", "DSLNT_nmol_scale", 
                 "FLNH_nmol_scale", "DFLNH_nmol_scale", "FDSLNH_nmol_scale", "DSLNH_nmol_scale"))

m0 <- vegan::capscale(D_relabundance ~ 1, data = meta3kk_HMO[2:20])  
m1 <- vegan::capscale(D_relabundance ~ ., data = meta3kk_HMO[2:20])
set.seed(123)
m <- vegan::ordistep(m0, scope = formula(m1), direction = "both")
```

```
## 
## Start: D_relabundance ~ 1 
## 
##                       Df    AIC      F Pr(>F)  
## + LNFP_III_nmol_scale  1 2862.3 1.2951  0.030 *
## + FDSLNH_nmol_scale    1 2862.2 1.3970  0.080 .
## + LNFP_II_nmol_scale   1 2862.2 1.3308  0.160  
## + DSLNT_nmol_scale     1 2862.3 1.2477  0.170  
## + LNH_nmol_scale       1 2862.4 1.1912  0.230  
## + LNFP_I_nmol_scale    1 2862.4 1.1752  0.240  
## + LSTb_nmol_scale      1 2862.4 1.1655  0.240  
## + FLNH_nmol_scale      1 2862.5 1.1196  0.260  
## + DSLNH_nmol_scale     1 2862.5 1.0957  0.310  
## + LNT_nmol_scale       1 2862.5 1.0365  0.365  
## + DFLac_nmol_scale     1 2862.5 1.0782  0.375  
## + X_2FL_nmol_scale     1 2862.5 1.0772  0.400  
## + DFLNH_nmol_scale     1 2862.6 0.9864  0.400  
## + X_6SL_nmol_scale     1 2862.6 0.9694  0.470  
## + LSTc_nmol_scale      1 2862.6 0.9805  0.540  
## + X_3FL_nmol_scale     1 2862.8 0.8055  0.670  
## + DFLNT_nmol_scale     1 2862.7 0.8529  0.690  
## + X_3SL_nmol_scale     1 2862.9 0.6785  0.930  
## + LNnT_nmol_scale      1 2863.0 0.6155  0.980  
## ---
## Signif. codes:  0 '***' 0.001 '**' 0.01 '*' 0.05 '.' 0.1 ' ' 1
## 
## Step: D_relabundance ~ LNFP_III_nmol_scale 
## 
##                       Df    AIC      F Pr(>F)  
## - LNFP_III_nmol_scale  1 2861.6 1.2951  0.045 *
## ---
## Signif. codes:  0 '***' 0.001 '**' 0.01 '*' 0.05 '.' 0.1 ' ' 1
## 
##                      Df    AIC      F Pr(>F)
## + LNFP_II_nmol_scale  1 2863.0 1.2539  0.135
## + FDSLNH_nmol_scale   1 2863.0 1.2917  0.140
## + DSLNT_nmol_scale    1 2863.0 1.2518  0.165
## + FLNH_nmol_scale     1 2863.1 1.2140  0.190
## + DFLNH_nmol_scale    1 2863.1 1.1304  0.270
## + LSTb_nmol_scale     1 2863.1 1.1596  0.285
## + DFLac_nmol_scale    1 2863.2 1.0583  0.345
## + DSLNH_nmol_scale    1 2863.2 1.0660  0.380
## + LNT_nmol_scale      1 2863.2 1.0352  0.405
## + LNFP_I_nmol_scale   1 2863.3 1.0059  0.415
## + LSTc_nmol_scale     1 2863.3 1.0057  0.445
## + X_6SL_nmol_scale    1 2863.3 0.9457  0.530
## + LNH_nmol_scale      1 2863.3 0.9383  0.555
## + DFLNT_nmol_scale    1 2863.4 0.8243  0.655
## + X_2FL_nmol_scale    1 2863.4 0.8606  0.690
## + X_3FL_nmol_scale    1 2863.5 0.8129  0.735
## + X_3SL_nmol_scale    1 2863.6 0.6856  0.910
## + LNnT_nmol_scale     1 2863.7 0.5933  0.985
```

HMO groups + Secretor 3 month

```
#select HMO data  
meta3kk_HMO_groups <- dplyr::select(meta3kk_All, 
                       c("sample_ID", "Secretor","SUM_nmol", "Sia","Fuc","Diversity"))


m0 <- vegan::capscale(D_relabundance ~ 1, data = meta3kk_HMO_groups[2:5])  
m1 <- vegan::capscale(D_relabundance ~ ., data = meta3kk_HMO_groups[2:5])
set.seed(123)
m <- vegan::ordistep(m0, scope = formula(m1))
```

```
## 
## Start: D_relabundance ~ 1 
## 
##            Df    AIC      F Pr(>F)  
## + Secretor  1 2861.9 1.6865  0.030 *
## + SUM_nmol  1 2862.2 1.3330  0.100 .
## + Sia       1 2862.4 1.1472  0.245  
## + Fuc       1 2862.4 1.1415  0.280  
## ---
## Signif. codes:  0 '***' 0.001 '**' 0.01 '*' 0.05 '.' 0.1 ' ' 1
## 
## Step: D_relabundance ~ Secretor 
## 
##            Df    AIC      F Pr(>F)  
## - Secretor  1 2861.6 1.6865   0.02 *
## ---
## Signif. codes:  0 '***' 0.001 '**' 0.01 '*' 0.05 '.' 0.1 ' ' 1
## 
##            Df    AIC      F Pr(>F)
## + Sia       1 2862.8 1.0862   0.37
## + Fuc       1 2863.1 0.7994   0.81
## + SUM_nmol  1 2863.3 0.6082   0.98
```

```
method <- "relabundance"
diss_method <- "bray"

set.seed(123)

permanova <- adonis2(t(assay(tse_subset_PERMANOVA, method)) ~  Secretor + BMI_aiti+ AIEMMATSYNNYTYKSET_cat +  SYNNYTYSTAPA_2luok ,
                     by = "margin",
                     data = colData(tse_subset_PERMANOVA),
                     method = diss_method,
                     permutations = 999)

permanova
```

```
## Permutation test for adonis under reduced model
## Marginal effects of terms
## Permutation: free
## Number of permutations: 999
## 
## adonis2(formula = t(assay(tse_subset_PERMANOVA, method)) ~ Secretor + BMI_aiti + AIEMMATSYNNYTYKSET_cat + SYNNYTYSTAPA_2luok, data = colData(tse_subset_PERMANOVA), permutations = 999, method = diss_method, by = "margin")
##                         Df SumOfSqs      R2      F Pr(>F)    
## Secretor                 1    0.687 0.00318 1.6635  0.039 *  
## BMI_aiti                 1    0.592 0.00274 1.4337  0.105    
## AIEMMATSYNNYTYKSET_cat   1    1.744 0.00807 4.2209  0.001 ***
## SYNNYTYSTAPA_2luok       1    1.657 0.00767 4.0113  0.001 ***
## Residual               511  211.147 0.97756                  
## Total                  515  215.995 1.00000                  
## ---
## Signif. codes:  0 '***' 0.001 '**' 0.01 '*' 0.05 '.' 0.1 ' ' 1
```

Subset secretor status

```
#subset secretor

tse_subset_03kk_secretor <- tse_subset_by_03kk_ASV[ , tse_subset_by_03kk_ASV$Secretor %in% c("1")] 

tse_subset_03kk_nonsecretor <- tse_subset_by_03kk_ASV[ , tse_subset_by_03kk_ASV$Secretor %in% c("0")]
```

```
meta3kk_s <- as.data.frame(colData(tse_subset_03kk_secretor))

data3kk <- dplyr::select(meta3kk_s, 
                       c("nro", "LNFP_III_nmol",  "BMI_aiti", "AIEMMATSYNNYTYKSET_cat", "SYNNYTYSTAPA_2luok"))

data3kk1 <- na.omit(data3kk)

tse_subset_PERMANOVA_s<- tse_subset_03kk_secretor[ , tse_subset_03kk_secretor$nro %in% c(data3kk1$nro)]

meta3kk_sec <- as.data.frame(colData(tse_subset_PERMANOVA_s))
```

```
tse_subset_PERMANOVA_s <- transformAssay(tse_subset_PERMANOVA_s, assay.type = "counts", method = "relabundance")

set.seed(123)

D_relabundance <- vegdist(t(assays(tse_subset_PERMANOVA_s)$relabundance), method="bray")
```

Independent HMOs subset secretors

```
#select HMO data  
meta3kk_HMO <- dplyr::select(meta3kk_sec, 
                       c("sample_ID","X_2FL_nmol_scale", "X_3FL_nmol_scale", "LNnT_nmol_scale", "X_3SL_nmol_scale", "DFLac_nmol_scale", 
                 "X_6SL_nmol_scale", "LNT_nmol_scale", "LNFP_I_nmol_scale", "LNFP_II_nmol_scale", "LNFP_III_nmol_scale", 
                 "LSTb_nmol_scale", "LSTc_nmol_scale", "DFLNT_nmol_scale", "LNH_nmol_scale", "DSLNT_nmol_scale", 
                 "FLNH_nmol_scale", "DFLNH_nmol_scale", "FDSLNH_nmol_scale", "DSLNH_nmol_scale"))


m0 <- vegan::capscale(D_relabundance ~ 1, data = meta3kk_HMO[2:20])  
m1 <- vegan::capscale(D_relabundance ~ ., data = meta3kk_HMO[2:20])
set.seed(123)
m <- vegan::ordistep(m0, scope = formula(m1), direction = "both")
```

```
## 
## Start: D_relabundance ~ 1 
## 
##                       Df    AIC      F Pr(>F)
## + FDSLNH_nmol_scale    1 2508.0 1.2790  0.120
## + FLNH_nmol_scale      1 2508.0 1.2573  0.160
## + DSLNT_nmol_scale     1 2508.0 1.2473  0.170
## + DSLNH_nmol_scale     1 2508.1 1.1582  0.190
## + DFLNH_nmol_scale     1 2508.1 1.2203  0.265
## + LSTb_nmol_scale      1 2508.2 1.0610  0.335
## + LNFP_III_nmol_scale  1 2508.2 1.0816  0.345
## + LNH_nmol_scale       1 2508.3 0.9561  0.480
## + LSTc_nmol_scale      1 2508.3 0.9623  0.485
## + X_3FL_nmol_scale     1 2508.3 0.9646  0.515
## + LNT_nmol_scale       1 2508.3 0.9387  0.525
## + DFLac_nmol_scale     1 2508.3 0.9524  0.605
## + LNFP_II_nmol_scale   1 2508.4 0.9148  0.615
## + X_2FL_nmol_scale     1 2508.5 0.7718  0.785
## + LNFP_I_nmol_scale    1 2508.5 0.7652  0.815
## + X_3SL_nmol_scale     1 2508.6 0.6951  0.865
## + LNnT_nmol_scale      1 2508.6 0.6687  0.925
## + DFLNT_nmol_scale     1 2508.6 0.6668  0.925
## + X_6SL_nmol_scale     1 2508.7 0.5416  0.980
```

HMO groups subset secretors

```
#select HMO data  
meta3kk_HMO_groups <- dplyr::select(meta3kk_sec, 
                       c("sample_ID","SUM_nmol", "Sia","Fuc","Diversity"))


m0 <- vegan::capscale(D_relabundance ~ 1, data = meta3kk_HMO_groups[2:5])  
m1 <- vegan::capscale(D_relabundance ~ ., data = meta3kk_HMO_groups[2:5])
set.seed(123)
m <- vegan::ordistep(m0, scope = formula(m1))
```

```
## 
## Start: D_relabundance ~ 1 
## 
##             Df    AIC      F Pr(>F)
## + Sia        1 2508.0 1.2913  0.135
## + Diversity  1 2508.5 0.7513  0.805
## + SUM_nmol   1 2508.6 0.7272  0.870
## + Fuc        1 2508.6 0.6429  0.940
```

```
method <- "relabundance"
diss_method <- "bray"

set.seed(123)

permanova <- adonis2(t(assay(tse_subset_PERMANOVA_s, method)) ~  DFLNH_nmol + BMI_aiti + AIEMMATSYNNYTYKSET_cat +  SYNNYTYSTAPA_2luok  ,
                     by = "margin",
                     data = colData(tse_subset_PERMANOVA_s),
                     method = diss_method,
                     permutations = 999)
```

## 3 month Exclusivelly BF

```
tse_subset_03kk_BF_Exclus <- tse_subset_by_03kk_ASV[ , tse_subset_by_03kk_ASV$breastfeeding_3kk_category %in% c("exclusive")] 

data3kk <- dplyr::select(meta3kk, 
                       c("nro", "LNnT_nmol", "BMI_aiti", "AIEMMATSYNNYTYKSET_cat", "SYNNYTYSTAPA_2luok"))

data3kk1 <- na.omit(data3kk)

tse_subset_PERMANOVA <- tse_subset_03kk_BF_Exclus[ , tse_subset_03kk_BF_Exclus$nro %in% c(data3kk1$nro)]

meta3kk_Excl <- as.data.frame(colData(tse_subset_PERMANOVA))
```

OORDISTEP USING DIST MATRIX

```
tse_subset_PERMANOVA <- transformAssay(tse_subset_PERMANOVA, assay.type = "counts", method = "relabundance")

set.seed(123)
D_relabundance <- vegdist(t(assays(tse_subset_PERMANOVA)$relabundance), method="bray")
```

scaled HMO data

```
#select HMO data  
meta3kk_HMO <- dplyr::select(meta3kk_Excl, 
                       c("sample_ID","X_2FL_nmol_scale", "X_3FL_nmol_scale", "LNnT_nmol_scale", "X_3SL_nmol_scale", "DFLac_nmol_scale", 
                 "X_6SL_nmol_scale", "LNT_nmol_scale", "LNFP_I_nmol_scale", "LNFP_II_nmol_scale", "LNFP_III_nmol_scale", 
                 "LSTb_nmol_scale", "LSTc_nmol_scale", "DFLNT_nmol_scale", "LNH_nmol_scale", "DSLNT_nmol_scale", 
                 "FLNH_nmol_scale", "DFLNH_nmol_scale", "FDSLNH_nmol_scale", "DSLNH_nmol_scale"))


m0 <- vegan::capscale(D_relabundance ~ 1, data = meta3kk_HMO[2:20])  
m1 <- vegan::capscale(D_relabundance ~ ., data = meta3kk_HMO[2:20])
set.seed(123)
m <- vegan::ordistep(m0, scope = formula(m1), direction = "both")
```

```
## 
## Start: D_relabundance ~ 1 
## 
##                       Df    AIC      F Pr(>F)  
## + DSLNT_nmol_scale     1 1245.7 1.5125  0.045 *
## + FLNH_nmol_scale      1 1245.7 1.4571  0.050 *
## + DFLac_nmol_scale     1 1245.6 1.5720  0.065 .
## + LSTc_nmol_scale      1 1245.8 1.3584  0.075 .
## + LNFP_II_nmol_scale   1 1245.8 1.3784  0.110  
## + DFLNH_nmol_scale     1 1246.0 1.2418  0.160  
## + FDSLNH_nmol_scale    1 1246.1 1.1406  0.200  
## + DFLNT_nmol_scale     1 1245.9 1.2696  0.205  
## + LNFP_I_nmol_scale    1 1246.1 1.0610  0.365  
## + LNFP_III_nmol_scale  1 1246.2 1.0059  0.400  
## + X_3SL_nmol_scale     1 1246.2 1.0015  0.440  
## + LNT_nmol_scale       1 1246.2 0.9705  0.445  
## + LNH_nmol_scale       1 1246.3 0.9323  0.505  
## + X_6SL_nmol_scale     1 1246.2 0.9533  0.520  
## + X_2FL_nmol_scale     1 1246.3 0.9322  0.565  
## + DSLNH_nmol_scale     1 1246.3 0.8792  0.595  
## + LNnT_nmol_scale      1 1246.4 0.8097  0.755  
## + LSTb_nmol_scale      1 1246.5 0.7330  0.840  
## + X_3FL_nmol_scale     1 1246.7 0.5234  0.990  
## ---
## Signif. codes:  0 '***' 0.001 '**' 0.01 '*' 0.05 '.' 0.1 ' ' 1
## 
## Step: D_relabundance ~ DSLNT_nmol_scale 
## 
##                    Df    AIC      F Pr(>F)  
## - DSLNT_nmol_scale  1 1245.2 1.5125  0.065 .
## ---
## Signif. codes:  0 '***' 0.001 '**' 0.01 '*' 0.05 '.' 0.1 ' ' 1
## 
##                       Df    AIC      F Pr(>F)  
## + DFLac_nmol_scale     1 1246.1 1.5952  0.050 *
## + FLNH_nmol_scale      1 1246.1 1.5419  0.060 .
## + LNFP_II_nmol_scale   1 1246.2 1.4442  0.065 .
## + LSTc_nmol_scale      1 1246.3 1.4097  0.075 .
## + DFLNT_nmol_scale     1 1246.4 1.2386  0.165  
## + DFLNH_nmol_scale     1 1246.4 1.2491  0.200  
## + X_3SL_nmol_scale     1 1246.5 1.1485  0.290  
## + FDSLNH_nmol_scale    1 1246.6 1.1103  0.295  
## + X_6SL_nmol_scale     1 1246.6 1.0541  0.360  
## + X_2FL_nmol_scale     1 1246.6 1.0541  0.375  
## + LNFP_I_nmol_scale    1 1246.6 1.0468  0.380  
## + LNFP_III_nmol_scale  1 1246.7 1.0118  0.400  
## + LNH_nmol_scale       1 1246.7 0.9857  0.480  
## + LNT_nmol_scale       1 1246.8 0.9185  0.535  
## + DSLNH_nmol_scale     1 1246.8 0.8841  0.625  
## + LSTb_nmol_scale      1 1247.0 0.7125  0.845  
## + LNnT_nmol_scale      1 1247.0 0.6742  0.905  
## + X_3FL_nmol_scale     1 1247.0 0.6289  0.940  
## ---
## Signif. codes:  0 '***' 0.001 '**' 0.01 '*' 0.05 '.' 0.1 ' ' 1
## 
## Step: D_relabundance ~ DSLNT_nmol_scale + DFLac_nmol_scale 
## 
##                    Df    AIC      F Pr(>F)  
## - DSLNT_nmol_scale  1 1245.6 1.5359   0.04 *
## - DFLac_nmol_scale  1 1245.7 1.5952   0.04 *
## ---
## Signif. codes:  0 '***' 0.001 '**' 0.01 '*' 0.05 '.' 0.1 ' ' 1
## 
##                       Df    AIC      F Pr(>F)  
## + FLNH_nmol_scale      1 1246.5 1.5174  0.055 .
## + LNFP_II_nmol_scale   1 1246.8 1.2911  0.170  
## + LSTc_nmol_scale      1 1246.8 1.2269  0.180  
## + DFLNH_nmol_scale     1 1246.9 1.1945  0.185  
## + LNFP_I_nmol_scale    1 1246.9 1.1362  0.300  
## + X_2FL_nmol_scale     1 1247.1 0.9460  0.425  
## + LNT_nmol_scale       1 1247.1 0.9695  0.465  
## + LNH_nmol_scale       1 1247.1 0.9889  0.480  
## + X_3SL_nmol_scale     1 1247.1 0.9369  0.495  
## + DFLNT_nmol_scale     1 1247.2 0.8870  0.595  
## + DSLNH_nmol_scale     1 1247.2 0.8984  0.600  
## + LNFP_III_nmol_scale  1 1247.2 0.9101  0.610  
## + LNnT_nmol_scale      1 1247.3 0.8085  0.700  
## + X_6SL_nmol_scale     1 1247.3 0.8073  0.735  
## + FDSLNH_nmol_scale    1 1247.3 0.8054  0.740  
## + LSTb_nmol_scale      1 1247.3 0.7537  0.775  
## + X_3FL_nmol_scale     1 1247.6 0.5032  0.990  
## ---
## Signif. codes:  0 '***' 0.001 '**' 0.01 '*' 0.05 '.' 0.1 ' ' 1
```

HMO groups + Secretor

```
#select HMO data  
meta3kk_HMO_groups <- dplyr::select(meta3kk_Excl, 
                       c("sample_ID","Secretor", "SUM_nmol_scale", "Sia_scale","Fuc_scale","Diversity"))


m0 <- vegan::capscale(D_relabundance ~ 1, data = meta3kk_HMO_groups[2:5])  
m1 <- vegan::capscale(D_relabundance ~ ., data = meta3kk_HMO_groups[2:5])
set.seed(123)
m <- vegan::ordistep(m0, scope = formula(m1))
```

```
## 
## Start: D_relabundance ~ 1 
## 
##                  Df    AIC      F Pr(>F)  
## + Secretor        1 1245.8 1.4404  0.055 .
## + Sia_scale       1 1246.1 1.0718  0.315  
## + Fuc_scale       1 1246.2 0.9837  0.455  
## + SUM_nmol_scale  1 1246.2 0.9874  0.490  
## ---
## Signif. codes:  0 '***' 0.001 '**' 0.01 '*' 0.05 '.' 0.1 ' ' 1
```

Test significance of the HMOs from ordistep:

```
#tse_subset_PERMANOVA <- transformAssay(tse_subset_PERMANOVA, assay.type = "pseudo", method = "relabundance")

method <- "relabundance"
diss_method <- "bray"

set.seed(123)

permanova <- adonis2(t(assay(tse_subset_PERMANOVA, method)) ~ DSLNT_nmol_scale + BMI_aiti + AIEMMATSYNNYTYKSET_cat +  SYNNYTYSTAPA_2luok ,
                     by = "margin",
                     data = colData(tse_subset_PERMANOVA),
                     method = diss_method,
                     permutations = 999)
```

Subset secretor status

```
tse_subset_03kk_secretor <- tse_subset_03kk_BF_Exclus[ , tse_subset_03kk_BF_Exclus$Secretor %in% c("1")] 


meta3kk_s <- as.data.frame(colData(tse_subset_03kk_secretor))

data3kk <- dplyr::select(meta3kk_s, 
                       c("nro", "LNFP_III_nmol",  "BMI_aiti", "AIEMMATSYNNYTYKSET_cat", "SYNNYTYSTAPA_2luok"))

data3kk1 <- na.omit(data3kk)

tse_subset_PERMANOVA_s<- tse_subset_03kk_secretor[ , tse_subset_03kk_secretor$nro %in% c(data3kk1$nro)]

meta3kk_Excl <- as.data.frame(colData(tse_subset_PERMANOVA_s))
```

```
#Add relabundance assay

tse_subset_PERMANOVA_s <- transformAssay(tse_subset_PERMANOVA_s, assay.type = "counts", method = "relabundance")
```

OORDISTEP USING DIST MATRIX

```
set.seed(123)

D_relabundance <- vegdist(t(assays(tse_subset_PERMANOVA_s)$relabundance), method="bray")
```

Independent HMOs subset secretors

```
#select HMO data  
meta3kk_HMO <- dplyr::select(meta3kk_Excl, 
                       c("sample_ID","X_2FL_nmol_scale", "X_3FL_nmol_scale", "LNnT_nmol_scale", "X_3SL_nmol_scale", "DFLac_nmol_scale", 
                 "X_6SL_nmol_scale", "LNT_nmol_scale", "LNFP_I_nmol_scale", "LNFP_II_nmol_scale", "LNFP_III_nmol_scale", 
                 "LSTb_nmol_scale", "LSTc_nmol_scale", "DFLNT_nmol_scale", "LNH_nmol_scale", "DSLNT_nmol_scale", 
                 "FLNH_nmol_scale", "DFLNH_nmol_scale", "FDSLNH_nmol_scale", "DSLNH_nmol_scale"))


m0 <- vegan::capscale(D_relabundance ~ 1, data = meta3kk_HMO[2:20])  
m1 <- vegan::capscale(D_relabundance ~ ., data = meta3kk_HMO[2:20])
set.seed(123)
m <- vegan::ordistep(m0, scope = formula(m1), direction = "both")
```

```
## 
## Start: D_relabundance ~ 1 
## 
##                       Df    AIC      F Pr(>F)  
## + DSLNT_nmol_scale     1 1099.5 1.6296  0.065 .
## + X_2FL_nmol_scale     1 1099.6 1.4531  0.075 .
## + FLNH_nmol_scale      1 1099.8 1.3323  0.130  
## + LSTc_nmol_scale      1 1099.9 1.2081  0.195  
## + DFLac_nmol_scale     1 1099.9 1.2278  0.210  
## + DFLNH_nmol_scale     1 1099.9 1.1885  0.235  
## + LNFP_II_nmol_scale   1 1099.9 1.1991  0.285  
## + LNH_nmol_scale       1 1100.0 1.0719  0.420  
## + DFLNT_nmol_scale     1 1100.1 0.9778  0.425  
## + X_3SL_nmol_scale     1 1100.1 0.9459  0.450  
## + LSTb_nmol_scale      1 1100.1 0.9826  0.470  
## + LNnT_nmol_scale      1 1100.1 0.9525  0.540  
## + LNFP_III_nmol_scale  1 1100.2 0.8993  0.580  
## + LNT_nmol_scale       1 1100.2 0.9022  0.590  
## + DSLNH_nmol_scale     1 1100.2 0.8479  0.685  
## + FDSLNH_nmol_scale    1 1100.3 0.7832  0.715  
## + LNFP_I_nmol_scale    1 1100.3 0.7749  0.770  
## + X_3FL_nmol_scale     1 1100.5 0.6198  0.945  
## + X_6SL_nmol_scale     1 1100.5 0.5348  0.990  
## ---
## Signif. codes:  0 '***' 0.001 '**' 0.01 '*' 0.05 '.' 0.1 ' ' 1
```

HMO groups subset secretors

```
#select HMO data  
meta3kk_HMO_groups <- dplyr::select(meta3kk_s, 
                       c("sample_ID", "SUM_nmol", "Sia","Fuc","Diversity"))


m0 <- vegan::capscale(D_relabundance ~ 1, data = meta3kk_HMO_groups[2:5])  
m1 <- vegan::capscale(D_relabundance ~ ., data = meta3kk_HMO_groups[2:5])
set.seed(123)
m <- vegan::ordistep(m0, scope = formula(m1))
```

```
## 
## Start: D_relabundance ~ 1 
## 
##             Df    AIC      F Pr(>F)  
## + Sia        1 1099.6 1.4442  0.080 .
## + Diversity  1 1099.7 1.4011  0.080 .
## + SUM_nmol   1 1099.8 1.3169  0.140  
## + Fuc        1 1099.9 1.1771  0.195  
## ---
## Signif. codes:  0 '***' 0.001 '**' 0.01 '*' 0.05 '.' 0.1 ' ' 1
```

Test significance of the HMOs from ordistep:

```
method <- "relabundance"
diss_method <- "bray"

set.seed(123)

permanova <- adonis2(t(assay(tse_subset_PERMANOVA_s, method)) ~  DSLNT_nmol_scale   + BMI_aiti + AIEMMATSYNNYTYKSET_cat +  SYNNYTYSTAPA_2luok  ,
                     by = "margin",
                     data = colData(tse_subset_PERMANOVA_s),
                     method = diss_method,
                     permutations = 999)
```

## Late infanfy 13 month

```
meta13kk <- as.data.frame(colData(tse_subset_by_13kk_ASV))

data13kk <- dplyr::select(meta13kk, 
                       c("nro", "LNnT_nmol", "BMI_aiti", "AIEMMATSYNNYTYKSET_cat", "SYNNYTYSTAPA_2luok"))

data13kk1 <- na.omit(data13kk)

tse_subset_PERMANOVA<- tse_subset_by_13kk_ASV[ , tse_subset_by_13kk_ASV$nro %in% c(data13kk1$nro)]

meta13kk_all <- as.data.frame(colData(tse_subset_PERMANOVA))
```

```
#Add relabundance assay

tse_subset_PERMANOVA <- transformAssay(tse_subset_PERMANOVA, assay.type = "counts", method = "relabundance")


method <- "relabundance"
diss_method <- "bray"

set.seed(123)

permanova <- adonis2(t(assay(tse_subset_PERMANOVA, method)) ~  Secretor + BMI_aiti+ AIEMMATSYNNYTYKSET_cat +  SYNNYTYSTAPA_2luok ,
                     by = "margin",
                     data = colData(tse_subset_PERMANOVA),
                     method = diss_method,
                     permutations = 999)

permanova
```

```
## Permutation test for adonis under reduced model
## Marginal effects of terms
## Permutation: free
## Number of permutations: 999
## 
## adonis2(formula = t(assay(tse_subset_PERMANOVA, method)) ~ Secretor + BMI_aiti + AIEMMATSYNNYTYKSET_cat + SYNNYTYSTAPA_2luok, data = colData(tse_subset_PERMANOVA), permutations = 999, method = diss_method, by = "margin")
##                         Df SumOfSqs      R2      F Pr(>F)    
## Secretor                 1    0.380 0.00219 1.1471  0.243    
## BMI_aiti                 1    0.354 0.00204 1.0697  0.333    
## AIEMMATSYNNYTYKSET_cat   1    1.487 0.00856 4.4889  0.001 ***
## SYNNYTYSTAPA_2luok       1    0.611 0.00351 1.8441  0.007 ** 
## Residual               516  170.964 0.98347                  
## Total                  520  173.838 1.00000                  
## ---
## Signif. codes:  0 '***' 0.001 '**' 0.01 '*' 0.05 '.' 0.1 ' ' 1
```

OORDISTEP USING DIST MATRIX

```
set.seed(123)
D_relabundance <- vegdist(t(assays(tse_subset_PERMANOVA)$relabundance), method="bray")
```

Independent HMOs 13 month

```
#select HMO data  
meta3kk_HMO <- dplyr::select(meta13kk_all, 
                       c("sample_ID","X_2FL_nmol_scale", "X_3FL_nmol_scale", "LNnT_nmol_scale", "X_3SL_nmol_scale", "DFLac_nmol_scale", 
                 "X_6SL_nmol_scale", "LNT_nmol_scale", "LNFP_I_nmol_scale", "LNFP_II_nmol_scale", "LNFP_III_nmol_scale", 
                 "LSTb_nmol_scale", "LSTc_nmol_scale", "DFLNT_nmol_scale", "LNH_nmol_scale", "DSLNT_nmol_scale", 
                 "FLNH_nmol_scale", "DFLNH_nmol_scale", "FDSLNH_nmol_scale", "DSLNH_nmol_scale"))

m0 <- vegan::capscale(D_relabundance ~ 1, data = meta3kk_HMO[2:20])  
m1 <- vegan::capscale(D_relabundance ~ ., data = meta3kk_HMO[2:20])
set.seed(123)
m <- vegan::ordistep(m0, scope = formula(m1), direction = "both")
```

```
## 
## Start: D_relabundance ~ 1 
## 
##                       Df    AIC      F Pr(>F)  
## + FDSLNH_nmol_scale    1 2777.5 1.4600  0.030 *
## + X_3FL_nmol_scale     1 2777.5 1.4529  0.030 *
## + X_2FL_nmol_scale     1 2777.5 1.4275  0.045 *
## + LNFP_II_nmol_scale   1 2777.5 1.3936  0.045 *
## + LNnT_nmol_scale      1 2777.5 1.4610  0.050 *
## + LNFP_I_nmol_scale    1 2777.5 1.4037  0.055 .
## + DSLNT_nmol_scale     1 2777.6 1.2988  0.090 .
## + LNH_nmol_scale       1 2777.7 1.2725  0.120  
## + LSTb_nmol_scale      1 2777.7 1.2220  0.145  
## + LNFP_III_nmol_scale  1 2777.8 1.1238  0.275  
## + X_6SL_nmol_scale     1 2777.9 1.0586  0.305  
## + X_3SL_nmol_scale     1 2777.9 1.0460  0.325  
## + LNT_nmol_scale       1 2777.9 1.0296  0.405  
## + LSTc_nmol_scale      1 2777.9 1.0110  0.425  
## + FLNH_nmol_scale      1 2777.9 0.9976  0.445  
## + DFLac_nmol_scale     1 2777.9 0.9849  0.555  
## + DFLNH_nmol_scale     1 2778.1 0.8927  0.635  
## + DFLNT_nmol_scale     1 2778.1 0.8639  0.715  
## + DSLNH_nmol_scale     1 2778.1 0.8777  0.725  
## ---
## Signif. codes:  0 '***' 0.001 '**' 0.01 '*' 0.05 '.' 0.1 ' ' 1
## 
## Step: D_relabundance ~ FDSLNH_nmol_scale 
## 
##                     Df    AIC    F Pr(>F)  
## - FDSLNH_nmol_scale  1 2776.9 1.46  0.025 *
## ---
## Signif. codes:  0 '***' 0.001 '**' 0.01 '*' 0.05 '.' 0.1 ' ' 1
## 
##                       Df    AIC      F Pr(>F)  
## + LNnT_nmol_scale      1 2778.0 1.5127  0.050 *
## + X_3FL_nmol_scale     1 2778.2 1.3088  0.050 *
## + DSLNT_nmol_scale     1 2778.2 1.2956  0.105  
## + LSTb_nmol_scale      1 2778.2 1.2227  0.125  
## + X_2FL_nmol_scale     1 2778.3 1.1789  0.155  
## + LNT_nmol_scale       1 2778.4 1.1050  0.340  
## + LNFP_III_nmol_scale  1 2778.4 1.0497  0.355  
## + DSLNH_nmol_scale     1 2778.4 1.0316  0.370  
## + X_3SL_nmol_scale     1 2778.4 1.0248  0.420  
## + FLNH_nmol_scale      1 2778.5 0.9955  0.440  
## + DFLNH_nmol_scale     1 2778.5 1.0051  0.445  
## + LNH_nmol_scale       1 2778.5 1.0059  0.450  
## + X_6SL_nmol_scale     1 2778.5 0.9952  0.460  
## + LSTc_nmol_scale      1 2778.5 0.9848  0.490  
## + LNFP_I_nmol_scale    1 2778.6 0.9095  0.640  
## + DFLac_nmol_scale     1 2778.6 0.8699  0.740  
## + DFLNT_nmol_scale     1 2778.6 0.8776  0.755  
## + LNFP_II_nmol_scale   1 2778.7 0.8200  0.790  
## ---
## Signif. codes:  0 '***' 0.001 '**' 0.01 '*' 0.05 '.' 0.1 ' ' 1
## 
## Step: D_relabundance ~ FDSLNH_nmol_scale + LNnT_nmol_scale 
## 
##                     Df    AIC      F Pr(>F)  
## - LNnT_nmol_scale    1 2777.5 1.5127  0.035 *
## - FDSLNH_nmol_scale  1 2777.5 1.5116  0.015 *
## ---
## Signif. codes:  0 '***' 0.001 '**' 0.01 '*' 0.05 '.' 0.1 ' ' 1
## 
##                       Df    AIC      F Pr(>F)
## + DSLNT_nmol_scale     1 2778.8 1.1156  0.250
## + LNFP_III_nmol_scale  1 2778.9 1.0538  0.305
## + X_3FL_nmol_scale     1 2778.9 1.0336  0.355
## + LSTb_nmol_scale      1 2778.9 1.0336  0.400
## + LNH_nmol_scale       1 2778.9 1.0073  0.420
## + FLNH_nmol_scale      1 2778.9 1.0008  0.435
## + LSTc_nmol_scale      1 2779.0 0.9826  0.475
## + DFLNH_nmol_scale     1 2779.0 0.9598  0.505
## + X_2FL_nmol_scale     1 2779.0 0.9271  0.585
## + DSLNH_nmol_scale     1 2779.0 0.9100  0.620
## + LNT_nmol_scale       1 2779.0 0.9084  0.665
## + LNFP_I_nmol_scale    1 2779.1 0.8808  0.685
## + DFLNT_nmol_scale     1 2779.1 0.8999  0.690
## + X_3SL_nmol_scale     1 2779.1 0.8181  0.825
## + LNFP_II_nmol_scale   1 2779.1 0.8127  0.840
## + DFLac_nmol_scale     1 2779.2 0.7494  0.930
## + X_6SL_nmol_scale     1 2779.2 0.7892  0.960
```

HMO groups 13 month

```
#select HMO data  
meta13kk_HMO_groups <- dplyr::select(meta13kk_all, 
                       c("sample_ID" ,"SUM_nmol_scale", "Sia_scale","Fuc_scale","Diversity"))


m0 <- vegan::capscale(D_relabundance ~ 1, data = meta13kk_HMO_groups[2:5])  
m1 <- vegan::capscale(D_relabundance ~ ., data = meta13kk_HMO_groups[2:5])
set.seed(123)
m <- vegan::ordistep(m0, scope = formula(m1))
```

```
## 
## Start: D_relabundance ~ 1 
## 
##                  Df    AIC      F Pr(>F)  
## + Fuc_scale       1 2777.5 1.4211  0.030 *
## + SUM_nmol_scale  1 2777.6 1.3139  0.085 .
## + Diversity       1 2777.7 1.2603  0.090 .
## + Sia_scale       1 2777.8 1.1410  0.185  
## ---
## Signif. codes:  0 '***' 0.001 '**' 0.01 '*' 0.05 '.' 0.1 ' ' 1
## 
## Step: D_relabundance ~ Fuc_scale 
## 
##             Df    AIC      F Pr(>F)  
## - Fuc_scale  1 2776.9 1.4211   0.03 *
## ---
## Signif. codes:  0 '***' 0.001 '**' 0.01 '*' 0.05 '.' 0.1 ' ' 1
## 
##                  Df    AIC      F Pr(>F)
## + SUM_nmol_scale  1 2778.3 1.1922  0.145
## + Diversity       1 2778.6 0.8723  0.745
## + Sia_scale       1 2778.7 0.7846  0.890
```

Test significance of the HMOs from ordistep:

```
method <- "relabundance"
diss_method <- "bray"

set.seed(123)

permanova <- adonis2(t(assay(tse_subset_PERMANOVA, method)) ~  Fuc_scale + BMI_aiti+ AIEMMATSYNNYTYKSET_cat +  SYNNYTYSTAPA_2luok ,
                     by = "margin",
                     data = colData(tse_subset_PERMANOVA),
                     method = diss_method,
                     permutations = 999)
permanova
```

```
## Permutation test for adonis under reduced model
## Marginal effects of terms
## Permutation: free
## Number of permutations: 999
## 
## adonis2(formula = t(assay(tse_subset_PERMANOVA, method)) ~ Fuc_scale + BMI_aiti + AIEMMATSYNNYTYKSET_cat + SYNNYTYSTAPA_2luok, data = colData(tse_subset_PERMANOVA), permutations = 999, method = diss_method, by = "margin")
##                         Df SumOfSqs      R2      F Pr(>F)    
## Fuc_scale                1    0.524 0.00301 1.5830  0.019 *  
## BMI_aiti                 1    0.356 0.00205 1.0740  0.325    
## AIEMMATSYNNYTYKSET_cat   1    1.484 0.00853 4.4815  0.001 ***
## SYNNYTYSTAPA_2luok       1    0.613 0.00353 1.8529  0.006 ** 
## Residual               516  170.820 0.98264                  
## Total                  520  173.838 1.00000                  
## ---
## Signif. codes:  0 '***' 0.001 '**' 0.01 '*' 0.05 '.' 0.1 ' ' 1
```

Subset secretor status

```
#subset secretor

tse_subset_13kk_secretor <- tse_subset_by_13kk_ASV[ , tse_subset_by_13kk_ASV$Secretor %in% c("1")] 

tse_subset_13kk_nonsecretor <- tse_subset_by_13kk_ASV[ , tse_subset_by_13kk_ASV$Secretor %in% c("0")]
```

Secretors

```
meta13kk_s <- as.data.frame(colData(tse_subset_13kk_secretor))

data13kk <- dplyr::select(meta13kk_s, 
                       c("nro", "LNFP_III_nmol", "BMI_aiti", "AIEMMATSYNNYTYKSET_cat", "SYNNYTYSTAPA_2luok"))

data13kk1 <- na.omit(data13kk)

tse_subset_PERMANOVA_s<- tse_subset_13kk_secretor[ , tse_subset_13kk_secretor$nro %in% c(data13kk1$nro)]

meta13kk_s <- as.data.frame(colData(tse_subset_PERMANOVA_s))

#Add relabundance assay

tse_subset_PERMANOVA_s <- transformAssay(tse_subset_PERMANOVA_s, assay.type = "counts", method = "relabundance")
```

OORDISTEP USING DIST MATRIX

```
set.seed(123)
D_relabundance <- vegdist(t(assays(tse_subset_PERMANOVA_s)$relabundance), method="bray")
```

Independent HMOs subset secretors 13 months

```
#select HMO data  
meta13kk_HMO <- dplyr::select(meta13kk_s, 
                       c("sample_ID","X_2FL_nmol_scale", "X_3FL_nmol_scale", "LNnT_nmol_scale", "X_3SL_nmol_scale", "DFLac_nmol_scale", 
                 "X_6SL_nmol_scale", "LNT_nmol_scale", "LNFP_I_nmol_scale", "LNFP_II_nmol_scale", "LNFP_III_nmol_scale", 
                 "LSTb_nmol_scale", "LSTc_nmol_scale", "DFLNT_nmol_scale", "LNH_nmol_scale", "DSLNT_nmol_scale", 
                 "FLNH_nmol_scale", "DFLNH_nmol_scale", "FDSLNH_nmol_scale", "DSLNH_nmol_scale"))


m0 <- vegan::capscale(D_relabundance ~ 1, data = meta13kk_HMO[2:20])  
m1 <- vegan::capscale(D_relabundance ~ ., data = meta13kk_HMO[2:20])
set.seed(123)
m <- vegan::ordistep(m0, scope = formula(m1), direction = "both")
```

```
## 
## Start: D_relabundance ~ 1 
## 
##                       Df    AIC      F Pr(>F)  
## + LNFP_III_nmol_scale  1 2416.7 1.4047  0.040 *
## + DFLNH_nmol_scale     1 2416.7 1.4004  0.045 *
## + LNH_nmol_scale       1 2416.7 1.3548  0.050 *
## + LNFP_II_nmol_scale   1 2416.7 1.3993  0.070 .
## + X_3FL_nmol_scale     1 2416.7 1.3161  0.075 .
## + DSLNT_nmol_scale     1 2416.7 1.3866  0.080 .
## + LNFP_I_nmol_scale    1 2416.8 1.2986  0.080 .
## + LSTb_nmol_scale      1 2416.8 1.2752  0.080 .
## + LNnT_nmol_scale      1 2416.8 1.2732  0.105  
## + FDSLNH_nmol_scale    1 2416.8 1.2078  0.110  
## + X_2FL_nmol_scale     1 2416.9 1.1855  0.135  
## + LSTc_nmol_scale      1 2416.9 1.1181  0.285  
## + X_6SL_nmol_scale     1 2417.0 1.0675  0.365  
## + DSLNH_nmol_scale     1 2417.1 1.0049  0.365  
## + FLNH_nmol_scale      1 2417.1 0.9558  0.540  
## + DFLNT_nmol_scale     1 2417.1 0.9531  0.555  
## + X_3SL_nmol_scale     1 2417.1 0.9494  0.565  
## + LNT_nmol_scale       1 2417.2 0.8941  0.625  
## + DFLac_nmol_scale     1 2417.2 0.8832  0.665  
## ---
## Signif. codes:  0 '***' 0.001 '**' 0.01 '*' 0.05 '.' 0.1 ' ' 1
## 
## Step: D_relabundance ~ LNFP_III_nmol_scale 
## 
##                       Df    AIC      F Pr(>F)  
## - LNFP_III_nmol_scale  1 2416.1 1.4047   0.03 *
## ---
## Signif. codes:  0 '***' 0.001 '**' 0.01 '*' 0.05 '.' 0.1 ' ' 1
## 
##                      Df    AIC      F Pr(>F)  
## + DSLNT_nmol_scale    1 2417.3 1.3726  0.035 *
## + DFLNH_nmol_scale    1 2417.2 1.4771  0.045 *
## + X_3FL_nmol_scale    1 2417.3 1.3240  0.090 .
## + LSTb_nmol_scale     1 2417.4 1.2833  0.090 .
## + LNnT_nmol_scale     1 2417.4 1.2678  0.105  
## + LNFP_I_nmol_scale   1 2417.4 1.2802  0.110  
## + LNFP_II_nmol_scale  1 2417.5 1.1926  0.170  
## + X_2FL_nmol_scale    1 2417.5 1.1125  0.235  
## + FDSLNH_nmol_scale   1 2417.5 1.1196  0.265  
## + LNH_nmol_scale      1 2417.6 1.0704  0.405  
## + LSTc_nmol_scale     1 2417.6 1.0083  0.405  
## + X_6SL_nmol_scale    1 2417.6 1.0331  0.420  
## + DSLNH_nmol_scale    1 2417.7 0.9957  0.450  
## + DFLNT_nmol_scale    1 2417.7 0.9462  0.585  
## + X_3SL_nmol_scale    1 2417.7 0.9384  0.660  
## + FLNH_nmol_scale     1 2417.8 0.8806  0.690  
## + DFLac_nmol_scale    1 2417.8 0.8662  0.715  
## + LNT_nmol_scale      1 2417.8 0.8384  0.740  
## ---
## Signif. codes:  0 '***' 0.001 '**' 0.01 '*' 0.05 '.' 0.1 ' ' 1
## 
## Step: D_relabundance ~ LNFP_III_nmol_scale + DSLNT_nmol_scale 
## 
##                       Df    AIC      F Pr(>F)  
## - DSLNT_nmol_scale     1 2416.7 1.3726  0.050 *
## - LNFP_III_nmol_scale  1 2416.7 1.3906  0.035 *
## ---
## Signif. codes:  0 '***' 0.001 '**' 0.01 '*' 0.05 '.' 0.1 ' ' 1
## 
##                      Df    AIC      F Pr(>F)  
## + DFLNH_nmol_scale    1 2417.8 1.5197  0.020 *
## + LNFP_II_nmol_scale  1 2418.0 1.2363  0.140  
## + X_3FL_nmol_scale    1 2418.1 1.1935  0.140  
## + LNFP_I_nmol_scale   1 2418.0 1.2286  0.145  
## + X_3SL_nmol_scale    1 2418.2 1.0594  0.255  
## + FDSLNH_nmol_scale   1 2418.2 1.0968  0.275  
## + DFLac_nmol_scale    1 2418.2 1.0447  0.330  
## + LNnT_nmol_scale     1 2418.2 1.0508  0.365  
## + LNH_nmol_scale      1 2418.2 1.0549  0.400  
## + LSTc_nmol_scale     1 2418.2 1.0435  0.410  
## + DFLNT_nmol_scale    1 2418.3 0.9788  0.515  
## + DSLNH_nmol_scale    1 2418.3 0.9527  0.535  
## + X_6SL_nmol_scale    1 2418.3 0.9193  0.620  
## + FLNH_nmol_scale     1 2418.4 0.8826  0.680  
## + X_2FL_nmol_scale    1 2418.4 0.8619  0.800  
## + LNT_nmol_scale      1 2418.5 0.7693  0.905  
## + LSTb_nmol_scale     1 2418.6 0.6517  1.000  
## ---
## Signif. codes:  0 '***' 0.001 '**' 0.01 '*' 0.05 '.' 0.1 ' ' 1
## 
## Step: D_relabundance ~ LNFP_III_nmol_scale + DSLNT_nmol_scale + DFLNH_nmol_scale 
## 
##                       Df    AIC      F Pr(>F)  
## - DSLNT_nmol_scale     1 2417.2 1.4155  0.055 .
## - LNFP_III_nmol_scale  1 2417.2 1.4744  0.025 *
## - DFLNH_nmol_scale     1 2417.3 1.5197  0.025 *
## ---
## Signif. codes:  0 '***' 0.001 '**' 0.01 '*' 0.05 '.' 0.1 ' ' 1
## 
##                      Df    AIC      F Pr(>F)   
## + LNFP_I_nmol_scale   1 2418.2 1.4893  0.005 **
## + LNFP_II_nmol_scale  1 2418.4 1.3226  0.095 . 
## + FDSLNH_nmol_scale   1 2418.5 1.2627  0.095 . 
## + LSTc_nmol_scale     1 2418.5 1.2055  0.195   
## + X_3FL_nmol_scale    1 2418.7 1.0688  0.325   
## + LNH_nmol_scale      1 2418.7 1.0246  0.405   
## + DFLNT_nmol_scale    1 2418.7 1.0323  0.415   
## + DSLNH_nmol_scale    1 2418.8 0.9166  0.605   
## + DFLac_nmol_scale    1 2418.8 0.9333  0.620   
## + X_6SL_nmol_scale    1 2418.8 0.9200  0.635   
## + FLNH_nmol_scale     1 2418.9 0.8793  0.680   
## + LNnT_nmol_scale     1 2418.9 0.8486  0.725   
## + X_3SL_nmol_scale    1 2418.9 0.8276  0.830   
## + X_2FL_nmol_scale    1 2418.9 0.7885  0.855   
## + LNT_nmol_scale      1 2419.0 0.7395  0.925   
## + LSTb_nmol_scale     1 2419.1 0.6702  0.995   
## ---
## Signif. codes:  0 '***' 0.001 '**' 0.01 '*' 0.05 '.' 0.1 ' ' 1
## 
## Step: D_relabundance ~ LNFP_III_nmol_scale + DSLNT_nmol_scale + DFLNH_nmol_scale +      LNFP_I_nmol_scale 
## 
##                       Df    AIC      F Pr(>F)   
## - LNFP_III_nmol_scale  1 2417.7 1.4209  0.055 . 
## - DSLNT_nmol_scale     1 2417.6 1.3704  0.045 * 
## - LNFP_I_nmol_scale    1 2417.8 1.4893  0.025 * 
## - DFLNH_nmol_scale     1 2418.0 1.7799  0.005 **
## ---
## Signif. codes:  0 '***' 0.001 '**' 0.01 '*' 0.05 '.' 0.1 ' ' 1
## 
##                      Df    AIC      F Pr(>F)  
## + LSTc_nmol_scale     1 2418.9 1.2982  0.085 .
## + X_3FL_nmol_scale    1 2419.2 1.0759  0.305  
## + DFLac_nmol_scale    1 2419.2 1.0375  0.365  
## + X_3SL_nmol_scale    1 2419.2 1.0485  0.385  
## + DSLNH_nmol_scale    1 2419.2 1.0248  0.385  
## + X_6SL_nmol_scale    1 2419.3 0.9087  0.600  
## + LNH_nmol_scale      1 2419.3 0.9158  0.610  
## + LNFP_II_nmol_scale  1 2419.3 0.9325  0.615  
## + FDSLNH_nmol_scale   1 2419.3 0.9034  0.650  
## + FLNH_nmol_scale     1 2419.3 0.8832  0.685  
## + LNnT_nmol_scale     1 2419.4 0.8480  0.790  
## + LNT_nmol_scale      1 2419.5 0.7616  0.885  
## + DFLNT_nmol_scale    1 2419.5 0.7347  0.925  
## + X_2FL_nmol_scale    1 2419.5 0.7009  0.965  
## + LSTb_nmol_scale     1 2419.6 0.6130  0.990  
## ---
## Signif. codes:  0 '***' 0.001 '**' 0.01 '*' 0.05 '.' 0.1 ' ' 1
```

HMO groups subset secretors 13 months

```
#select HMO data  
meta13kk_HMO_groups <- dplyr::select(meta13kk_s, 
                       c("sample_ID", "SUM_nmol", "Sia","Fuc","Diversity"))


m0 <- vegan::capscale(D_relabundance ~ 1, data = meta13kk_HMO_groups[2:5])  
m1 <- vegan::capscale(D_relabundance ~ ., data = meta13kk_HMO_groups[2:5])
set.seed(123)
m <- vegan::ordistep(m0, scope = formula(m1))
```

```
## 
## Start: D_relabundance ~ 1 
## 
##             Df    AIC      F Pr(>F)  
## + Fuc        1 2416.7 1.3567  0.090 .
## + SUM_nmol   1 2416.9 1.1524  0.185  
## + Diversity  1 2417.0 1.0573  0.325  
## + Sia        1 2417.1 0.9771  0.520  
## ---
## Signif. codes:  0 '***' 0.001 '**' 0.01 '*' 0.05 '.' 0.1 ' ' 1
```

Test significance of the HMOs from ordistep:

```
method <- "relabundance"
diss_method <- "bray"

set.seed(123)

permanova <- adonis2(t(assay(tse_subset_PERMANOVA_s, method)) ~ LNFP_III_nmol_scale + BMI_aiti + AIEMMATSYNNYTYKSET_cat +  SYNNYTYSTAPA_2luok ,
                     by = "margin",
                     data = colData(tse_subset_PERMANOVA_s),
                     method = diss_method,
                     permutations = 999)
```

# Alpha diversity

Add diversity estimates to this data set

```
tse_subset_by_03kk_ASV <- mia::addAlpha(tse_subset_by_03kk_ASV, 
                             assay_name = "counts", 
                             index = "observed", 
                             name="observed_richness")


tse_subset_by_03kk_ASV <- mia::addAlpha(tse_subset_by_03kk_ASV, 
                              assay.type = "counts", # Calculate diversity from "counts" assay 
                              index = "shannon",
                              name = "shannon_diversity")     # Use Shannon index


sample_data_3kk <- as.data.frame(colData(tse_subset_by_03kk_ASV))

###################13 mo
tse_subset_by_13kk_ASV <- mia::addAlpha(tse_subset_by_13kk_ASV, 
                             assay_name = "counts", 
                             index = "observed", 
                             name="observed_richness")


tse_subset_by_13kk_ASV <- mia::addAlpha(tse_subset_by_13kk_ASV, 
                              assay.type = "counts", # Calculate diversity from "counts" assay 
                              index = "shannon",
                              name = "shannon_diversity")     # Use Shannon index


sample_data_13kk <- as.data.frame(colData(tse_subset_by_13kk_ASV))
```

## 3 month

1. Shannon, 3 month

```
# Initialize a data frame to store results
results_df <- data.frame()

# Define the variables you want to test
hmo_variables <- c("X_2FL_nmol_scale", "X_3FL_nmol_scale", "LNnT_nmol_scale", "X_3SL_nmol_scale", "DFLac_nmol_scale", 
                 "X_6SL_nmol_scale", "LNT_nmol_scale", "LNFP_I_nmol_scale", "LNFP_II_nmol_scale", "LNFP_III_nmol_scale", 
                 "LSTb_nmol_scale", "LSTc_nmol_scale", "DFLNT_nmol_scale", "LNH_nmol_scale", "DSLNT_nmol_scale", 
                 "FLNH_nmol_scale", "DFLNH_nmol_scale", "FDSLNH_nmol_scale", "DSLNH_nmol_scale","Diversity","SUM_nmol_scale", "Sia_scale", "Fuc_scale", "Secretor") 

# Define the adjustment covariates
adjustment_vars <- "SYNNYTYSTAPA_2luok + AIEMMATSYNNYTYKSET_cat + BMI_aiti"

# Initialize a list to store diagnostics
assumption_diagnostics <- list()

# Loop through each HMO variable
for (var in hmo_variables) {
  # Build the formula dynamically to include the covariates
  formula <- as.formula(paste("shannon_diversity ~", var, "+", adjustment_vars))
  
  # Fit the glm model
  model <- glm(formula, data = sample_data_3kk)
  
  # Extract coefficients, standard errors, t-values, p-values, and confidence intervals
  res <- cbind(coefficients(summary(model)), confint(model))
  
  # Convert to a data frame
  res_df <- as.data.frame(res)[2,]
  
  # Add a column for the variable name
  res_df$Variable <- var
  
  # Append to the results data frame
  results_df <- rbind(results_df, res_df)
  
  # Run assumption tests (simulate residuals for model diagnostics)
  simulationOutput <- simulateResiduals(fittedModel = model)
  
  # Store the diagnostics results
  assumption_diagnostics[[var]] <- simulationOutput
}

# Add column names to results_df (if necessary)
colnames(results_df) <- c("Estimate", "Std.Error", "t.value", "Pr(>|t|)", "CI_2.5%", "CI_97.5%", "Variable")

# Extract p-values and apply Benjamini-Hochberg (BH) correction
results_df$BH_Adjusted_P <- p.adjust(results_df$`Pr(>|t|)`, method = "BH")

results_df_1 <- results_df

# Plot diagnostics for each HMO model
#for (var in hmo_variables) { plot(assumption_diagnostics[[var]], main = paste("Diagnostic Plots for", var))}
```

1. Observed richness, 3 month

```
# Initialize a data frame to store results
results_df_2 <- data.frame()

# Define the variables you want to test
hmo_variables <- c("X_2FL_nmol_scale", "X_3FL_nmol_scale", "LNnT_nmol_scale", "X_3SL_nmol_scale", "DFLac_nmol_scale", 
                 "X_6SL_nmol_scale", "LNT_nmol_scale", "LNFP_I_nmol_scale", "LNFP_II_nmol_scale", "LNFP_III_nmol_scale", 
                 "LSTb_nmol_scale", "LSTc_nmol_scale", "DFLNT_nmol_scale", "LNH_nmol_scale", "DSLNT_nmol_scale", 
                 "FLNH_nmol_scale", "DFLNH_nmol_scale", "FDSLNH_nmol_scale", "DSLNH_nmol_scale","Diversity","SUM_nmol_scale", "Sia_scale", "Fuc_scale", "Secretor") 

# Define the adjustment covariates
adjustment_vars <- "SYNNYTYSTAPA_2luok + AIEMMATSYNNYTYKSET_cat + BMI_aiti"

# Initialize a list to store diagnostics
assumption_diagnostics <- list()

# Loop through each HMO variable
for (var in hmo_variables) {
  # Build the formula dynamically to include the covariates
  formula <- as.formula(paste("observed_richness ~", var, "+", adjustment_vars))
  
  # Fit the glm model
  model <- glm(formula, data = sample_data_3kk)
  
  # Extract coefficients, standard errors, t-values, p-values, and confidence intervals
  res <- cbind(coefficients(summary(model)), confint(model))
  
  # Convert to a data frame
  res_df <- as.data.frame(res)[2,]
  
  # Add a column for the variable name
  res_df$Variable <- var
  
  # Append to the results data frame
  results_df_2 <- rbind(results_df_2, res_df)
  
  # Run assumption tests (simulate residuals for model diagnostics)
  simulationOutput <- simulateResiduals(fittedModel = model)
  
  # Store the diagnostics results
  assumption_diagnostics[[var]] <- simulationOutput
}

# Add column names to results_df (if necessary)
colnames(results_df_2) <- c("Estimate", "Std.Error", "t.value", "Pr(>|t|)", "CI_2.5%", "CI_97.5%", "Variable")

# Extract p-values and apply Benjamini-Hochberg (BH) correction
results_df_2$BH_Adjusted_P <- p.adjust(results_df_2$`Pr(>|t|)`, method = "BH")
```

Subset secretor and non secretors

```
tse_subset_by_03kk_ASV_secretor <- tse_subset_by_03kk_ASV[ , tse_subset_by_03kk_ASV$Secretor %in% c("1")] 

tse_subset_by_03kk_ASV_non_secretor <- tse_subset_by_03kk_ASV[ , tse_subset_by_03kk_ASV$Secretor %in% c("0")] 

#Add diversity estimates to this subsetted data set
tse_subset_by_03kk_ASV_secretor <- mia::addAlpha(tse_subset_by_03kk_ASV_secretor, 
                             assay_name = "counts", 
                             index = "observed", 
                             name="observed_richness")


tse_subset_by_03kk_ASV_secretor <- mia::addAlpha(tse_subset_by_03kk_ASV_secretor, 
                              assay.type = "counts", # Calculate diversity from "counts" assay 
                              index = "shannon",
                              name = "shannon_diversity")     # Use Shannon index

###################

tse_subset_by_03kk_ASV_non_secretor <- mia::addAlpha(tse_subset_by_03kk_ASV_non_secretor, 
                             assay_name = "counts", 
                             index = "observed", 
                             name="observed_richness")


tse_subset_by_03kk_ASV_non_secretor <- mia::addAlpha(tse_subset_by_03kk_ASV_non_secretor, 
                              assay.type = "counts", # Calculate diversity from "counts" assay 
                              index = "shannon",
                              name = "shannon_diversity")     # Use Shannon index


sample_data_3kk_se <- as.data.frame(colData(tse_subset_by_03kk_ASV_secretor))
sample_data_3kk_nse <- as.data.frame(colData(tse_subset_by_03kk_ASV_non_secretor))
```

2. Secretors, Shannon, 3 month

```
# Initialize a data frame to store results
results_df <- data.frame()

# Define the variables you want to test
hmo_variables <- c("X_2FL_nmol_scale", "X_3FL_nmol_scale", "LNnT_nmol_scale", "X_3SL_nmol_scale", "DFLac_nmol_scale", 
                 "X_6SL_nmol_scale", "LNT_nmol_scale", "LNFP_I_nmol_scale", "LNFP_II_nmol_scale", "LNFP_III_nmol_scale", 
                 "LSTb_nmol_scale", "LSTc_nmol_scale", "DFLNT_nmol_scale", "LNH_nmol_scale", "DSLNT_nmol_scale", 
                 "FLNH_nmol_scale", "DFLNH_nmol_scale", "FDSLNH_nmol_scale", "DSLNH_nmol_scale","Diversity","Evenness","SUM_nmol_scale", "Sia_scale", "Fuc_scale") 

# Define the adjustment covariates
adjustment_vars <- "SYNNYTYSTAPA_2luok + AIEMMATSYNNYTYKSET_cat + BMI_aiti"

# Initialize a list to store diagnostics
assumption_diagnostics <- list()

# Loop through each HMO variable
for (var in hmo_variables) {
  # Build the formula dynamically to include the covariates
  formula <- as.formula(paste("shannon_diversity ~", var, "+", adjustment_vars))
  
  # Fit the glm model
  model <- glm(formula, data = sample_data_3kk_se)
  
  # Extract coefficients, standard errors, t-values, p-values, and confidence intervals
  res <- cbind(coefficients(summary(model)), confint(model))
  
  # Convert to a data frame
  res_df <- as.data.frame(res)[2,]
  
  # Add a column for the variable name
  res_df$Variable <- var
  
  # Append to the results data frame
  results_df <- rbind(results_df, res_df)
  
  # Run assumption tests (simulate residuals for model diagnostics)
  simulationOutput <- simulateResiduals(fittedModel = model)
  
  # Store the diagnostics results
  assumption_diagnostics[[var]] <- simulationOutput
}

# Add column names to results_df (if necessary)
colnames(results_df) <- c("Estimate", "Std.Error", "t.value", "Pr(>|t|)", "CI_2.5%", "CI_97.5%", "Variable")

# Extract p-values and apply Benjamini-Hochberg (BH) correction
results_df$BH_Adjusted_P <- p.adjust(results_df$`Pr(>|t|)`, method = "BH")

results_df_3 <- results_df
```

2. Secretors, Observed richness, 3 month

```
# Initialize a data frame to store results
results_df <- data.frame()

# Define the variables you want to test
hmo_variables <- c("X_2FL_nmol_scale", "X_3FL_nmol_scale", "LNnT_nmol_scale", "X_3SL_nmol_scale", "DFLac_nmol_scale", 
                 "X_6SL_nmol_scale", "LNT_nmol_scale", "LNFP_I_nmol_scale", "LNFP_II_nmol_scale", "LNFP_III_nmol_scale", 
                 "LSTb_nmol_scale", "LSTc_nmol_scale", "DFLNT_nmol_scale", "LNH_nmol_scale", "DSLNT_nmol_scale", 
                 "FLNH_nmol_scale", "DFLNH_nmol_scale", "FDSLNH_nmol_scale", "DSLNH_nmol_scale","Diversity","Evenness","SUM_nmol_scale", "Sia_scale", "Fuc_scale") 

# Define the adjustment covariates
adjustment_vars <- "SYNNYTYSTAPA_2luok + AIEMMATSYNNYTYKSET_cat + BMI_aiti"

# Initialize a list to store diagnostics
assumption_diagnostics <- list()

# Loop through each HMO variable
for (var in hmo_variables) {
  # Build the formula dynamically to include the covariates
  formula <- as.formula(paste("observed_richness ~", var, "+", adjustment_vars))
  
  # Fit the glm model
  model <- glm(formula, data = sample_data_3kk_se)
  
  # Extract coefficients, standard errors, t-values, p-values, and confidence intervals
  res <- cbind(coefficients(summary(model)), confint(model))
  
  # Convert to a data frame
  res_df <- as.data.frame(res)[2,]
  
  # Add a column for the variable name
  res_df$Variable <- var
  
  # Append to the results data frame
  results_df <- rbind(results_df, res_df)
  
  # Run assumption tests (simulate residuals for model diagnostics)
  simulationOutput <- simulateResiduals(fittedModel = model)
  
  # Store the diagnostics results
  assumption_diagnostics[[var]] <- simulationOutput
}

# Add column names to results_df (if necessary)
colnames(results_df) <- c("Estimate", "Std.Error", "t.value", "Pr(>|t|)", "CI_2.5%", "CI_97.5%", "Variable")

# Extract p-values and apply Benjamini-Hochberg (BH) correction
results_df$BH_Adjusted_P <- p.adjust(results_df$`Pr(>|t|)`, method = "BH")

results_df_4 <- results_df
```

3. Non-secretors, Shannon, 3 month

```
# Initialize a data frame to store results
results_df <- data.frame()

# Define the variables you want to test
hmo_variables <- c("X_2FL_nmol_scale", "X_3FL_nmol_scale", "LNnT_nmol_scale", "X_3SL_nmol_scale", "DFLac_nmol_scale", 
                 "X_6SL_nmol_scale", "LNT_nmol_scale", "LNFP_I_nmol_scale", "LNFP_II_nmol_scale", "LNFP_III_nmol_scale", 
                 "LSTb_nmol_scale", "LSTc_nmol_scale", "DFLNT_nmol_scale", "LNH_nmol_scale", "DSLNT_nmol_scale", 
                 "FLNH_nmol_scale", "DFLNH_nmol_scale", "FDSLNH_nmol_scale", "DSLNH_nmol_scale","Diversity","Evenness","SUM_nmol_scale", "Sia_scale", "Fuc_scale") 

# Define the adjustment covariates
adjustment_vars <- "SYNNYTYSTAPA_2luok + AIEMMATSYNNYTYKSET_cat + BMI_aiti"

# Initialize a list to store diagnostics
assumption_diagnostics <- list()

# Loop through each HMO variable
for (var in hmo_variables) {
  # Build the formula dynamically to include the covariates
  formula <- as.formula(paste("shannon_diversity ~", var, "+", adjustment_vars))
  
  # Fit the glm model
  model <- glm(formula, data = sample_data_3kk_nse)
  
  # Extract coefficients, standard errors, t-values, p-values, and confidence intervals
  res <- cbind(coefficients(summary(model)), confint(model))
  
  # Convert to a data frame
  res_df <- as.data.frame(res)[2,]
  
  # Add a column for the variable name
  res_df$Variable <- var
  
  # Append to the results data frame
  results_df <- rbind(results_df, res_df)
  
  # Run assumption tests (simulate residuals for model diagnostics)
  simulationOutput <- simulateResiduals(fittedModel = model)
  
  # Store the diagnostics results
  assumption_diagnostics[[var]] <- simulationOutput
}

# Add column names to results_df (if necessary)
colnames(results_df) <- c("Estimate", "Std.Error", "t.value", "Pr(>|t|)", "CI_2.5%", "CI_97.5%", "Variable")

# Extract p-values and apply Benjamini-Hochberg (BH) correction
results_df$BH_Adjusted_P <- p.adjust(results_df$`Pr(>|t|)`, method = "BH")

results_df_5 <- results_df
```

3. Non-secretors, Observed richness, 3 month

```
# Initialize a data frame to store results
results_df <- data.frame()

# Define the variables you want to test
hmo_variables <- c("X_2FL_nmol_scale", "X_3FL_nmol_scale", "LNnT_nmol_scale", "X_3SL_nmol_scale", "DFLac_nmol_scale", 
                 "X_6SL_nmol_scale", "LNT_nmol_scale", "LNFP_I_nmol_scale", "LNFP_II_nmol_scale", "LNFP_III_nmol_scale", 
                 "LSTb_nmol_scale", "LSTc_nmol_scale", "DFLNT_nmol_scale", "LNH_nmol_scale", "DSLNT_nmol_scale", 
                 "FLNH_nmol_scale", "DFLNH_nmol_scale", "FDSLNH_nmol_scale", "DSLNH_nmol_scale","Diversity","Evenness","SUM_nmol_scale", "Sia_scale", "Fuc_scale") 

# Define the adjustment covariates
adjustment_vars <- "SYNNYTYSTAPA_2luok + AIEMMATSYNNYTYKSET_cat + BMI_aiti"

# Initialize a list to store diagnostics
assumption_diagnostics <- list()

# Loop through each HMO variable
for (var in hmo_variables) {
  # Build the formula dynamically to include the covariates
  formula <- as.formula(paste("observed_richness ~", var, "+", adjustment_vars))
  
  # Fit the glm model
  model <- glm(formula, data = sample_data_3kk_nse)
  
  # Extract coefficients, standard errors, t-values, p-values, and confidence intervals
  res <- cbind(coefficients(summary(model)), confint(model))
  
  # Convert to a data frame
  res_df <- as.data.frame(res)[2,]
  
  # Add a column for the variable name
  res_df$Variable <- var
  
  # Append to the results data frame
  results_df <- rbind(results_df, res_df)
  
  # Run assumption tests (simulate residuals for model diagnostics)
  simulationOutput <- simulateResiduals(fittedModel = model)
  
  # Store the diagnostics results
  assumption_diagnostics[[var]] <- simulationOutput
}

# Add column names to results_df (if necessary)
colnames(results_df) <- c("Estimate", "Std.Error", "t.value", "Pr(>|t|)", "CI_2.5%", "CI_97.5%", "Variable")

# Extract p-values and apply Benjamini-Hochberg (BH) correction
results_df$BH_Adjusted_P <- p.adjust(results_df$`Pr(>|t|)`, method = "BH")

results_df_6 <- results_df
```

## Exclusive BF 3 month

Subset the data

```
tse_subset_03kk_ASV_BF_Exclus <- tse_subset_by_03kk_ASV[ , tse_subset_by_03kk_ASV$breastfeeding_3kk_category %in% c("exclusive")] 

tse_subset_03kk_ASV_BF_Exclus_s <- tse_subset_03kk_ASV_BF_Exclus[ , tse_subset_03kk_ASV_BF_Exclus$Secretor %in% c("1")]
```

Add diversity estimates to this substted data set

```
tse_subset_03kk_ASV_BF_Exclus <- mia::addAlpha(tse_subset_03kk_ASV_BF_Exclus, 
                             assay_name = "counts", 
                             index = "observed", 
                             name="observed_richness")


tse_subset_03kk_ASV_BF_Exclus <- mia::addAlpha(tse_subset_03kk_ASV_BF_Exclus, 
                              assay.type = "counts", # Calculate diversity from "counts" assay 
                              index = "shannon",
                              name = "shannon_diversity")     # Use Shannon index

#################
tse_subset_03kk_ASV_BF_Exclus_s <- mia::addAlpha(tse_subset_03kk_ASV_BF_Exclus_s, 
                             assay_name = "counts", 
                             index = "observed", 
                             name="observed_richness")


tse_subset_03kk_ASV_BF_Exclus_s <- mia::addAlpha(tse_subset_03kk_ASV_BF_Exclus_s, 
                              assay.type = "counts", # Calculate diversity from "counts" assay 
                              index = "shannon",
                              name = "shannon_diversity")     # Use Shannon index

sample_data_3kk_exc <- as.data.frame(colData(tse_subset_03kk_ASV_BF_Exclus))
sample_data_3kk_exc_se <- as.data.frame(colData(tse_subset_03kk_ASV_BF_Exclus_s))
```

1. Shannon, Exclusive BF 3 month

```
# Initialize a data frame to store results
results_df <- data.frame()

# Define the variables you want to test
hmo_variables <- c("X_2FL_nmol_scale", "X_3FL_nmol_scale", "LNnT_nmol_scale", "X_3SL_nmol_scale", "DFLac_nmol_scale", 
                 "X_6SL_nmol_scale", "LNT_nmol_scale", "LNFP_I_nmol_scale", "LNFP_II_nmol_scale", "LNFP_III_nmol_scale", 
                 "LSTb_nmol_scale", "LSTc_nmol_scale", "DFLNT_nmol_scale", "LNH_nmol_scale", "DSLNT_nmol_scale", 
                 "FLNH_nmol_scale", "DFLNH_nmol_scale", "FDSLNH_nmol_scale", "DSLNH_nmol_scale","Diversity","Evenness","SUM_nmol_scale", "Sia_scale", "Fuc_scale", "Secretor") 

# Define the adjustment covariates
adjustment_vars <- "SYNNYTYSTAPA_2luok + AIEMMATSYNNYTYKSET_cat + BMI_aiti"

# Initialize a list to store diagnostics
assumption_diagnostics <- list()

# Loop through each HMO variable
for (var in hmo_variables) {
  # Build the formula dynamically to include the covariates
  formula <- as.formula(paste("shannon_diversity ~", var, "+", adjustment_vars))
  
  # Fit the glm model
  model <- glm(formula, data = sample_data_3kk_exc)
  
  # Extract coefficients, standard errors, t-values, p-values, and confidence intervals
  res <- cbind(coefficients(summary(model)), confint(model))
  
  # Convert to a data frame
  res_df <- as.data.frame(res)[2,]
  
  # Add a column for the variable name
  res_df$Variable <- var
  
  # Append to the results data frame
  results_df <- rbind(results_df, res_df)
  
  # Run assumption tests (simulate residuals for model diagnostics)
  simulationOutput <- simulateResiduals(fittedModel = model)
  
  # Store the diagnostics results
  assumption_diagnostics[[var]] <- simulationOutput
}

# Add column names to results_df (if necessary)
colnames(results_df) <- c("Estimate", "Std.Error", "t.value", "Pr(>|t|)", "CI_2.5%", "CI_97.5%", "Variable")

# Extract p-values and apply Benjamini-Hochberg (BH) correction
results_df$BH_Adjusted_P <- p.adjust(results_df$`Pr(>|t|)`, method = "BH")

results_df_7 <- results_df
```

1. Observed richness, Exclusive BF 3 month

```
# Initialize a data frame to store results
results_df <- data.frame()

# Define the variables you want to test
hmo_variables <- c("X_2FL_nmol_scale", "X_3FL_nmol_scale", "LNnT_nmol_scale", "X_3SL_nmol_scale", "DFLac_nmol_scale", 
                 "X_6SL_nmol_scale", "LNT_nmol_scale", "LNFP_I_nmol_scale", "LNFP_II_nmol_scale", "LNFP_III_nmol_scale", 
                 "LSTb_nmol_scale", "LSTc_nmol_scale", "DFLNT_nmol_scale", "LNH_nmol_scale", "DSLNT_nmol_scale", 
                 "FLNH_nmol_scale", "DFLNH_nmol_scale", "FDSLNH_nmol_scale", "DSLNH_nmol_scale","Diversity","Evenness","SUM_nmol_scale", "Sia_scale", "Fuc_scale", "Secretor") 

# Define the adjustment covariates
adjustment_vars <- "SYNNYTYSTAPA_2luok + AIEMMATSYNNYTYKSET_cat + BMI_aiti"

# Initialize a list to store diagnostics
assumption_diagnostics <- list()

# Loop through each HMO variable
for (var in hmo_variables) {
  # Build the formula dynamically to include the covariates
  formula <- as.formula(paste("observed_richness ~", var, "+", adjustment_vars))
  
  # Fit the glm model
  model <- glm(formula, data = sample_data_3kk_exc)
  
  # Extract coefficients, standard errors, t-values, p-values, and confidence intervals
  res <- cbind(coefficients(summary(model)), confint(model))
  
  # Convert to a data frame
  res_df <- as.data.frame(res)[2,]
  
  # Add a column for the variable name
  res_df$Variable <- var
  
  # Append to the results data frame
  results_df <- rbind(results_df, res_df)
  
  # Run assumption tests (simulate residuals for model diagnostics)
  simulationOutput <- simulateResiduals(fittedModel = model)
  
  # Store the diagnostics results
  assumption_diagnostics[[var]] <- simulationOutput
}

# Add column names to results_df (if necessary)
colnames(results_df) <- c("Estimate", "Std.Error", "t.value", "Pr(>|t|)", "CI_2.5%", "CI_97.5%", "Variable")

# Extract p-values and apply Benjamini-Hochberg (BH) correction
results_df$BH_Adjusted_P <- p.adjust(results_df$`Pr(>|t|)`, method = "BH")

results_df_8 <- results_df
```

2. Secretors, Shannon, Exclusive BF 3 month

```
# Initialize a data frame to store results
results_df <- data.frame()

# Define the variables you want to test
hmo_variables <- c("X_2FL_nmol_scale", "X_3FL_nmol_scale", "LNnT_nmol_scale", "X_3SL_nmol_scale", "DFLac_nmol_scale", 
                 "X_6SL_nmol_scale", "LNT_nmol_scale", "LNFP_I_nmol_scale", "LNFP_II_nmol_scale", "LNFP_III_nmol_scale", 
                 "LSTb_nmol_scale", "LSTc_nmol_scale", "DFLNT_nmol_scale", "LNH_nmol_scale", "DSLNT_nmol_scale", 
                 "FLNH_nmol_scale", "DFLNH_nmol_scale", "FDSLNH_nmol_scale", "DSLNH_nmol_scale","Diversity","Evenness","SUM_nmol_scale", "Sia_scale", "Fuc_scale") 


# Define the adjustment covariates
adjustment_vars <- "SYNNYTYSTAPA_2luok + AIEMMATSYNNYTYKSET_cat + BMI_aiti"

# Initialize a list to store diagnostics
assumption_diagnostics <- list()

# Loop through each HMO variable
for (var in hmo_variables) {
  # Build the formula dynamically to include the covariates
  formula <- as.formula(paste("shannon_diversity ~", var, "+", adjustment_vars))
  
  # Fit the glm model
  model <- glm(formula, data = sample_data_3kk_exc_se)
  
  # Extract coefficients, standard errors, t-values, p-values, and confidence intervals
  res <- cbind(coefficients(summary(model)), confint(model))
  
  # Convert to a data frame
  res_df <- as.data.frame(res)[2,]
  
  # Add a column for the variable name
  res_df$Variable <- var
  
  # Append to the results data frame
  results_df <- rbind(results_df, res_df)
  
  # Run assumption tests (simulate residuals for model diagnostics)
  simulationOutput <- simulateResiduals(fittedModel = model)
  
  # Store the diagnostics results
  assumption_diagnostics[[var]] <- simulationOutput
}

# Add column names to results_df (if necessary)
colnames(results_df) <- c("Estimate", "Std.Error", "t.value", "Pr(>|t|)", "CI_2.5%", "CI_97.5%", "Variable")

# Extract p-values and apply Benjamini-Hochberg (BH) correction
results_df$BH_Adjusted_P <- p.adjust(results_df$`Pr(>|t|)`, method = "BH")


results_df_9 <- results_df
```

2. Secretors, Observed richness, Exclusive BF 3 month

```
# Initialize a data frame to store results
results_df <- data.frame()

# Define the variables you want to test
hmo_variables <- c("X_2FL_nmol_scale", "X_3FL_nmol_scale", "LNnT_nmol_scale", "X_3SL_nmol_scale", "DFLac_nmol_scale", 
                 "X_6SL_nmol_scale", "LNT_nmol_scale", "LNFP_I_nmol_scale", "LNFP_II_nmol_scale", "LNFP_III_nmol_scale", 
                 "LSTb_nmol_scale", "LSTc_nmol_scale", "DFLNT_nmol_scale", "LNH_nmol_scale", "DSLNT_nmol_scale", 
                 "FLNH_nmol_scale", "DFLNH_nmol_scale", "FDSLNH_nmol_scale", "DSLNH_nmol_scale","Diversity","Evenness","SUM_nmol_scale", "Sia_scale", "Fuc_scale") 

# Define the adjustment covariates
adjustment_vars <- "SYNNYTYSTAPA_2luok + AIEMMATSYNNYTYKSET_cat + BMI_aiti"

# Initialize a list to store diagnostics
assumption_diagnostics <- list()

# Loop through each HMO variable
for (var in hmo_variables) {
  # Build the formula dynamically to include the covariates
  formula <- as.formula(paste("observed_richness ~", var, "+", adjustment_vars))
  
  # Fit the glm model
  model <- glm(formula, data = sample_data_3kk_exc_se)
  
  # Extract coefficients, standard errors, t-values, p-values, and confidence intervals
  res <- cbind(coefficients(summary(model)), confint(model))
  
  # Convert to a data frame
  res_df <- as.data.frame(res)[2,]
  
  # Add a column for the variable name
  res_df$Variable <- var
  
  # Append to the results data frame
  results_df <- rbind(results_df, res_df)
  
  # Run assumption tests (simulate residuals for model diagnostics)
  simulationOutput <- simulateResiduals(fittedModel = model)
  
  # Store the diagnostics results
  assumption_diagnostics[[var]] <- simulationOutput
}

# Add column names to results_df (if necessary)
colnames(results_df) <- c("Estimate", "Std.Error", "t.value", "Pr(>|t|)", "CI_2.5%", "CI_97.5%", "Variable")

# Extract p-values and apply Benjamini-Hochberg (BH) correction
results_df$BH_Adjusted_P <- p.adjust(results_df$`Pr(>|t|)`, method = "BH")

results_df_10 <- results_df
```

## 13 months

Subset the data

```
tse_subset_by_13kk_ASV_se <- tse_subset_by_13kk_ASV[ , tse_subset_by_13kk_ASV$Secretor %in% c("1")] 

tse_subset_by_13kk_ASV_nse <- tse_subset_by_13kk_ASV[ , tse_subset_by_13kk_ASV$Secretor %in% c("0")]
```

Add diversity estimates to this substted data set

```
tse_subset_by_13kk_ASV <- mia::addAlpha(tse_subset_by_13kk_ASV, 
                             assay_name = "counts", 
                             index = "observed", 
                             name="observed_richness")


tse_subset_by_13kk_ASV <- mia::addAlpha(tse_subset_by_13kk_ASV, 
                              assay.type = "counts", # Calculate diversity from "counts" assay 
                              index = "shannon",
                              name = "shannon_diversity")     # Use Shannon index

sample_data_13mo <- as.data.frame(colData(tse_subset_by_13kk_ASV))

###########

tse_subset_by_13kk_ASV_se <- mia::addAlpha(tse_subset_by_13kk_ASV_se, 
                             assay_name = "counts", 
                             index = "observed", 
                             name="observed_richness")


tse_subset_by_13kk_ASV_se <- mia::addAlpha(tse_subset_by_13kk_ASV_se, 
                              assay.type = "counts", # Calculate diversity from "counts" assay 
                              index = "shannon",
                              name = "shannon_diversity")     # Use Shannon index

sample_data_13_se <- as.data.frame(colData(tse_subset_by_13kk_ASV_se))

##########
tse_subset_by_13kk_ASV_nse <- mia::addAlpha(tse_subset_by_13kk_ASV_nse, 
                             assay_name = "counts", 
                             index = "observed", 
                             name="observed_richness")


tse_subset_by_13kk_ASV_nse <- mia::addAlpha(tse_subset_by_13kk_ASV_nse, 
                              assay.type = "counts", # Calculate diversity from "counts" assay 
                              index = "shannon",
                              name = "shannon_diversity")     # Use Shannon index

sample_data_13_nse <- as.data.frame(colData(tse_subset_by_13kk_ASV_nse))
```

1. Shannon, 13 month

```
# Initialize a data frame to store results
results_df <- data.frame()

# Define the variables you want to test
hmo_variables <- c("X_2FL_nmol_scale", "X_3FL_nmol_scale", "LNnT_nmol_scale", "X_3SL_nmol_scale", "DFLac_nmol_scale", 
                 "X_6SL_nmol_scale", "LNT_nmol_scale", "LNFP_I_nmol_scale", "LNFP_II_nmol_scale", "LNFP_III_nmol_scale", 
                 "LSTb_nmol_scale", "LSTc_nmol_scale", "DFLNT_nmol_scale", "LNH_nmol_scale", "DSLNT_nmol_scale", 
                 "FLNH_nmol_scale", "DFLNH_nmol_scale", "FDSLNH_nmol_scale", "DSLNH_nmol_scale","Diversity","SUM_nmol_scale", "Sia_scale", "Fuc_scale", "Secretor") 

# Define the adjustment covariates
adjustment_vars <- "SYNNYTYSTAPA_2luok + AIEMMATSYNNYTYKSET_cat + BMI_aiti"

# Initialize a list to store diagnostics
assumption_diagnostics <- list()

# Loop through each HMO variable
for (var in hmo_variables) {
  # Build the formula dynamically to include the covariates
  formula <- as.formula(paste("shannon_diversity ~", var, "+", adjustment_vars))
  
  # Fit the glm model
  model <- glm(formula, data = sample_data_13mo)
  
  # Extract coefficients, standard errors, t-values, p-values, and confidence intervals
  res <- cbind(coefficients(summary(model)), confint(model))
  
  # Convert to a data frame
  res_df <- as.data.frame(res)[2,]
  
  # Add a column for the variable name
  res_df$Variable <- var
  
  # Append to the results data frame
  results_df <- rbind(results_df, res_df)
  
  # Run assumption tests (simulate residuals for model diagnostics)
  simulationOutput <- simulateResiduals(fittedModel = model)
  
  # Store the diagnostics results
  assumption_diagnostics[[var]] <- simulationOutput
}

# Add column names to results_df (if necessary)
colnames(results_df) <- c("Estimate", "Std.Error", "t.value", "Pr(>|t|)", "CI_2.5%", "CI_97.5%", "Variable")

# Extract p-values and apply Benjamini-Hochberg (BH) correction
results_df$BH_Adjusted_P <- p.adjust(results_df$`Pr(>|t|)`, method = "BH")

results_df_11 <- results_df
```

1. Observed richness, 13 month:

```
# Initialize a data frame to store results
results_df <- data.frame()

# Define the variables you want to test
hmo_variables <- c("X_2FL_nmol_scale", "X_3FL_nmol_scale", "LNnT_nmol_scale", "X_3SL_nmol_scale", "DFLac_nmol_scale", 
                 "X_6SL_nmol_scale", "LNT_nmol_scale", "LNFP_I_nmol_scale", "LNFP_II_nmol_scale", "LNFP_III_nmol_scale", 
                 "LSTb_nmol_scale", "LSTc_nmol_scale", "DFLNT_nmol_scale", "LNH_nmol_scale", "DSLNT_nmol_scale", 
                 "FLNH_nmol_scale", "DFLNH_nmol_scale", "FDSLNH_nmol_scale", "DSLNH_nmol_scale","Diversity","SUM_nmol_scale", "Sia_scale", "Fuc_scale", "Secretor") 

# Define the adjustment covariates
adjustment_vars <- "SYNNYTYSTAPA_2luok + AIEMMATSYNNYTYKSET_cat + BMI_aiti"

# Initialize a list to store diagnostics
assumption_diagnostics <- list()

# Loop through each HMO variable
for (var in hmo_variables) {
  # Build the formula dynamically to include the covariates
  formula <- as.formula(paste("observed_richness ~", var, "+", adjustment_vars))
  
  # Fit the glm model
  model <- glm(formula, data = sample_data_13mo)
  
  # Extract coefficients, standard errors, t-values, p-values, and confidence intervals
  res <- cbind(coefficients(summary(model)), confint(model))
  
  # Convert to a data frame
  res_df <- as.data.frame(res)[2,]
  
  # Add a column for the variable name
  res_df$Variable <- var
  
  # Append to the results data frame
  results_df <- rbind(results_df, res_df)
  
  # Run assumption tests (simulate residuals for model diagnostics)
  simulationOutput <- simulateResiduals(fittedModel = model)
  
  # Store the diagnostics results
  assumption_diagnostics[[var]] <- simulationOutput
}

# Add column names to results_df (if necessary)
colnames(results_df) <- c("Estimate", "Std.Error", "t.value", "Pr(>|t|)", "CI_2.5%", "CI_97.5%", "Variable")

# Extract p-values and apply Benjamini-Hochberg (BH) correction
results_df$BH_Adjusted_P <- p.adjust(results_df$`Pr(>|t|)`, method = "BH")

results_df_12 <- results_df
```

2. Secretors, Shannon, 13 month

```
# Initialize a data frame to store results
results_df <- data.frame()

# Define the variables you want to test
hmo_variables <- c("X_2FL_nmol_scale", "X_3FL_nmol_scale", "LNnT_nmol_scale", "X_3SL_nmol_scale", "DFLac_nmol_scale", 
                 "X_6SL_nmol_scale", "LNT_nmol_scale", "LNFP_I_nmol_scale", "LNFP_II_nmol_scale", "LNFP_III_nmol_scale", 
                 "LSTb_nmol_scale", "LSTc_nmol_scale", "DFLNT_nmol_scale", "LNH_nmol_scale", "DSLNT_nmol_scale", 
                 "FLNH_nmol_scale", "DFLNH_nmol_scale", "FDSLNH_nmol_scale", "DSLNH_nmol_scale","Diversity","Evenness","SUM_nmol_scale", "Sia_scale", "Fuc_scale") 

# Define the adjustment covariates
adjustment_vars <- "SYNNYTYSTAPA_2luok + AIEMMATSYNNYTYKSET_cat + BMI_aiti"

# Initialize a list to store diagnostics
assumption_diagnostics <- list()

# Loop through each HMO variable
for (var in hmo_variables) {
  # Build the formula dynamically to include the covariates
  formula <- as.formula(paste("shannon_diversity ~", var, "+", adjustment_vars))
  
  # Fit the glm model
  model <- glm(formula, data = sample_data_13_se)
  
  # Extract coefficients, standard errors, t-values, p-values, and confidence intervals
  res <- cbind(coefficients(summary(model)), confint(model))
  
  # Convert to a data frame
  res_df <- as.data.frame(res)[2,]
  
  # Add a column for the variable name
  res_df$Variable <- var
  
  # Append to the results data frame
  results_df <- rbind(results_df, res_df)
  
  # Run assumption tests (simulate residuals for model diagnostics)
  simulationOutput <- simulateResiduals(fittedModel = model)
  
  # Store the diagnostics results
  assumption_diagnostics[[var]] <- simulationOutput
}

# Add column names to results_df (if necessary)
colnames(results_df) <- c("Estimate", "Std.Error", "t.value", "Pr(>|t|)", "CI_2.5%", "CI_97.5%", "Variable")

# Extract p-values and apply Benjamini-Hochberg (BH) correction
results_df$BH_Adjusted_P <- p.adjust(results_df$`Pr(>|t|)`, method = "BH")

results_df_13 <- results_df
```

2. Secretors, Observed richness, 13 month

```
# Initialize a data frame to store results
results_df <- data.frame()

# Define the variables you want to test
hmo_variables <- c("X_2FL_nmol_scale", "X_3FL_nmol_scale", "LNnT_nmol_scale", "X_3SL_nmol_scale", "DFLac_nmol_scale", 
                 "X_6SL_nmol_scale", "LNT_nmol_scale", "LNFP_I_nmol_scale", "LNFP_II_nmol_scale", "LNFP_III_nmol_scale", 
                 "LSTb_nmol_scale", "LSTc_nmol_scale", "DFLNT_nmol_scale", "LNH_nmol_scale", "DSLNT_nmol_scale", 
                 "FLNH_nmol_scale", "DFLNH_nmol_scale", "FDSLNH_nmol_scale", "DSLNH_nmol_scale","Diversity","Evenness","SUM_nmol_scale", "Sia_scale", "Fuc_scale") 

# Define the adjustment covariates
adjustment_vars <- "SYNNYTYSTAPA_2luok + AIEMMATSYNNYTYKSET_cat + BMI_aiti"

# Initialize a list to store diagnostics
assumption_diagnostics <- list()

# Loop through each HMO variable
for (var in hmo_variables) {
  # Build the formula dynamically to include the covariates
  formula <- as.formula(paste("observed_richness ~", var, "+", adjustment_vars))
  
  # Fit the glm model
  model <- glm(formula, data = sample_data_13_se)
  
  # Extract coefficients, standard errors, t-values, p-values, and confidence intervals
  res <- cbind(coefficients(summary(model)), confint(model))
  
  # Convert to a data frame
  res_df <- as.data.frame(res)[2,]
  
  # Add a column for the variable name
  res_df$Variable <- var
  
  # Append to the results data frame
  results_df <- rbind(results_df, res_df)
  
  # Run assumption tests (simulate residuals for model diagnostics)
  simulationOutput <- simulateResiduals(fittedModel = model)
  
  # Store the diagnostics results
  assumption_diagnostics[[var]] <- simulationOutput
}

# Add column names to results_df (if necessary)
colnames(results_df) <- c("Estimate", "Std.Error", "t.value", "Pr(>|t|)", "CI_2.5%", "CI_97.5%", "Variable")

# Extract p-values and apply Benjamini-Hochberg (BH) correction
results_df$BH_Adjusted_P <- p.adjust(results_df$`Pr(>|t|)`, method = "BH")

results_df_14 <- results_df
```

3. Non-secretors, Shannon, 13 month

```
# Initialize a data frame to store results
results_df <- data.frame()

# Define the variables you want to test
hmo_variables <- c("X_2FL_nmol_scale", "X_3FL_nmol_scale", "LNnT_nmol_scale", "X_3SL_nmol_scale", "DFLac_nmol_scale", 
                 "X_6SL_nmol_scale", "LNT_nmol_scale", "LNFP_I_nmol_scale", "LNFP_II_nmol_scale", "LNFP_III_nmol_scale", 
                 "LSTb_nmol_scale", "LSTc_nmol_scale", "DFLNT_nmol_scale", "LNH_nmol_scale", "DSLNT_nmol_scale", 
                 "FLNH_nmol_scale", "DFLNH_nmol_scale", "FDSLNH_nmol_scale", "DSLNH_nmol_scale","Diversity","Evenness","SUM_nmol_scale", "Sia_scale", "Fuc_scale") 

# Define the adjustment covariates
adjustment_vars <- "SYNNYTYSTAPA_2luok + AIEMMATSYNNYTYKSET_cat + BMI_aiti"

# Initialize a list to store diagnostics
assumption_diagnostics <- list()

# Loop through each HMO variable
for (var in hmo_variables) {
  # Build the formula dynamically to include the covariates
  formula <- as.formula(paste("shannon_diversity ~", var, "+", adjustment_vars))
  
  # Fit the glm model
  model <- glm(formula, data = sample_data_13_nse)
  
  # Extract coefficients, standard errors, t-values, p-values, and confidence intervals
  res <- cbind(coefficients(summary(model)), confint(model))
  
  # Convert to a data frame
  res_df <- as.data.frame(res)[2,]
  
  # Add a column for the variable name
  res_df$Variable <- var
  
  # Append to the results data frame
  results_df <- rbind(results_df, res_df)
  
  # Run assumption tests (simulate residuals for model diagnostics)
  simulationOutput <- simulateResiduals(fittedModel = model)
  
  # Store the diagnostics results
  assumption_diagnostics[[var]] <- simulationOutput
}

# Add column names to results_df (if necessary)
colnames(results_df) <- c("Estimate", "Std.Error", "t.value", "Pr(>|t|)", "CI_2.5%", "CI_97.5%", "Variable")

# Extract p-values and apply Benjamini-Hochberg (BH) correction
results_df$BH_Adjusted_P <- p.adjust(results_df$`Pr(>|t|)`, method = "BH")

results_df_15 <- results_df
```

3. Non-secretors, Observed richness, 13 month

```
# Initialize a data frame to store results
results_df <- data.frame()

# Define the variables you want to test
hmo_variables <- c("X_2FL_nmol_scale", "X_3FL_nmol_scale", "LNnT_nmol_scale", "X_3SL_nmol_scale", "DFLac_nmol_scale", 
                 "X_6SL_nmol_scale", "LNT_nmol_scale", "LNFP_I_nmol_scale", "LNFP_II_nmol_scale", "LNFP_III_nmol_scale", 
                 "LSTb_nmol_scale", "LSTc_nmol_scale", "DFLNT_nmol_scale", "LNH_nmol_scale", "DSLNT_nmol_scale", 
                 "FLNH_nmol_scale", "DFLNH_nmol_scale", "FDSLNH_nmol_scale", "DSLNH_nmol_scale","Diversity","Evenness","SUM_nmol_scale", "Sia_scale", "Fuc_scale") 

# Define the adjustment covariates
adjustment_vars <- "SYNNYTYSTAPA_2luok + AIEMMATSYNNYTYKSET_cat + BMI_aiti"

# Initialize a list to store diagnostics
assumption_diagnostics <- list()

# Loop through each HMO variable
for (var in hmo_variables) {
  # Build the formula dynamically to include the covariates
  formula <- as.formula(paste("observed_richness ~", var, "+", adjustment_vars))
  
  # Fit the glm model
  model <- glm(formula, data = sample_data_13_nse)
  
  # Extract coefficients, standard errors, t-values, p-values, and confidence intervals
  res <- cbind(coefficients(summary(model)), confint(model))
  
  # Convert to a data frame
  res_df <- as.data.frame(res)[2,]
  
  # Add a column for the variable name
  res_df$Variable <- var
  
  # Append to the results data frame
  results_df <- rbind(results_df, res_df)
  
  # Run assumption tests (simulate residuals for model diagnostics)
  simulationOutput <- simulateResiduals(fittedModel = model)
  
  # Store the diagnostics results
  assumption_diagnostics[[var]] <- simulationOutput
}

# Add column names to results_df (if necessary)
colnames(results_df) <- c("Estimate", "Std.Error", "t.value", "Pr(>|t|)", "CI_2.5%", "CI_97.5%", "Variable")

# Extract p-values and apply Benjamini-Hochberg (BH) correction
results_df$BH_Adjusted_P <- p.adjust(results_df$`Pr(>|t|)`, method = "BH")


results_df_16 <- results_df
```

combine data frames:

```
Alpha_div_scale <- list(
  Shannon_3mo = results_df_1,
  Richness_3mo = results_df_2,
  
  Shannon_3mo_se = results_df_3,
  Richness_3mo_se = results_df_4,
  Shannon_3mo_nse = results_df_5,
  Richness_3mo_nse = results_df_6,
  
  Shannon_3mo_exc = results_df_7,
  Richness_3mo_exc = results_df_8,
  Shannon_3mo_se_exc = results_df_9,
  Richness_3mo_se_exc = results_df_10,
  
  Shannon_13mo = results_df_11,
  Richness_13mo = results_df_12,
  
  Shannon_13mo_se = results_df_13,
  Richness_13mo_se = results_df_14,
  Shannon_13mo_nse = results_df_15,
  Richness_13mo_nse = results_df_16
)

# Write to Excel file with each data frame in its own sheet
#write_xlsx(Alpha_div_scale, path = "Alpha_div_scale_final.xlsx")
```

# Maternal secretor status

Association between maternal secretor status and infant gut
microbiota diversity measures in 3-month and 13-month-old infants. Beta
diversity visualized with PCoA for ASV-level data using Bray-Curtis
distances. Ellipses represent maternal secretor status and arrows
represent the direction and strength of correlation between HMO summary
measures and microbiota composition (scaled 3× for visualization) in (A)
3-month-old and (B) 13-month-old infants. Alpha diversity calculated
using Shannon diversity index and Observed species richness between
infants receiving secretor and non-secretor milk in (C) 3-month-old and
(D)13-month-old infants

### Figure 3 (3 months)

```
metadata <- as.data.frame(colData(tse_subset_by_03kk_ASV)) %>%
  dplyr::select(nro, sample_ID , breastfeeding_3kk_category, SYNNYTYSTAPA_2luok,BMI_aiti,AIEMMATSYNNYTYKSET_cat, Secretor, dmmclust, Sia, Fuc, SUM_nmol, Diversity )


tse_subset_by_03kk_ASV <- transformCounts(tse_subset_by_03kk_ASV, assay.type = "counts", method = "relabundance")


# Perform PCoA
method <- "relabundance"
diss_method <- "bray"

tse_subset_by_03kk_ASV <- runMDS(tse_subset_by_03kk_ASV,
              FUN = vegan::vegdist, 
              method = diss_method, 
              name = "PCoA_BC", 
              exprs_values = method)

pcoa <- as.data.frame(reducedDim(tse_subset_by_03kk_ASV, "PCoA_BC"))

pcoa <- rownames_to_column(pcoa)
# Change the column names
colnames(pcoa) <- c("sample_ID", "PCoA1", "PCoA2")
metadata_pcoa <- dplyr::inner_join(metadata, pcoa,  by=c("sample_ID"))

metadata_pcoa <- metadata_pcoa %>%
  mutate(Secretor = ifelse(Secretor == "0", "Non-secretor","Secretor"))

# Calculate explained variance
e <- attr(reducedDim(tse_subset_by_03kk_ASV, "PCoA_BC"), "eig")
rel_eig <- e / sum(e[e > 0])
```

Add HMO values and use envfit function for plotting

```
# extract HMO variables only
# Ensure the rows are in the same order as in the PCoA result
hmo_data <- metadata_pcoa %>%
  select(Sia, Fuc, SUM_nmol, Diversity) 

colnames(hmo_data) <- c("HMO-bound sialic acid", "HMO-bound fucose", "HMO Summary", "HMO Diversity")


# Get PCoA coordinates
pcoa_coords <- metadata_pcoa %>% select(PCoA1, PCoA2)

# Fit HMO vectors
ef <- envfit(pcoa_coords, hmo_data, permutations = 999)

# Convert vectors to data frame
ef_arrows <- as.data.frame(scores(ef, "vectors"))
ef_arrows$HMO <- rownames(ef_arrows)


# Scale the arrows
scale_factor <- 3

ef_arrows_scaled <- ef_arrows %>%
  mutate(
    PCoA1 = PCoA1 * scale_factor,
    PCoA2 = PCoA2 * scale_factor
  )
```

```
# Color scale
secretor_colors <- c("Non-secretor" = "#7A0177", "Secretor" = "#FA9FB5")


pcoa_3 <- metadata_pcoa %>%
  ggplot(aes(x = PCoA1, y = PCoA2, color = Secretor)) +
  geom_point(size = 2, alpha = 0.8) +
  stat_ellipse(aes(group = Secretor), show.legend = FALSE, linewidth = 1) +
  geom_segment(data = ef_arrows_scaled,
               aes(x = 0, y = 0, xend = PCoA1, yend = PCoA2),
               arrow = arrow(length = unit(0.25, "cm")),
               color = "black", linewidth = 0.8,
               inherit.aes = FALSE) +
  geom_text_repel(
    data = ef_arrows_scaled,
    aes(x = PCoA1, y = PCoA2, label = HMO),
    color = "black", size = 3.9,
    inherit.aes = FALSE,
    box.padding = 0.5,
    point.padding = 0.3,
    force = 2,
    max.overlaps = Inf
  ) +
  scale_color_manual(name = "Secretor status", values = secretor_colors) +
  labs(
    title = "nPCoA with Bray-Curtis dissimilarity",
    x = paste0("PCoA 1 (", round(100 * rel_eig[[1]], 1), "%)"),
    y = paste0("PCoA 2 (", round(100 * rel_eig[[2]], 1), "%)")
  ) +
  annotate("text", 
           x = -0.13, y = -0.4, 
           label = "R²= 0.032, p= 0.039*", 
           hjust = 1.1, vjust = 2, size = 4.5, fontface = "italic") +
  theme_classic(base_size = 12) +
  theme(
    legend.key.size = unit(0.8, "cm"),
    legend.text = element_text(size = 12),
    legend.title = element_text(face = "bold", size = 11),
    axis.title = element_text(face = "bold", size = 11),
    plot.title = element_text(hjust = 0.5, face = "bold") 
  )

# Compute one or multiple indices simultaneously through the index 'parameter'. 
tse_subset_by_03kk_ASV <- addAlpha(
    tse_subset_by_03kk_ASV, assay.type = "counts", index = c("observed", "shannon"))


# Create alpha diversity boxplots with updated metric names
col_df <- as.data.frame(colData(tse_subset_by_03kk_ASV))
metrics <- c("observed" = "Observed richness", "shannon" = "Shannon diversity")
col_df <- col_df %>%
  mutate(Secretor = ifelse(Secretor == "0", "Non-secretor","Secretor"))

alpha_plots_3 <- lapply(names(metrics), function(metric) {
  p <- ggplot(col_df, aes(x = Secretor, y = .data[[metric]], fill = Secretor)) +
    geom_boxplot(outlier.shape = NA, width = 0.6, color = "black") +
    geom_jitter(width = 0.15, alpha = 0.6, size = 1) +
    labs(title = metrics[metric], y = metrics[metric]) +
    scale_fill_manual(values = secretor_colors) +
    theme_bw(base_size = 12) +
    guides(fill = "none") +
    theme(
      axis.title.x = element_blank(),
      axis.text.x = element_text(angle = 45, hjust = 1),
      legend.position = "none",
      plot.title = element_text(hjust = 0.5, face = "bold")
    )

  # add significance annotation for Shannon
  if (metric == "shannon") {
    max_y <- max(col_df[[metric]], na.rm = TRUE)
    p <- p +
      annotate("text", x = 1.5, y = max_y * 1.05, label = "*", size = 6) +
      annotate("segment", x = 1, xend = 2, y = max_y * 1.02, yend = max_y * 1.02)
  }

  return(p)
})


# Combine everything
combined_3 <- (pcoa_3 | wrap_plots(alpha_plots_3, ncol = 2)) +
  plot_layout(guides = "collect",
              nrow = 2) &
  theme(legend.position = "none")

combined_3
```

### Figure 3( 13 months)

```
metadata <- as.data.frame(colData(tse_subset_by_13kk_ASV)) %>%
  dplyr::select(nro, sample_ID , breastfeeding_13kk_category, SYNNYTYSTAPA_2luok,BMI_aiti,AIEMMATSYNNYTYKSET_cat, Secretor, dmmclust, Sia, Fuc, SUM_nmol, Diversity )

tse_subset_by_13kk_ASV <- transformCounts(tse_subset_by_13kk_ASV, assay.type = "counts", method = "relabundance")


# Perform PCoA
method <- "relabundance"
diss_method <- "bray"

tse_subset_by_13kk_ASV <- runMDS(tse_subset_by_13kk_ASV,
              FUN = vegan::vegdist, 
              method = diss_method, 
              name = "PCoA_BC", 
              exprs_values = method)


pcoa <- as.data.frame(reducedDim(tse_subset_by_13kk_ASV, "PCoA_BC"))

pcoa <- rownames_to_column(pcoa)
# Change the column names
colnames(pcoa) <- c("sample_ID", "PCoA1", "PCoA2")
metadata_pcoa <- dplyr::inner_join(metadata, pcoa,  by=c("sample_ID"))

metadata_pcoa <- metadata_pcoa %>%
  mutate(Secretor = ifelse(Secretor == "0", "Non-secretor","Secretor"))

# Calculate explained variance
e <- attr(reducedDim(tse_subset_by_13kk_ASV, "PCoA_BC"), "eig")
rel_eig <- e / sum(e[e > 0])
```

Add HMO values and use envfit function for plotting

```
# Example: extract HMO variables only
# Ensure the rows are in the same order as in the PCoA result
hmo_data <- metadata_pcoa %>%
  select( Sia, Fuc, SUM_nmol, Diversity )  # Replace with your actual HMO variable names

colnames(hmo_data) <- c("HMO-bound sialic acid", "HMO-bound fucose", "HMO Summary", "HMO Diversity")


# Get PCoA coordinates
pcoa_coords <- metadata_pcoa %>% select(PCoA1, PCoA2)

# Fit HMO vectors
ef <- envfit(pcoa_coords, hmo_data, permutations = 999)


# Convert vectors to data frame
ef_arrows <- as.data.frame(scores(ef, "vectors"))
ef_arrows$HMO <- rownames(ef_arrows)


# Scale the arrows
scale_factor <- 3

ef_arrows_scaled <- ef_arrows %>%
  mutate(
    PCoA1 = PCoA1 * scale_factor,
    PCoA2 = PCoA2 * scale_factor
  )
```

```
# Color scale
secretor_colors <- c("Non-secretor" = "#7A0177", "Secretor" = "#FA9FB5")

# Update the PCoA plot
pcoa_13 <- metadata_pcoa %>%
  ggplot(aes(x = PCoA1, y = PCoA2, color = Secretor)) +
  geom_point(size = 2, alpha = 0.8) +
  stat_ellipse(aes(group = Secretor), show.legend = FALSE, linewidth = 1) +
  geom_segment(data = ef_arrows_scaled,
               aes(x = 0, y = 0, xend = PCoA1, yend = PCoA2),
               arrow = arrow(length = unit(0.25, "cm")),
               color = "black", linewidth = 0.8,
               inherit.aes = FALSE) +
  geom_text_repel(
  data = ef_arrows_scaled,
  aes(x = PCoA1, y = PCoA2, label = HMO),
  color = "black", size = 3.9,
  inherit.aes = FALSE,
  box.padding = 0.5,    # space around labels
  point.padding = 0.3,  # space around arrow tip
  force = 2,            # stronger repulsion
  max.overlaps = Inf    # has no effect in ggrepel
) +
  scale_color_manual(name = "Secretor status", values = secretor_colors) +
  labs(
    title = "PCoA with Bray-Curtis dissimilarity",   # Heading for PCoA plot
    x = paste0("PCoA 1 (", round(100 * rel_eig[[1]], 1), "%)"),
    y = paste0("PCoA 2 (", round(100 * rel_eig[[2]], 1), "%)")
  ) +
  annotate("text", 
           x = -0.18, y = -0.35, 
           label = "R²= 0.002, p= 0.243", 
           hjust = 1.1, vjust = 2, size = 4.5, fontface = "italic") + # Adds R/p text
  theme_classic(base_size = 12) +
  theme(
    legend.key.size = unit(0.8, "cm"),
    legend.text = element_text(size = 12),
    legend.title = element_text(face = "bold", size = 11),
    axis.title = element_text(face = "bold", size = 11),
    plot.title = element_text(hjust = 0.5, face = "bold")  # center title
  )


# Compute one or multiple indices simultaneously through the index 'parameter'. 
tse_subset_by_13kk_ASV <- addAlpha(
    tse_subset_by_13kk_ASV, assay.type = "counts", index = c("observed", "shannon"))

# Create alpha diversity boxplots with updated metric names
col_df <- as.data.frame(colData(tse_subset_by_13kk_ASV))

# Rename metrics
metrics <- c("observed" = "Observed richness", "shannon" = "Shannon diversity")

col_df <- col_df %>%
  mutate(Secretor = ifelse(Secretor == "0", "Non-secretor","Secretor"))

alpha_plots <- lapply(names(metrics), function(metric) {
  p <- ggplot(col_df, aes(x = Secretor, y = .data[[metric]], fill = Secretor)) +
    geom_boxplot(outlier.shape = NA, width = 0.6, color = "black") +
    geom_jitter(width = 0.15, alpha = 0.6, size = 1) +
    labs(title = metrics[metric], y = metrics[metric]) +
    scale_fill_manual(values = secretor_colors) +
    theme_bw(base_size = 12) +
    guides(fill = "none") +
    theme(
      axis.title.x = element_blank(),
      axis.text.x = element_text(angle = 45, hjust = 1),
      legend.position = "none",
      plot.title = element_text(hjust = 0.5, face = "bold")
    )

  # add significance annotation
  if (metric == "observed") {
    max_y <- max(col_df[[metric]], na.rm = TRUE)
    p <- p +
      annotate("text", x = 1.5, y = max_y * 1.05, label = "*", size = 6) +
      annotate("segment", x = 1, xend = 2, y = max_y * 1.02, yend = max_y * 1.02)
  }

  return(p)
})

# Combine everything
combined_13 <- (pcoa_13 | wrap_plots(alpha_plots, ncol = 2)) +
  plot_layout(guides = "collect",
              nrow = 2)

combined_13
```

```
legend <- cowplot::get_legend(
  combined_13 + theme(legend.position = "right")
)


# removw the legend now
combined_13 <- (pcoa_13 | wrap_plots(alpha_plots, ncol = 2)) +
  plot_layout(guides = "collect",
              nrow = 2) & theme(legend.position = "none")
```

```
# Combine everything

# Put plots together with equal size
plots <- cowplot::plot_grid(combined_3, combined_13, nrow = 1)


# Add the legend on the right side
fig2_combined <- cowplot::plot_grid(
  plots, legend, 
  ncol = 2, 
  rel_widths = c(1, 0.2)  # adjust width ratio
)

#ggsave("fig2_combined2_final.png", width = 15, height = 12, dpi = 300)
```

# DA analysis Secretor status

Differential abundance analysis was performed using the LinDA package
version 0.1.0, which fits linear regression model, and the ALDEx2
package version 1.30.0 which fits regression model on the centered
log-ratio (clr) transformed data. The analysis was conducted to the
genus level data, focusing on genera present in >10% of the
samples.

### 3 months

```
#tse_subset_by_03kk_ASV_cecarean_Genus
# Prepare data
tse_genus <- altExp(tse_subset_by_03kk_ASV, "Genus")
tse0 <- tse_genus %>% subsetByPrevalentTaxa(detection = 0, prevalence = 0.1)
counts <- assay(tse0)
meta <- data.frame(colData(tse0)) %>%
  mutate(libr_size = colSums(counts))


obj_linda <- LinDA::linda(otu.tab = counts, meta = meta,
                   formula = '~ Secretor + BMI_aiti + AIEMMATSYNNYTYKSET_cat + SYNNYTYSTAPA_2luok',
                   adaptive = F,
                   imputation = T,)
                   #pseudo.cnt = .5)


res_linda_environment <- obj_linda$output$Secretor1 %>% 
  rownames_to_column('otu') %>% 
  mutate(est = log2FoldChange,
         se = lfcSE,
         lwr = est + qt(.025, df = df) * se,
         upr = est + qt(.975, df = df) * se)


ggplot(res_linda_environment %>% filter(padj < .05) %>% 
         mutate(OTU = fct_reorder(otu, est)), 
       aes(est, OTU)) +
  geom_vline(xintercept = 0) +
  geom_point() +
  geom_errorbarh(aes(xmin = lwr, xmax = upr), height = .2) +
  labs(x = 'Log2 fold difference') +
  labs(x = 'Ratio of geometric means of absolute abundances') +
  labs(title = "HMO")+
  theme(axis.text.y = element_text(size = 8)) +
  theme_minimal()
```

ALDEx2 3 month

```
library(ALDEx2)

tse_genus <- altExp(tse_subset_by_03kk_ASV, "Genus")
tse00 <- tse_genus %>% subsetByPrevalentTaxa(detection = 0, prevalence = 0.1)
tse0 <- tse00[, !is.na(colData(tse00)$BMI_aiti)] 
counts <- assay(tse0)

meta <- data.frame(colData(tse0))  %>%
  mutate(libr_size = colSums(counts))

#Create model matrix. This needs to be done with covariates
mm <- model.matrix(~ Secretor + BMI_aiti + AIEMMATSYNNYTYKSET_cat + SYNNYTYSTAPA_2luok, meta)

#Create the CLR transformed random samples form Dirichlet distribution
set.seed(123)
aldex_clr <- aldex.clr(counts, mm)

#Run ALDEx2 (This will now take some time..)
obj_aldex <- aldex.glm(aldex_clr, mm)

#Tidy the results from obj_aldex
res_aldex <- obj_aldex %>% 
  rownames_to_column('otu') %>% 
  dplyr::select(otu, 
         est = 'Secretor1:Est',
         se = 'Secretor1:SE',
         p = 'Secretor1:pval') %>% 
 mutate(lwr = est - 2 * se, #It is now possible to calculate sensible
         upr = est + 2 * se, #confidence intervals
         p_adj = p.adjust(p, method = 'BH')) %>% 
  dplyr::left_join(., as.data.frame(rowData(tse0)) %>% #This is here to add the genus
              rownames_to_column("otu"),       #names from the tse object.
            by = 'otu') %>% 
  dplyr::select(-c(se))
                                              

#Visualize the effect sizes of significant taxa
ggplot(res_aldex %>% filter(p_adj < .1) %>% 
         mutate(Genus = fct_reorder(Genus, est)), 
       aes(est, Genus)) +
  geom_vline(xintercept = 0) +
  geom_point(size = 3) +
  labs(x = 'Effect size (difference in CLR transformed counts)') +
  geom_errorbarh(aes(xmin = lwr, xmax = upr), height = .2)+
  theme_minimal()
```

Exclusve BF, 3 month

```
# Prepare data
tse_genus <- altExp(tse_subset_03kk_ASV_BF_Exclus, "Genus")
tse0 <- tse_genus %>% subsetByPrevalentTaxa(detection = 0, prevalence = 0.1)
counts <- assay(tse0)
meta <- data.frame(colData(tse0)) %>%
  mutate(libr_size = colSums(counts))


obj_linda <- LinDA::linda(otu.tab = counts, meta = meta,
                   formula = '~ Secretor + BMI_aiti + AIEMMATSYNNYTYKSET_cat + SYNNYTYSTAPA_2luok',
                   adaptive = F,
                   imputation = T,)
                   #pseudo.cnt = .5)


res_linda_environment <- obj_linda$output$Secretor1 %>% 
  rownames_to_column('otu') %>% 
  mutate(est = log2FoldChange,
         se = lfcSE,
         lwr = est + qt(.025, df = df) * se,
         upr = est + qt(.975, df = df) * se)


ggplot(res_linda_environment %>% filter(padj < .05) %>% 
         mutate(OTU = fct_reorder(otu, est)), 
       aes(est, OTU)) +
  geom_vline(xintercept = 0) +
  geom_point() +
  geom_errorbarh(aes(xmin = lwr, xmax = upr), height = .2) +
  labs(x = 'Log2 fold difference') +
  labs(x = 'Ratio of geometric means of absolute abundances') +
  labs(title = "HMO")+
  theme(axis.text.y = element_text(size = 8)) +
  theme_minimal()
```

ALDEx2, 3 month exclusive BF

```
tse_genus <- altExp(tse_subset_03kk_ASV_BF_Exclus, "Genus")
tse00 <- tse_genus %>% subsetByPrevalentTaxa(detection = 0, prevalence = 0.1)
tse0 <- tse00[, !is.na(colData(tse00)$BMI_aiti)] 
counts <- assay(tse0)

meta <- data.frame(colData(tse0))  %>%
  mutate(libr_size = colSums(counts))

#Create model matrix. This needs to be done with covariates
mm <- model.matrix(~ Secretor + BMI_aiti + AIEMMATSYNNYTYKSET_cat + SYNNYTYSTAPA_2luok, meta)

#Create the CLR transformed random samples form Dirichlet distribution
set.seed(123)
aldex_clr <- aldex.clr(counts, mm)

#Run ALDEx2 (This will now take some time..)
obj_aldex <- aldex.glm(aldex_clr, mm)

#Tidy the results from obj_aldex
res_aldex <- obj_aldex %>% 
  rownames_to_column('otu') %>% 
  dplyr::select(otu, 
         est = 'Secretor1:Est',
         se = 'Secretor1:SE',
         p = 'Secretor1:pval') %>% 
 mutate(lwr = est - 2 * se, #It is now possible to calculate sensible
         upr = est + 2 * se, #confidence intervals
         p_adj = p.adjust(p, method = 'BH')) %>% 
  dplyr::left_join(., as.data.frame(rowData(tse0)) %>% #This is here to add the genus
              rownames_to_column("otu"),       #names from the tse object.
            by = 'otu') %>% 
  dplyr::select(-c(se))
                                              

#Visualize the effect sizes of significant taxa
ggplot(res_aldex %>% filter(p_adj < .1) %>% 
         mutate(Genus = fct_reorder(Genus, est)), 
       aes(est, Genus)) +
  geom_vline(xintercept = 0) +
  geom_point(size = 3) +
  labs(x = 'Effect size (difference in CLR transformed counts)') +
  geom_errorbarh(aes(xmin = lwr, xmax = upr), height = .2)+
  theme_minimal()
```

### 13 months

```
# Prepare data
tse_genus <- altExp(tse_subset_by_13kk_ASV, "Genus")
tse0 <- tse_genus %>% subsetByPrevalentTaxa(detection = 0, prevalence = 0.1)
counts <- assay(tse0)
meta <- data.frame(colData(tse0)) %>%
  mutate(libr_size = colSums(counts))


obj_linda <- LinDA::linda(otu.tab = counts, meta = meta,
                   formula = '~ Secretor + BMI_aiti + AIEMMATSYNNYTYKSET_cat + SYNNYTYSTAPA_2luok',
                   adaptive = F,
                   imputation = T,)
                   #pseudo.cnt = .5)


res_linda_environment <- obj_linda$output$Secretor1 %>% 
  rownames_to_column('otu') %>% 
  mutate(est = log2FoldChange,
         se = lfcSE,
         lwr = est + qt(.025, df = df) * se,
         upr = est + qt(.975, df = df) * se)


ggplot(res_linda_environment %>% filter(padj < .05) %>% 
         mutate(OTU = fct_reorder(otu, est)), 
       aes(est, OTU)) +
  geom_vline(xintercept = 0) +
  geom_point() +
  geom_errorbarh(aes(xmin = lwr, xmax = upr), height = .2) +
  labs(x = 'Log2 fold difference') +
  labs(x = 'Ratio of geometric means of absolute abundances') +
  labs(title = "HMO")+
  theme(axis.text.y = element_text(size = 8)) +
  theme_minimal()
```

# Spearman Correlation Figure 4

### HMO and genera 3 month

```
tse_subset_by_03kk_Genus <- agglomerateByRank(tse_subset_by_03kk_ASV, rank = "Genus")

# STEP 2: Compute relative abundances
tse_subset_by_03kk_Genus <- transformAssay(tse_subset_by_03kk_Genus, assay.type = "counts", method = "relabundance")

# STEP 3: Identify top 19 genera
top_taxa <- getTop(tse_subset_by_03kk_Genus, top = 19, assay.type = "relabundance")

# STEP 4: Rename all other genera as "Other"
Genus_renamed <- lapply(rowData(tse_subset_by_03kk_Genus)$Genus, function(x) {
  if (x %in% top_taxa) x else "Other"
})

rowData(tse_subset_by_03kk_Genus)$Genus_sub <- as.character(Genus_renamed)

# STEP 5: Agglomerate again by top 19 genera + Other
tse_subset_by_03kk_Genus_sub <- agglomerateByVariable(tse_subset_by_03kk_Genus, by = "rows", f = "Genus_sub")

# 6. Extract sample metadata (HMO concentrations)
HMO <- as.data.frame(colData(tse_subset_by_03kk_Genus_sub))

# 7. Select and transpose HMO data
counts <- dplyr::select(HMO, 
                       c("X_2FL_nmol_scale", "X_3FL_nmol_scale", "LNnT_nmol_scale", "X_3SL_nmol_scale", "DFLac_nmol_scale", 
                         "X_6SL_nmol_scale", "LNT_nmol_scale", "LNFP_I_nmol_scale", "LNFP_II_nmol_scale", "LNFP_III_nmol_scale", 
                         "LSTb_nmol_scale", "LSTc_nmol_scale", "DFLNT_nmol_scale", "LNH_nmol_scale", "DSLNT_nmol_scale", 
                         "FLNH_nmol_scale", "DFLNH_nmol_scale", "FDSLNH_nmol_scale", "DSLNH_nmol_scale", "SUM_nmol_scale", "Fuc_scale",
                         "Sia_scale", "Diversity"))
counts <- t(counts)

samples <- as.data.frame(colData(tse_subset_by_03kk_Genus_sub))

# Create a TreeSE for the HMO data
tse_HMO <- TreeSummarizedExperiment(
    assays = SimpleList(counts = as.matrix(counts)),
    colData = DataFrame(samples))
```

```
# Create an ExperimentList that includes experiments
experiments <- ExperimentList(
    microbiome = tse_subset_by_03kk_Genus_sub, HMO = tse_HMO)

# Create a MAE
mae <- MultiAssayExperiment(experiments = experiments)
```

```
# Give unique names, so that we do not have problems when we are creating a plot
rownames(mae[[1]]) <- getTaxonomyLabels(mae[[1]])
rownames(mae[[2]]) <- gsub("^X_", "", gsub("_nmol$", "", gsub("_scale$", "", rownames(mae[[2]]))))


result <- getExperimentCrossAssociation(mae, experiment1= 1, experiment2 = 2, 
                                        assay.type1 = "counts",
                                        assay.type2 = "counts",
                                        method = "spearman",
                                        mode = "matrix", test_significance = TRUE,
                                        show_warnings = TRUE)

add_signif <- function(j, i, x, y, width, height, fill) {
    # If the raw p-value is significant
    if (!is.na(result$pval[i, j]) & result$pval[i, j] < 0.05) {
        # Draw the "X"
        grid.shadowtext("X", x, y, gp = gpar(fontsize = 8, col = "#f5f5f5"))
        
        # If the adjusted p-value is also significant, add a circle
        if (!is.na(result$p_adj[i, j]) & result$p_adj[i, j] < 0.05) {
            grid.circle(x = x, y = y, r = unit(0.015, "npc"), 
                        gp = gpar(col = "black", fill = NA, lwd = 1))
        }
    }
}

heatmap_3_mo <- Heatmap(result$cor,
        # Color scheme for better visualization
        col = colorRamp2(c(-0.2, 0, 0.2), c("blue", "white", "red")),
        
        # Print correlation values on cells
        cell_fun = add_signif,
        
        show_heatmap_legend = FALSE,
        
        # Improve column name readability
        column_names_rot = 50,
        column_names_gp = gpar(fontsize = 10),
        
        # Adjust row name font size
        row_names_gp = gpar(fontsize = 10),
        
        # Adjust heatmap dimensions (margins, padding)
        width = unit(12, "cm"),
        height = unit(12, "cm"),
        row_title = "Top 20 Genera",
        column_title = "3-month-old infants (n=517)",
        row_title_gp = gpar(fontsize = 16, fontface = "bold"),
        column_title_gp = gpar(fontsize = 16, fontface = "bold"),
        
        # Add grid lines for better separation
        rect_gp = gpar(col = "black", lwd = 0.5),
        
        cluster_rows = TRUE,
        cluster_columns = FALSE
)

heatmap_3_mo
```

```
#ggsave("corr_mat_3kk_HMO.tiff", width = 10, height = 10, dpi=300)
```

### Exclusive BF

```
tse_subset_03kk_ASV_BF_Exclus <- tse_subset_by_03kk_ASV[ , tse_subset_by_03kk_ASV$breastfeeding_3kk_category %in% c("exclusive")] 
meta3kk_ecx <- as.data.frame(colData(tse_subset_03kk_ASV_BF_Exclus))
```

```
# Getting top taxa on a Genus level
tse_subset_03kk_Genus_BF_Exclus <- agglomerateByRank(tse_subset_03kk_ASV_BF_Exclus, rank ="Genus")
top_taxa <- getTop(tse_subset_03kk_Genus_BF_Exclus, top = 19, assay.type = "relabundance")

# Renaming the "Genus" rank to keep only top taxa and the rest to "Other"
Genus_renamed <- lapply(rowData(tse_subset_03kk_Genus_BF_Exclus)$Genus, function(x){
    if (x %in% top_taxa) {x} else {"Other"}
    })
rowData(tse_subset_03kk_Genus_BF_Exclus)$Genus_sub <- as.character(Genus_renamed)
# Agglomerate the data based on specified taxa
tse_subset_03kk_Genus_BF_Exclus_sub <- agglomerateByVariable(tse_subset_03kk_Genus_BF_Exclus, by = "rows", f = "Genus_sub")


HMO <- as.data.frame(colData(tse_subset_03kk_Genus_BF_Exclus_sub)) 

#only HMO concentrations
counts <- dplyr::select(HMO, 
                       c("X_2FL_nmol_scale", "X_3FL_nmol_scale", "LNnT_nmol_scale", "X_3SL_nmol_scale", "DFLac_nmol_scale", 
                         "X_6SL_nmol_scale", "LNT_nmol_scale", "LNFP_I_nmol_scale", "LNFP_II_nmol_scale", "LNFP_III_nmol_scale", 
                         "LSTb_nmol_scale", "LSTc_nmol_scale", "DFLNT_nmol_scale", "LNH_nmol_scale", "DSLNT_nmol_scale", 
                         "FLNH_nmol_scale", "DFLNH_nmol_scale", "FDSLNH_nmol_scale", "DSLNH_nmol_scale", "SUM_nmol_scale", "Fuc_scale",
                         "Sia_scale", "Diversity"))

samples <- as.data.frame(colData(tse_subset_03kk_Genus_BF_Exclus_sub))

counts <- t(counts)

# Create a TreeSE for the HMO data
tse_HMO <- TreeSummarizedExperiment(
    assays = SimpleList(counts = as.matrix(counts)),
    colData = DataFrame(samples))
```

```
# Create an ExperimentList that includes experiments
experiments <- ExperimentList(
    microbiome = tse_subset_03kk_Genus_BF_Exclus_sub, HMO = tse_HMO)

# Create a MAE
mae <- MultiAssayExperiment(experiments = experiments)
```

```
# Give unique names, so that we do not have problems when we are creating a plot
rownames(mae[[1]]) <- getTaxonomyLabels(mae[[1]])
rownames(mae[[2]]) <- gsub("^X_", "", gsub("_nmol$", "", gsub("_scale$", "", rownames(mae[[2]]))))


result <- getExperimentCrossAssociation(mae, experiment1= 1, experiment2 = 2, 
                                        assay.type1 = "counts",
                                        assay.type2 = "counts",
                                        method = "spearman",
                                        mode = "matrix", test_significance = TRUE,
                                        show_warnings = TRUE)

add_signif <- function(j, i, x, y, width, height, fill) {
    # If the p-value is under threshold
    if( !is.na(result$pval[i, j]) & result$pval[i, j] < 0.05 ){
        # Print "X"
        grid.shadowtext(
            sprintf("%s", "X"), x, y, gp = gpar(fontsize = 8, col = "#f5f5f5"))
    }
}

heatmap_3_mo_ex <- Heatmap(result$cor,
        # Color scheme for better visualization
        col = colorRamp2(c(-0.2, 0, 0.2),  c("blue", "white", "red")),
        
        # Print correlation values on cells
        cell_fun = add_signif,
        
        show_heatmap_legend = FALSE,
        
        # Improve column name readability
        column_names_rot = 50,
        column_names_gp = gpar(fontsize = 10),
        
        # Adjust row name font size
        row_names_gp = gpar(fontsize = 10),
        
        # Adjust heatmap dimensions (margins, padding)
        width = unit(12, "cm"),
        height = unit(12, "cm"),
        row_title = "Top 20 Genera",
        column_title = "Exclusively BF 3-month-old infants (n=259)",
        row_title_gp = gpar(fontsize = 16, fontface = "bold"),
        column_title_gp = gpar(fontsize = 16, fontface = "bold"),
        
        # Add grid lines for better separation
        rect_gp = gpar(col = "black", lwd = 0.5),
        
        cluster_rows = TRUE,
        cluster_columns = FALSE
)

heatmap_3_mo_ex
```

```
#ggsave("corr_mat_3kk_HMO.tiff", width = 10, height = 10, dpi=300)
```

### 13-month

```
# Getting top taxa on a Genus level
tse_subset_by_13kk_Genus <- agglomerateByRank(tse_subset_by_13kk_ASV, rank ="Genus")
top_taxa <- getTop(tse_subset_by_13kk_Genus, top = 19, assay.type = "relabundance")

# Renaming the "Genus" rank to keep only top taxa and the rest to "Other"
Genus_renamed <- lapply(rowData(tse_subset_by_13kk_Genus)$Genus, function(x){
    if (x %in% top_taxa) {x} else {"Other"}
    })
rowData(tse_subset_by_13kk_Genus)$Genus_sub <- as.character(Genus_renamed)
# Agglomerate the data based on specified taxa
tse_subset_by_13kk_Genus_sub <- agglomerateByVariable(tse_subset_by_13kk_Genus, by = "rows", f = "Genus_sub")


HMO <- as.data.frame(colData(tse_subset_by_13kk_Genus_sub)) 

#only HMO concentrations
counts <- dplyr::select(HMO, 
                       c("X_2FL_nmol_scale", "X_3FL_nmol_scale", "LNnT_nmol_scale", "X_3SL_nmol_scale", "DFLac_nmol_scale", 
                         "X_6SL_nmol_scale", "LNT_nmol_scale", "LNFP_I_nmol_scale", "LNFP_II_nmol_scale", "LNFP_III_nmol_scale", 
                         "LSTb_nmol_scale", "LSTc_nmol_scale", "DFLNT_nmol_scale", "LNH_nmol_scale", "DSLNT_nmol_scale", 
                         "FLNH_nmol_scale", "DFLNH_nmol_scale", "FDSLNH_nmol_scale", "DSLNH_nmol_scale", "SUM_nmol_scale", "Fuc_scale",
                         "Sia_scale", "Diversity"))

samples <- as.data.frame(colData(tse_subset_by_13kk_Genus_sub))

counts <- t(counts)


# Create a TreeSE for the HMO data
tse_HMO <- TreeSummarizedExperiment(
    assays = SimpleList(counts = as.matrix(counts)),
    colData = DataFrame(samples))
```

```
# Create an ExperimentList that includes experiments
experiments <- ExperimentList(
    microbiome = tse_subset_by_13kk_Genus_sub, HMO = tse_HMO)

# Create a MAE
mae <- MultiAssayExperiment(experiments = experiments)
```

```
# Give unique names, so that we do not have problems when we are creating a plot
rownames(mae[[1]]) <- getTaxonomyLabels(mae[[1]])
rownames(mae[[2]]) <- gsub("^X_", "", gsub("_nmol$", "", gsub("_scale$", "", rownames(mae[[2]]))))


result <- getExperimentCrossAssociation(mae, experiment1= 1, experiment2 = 2, 
                                        assay.type1 = "counts",
                                        assay.type2 = "counts",
                                        method = "spearman",
                                        mode = "matrix", test_significance = TRUE,
                                        show_warnings = TRUE)


add_signif <- function(j, i, x, y, width, height, fill) {
    # If the raw p-value is significant
    if (!is.na(result$pval[i, j]) & result$pval[i, j] < 0.05) {
        # Draw the "X"
        grid.shadowtext("X", x, y, gp = gpar(fontsize = 8, col = "#f5f5f5"))
        
        # If the adjusted p-value is also significant, add a circle
        if (!is.na(result$p_adj[i, j]) & result$p_adj[i, j] < 0.05) {
            grid.circle(x = x, y = y, r = unit(0.03, "npc"), 
                        gp = gpar(col = "black", fill = NA, lwd = 1.5))
        }
    }
}


heatmap_13_mo <- Heatmap(
  result$cor,
  
  # Color scheme for better visualization
  col = colorRamp2(c(-0.2, 0, 0.2), c("blue", "white", "red")),
  
  # Print correlation values on cells
  cell_fun = add_signif,
  
  # Adjust legend appearance (add direction = "horizontal")
  heatmap_legend_param = list(
    title = "Spearman\ncorrelation",
    legend_height = unit(5, "cm"),
    title_gp = gpar(fontsize = 12, fontface = "bold"),
    labels_gp = gpar(fontsize = 10),
    title_position = "topcenter",
    direction = "horizontal"       # <— added line
  ),
  
  # Improve column name readability
  column_names_rot = 50,
  column_names_gp = gpar(fontsize = 10),
  
  # Adjust row name font size
  row_names_gp = gpar(fontsize = 10),
  
  # Adjust heatmap dimensions (margins, padding)
  width = unit(12, "cm"),
  height = unit(12, "cm"),
  row_title = "Top 20 Genera",
  column_title = "13-month-old infants (n=522)",
  row_title_gp = gpar(fontsize = 16, fontface = "bold"),
  column_title_gp = gpar(fontsize = 16, fontface = "bold"),
  
  # Add grid lines for better separation
  rect_gp = gpar(col = "black", lwd = 0.5),
  
  cluster_rows = TRUE,
  cluster_columns = FALSE
)

# Draw the heatmap with legend below
heatmap_13_mo_v2 <- draw(
  heatmap_13_mo,
  heatmap_legend_side = "bottom"
)
```

```
fig_spearman <- cowplot::plot_grid(grid.grabExpr(draw(heatmap_3_mo)), grid.grabExpr(draw(heatmap_3_mo_ex)), grid.grabExpr(draw(heatmap_13_mo_v2)), nrow = 3, labels = c('A', "B", "C"))

#ggsave("fig_spearman_test.png", width = 8, height = ,dpi = 300)
```

# Cesaran delivery and HMO

```
tse_subset_by_03kk_ASV_cecarean<- tse_subset_by_03kk_ASV[ , tse_subset_by_03kk_ASV$SYNNYTYSTAPA_2luok %in% c("2")]

meta_cecarean_3mo <- as.data.frame(colData(tse_subset_by_03kk_ASV_cecarean))


tse_subset_by_13kk_ASV_cecarean <- tse_subset_by_13kk_ASV[ , tse_subset_by_13kk_ASV$SYNNYTYSTAPA_2luok %in% c("2")]

meta_cecarean_13mo <- as.data.frame(colData(tse_subset_by_13kk_ASV_cecarean))
```

## 3 months

### Binomial FCT

Supplementary 1

relative abundance of top to 10 genera of cesarean born infants in
FCT1&2 (n=30) and FCT3 (n=25) per sample in 3 month timepoint.

```
tse_subset_by_03kk_ASV_cecarean_Genus <- altExp(tse_subset_by_03kk_ASV_cecarean, "Genus")

colData(tse_subset_by_03kk_ASV_cecarean_Genus) <- colData(tse_subset_by_03kk_ASV_cecarean) 
colData(tse_subset_by_03kk_ASV_cecarean_Genus)$FCT <- ifelse(colData(tse_subset_by_03kk_ASV_cecarean_Genus)$dmmclust == 3, "FCT3", "FCT1&2")


# Computing relative abundance
tse_subset_by_03kk_ASV_cecarean_Genus <- transformAssay(tse_subset_by_03kk_ASV_cecarean_Genus, assay.type = "counts", method = "relabundance")

top_taxa <- getTop(tse_subset_by_03kk_ASV_cecarean_Genus, top = 9, assay.type = "relabundance")

# Renaming the "Phylum" rank to keep only top taxa and the rest to "Other"
genus_renamed <- lapply(rowData(tse_subset_by_03kk_ASV_cecarean_Genus)$Genus, function(x){
    if (x %in% top_taxa) {x} else {"Other"}
    })
rowData(tse_subset_by_03kk_ASV_cecarean_Genus)$genus_sub <- as.character(genus_renamed)
# Agglomerate the data based on specified taxa
tse_sub <- agglomerateByVariable(tse_subset_by_03kk_ASV_cecarean_Genus, by = "rows", f = "genus_sub")

plotAbundance(
    tse_sub, 
    assay.type = "relabundance", 
    order.col.by = "Other",
    col.var = "FCT", 
    add.legend = FALSE,
    add.x.text = TRUE,
    facet.cols = TRUE, 
    scales = "free_x"
) +
    theme_minimal(base_size = 14) +  # Modern minimal theme with larger base font
    theme(
        axis.text.x = element_blank(),  # Rotate x-axis labels
        axis.title = element_text(size = 14, face = "bold"),  # Improve axis titles
        strip.text = element_text(size = 12, face = "bold"),  # Facet labels
        panel.grid.major = element_blank(),  # Remove major grid lines
        panel.grid.minor = element_blank()   # Remove minor grid lines
    ) +
    scale_fill_brewer(palette = "Paired", name = "Genus")  # Use a better color palette for clarity
```

```
#ggsave("community_bar_plot_DMM_3mo_cecarean_bysample.tiff", width = 8, height = 6)
```

### Characteristics table

Characteristics table of the secarean infants in DMM clusters

To determine why a specific child is in one group (Group\_1\_2) versus
the other (Group\_3) and identify which variables (HMOs, sex, age,
antibiotics, etc.) influence group membership

3 month

```
meta_cecarean_3mo <- as.data.frame(colData(tse_subset_by_03kk_ASV_cecarean))

meta_cecarean_df <- dplyr::select(meta_cecarean_3mo, 
                       c("SP",
                         "SYNTYMAPAINO_kg",
                         "KESTOVKPV_num", 
                         "BMI_aiti", 
                         "Aidinika",
                         "aidinab", 
                         "Antib", 
                         "ika_3kk",
                         "season_3kk", 
                         "dmmclust",
                         "lib_size", 
                         "AIEMMATSYNNYTYKSET_cat",
                         "Secretor",
                         "breastfeeding_3kk_category",
                         "X_2FL_nmol", "X_3FL_nmol", "LNnT_nmol", "X_3SL_nmol", 
                                                "DFLac_nmol", "X_6SL_nmol", "LNT_nmol", "LNFP_I_nmol", 
                                                "LNFP_II_nmol", "LNFP_III_nmol", "LSTb_nmol", "LSTc_nmol", 
                                                "DFLNT_nmol", "LNH_nmol", "DSLNT_nmol", "FLNH_nmol", 
                                                "DFLNH_nmol", "FDSLNH_nmol", "DSLNH_nmol","Diversity", "SUM_nmol", 
                                                "Sia", "Fuc"))

meta_cecarean_df$FCT <- ifelse(meta_cecarean_df$dmmclust == 3, "FCT3", "FCT1&2")

# Convert group to a binary outcome (0 = Group_1_2, 1 = Group_3)
meta_cecarean_df$FCT_binary <- ifelse(meta_cecarean_df$FCT == "FCT3", 1, 0)
```

```
meta_cecarean_df_table <- dplyr::select(meta_cecarean_df, 
                       c("SP",
                         "SYNTYMAPAINO_kg",
                         "KESTOVKPV_num",
                         "BMI_aiti", 
                         "Aidinika",
                         "aidinab", 
                         "Antib", 
                         "ika_3kk",
                         "season_3kk", 
                         "dmmclust",
                         "lib_size", 
                         "AIEMMATSYNNYTYKSET_cat",
                         "Secretor",
                         "breastfeeding_3kk_category",
                         "FCT"
                         ))


# Define continuous and categorical variables
continuous_vars <- c("SYNTYMAPAINO_kg", "KESTOVKPV_num", "Aidinika", "ika_3kk", "lib_size", "BMI_aiti")
categorical_vars <- c("SP", "aidinab", "Antib",  "season_3kk","AIEMMATSYNNYTYKSET_cat", "Secretor", "breastfeeding_3kk_category")

# Combine variables into a single list
vars <- c(continuous_vars, categorical_vars)


# Create table and specify tests
table_one1 <- CreateTableOne(
  vars = vars,
  strata = "FCT",  # Groups to compare
  data = meta_cecarean_df_table,
  factorVars = categorical_vars,  # Categorical variables
  smd = TRUE,  # Add standardized mean differences
  addOverall = TRUE  # Add overall summary
)

# Step 1: Print with all levels (this works for console and data capture)
table_one_printed <- print(table_one1,
                           quote = FALSE,
                           noSpaces = TRUE,
                           printToggle = FALSE,
                           showAllLevels = TRUE)

# Step 2: Convert properly: row names → "Variable" column
table_one_df_cs_3mo <- cbind(Variable = rownames(table_one_printed),
                      as.data.frame.matrix(table_one_printed))  # better than data.frame()


#Write to Excel
#write_xlsx(table_one_df_cs_3mo, "table_one_df_cs_3mo.xlsx")

#Test statistical significance: 

# # Grouping variable
# group_var <- "FCT"
# 
# # Define continuous and categorical variables
# continuous_vars <- c("SYNTYMAPAINO_kg", "KESTOVKPV_num", "BMI_aiti", 
#                      "Aidinika", "ika_3kk", "lib_size")
# 
# categorical_vars <- c("SP", "aidinab", "Antib",  
#                       "season_3kk", "AIEMMATSYNNYTYKSET_cat", 
#                       "Secretor", "breastfeeding_3kk_category")
# 
# for (var in continuous_vars) {
#   cat("\nwilcox test for:", var, "\n")
#   print(wilcox.test(as.formula(paste(var, "~", group_var)), data = meta_cecarean_df_table))
# }
# Since Chi-squared test is not suitable for small sample sizes use fisher test for categorical variables in cesarean group. 

# for (var in categorical_vars) {
#   # Remove NAs to avoid errors in table()
#   df_clean <- meta_cecarean_df_table[!is.na(meta_cecarean_df_table[[var]]) & !is.na(meta_cecarean_df_table[[group_var]]), ]
#   
#   cat("\nFisher's exact test for:", var, "\n")
#   tbl <- table(df_clean[[var]], df_clean[[group_var]])
#   print(fisher.test(tbl))
# }
```

Characteristics table of the secarean infants in DMM clusters

13 month

```
meta_cecarean_3mo$FCT <- ifelse(meta_cecarean_3mo$dmmclust == 3, "FCT3", "FCT1&2")

# Convert group to a binary outcome (0 = Group_1_2, 1 = Group_3)
meta_cecarean_3mo$FCT_binary <- ifelse(meta_cecarean_3mo$FCT == "FCT3", 1, 0)

meta3kk_cs_sub <- meta_cecarean_3mo %>%
  dplyr::select(nro, FCT_binary, FCT)

common_ids <- intersect(meta_cecarean_3mo$nro, meta_cecarean_13mo$nro)
```

```
# Extract sample IDs (nro) from the colData of the SummarizedExperiment object
sample_ids_tse <- colData(tse_subset_by_13kk_ASV_cecarean)$nro

# Subset the SummarizedExperiment object to include only samples with common IDs
tse_subset_common_13 <- tse_subset_by_13kk_ASV_cecarean[, sample_ids_tse %in% common_ids]

subset_common_13mo1 <- as.data.frame(colData(tse_subset_common_13))
subset_common_13mo <- inner_join(subset_common_13mo1, meta3kk_cs_sub, by = "nro")
```

```
subset_common_13mo_table <- dplyr::select(subset_common_13mo, 
                       c("SP",
                         "SYNTYMAPAINO_kg",
                         "KESTOVKPV_num",
                         "BMI_aiti", 
                         "Aidinika",
                         "aidinab", 
                         "Antib", 
                         "ika_13kk",
                         "season_13kk", 
                         "dmmclust",
                         "lib_size", 
                         "AIEMMATSYNNYTYKSET_cat",
                         "Secretor",
                         "breastfeeding_13kk_category",
                         "FCT"
                         ))


# Define continuous and categorical variables
continuous_vars <- c("SYNTYMAPAINO_kg", "KESTOVKPV_num", "Aidinika", "ika_13kk", "lib_size", "BMI_aiti", "FCT")
categorical_vars <- c("SP", "aidinab", "Antib",  "season_13kk","AIEMMATSYNNYTYKSET_cat", "Secretor", "breastfeeding_13kk_category")

# Combine variables into a single list
vars <- c(continuous_vars, categorical_vars)

# Create table and specify tests
table_one13 <- CreateTableOne(
  vars = vars,
  strata = "dmmclust",  # Groups to compare
  data = subset_common_13mo_table,
  factorVars = categorical_vars,  # Categorical variables
  smd = TRUE,  # Add standardized mean differences
  addOverall = TRUE  # Add overall summary
)


# Step 1: Print with all levels (this works for console and data capture)
table_one_printed <- print(table_one13,
                           quote = FALSE,
                           noSpaces = TRUE,
                           printToggle = FALSE,
                           showAllLevels = TRUE)

# Step 2: Convert properly: row names → "Variable" column
table_one_df_cs_13mo <- cbind(Variable = rownames(table_one_printed),
                      as.data.frame.matrix(table_one_printed))  # better than data.frame()


#Write to Excel
#write_xlsx(table_one_df_cs_13mo, "table_one_df_cs_13mo.xlsx")

#Test statistical significance: 

# # Grouping variable
# group_var <- "dmmclust"
# 
# # Define continuous and categorical variables
# continuous_vars <- c("SYNTYMAPAINO_kg", "KESTOVKPV_num", "BMI_aiti", 
#                      "Aidinika", "ika_13kk", "lib_size")
# 
# categorical_vars <- c("SP", "aidinab", "Antib",  
#                       "season_13kk", "AIEMMATSYNNYTYKSET_cat", 
#                       "Secretor", "breastfeeding_13kk_category", "FCT")
# for (var in continuous_vars) {
#   cat("\nKruskal-Wallis test for:", var, "\n")
#   print(kruskal.test(as.formula(paste(var, "~", group_var)), data = subset_common_13mo_table))
# }
# 
# 
# for (var in categorical_vars) {
#   # Remove NAs
#   df_clean <- subset_common_13mo_table[
#     !is.na(subset_common_13mo_table[[var]]) & 
#     !is.na(subset_common_13mo_table[[group_var]]), ]
#   
#   cat("\nFisher's exact test for:", var, "\n")
#   
#   tbl <- table(df_clean[[var]], df_clean[[group_var]])
#   
#   # Skip empty tables
#   if (sum(tbl) == 0) {
#     cat("Skipping", var, "- table has no data.\n")
#     next
#   }
#   
#   #  Fisher’s test
#   # For large tables use Monte Carlo to avoid computational overload
#   if (nrow(tbl) > 2 || ncol(tbl) > 2) {
#     print(fisher.test(tbl))
#   } else {
#     print(fisher.test(tbl))
#   }
# }
```

## Logistic regression

Association between HMO and binary FCTs at 3 month (logistic
regression)

model: glm(DMM ~ DSLNT, data=mata3kk, family=binomial)

```
meta3kk <- as.data.frame(colData(tse_subset_by_03kk_ASV_cecarean))

meta3kk$FCT <- ifelse(meta3kk$dmmclust == 3, "FCT3", "FCT1&2")

# Convert group to a binary outcome (0 = Group_1_2, 1 = Group_3)
meta3kk$FCT_binary <- ifelse(meta3kk$FCT == "FCT3", 1, 0)
```

```
meta3kk$LNFP_III_nmol_scale <- ifelse(meta3kk$LNFP_III_nmol_scale > 15, 
                                      0, 
                                      meta3kk$LNFP_III_nmol_scale)
```

```
# Define HMO variables
hmo_vars <- c("SUM_nmol_scale", "Diversity", "Fuc_scale","Sia_scale", "X_2FL_nmol_scale", "X_3FL_nmol_scale", "LNnT_nmol_scale", "X_3SL_nmol_scale", "DFLac_nmol_scale", 
              "X_6SL_nmol_scale", "LNT_nmol_scale", "LNFP_I_nmol_scale", "LNFP_II_nmol_scale", "LNFP_III_nmol_scale", 
              "LSTb_nmol_scale", "LSTc_nmol_scale", "DFLNT_nmol_scale", "LNH_nmol_scale", "DSLNT_nmol_scale", 
              "FLNH_nmol_scale", "DFLNH_nmol_scale", "FDSLNH_nmol_scale", "DSLNH_nmol_scale")

# Initialize results list without p-values
results <- data.frame(HMO = character(),
                      OR = numeric(),
                      CI_lower = numeric(),
                      CI_upper = numeric(),
                      stringsAsFactors = FALSE)

# Loop through each HMO
for (hmo in hmo_vars) {
  # Dynamically build formula
  formula <- as.formula(paste("FCT_binary ~", hmo, "+ AIEMMATSYNNYTYKSET_cat + BMI_aiti"))
  
  # Fit logistic regression model
  model <- glm(formula, data = meta3kk, family = binomial())
  
  # Extract stats
  coef_info <- summary(model)$coefficients
  confint_vals <- suppressMessages(confint(model))  # suppress CI warning about profile likelihood

  # Calculate OR and CI for the HMO
  or <- exp(coef_info[hmo, "Estimate"])
  ci_lower <- exp(confint_vals[hmo, 1])
  ci_upper <- exp(confint_vals[hmo, 2])

  # Append to results
  results <- rbind(results, data.frame(HMO = hmo,
                                       OR = or,
                                       CI_lower = ci_lower,
                                       CI_upper = ci_upper))
}

# View results
print(results)
```

```
##                    HMO         OR  CI_lower   CI_upper
## 1       SUM_nmol_scale  0.6333953 0.3590214   1.023463
## 2            Diversity  1.1594281 0.7815983   1.747910
## 3            Fuc_scale  0.6556807 0.3679754   1.091947
## 4            Sia_scale  0.8063671 0.4693073   1.311787
## 5     X_2FL_nmol_scale  0.6572303 0.3777914   1.089433
## 6     X_3FL_nmol_scale  0.9737881 0.6446198   1.404322
## 7      LNnT_nmol_scale  0.8446616 0.4351138   1.613483
## 8     X_3SL_nmol_scale  0.7455259 0.3633004   1.255237
## 9     DFLac_nmol_scale  0.8686832 0.5392782   1.269604
## 10    X_6SL_nmol_scale  0.9209063 0.4613812   1.744621
## 11      LNT_nmol_scale  1.1862136 0.6933892   2.106538
## 12   LNFP_I_nmol_scale  0.8925762 0.4824364   1.627606
## 13  LNFP_II_nmol_scale  1.2198916 0.7191623   2.131394
## 14 LNFP_III_nmol_scale 16.3035613 1.1427619 391.208766
## 15     LSTb_nmol_scale  0.8540223 0.4110555   1.676474
## 16     LSTc_nmol_scale  1.1527661 0.7539889   1.980088
## 17    DFLNT_nmol_scale  0.8644065 0.4995084   1.488773
## 18      LNH_nmol_scale  1.4840649 0.9246845   3.818980
## 19    DSLNT_nmol_scale  0.9034633 0.5546716   1.391765
## 20     FLNH_nmol_scale  1.3155638 0.8776121   2.331499
## 21    DFLNH_nmol_scale  1.1280941 0.8570318   1.621978
## 22   FDSLNH_nmol_scale  0.9739970 0.6354812   1.506619
## 23    DSLNH_nmol_scale  0.8532717 0.4825913   1.468279
```

```
#write_xlsx(results, "HMO_cs_results_3mo.xlsx")
```

Secretor status

```
# Run logistic regression for Secretor
model_secretor <- glm(FCT_binary ~ Secretor + AIEMMATSYNNYTYKSET_cat + BMI_aiti,
                      data = meta3kk, family = binomial())

coef_info <- summary(model_secretor)$coefficients
confint_vals <- suppressMessages(confint(model_secretor))

# Extract OR and CI for the "Secretor" effect (the non-reference category)
row_name <- grep("^Secretor", rownames(coef_info), value = TRUE)

or <- exp(coef_info[row_name, "Estimate"])
ci_lower <- exp(confint_vals[row_name, 1])
ci_upper <- exp(confint_vals[row_name, 2])

# Append to results
results <- rbind(results,
                 data.frame(HMO = "Secretor",
                            OR = or,
                            CI_lower = ci_lower,
                            CI_upper = ci_upper))
```

Plot the results (HMO + Secretor status)

```
results <- results %>%
  filter(HMO != "LNFP_III_nmol_scale")

#clean the names
results$HMO <- gsub("^X_", "", results$HMO)            
results$HMO <- gsub("_nmol_scale$", "", results$HMO) 
results$HMO <- gsub("_scale$", "", results$HMO) 

custom_order <- c("Secretor",
                  "SUM",
                  "Sia",
                  "Fuc",
                  "Diversity",
                  "2FL",
                  "3FL",
                  "LNnT",
                  "3SL",
                  "DFLac",
                  "6SL",
                  "LNT",
                  "LNFP_I",
                  "LNFP_II",
                  "LSTb",
                  "LSTc",
                  "DFLNT",
                  "LNH",
                  "DSLNT",
                  "FLNH",
                  "DFLNH",
                  "FDSLNH",
                  "DSLNH")

results$HMO <- factor(results$HMO, levels = custom_order)


cesarean <- ggplot(results, aes(x = HMO, y = OR, ymin = CI_lower, ymax = CI_upper)) +
  geom_pointrange() +
  geom_hline(yintercept = 1, linetype = "dashed", color = "red") +
  coord_flip() +
  scale_x_discrete(drop = FALSE, limits = rev(custom_order)) +  # reverse order top→bottom
  theme_classic(base_size = 14) +
  labs(x = "", y = "Odds Ratio (95% CI)")

cesarean
```

```
#ggsave("cesarean.png", width = 8, height = 8, dpi = 300)
```
