# Supplementary material for: The role of human milk oligosaccharides in shaping and restoring infant gut microbiota: population-based cohort study
Source: Am J Clin Nutr. 2026 Apr 16;123(6):101318. doi: 10.1016/j.ajcnut.2026.101318 (PMC13269344; doi:10.1016/j.ajcnut.2026.101318)
Supplement: Multimedia component 2 [file mmc2.docx]

**Supplemental material**

**The role of human milk oligosaccharides in shaping and restoring infant gut microbiota: Population-based cohort study**

Minka Ovaska ^a b^, Manu Tamminen ^c^, Mirkka Lahdenperä ^c^, Samuli Rautava ^d^, Aditya Jeevannavar ^c^, Heidi Isokääntä ^b e f^, Lars Bode ^g^, Hanna Lagström ^a b h^

^a^ Department of Public Health, University of Turku and Turku University Hospital, Turku, Finland

^b^ Centre for Population Health Research, University of Turku and Turku University Hospital, Turku, Finland

^c^ Department of Biology, University of Turku, Turku, Finland

^d^ Department of Pediatrics, University of Helsinki and Helsinki University Hospital and New Children’s Hospital, Pediatric Research Center, Helsinki, Finland

^e^ Research Center for Infections and Immunity, Institute of Biomedicine, University of Turku, Turku, Finland

^f^ Turku Bioscience Centre, University of Turku, Turku, Finland

^g^ Department of Pediatrics, Larsson-Rosenquist Foundation Mother-Milk-Infant Center of Research Excellence (LRF MOMI CORE), and the Human Milk Institute (HMI), University of California San Diego, La Jolla, CA, USA

^h^ Nutrition and Food Research Center, Faculty of Medicine, University of Turku, Turku, Finland

* Correspondence: Minka Ovaska, mmovas@utu.fi

**Content:**

**Figure 1.** Sequencing library size (number of sequencing reads)

**Figure 2.** Dagitty model for the exposures known to associate with human milk and infant gut microbiome

**Table 1.** Table for interpreting the z-score transformed HMO concentration results. Median, mean and standard deviation (std) values per HMO per: 3-month-old infants

**Table 2.** Table for interpreting the z-score transformed HMO concentration results. Median, mean and standard deviation (std) values per HMO per: 3-month-old exclusively BF infants

**Table 3.** Table for interpreting the z-score transformed HMO concentration results. Median, mean and standard deviation (std) values per HMO per: 13-month-old infants

**Figure 3.** DMM clustering in 3-month-old infants. Genus level data

**Figure 4.** DMM clustering in 13-month-old infants. Genus level data

**Table 4**. Differences in descriptive characteristics of the study population between DMM clusters in 3-month-old infants.

**Table 5**. Differences in descriptive characteristics of the study population between DMM clusters in 13-month-old infants

**Table 6**. Summary table of the associations between individual HMOs and HMO summary measures and gut microbiota in 3-month-old infants

**Table 7**. Summary table of the associations between individual HMOs and HMO summary measures and gut microbiota in 13-month-old infants

**Figure 5.** Spearman correlation between individual HMO variables and histogram of the HMO distribution.

**Table 8.** Association of z-score transformed HMO concentrations with FCTs in 3-month-old (n=517) and 13-month-old (n=522) infants assessed with covariate-adjusted multinomial logistic regression models.

**Table 9.** Association of z-score transformed HMO concentrations with Shannon diversity and Observed richness in 3-month-old (n=517) and 13-month-old (n=522) infants assessed with covariate-adjusted linear model.

**Table 10.** Sensitivity analyses of breastfeeding status. Association of z-score transformed HMO concentrations a with FCTs in exclusively BF 3-month-old (n= 259) and the whole cohort of 13-month-old (n=522) infants assessed with covariate-adjusted multinomial logistic regression models.

**Table 11**. Summary table of the associations between individual HMOs and HMO summary measures and gut microbiota in 3-month-old exclusively BF infants

**Table 12.** Sensitivity analyses of breastfeeding status. Association of z-score transformed HMO concentrations a with Shannon diversity and Observed richness in exclusively BF 3-month-old (n=259) and in the whole cohort of 13-month-old (n=522) infants assessed with covariate-adjusted linear model.

**Figure 6**. Relative abundance of top to 10 genera of cesarean born infants in FCT1&2 (n=30) and FCT3 (n=25) per sample.

**Table 13**. Differences in descriptive characteristics of the cesarean born infants between binary DMM clusters (FCT1&2 and FCT3) in 3-month-old infants

**Table 14.** The relationship between maternal HMO composition and FCTs in a subcohort of cesarean-born infants (n=55)

**Table 15.** Differences in descriptive characteristics of the cesarean born infants between FCTs in 13-month-old infants.


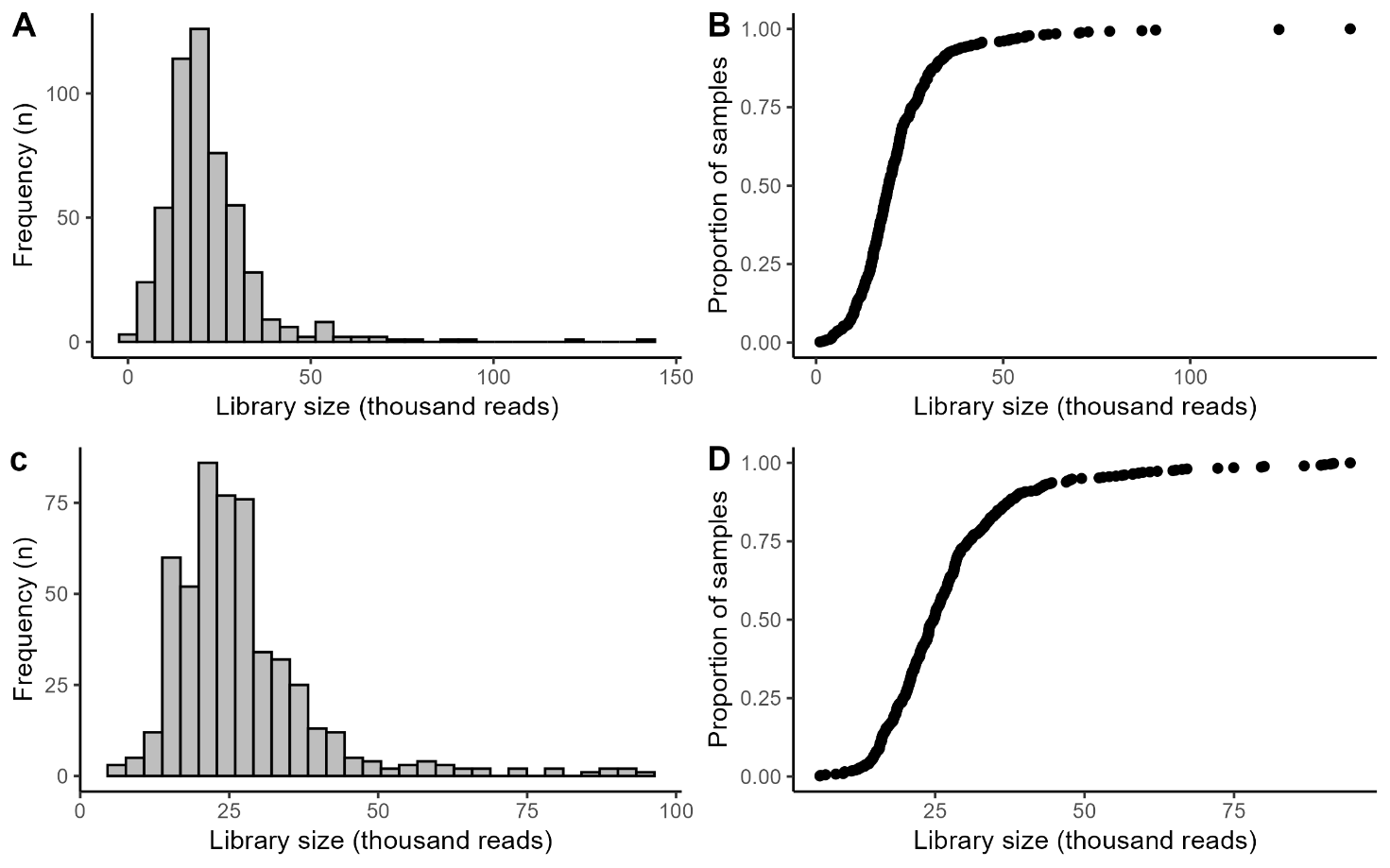


**Figure 1.** Sequencing library size (number of sequencing reads). 3-month (A, B) and 13-month (C, D) samples


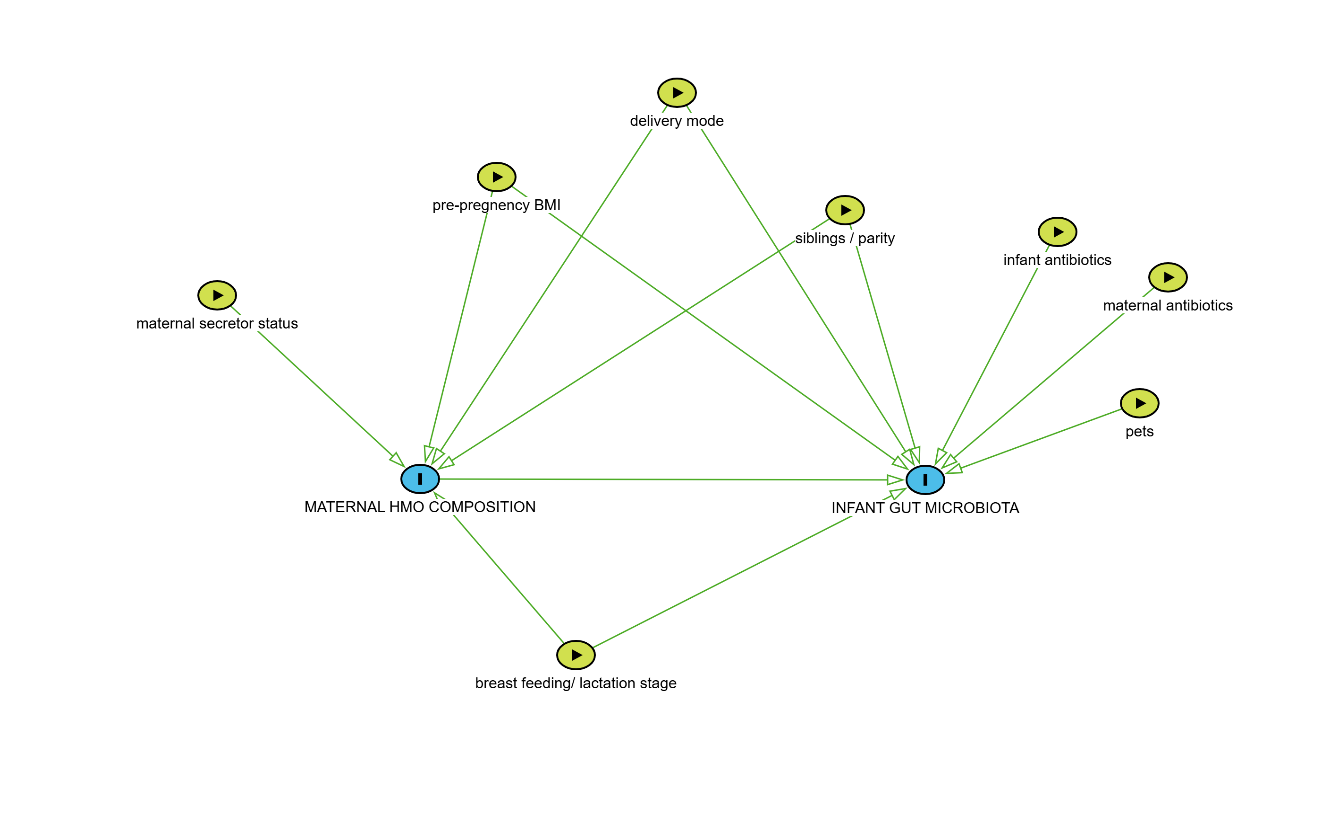


**Figure 2.** Dagitty model for the exposures known to associate with human milk and infant gut microbiota. We selected the following factors, associated with both maternal HMO composition and infant gut microbiota, as covariates: delivery mode (vaginal or caesarean section delivery), siblings/parity (primiparous or multiparous), and maternal pre-pregnancy BMI.

**Table 1.** Table for interpreting the z-score transformed HMO concentration results. Median, mean and standard deviation (std) values for HMO concentrations (nmol/mL) per: 3-month-old infants (n=517), 3-month-old infants receiving secretor milk (n=463) and 3-month-old infants receiving non-secretor milk (n=54).

|  | 3-month old infants (n=517) | | | 3-month-old infants receiving secretor milk (n=463) | | | 3-month-old infants receiving non-secretor milk (n=54) | | |
| --- | --- | --- | --- | --- | --- | --- | --- | --- | --- |
| HMO | median | mean | std | median | mean | std | median | mean | std |
| Total HMO concentration | 16242,4 | 15741,3 | 2492,9 | 16412,1 | 16500,4 | 1168,9 | 9189,8 | 9232,7 | 630,6 |
| HMO-bound sialic acid | 2768,4 | 2880,2 | 808,1 | 2681,1 | 2730,6 | 672,7 | 4223,9 | 4162,6 | 736,2 |
| HMO-bound fucose | 14590,8 | 13818,4 | 3318,4 | 14877,5 | 14811,5 | 1608,4 | 5513,2 | 5303,4 | 1472,4 |
| Diversity | 5,1 | 5,1 | 1,5 | 4,9 | 5,0 | 1,5 | 5,5 | 5,6 | 0,8 |
| 2FL | 6174,8 | 6076,7 | 2910,7 | 6468,0 | 6775,3 | 2186,0 | 68,5 | 86,5 | 68,2 |
| 3FL | 372,4 | 403,8 | 220,7 | 391,1 | 433,2 | 209,6 | 121,1 | 152,4 | 139,5 |
| LNnT | 976,8 | 1037,7 | 427,3 | 974,5 | 1025,7 | 382,2 | 1052,7 | 1141,1 | 701,0 |
| 3SL | 529,9 | 607,0 | 338,5 | 545,8 | 623,9 | 349,3 | 454,8 | 462,2 | 168,0 |
| DFLac | 497,5 | 557,3 | 422,4 | 540,9 | 621,1 | 400,3 | 8,5 | 10,6 | 9,7 |
| 6SL | 537,5 | 665,7 | 436,1 | 511,9 | 592,1 | 301,1 | 1187,0 | 1296,8 | 779,5 |
| LNT | 885,9 | 931,3 | 437,3 | 895,9 | 935,5 | 401,2 | 664,4 | 895,4 | 676,4 |
| LNFP I | 1127,1 | 1244,3 | 839,7 | 1226,2 | 1378,8 | 782,4 | 72,9 | 90,8 | 122,0 |
| LNFP II | 1533,2 | 1645,9 | 712,5 | 1459,8 | 1512,9 | 550,4 | 3050,1 | 2786,6 | 905,9 |
| LNFP III | 72,4 | 87,7 | 149,3 | 69,2 | 74,7 | 34,5 | 134,8 | 199,1 | 438,7 |
| LSTb | 108,5 | 119,1 | 64,6 | 103,0 | 113,1 | 62,3 | 159,4 | 169,9 | 61,8 |
| LSTc | 74,1 | 85,5 | 49,9 | 75,8 | 87,0 | 47,1 | 62,4 | 72,6 | 68,6 |
| DFLNT | 1528,3 | 1406,5 | 616,3 | 1577,7 | 1498,2 | 571,3 | 559,8 | 619,8 | 384,4 |
| LNH | 56,3 | 66,9 | 56,2 | 55,8 | 64,2 | 38,5 | 67,2 | 89,6 | 131,3 |
| DSLNT | 309,0 | 333,9 | 168,9 | 304,1 | 331,3 | 170,4 | 360,1 | 356,6 | 154,6 |
| FLNH | 53,3 | 64,3 | 48,6 | 55,7 | 65,5 | 42,9 | 24,5 | 54,0 | 82,5 |
| DFLNH | 37,5 | 40,1 | 25,3 | 39,7 | 41,9 | 19,9 | 12,6 | 24,8 | 50,4 |
| FDSLNH | 238,2 | 287,8 | 198,1 | 225,8 | 248,7 | 130,6 | 600,8 | 623,7 | 324,2 |
| DSLNH | 67,7 | 79,7 | 54,9 | 66,2 | 77,3 | 55,4 | 98,9 | 100,3 | 45,1 |

**Table 2.** Table for interpreting the z-score transformed HMO concentration results. Median, mean and standard deviation (std) values for HMO concentrations (nmol/mL) per: 3-month-old exclusively BF infants (n=259) 3-month-old exclusively BF infants receiving secretor milk (n=224).

|  | 3-month-old exclusively BF infants (n=259) | | | 3-month-old exclusively BF infants receiving secretor milk (n=224) | | |
| --- | --- | --- | --- | --- | --- | --- |
| HMO | median | mean | std | median | mean | std |
| Total HMO concentration | 16268,9 | 15769,4 | 2428,3 | 16427,3 | 16479,0 | 1120,3 |
| HMO-bound sialic acid | 2762,5 | 2835,4 | 771,2 | 2637,4 | 2668,7 | 583,1 |
| HMO-bound fucose | 14710,1 | 13887,1 | 3211,5 | 14891,9 | 14804,6 | 1590,5 |
| Diversity | 5,0 | 5,1 | 1,4 | 4,9 | 5,0 | 1,5 |
| 2FL | 6254,6 | 6182,7 | 2843,1 | 6606,7 | 6833,0 | 2133,3 |
| 3FL | 371,0 | 396,5 | 189,8 | 385,2 | 422,9 | 178,9 |
| LNnT | 1008,9 | 1054,3 | 429,4 | 997,4 | 1042,4 | 379,4 |
| 3SL | 527,4 | 586,0 | 250,2 | 552,5 | 598,8 | 257,0 |
| DFLac | 495,0 | 512,3 | 284,6 | 526,4 | 566,0 | 244,4 |
| 6SL | 549,1 | 661,0 | 397,8 | 518,1 | 592,5 | 297,7 |
| LNT | 886,1 | 915,1 | 418,0 | 898,0 | 939,0 | 400,8 |
| LNFP I | 1091,8 | 1201,6 | 782,2 | 1179,7 | 1323,3 | 723,3 |
| LNFP II | 1569,3 | 1669,1 | 682,1 | 1520,2 | 1539,9 | 534,1 |
| LNFP III | 74,9 | 85,2 | 46,4 | 72,4 | 76,8 | 34,0 |
| LSTb | 108,5 | 115,3 | 57,2 | 104,7 | 108,7 | 52,1 |
| LSTc | 72,7 | 80,7 | 40,9 | 74,3 | 82,0 | 40,2 |
| DFLNT | 1556,0 | 1441,4 | 600,2 | 1621,8 | 1534,1 | 551,8 |
| LNH | 60,1 | 69,0 | 42,0 | 60,0 | 67,9 | 40,9 |
| DSLNT | 288,4 | 317,4 | 149,5 | 287,9 | 312,6 | 150,6 |
| FLNH | 55,6 | 64,1 | 43,1 | 57,5 | 66,9 | 40,7 |
| DFLNH | 37,7 | 38,9 | 19,7 | 39,6 | 41,6 | 18,7 |
| FDSLNH | 255,4 | 302,6 | 202,9 | 242,9 | 258,5 | 120,4 |
| DSLNH | 66,9 | 76,2 | 42,3 | 64,6 | 72,2 | 39,6 |

**Table 3.** Table for interpreting the z-score transformed HMO concentration results. Median, mean and standard deviation (std) values for HMO concentrations (nmol/mL) per: 13-month-old infants (n=522), 13-month-old infants receiving secretor milk (n=465), and 13-month-old infants receiving non-secretor milk (n=5).

|  | 13-month-old infants (n=522) | | | 13-month-old infants receiving secretor milk (n=465) | | | 13-month-old infants receiving non-secretor milk (n=57) | | |
| --- | --- | --- | --- | --- | --- | --- | --- | --- | --- |
| HMO | median | mean | std | median | mean | std | median | mean | std |
| Total HMO concentration | 16226,2 | 15712,0 | 2542,8 | 16412,1 | 16507,6 | 1186,8 | 9193,2 | 9221,8 | 594,1 |
| HMO-bound sialic acid | 2777,5 | 2894,3 | 835,3 | 2669,2 | 2730,0 | 678,8 | 4211,7 | 4234,8 | 787,5 |
| HMO-bound fucose | 14561,5 | 13768,2 | 3381,9 | 14865,6 | 14805,2 | 1649,7 | 5456,5 | 5307,9 | 1440,3 |
| Diversity | 5,0 | 5,1 | 1,5 | 4,9 | 5,0 | 1,5 | 5,6 | 5,7 | 0,8 |
| 2FL | 6210,1 | 6083,7 | 2982,7 | 6566,6 | 6818,3 | 2244,1 | 61,8 | 91,1 | 94,9 |
| 3FL | 370,2 | 400,6 | 217,4 | 389,7 | 431,3 | 205,5 | 123,2 | 149,8 | 134,4 |
| LNnT | 972,5 | 1030,5 | 406,7 | 969,6 | 1022,2 | 381,0 | 1048,6 | 1098,4 | 575,1 |
| 3SL | 519,0 | 591,9 | 313,3 | 537,0 | 606,7 | 322,2 | 424,1 | 471,2 | 190,9 |
| DFLac | 501,0 | 548,8 | 391,4 | 546,8 | 614,8 | 363,5 | 8,5 | 10,9 | 9,6 |
| 6SL | 548,4 | 685,2 | 447,3 | 519,0 | 609,8 | 319,2 | 1174,4 | 1299,6 | 765,3 |
| LNT | 852,9 | 918,7 | 436,1 | 861,2 | 925,7 | 402,3 | 691,9 | 861,4 | 651,9 |
| LNFP I | 1137,7 | 1239,8 | 837,1 | 1233,0 | 1380,2 | 777,3 | 76,5 | 94,6 | 121,6 |
| LNFP II | 1535,4 | 1652,3 | 722,0 | 1459,8 | 1509,8 | 556,9 | 3057,7 | 2814,7 | 857,9 |
| LNFP III | 74,3 | 90,2 | 149,0 | 71,0 | 76,4 | 34,6 | 144,4 | 203,6 | 426,8 |
| LSTb | 108,5 | 119,6 | 66,3 | 103,4 | 112,7 | 62,7 | 160,2 | 175,3 | 69,0 |
| LSTc | 73,7 | 85,8 | 51,7 | 75,7 | 87,2 | 49,3 | 61,1 | 74,3 | 67,8 |
| DFLNT | 1507,5 | 1385,4 | 619,1 | 1568,5 | 1481,1 | 573,6 | 559,1 | 604,7 | 379,5 |
| LNH | 58,6 | 68,3 | 57,0 | 57,7 | 66,0 | 40,3 | 67,8 | 87,7 | 127,9 |
| DSLNT | 309,3 | 338,1 | 176,6 | 305,8 | 333,1 | 175,4 | 383,5 | 379,3 | 182,9 |
| FLNH | 52,9 | 65,3 | 50,1 | 55,5 | 66,6 | 44,8 | 26,1 | 54,6 | 80,9 |
| DFLNH | 37,7 | 39,9 | 24,9 | 40,1 | 42,0 | 19,2 | 12,0 | 22,7 | 48,7 |
| FDSLNH | 236,8 | 288,0 | 198,2 | 222,6 | 246,9 | 131,1 | 600,3 | 623,0 | 308,0 |
| DSLNH | 68,2 | 79,9 | 54,5 | 66,3 | 76,8 | 54,6 | 101,3 | 104,8 | 48,0 |


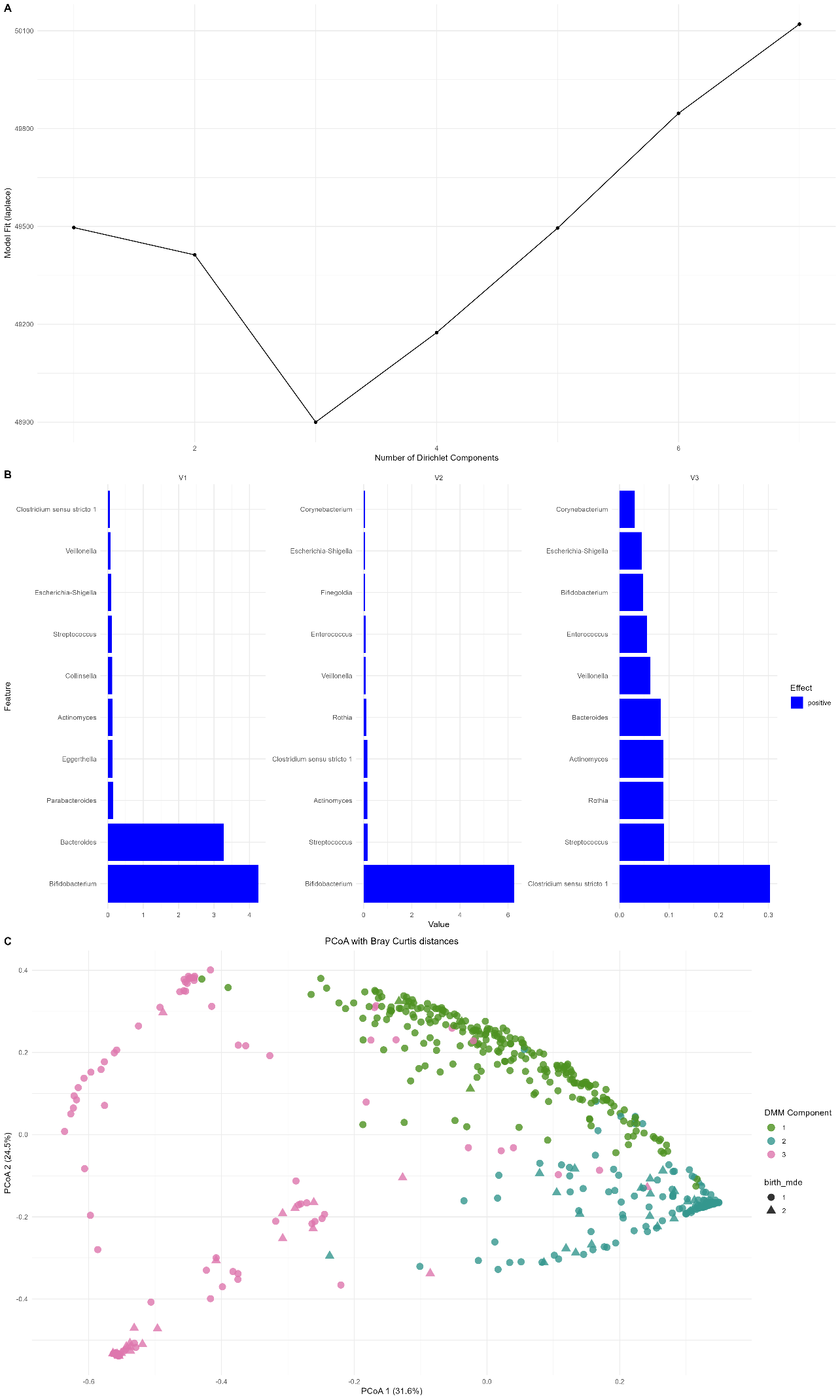


**Figure 3.** DMM clustering in 3-month-old infants. Genus level data. A) Laplace approximation to the model evidence for each of the seven models, B) taxonomic features defining the key cluster features, C) clusters visualized using PCoA ordination and Bray-Curtis dissimilarity index calculated on genus level data.


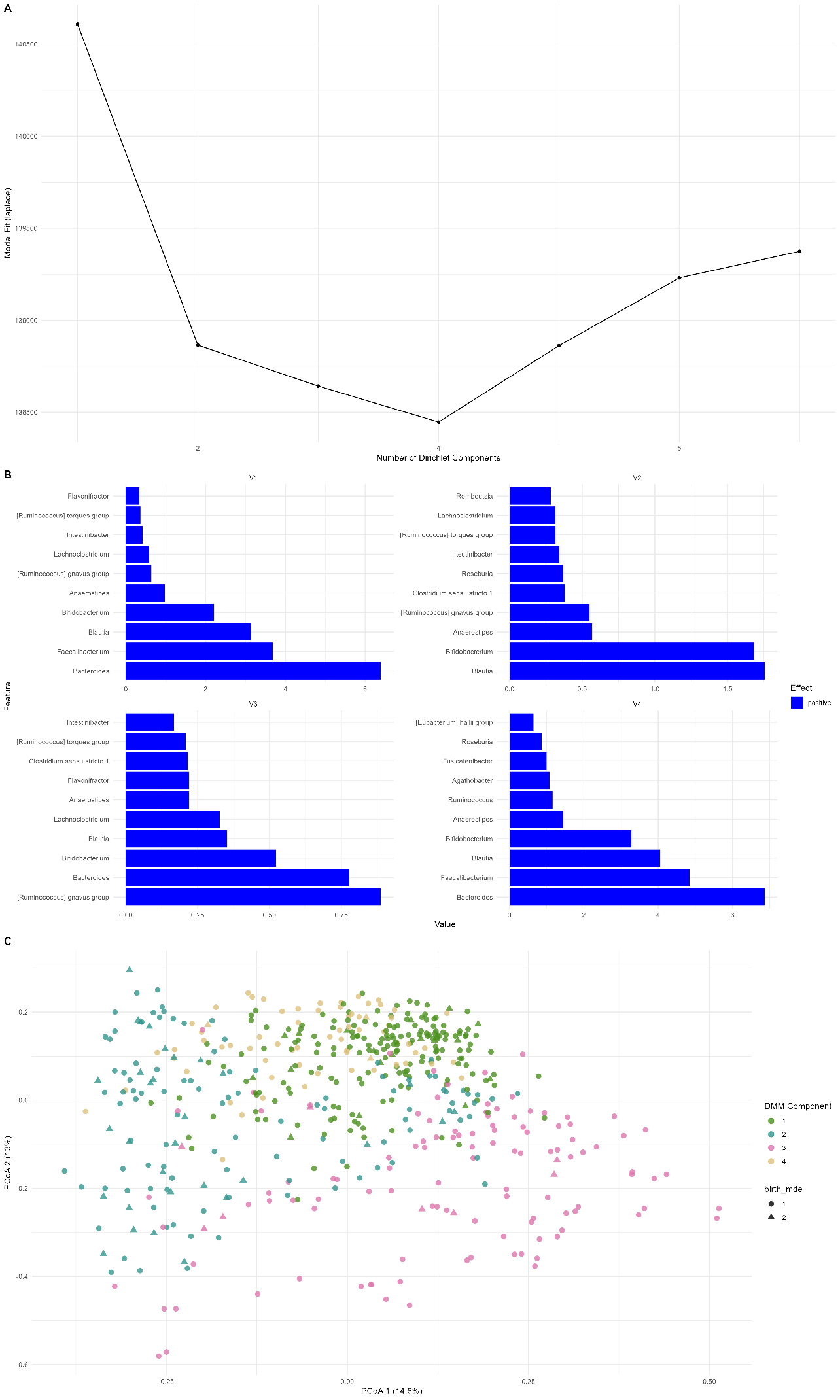


**Figure 4.** DMM clustering in 13-month-old infants. Genus level data. A) Laplace approximation to the model evidence for each of the seven models, B) taxonomic features defining the key cluster features, C) clusters visualized using PCoA ordination and Bray-Curtis dissimilarity index calculated on genus level data.

**Table 4**. Differences in descriptive characteristics of the study population between FCTs in 3-month-old infants. Sample sizes and percentages are given for categorical variables and means with standard deviations for continuous variables. Continuous variables tested with Kruskal-Wallis test due to non-normal distributions and categorical variables tested with Chi-squared test. Bolded values represent statistically significant (p<0.05) differences between the FCTs.

| **Variable** | **level** | **Overall** | **3M-FCT1** | **3M-FCT2** | **3M-FCT3** | **p** |
| --- | --- | --- | --- | --- | --- | --- |
| n |  | 517 | 235 | 169 | 113 |  |
| Birth weight (mean (SD)) |  | 3.58 (0.46) | 3.61 (0.46) | 3.53 (0.44) | 3.57 (0.50) | 0.492 |
| Duration of pregnancy (mean (SD)) |  | 40.08 (1.23) | 40.11 (1.24) | 40.04 (1.20) | 40.06 (1.26) | 0.809 |
| Mothers age (mean (SD)) |  | 31.25 (4.32) | 31.72 (4.54) | 30.71 (4.11) | 31.11 (4.10) | **0.035** |
| Infant age (months)(mean (SD)) |  | 2.83 (0.45) | 2.85 (0.44) | 2.83 (0.45) | 2.79 (0.44) | 0.175 |
| Number of sequencing reads (mean (SD)) |  | 21868 (13541) | 25783 (16352) | 19729 (9210) | 16927 (9755) | **<0.001** |
| Pre-preganency BMI (mean (SD)) |  | 24.23 (4.69) | 24.48 (4.64) | 24.16 (4.55) | 23.81 (4.99) | 0.1086 |
| Sex (%) | Boy | 281 (54.4) | 131 (55.7) | 90 (53.3) | 60 (53.1) | 0.845 |
|  | Girl | 236 (45.6) | 104 (44.3) | 79 (46.7) | 53 (46.9) |  |
| Birth mode (%) | Vaginal | 462 (89.4) | 232 (98.7) | 142 (84.0) | 88 (77.9) | **<0.001** |
|  | Cecarean | 55 (10.6) | 3 (1.3) | 27 (16.0) | 25 (22.1) |  |
| Maternal antibiotics (%) | No | 454 (87.8) | 216 (91.9) | 143 (84.6) | 95 (84.1) | **0.034** |
|  | Yes | 63 (12.2) | 19 (8.1) | 26 (15.4) | 18 (15.9) |  |
| Infant antibiotics (%) | No | 455 (88.0) | 215 (91.5) | 146 (86.4) | 94 (83.2) | 0.061 |
|  | Yes | 62 (12.0) | 20 (8.5) | 23 (13.6) | 19 (16.8) |  |
| Previous deliveries (%) | >0 | 199 (38.5) | 105 (44.7) | 64 (37.9) | 30 (26.5) | **0.005** |
|  | no previous deliveries | 318 (61.5) | 130 (55.3) | 105 (62.1) | 83 (73.5) |  |
| Secretor status (%) | No | 54 (10.4) | 23 (9.8) | 10 (5.9) | 21 (18.6) | **0.003** |
|  | Yes | 463 (89.6) | 212 (90.2) | 159 (94.1) | 92 (81.4) |  |
| Breastfeeding status (%) | exclusive | 259 (50.1) | 116 (49.4) | 89 (52.7) | 54 (47.8) | 0.889 |
|  | no | 17 (3.3) | 6 (2.6) | 6 (3.6) | 5 (4.4) |  |
|  | partial | 154 (29.8) | 71 (30.2) | 50 (29.6) | 33 (29.2) |  |
|  | unknown | 87 (16.8) | 42 (17.9) | 24 (14.2) | 21 (18.6) |  |

**Table 5**. Differences in descriptive characteristics of the study population between FCTs in 13-month-old infants. Sample sizes and percentages are given for categorical variables and means with standard deviations for continuous variables. Continuous variables tested with Kruskal-Wallis test due to non-normal distributions and categorical variables tested with Chi-squared test. Bolded values represent statistically significant (p<0.05) differences between the FCTs.

| **Variable** | **level** | **Overall** | **13M-FCT1** | **13M-FCT2** | **13M-FCT3** | **13M-FCT4** | **p** |
| --- | --- | --- | --- | --- | --- | --- | --- |
| n |  | 522 | 167 | 139 | 111 | 105 |  |
| Birth weight (mean (SD)) |  | 3.58 (0.46) | 3.61 (0.43) | 3.54 (0.45) | 3.49 (0.46) | 3.67 (0.51) | **0.015** |
| Duration of pregnancy (mean (SD)) |  | 40.05 (1.22) | 39.95 (1.20) | 40.14 (1.21) | 39.91 (1.22) | 40.21 (1.24) | 0.226 |
| Mothers age (mean (SD)) |  | 31.34 (4.44) | 31.33 (4.60) | 31.45 (4.46) | 30.92 (4.23) | 31.66 (4.40) | 0.509 |
| Infant age (mean (SD)) |  | 13.67 (0.66) | 13.76 (0.75) | 13.63 (0.60) | 13.58 (0.64) | 13.67 (0.60) | 0.115 |
| Number of sequencing reads (mean (SD)) |  | 27197 (12732) | 28953 (13992) | 26008 (13357) | 24254 (9624) | 29090 (12048) | **<0.001** |
| Pre-pregnency BMI (mean (SD)) |  | 24.17 (4.60) | 24.49 (4.65) | 23.36 (3.85) | 23.69 (4.16) | 25.22 (5.56) | **0.047** |
| Sex (%) | Boy | 279 (53.4) | 86 (51.5) | 73 (52.5) | 59 (53.2) | 61 (58.1) | 0.748 |
|  | Girl | 243 (46.6) | 81 (48.5) | 66 (47.5) | 52 (46.8) | 44 (41.9) |  |
| Birth mode (%) | Vaginal | 462 (88.5) | 153 (91.6) | 107 (77.0) | 105 (94.6) | 97 (92.4) | **<0.001** |
|  | Cecarean | 60 (11.5) | 14 (8.4) | 32 (23.0) | 6 (5.4) | 8 (7.6) |  |
| Maternal antibiotics (%) | No | 459 (87.9) | 150 (89.8) | 114 (82.0) | 99 (89.2) | 96 (91.4) | 0.089 |
|  | Yes | 63 (12.1) | 17 (10.2) | 25 (18.0) | 12 (10.8) | 9 (8.6) |  |
| Infant antibiotics (%) | No | 462 (88.5) | 152 (91.0) | 112 (80.6) | 101 (91.0) | 97 (92.4) | **0.008** |
|  | Yes | 60 (11.5) | 15 (9.0) | 27 (19.4) | 10 (9.0) | 8 (7.6) |  |
| Previous deliveries (%) | >0 | 201 (38.5) | 76 (45.5) | 36 (25.9) | 29 (26.1) | 60 (57.1) | **<0.001** |
|  | no previous deliveries | 321 (61.5) | 91 (54.5) | 103 (74.1) | 82 (73.9) | 45 (42.9) |  |
| Secretor (%) | No | 57 (10.9) | 19 (11.4) | 15 (10.8) | 8 (7.2) | 15 (14.3) | 0.418 |
|  | Yes | 465 (89.1) | 148 (88.6) | 124 (89.2) | 103 (92.8) | 90 (85.7) |  |
| Breastfeeding status (%) | no | 343 (65.7) | 112 (67.1) | 99 (71.2) | 65 (58.6) | 67 (63.8) | 0.431 |
|  | partial | 70 (13.4) | 21 (12.6) | 13 (9.4) | 20 (18.0) | 16 (15.2) |  |
|  | unknown | 109 (20.9) | 34 (20.4) | 27 (19.4) | 26 (23.4) | 22 (21.0) |  |

**Table 6**. Summary table of the associations between individual HMOs and HMO summary measures and gut microbiota beta diversity, alpha diversity (Shannon diversity, Observed richness), and FCT types in 3-month-old infants (n=517), 3-month-old infants receiving milk from secretors (n=463) and non-secretors (n=54). Beta diversity was studied using variable selection, followed by covariate adjusted permutational multivariate analysis of variance (PERMANOVA), alpha diversity was studied with covariate-adjusted linear models and FCT analyses was conducted with covariate-adjusted multinomial logistic regression models. The covariates used in the models were: birth mode (vaginal or caesarean section delivery), parity (primiparous or multiparous), and maternal pre-pregnancy BMI. In alpha diversity columns the (+) indicates increasing and (-) decreasing association of the HMO to the diversity metrics. Bolded and underlined values (**X**) represent BH-corrected statistical significance (p<0.05).

|  | 3-month (n=517) | | | | 3-month secretors (n=463) (x) non-secretors (n=54) (o) | | | |
| --- | --- | --- | --- | --- | --- | --- | --- | --- |
|  | Beta div | Shannon index | Species richness | FCT types | Beta div | Shannon index | Species richness | FCT types |
| Secretor status | x | **x (-)** |  | x |  |  |  |  |
| Total HMO concentration |  | **x (-)** |  | **x** |  |  |  |  |
| HMO diversity |  | x (+) |  |  |  |  |  |  |
| HMO-bound fucose |  | **x (-)** |  | **x** |  |  |  |  |
| HMO-bound sialic acid |  | **x (+)** | **x (+)** |  |  | x (+) | **x (+)** |  |
| 2’FL |  | **x (-)** |  | x |  |  |  |  |
| 3FL |  |  |  |  |  |  | x (+) |  |
| LNnT |  |  |  |  |  |  |  |  |
| 3’SL |  | x (+) | **x (+)** |  |  | x (+) | **x (+)** |  |
| DFLac |  |  | **x (+)** | x |  | **x (+)** | **x (+)** |  |
| 6’SL |  | **x (+)** |  | x |  |  |  |  |
| LNT |  |  |  |  |  |  |  |  |
| LNFP I |  |  |  |  |  |  |  |  |
| LNFP II |  | **x (+)** |  | x |  |  |  |  |
| LNFP III |  |  |  | **x** |  |  |  |  |
| LSTb |  | **x (+)** | **x (+)** |  |  | **x (+)** | **x (+)** |  |
| LSTc |  |  |  |  |  |  |  |  |
| DFLNT |  |  |  |  |  |  |  |  |
| LNH |  |  |  |  |  |  |  |  |
| DSLNT |  | x (+) | x (+) |  |  | x (+) | x (+) |  |
| FLNH |  |  |  |  |  |  |  |  |
| DFLNH |  |  |  |  |  |  |  |  |
| FDSLNH |  |  |  | **x** |  |  |  |  |
| DSLNH |  |  |  |  |  |  |  |  |

**Table 7.** Summary table of the associations between individual HMOs and HMO summary measures and gut microbiota beta diversity, alpha diversity (Shannon diversity, observed richness), and FCT types in 13-month-old infants (n=522), 13-month-old infants receiving milk from secretors (n=465) and non-secretors (n=57). Beta diversity was studied using variable selection, followed by covariate adjusted permutational multivariate analysis of variance (PERMANOVA), alpha diversity was studied with covariate-adjusted linear models and FCT analyses was conducted with covariate-adjusted multinomial logistic regression models. The covariates used in the models were: birth mode (vaginal or caesarean section delivery), parity (primiparous or multiparous), and maternal pre-pregnancy BMI. In alpha diversity columns the (+) indicates increasing and (-) decreasing association of the HMO to the diversity metrics. Bolded and underlined values **(X)** represent BH-corrected statistical significance (p<0.05).

|  | 13-month (n=522) | | | | 13-month secretors (n=465) (x) non-secretors (n=57) (o) | | | |
| --- | --- | --- | --- | --- | --- | --- | --- | --- |
|  | Beta div | Shannon index | Species richness | FCT types | Beta div | Shannon index | Species richness | FCT types |
| Secretor status |  |  | **x (-)** |  |  |  |  |  |
| Total HMO concentration |  | x (-) | **x (-)** | x |  |  |  | o |
| HMO diversity |  |  |  |  |  |  |  |  |
| HMO-bound fucose | x |  | **x (-)** | x |  |  |  |  |
| HMO-bound sialic acid |  |  | **x (+)** |  |  |  |  |  |
| 2’FL |  | x (-) | **x (-)** |  |  |  |  | o |
| 3FL |  |  | x (-) | x |  |  |  | o |
| LNnT | x |  |  |  |  |  |  |  |
| 3’SL |  |  |  |  |  |  |  |  |
| DFLac |  |  |  |  |  |  |  |  |
| 6’SL |  |  |  |  |  |  |  | o |
| LNT |  |  |  |  |  |  |  |  |
| LNFP I |  |  |  | x | x |  |  | **x** |
| LNFP II |  |  |  | x |  |  |  | x |
| LNFP III |  |  |  | x | x |  |  | **x** |
| LSTb |  |  |  | x |  |  |  | xo |
| LSTc |  |  |  |  |  |  |  |  |
| DFLNT |  |  |  |  | o |  |  | o |
| LNH |  |  |  | x |  |  |  | **x** |
| DSLNT |  |  |  |  | x |  |  | x |
| FLNH |  |  |  |  |  |  |  |  |
| DFLNH |  |  |  |  | x |  |  |  |
| FDSLNH | x | x (+) | **x (+)** | x |  |  |  | x |
| DSLNH |  |  |  |  |  |  |  |  |


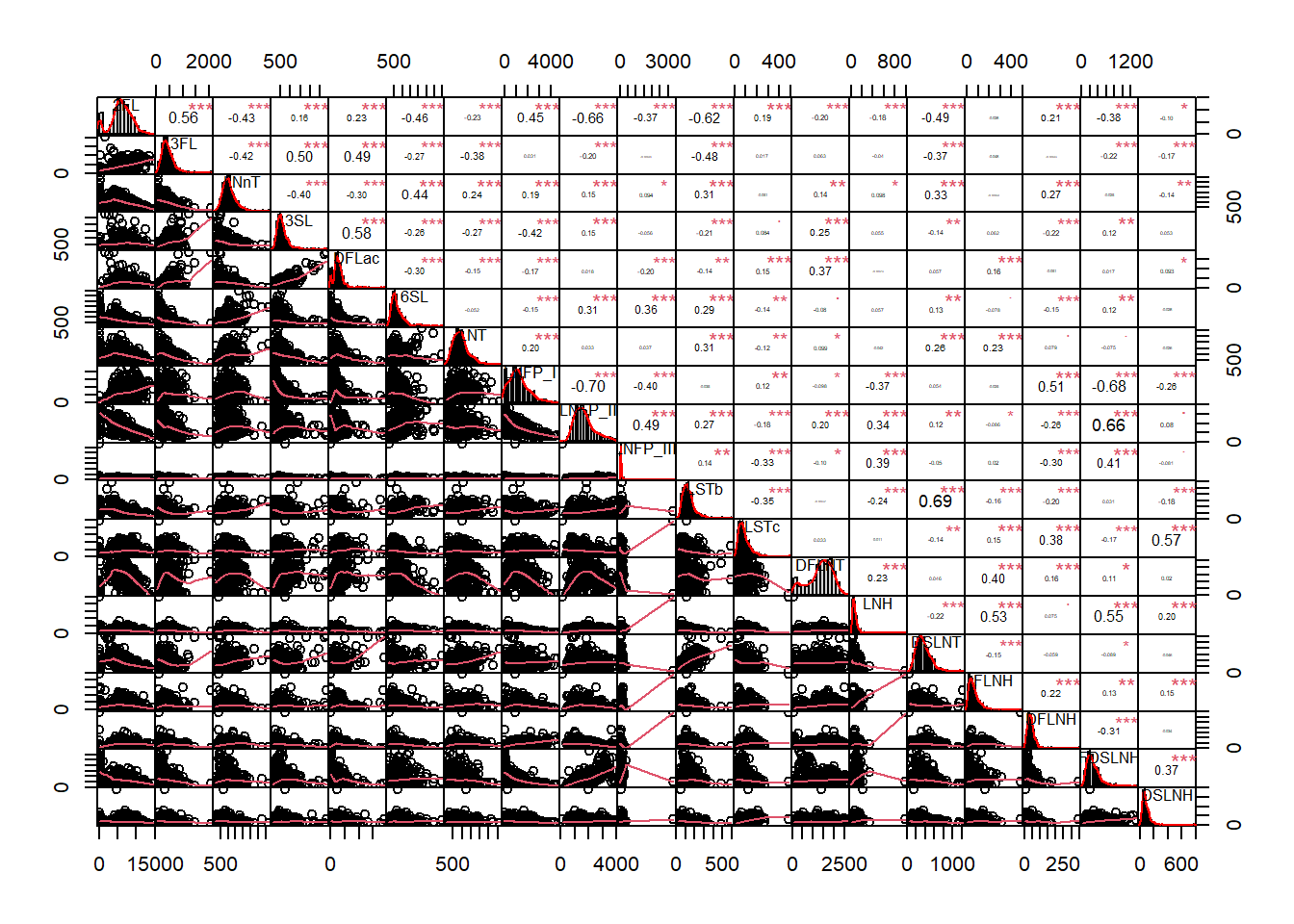


**Figure 5**. Spearman correlation between individual HMO variables and histogram of the HMO distribution. Pairwise correlations were computed using the chart.Correlation function from the PerformanceAnalytics R package (version 2.0.4). The diagonal panels display the distribution of each HMO variable. Below the diagonal, bivariate scatter plots with fitted trend lines are shown. Above the diagonal, Spearman correlation coefficients are presented along with significance levels indicated by stars: p-values: (0.001, 0.01, 0.05, 0.1, 1), symbols: (“***”, “**”, “*”, “.” , “ ”). HMO variables in the diagonal panel: 2’-fucosyllactose (2’FL), 3-fucosyllactose (3FL), lacto-N-neotetraose (LNnT), 3’-sialyllactose (3’SL), difucosyllactose (DFlac), 6’-sialyllactose (6’SL), lacto-N-tetraose (LNT), lacto-Nfucopentaose (LNFP) I, LNFP II, LNFP III, sialyl-LNT (LST) b, LSTc, difucosyllacto-LNT (DFLNT), lacto-N-hexaose (LNH), disialyllacto-Ntetraose (DSLNT), fucosyllacto-Nhexaose (FLNH), difucosyllacto-N-hexaose (DFLNH), fucodisialyllacto-N-hexaose (FDSLNH) and disialyllacto-N-hexaose (DSLNH)

**Table 8.** Association of z-score transformed HMO concentrations ^a^ with FCTs in 3-month-old (n=517) and 13-month-old (n=522) infants assessed with covariate-adjusted multinomial logistic regression models. An OR greater than 1 signifies increased odds of being classified in the first FCT category relative to the reference category listed second. Bolded values represent BH-corrected statistical significance (p<0.05).

|  | **FCT types 3-month**  **OR (95 %CI)** ^b^ | | | **FCT types 13-month**  **OR (95 %CI)** | | | | | |
| --- | --- | --- | --- | --- | --- | --- | --- | --- | --- |
| **HMO** | **2 vs 1** | **3 vs 1** | **3 vs 2** | **2 vs 1** | **3 vs 1** | **4 vs 1** | **3 vs 2** | **4 vs 2** | **4 vs 3** |
| **Secretor**  **(yes)** | 1.91  (0.86-4.23) | 0.57  (0.28-1.14) | 0.30  (0.13-0.66) | 1.39  (0.65-3.01) | 1.68  (0.70-4.03) | 0.75  (0.36-1.57) | 1.20  (0.47-3.06) | 0.54  (0.23-1.25) | 0.45  (0.18-1.14) |
| **Diversity** | 0.92  (0.80-1.06) | 0.97  (0.83-1.14) | 1.05  (0.89-1.24) | 0.94  (0.80-1.09) | 0.86  (0.73-1.02) | 0.95  (0.80-1.12) | 0.92  (0.78-1.1) | 1.01  (0.85-1.21) | 1.10  (0.91-1.32) |
| **Total HMO concentration** | 1.22  (0.97-1.53) | 0.88  (0.7-1.09) | **0.72**  **(0.56-0.91)** | 1.14  (0.9-1.45) | 1.28  (0.98-1.68) | 0.95  (0.75-1.20) | 1.12  (0.84-1.49) | 0.83  (0.64-1.08) | 0.74  (0.55-1.00) |
| **HMO-bound sialic acid** | 0.82  (0.66-1.02) | 0.94  (0.75-1.2) | 1.15  (0.90-1.46) | 0.85  (0.67-1.08) | 0.80  (0.62-1.04) | 1.04  (0.82-1.32) | 0.94  (0.71-1.24) | 1.23  (0.94-1.60) | 1.31  (0.98-1.74) |
| **HMO-bound fucose** | 1.19  (0.95-1.48) | 0.87  (0.70-1.09) | **0.74**  **(0.58-0.94)** | 1.09  (0.86-1.38) | 1.19  (0.91-1.56) | 0.89  (0.70-1.12) | 1.10  (0.83-1.46) | 0.82  (0.63-1.06) | 0.74  (0.56-0.99) |
| **2’FL** | 1.20  (0.97-1.48) | 0.92  (0.73-1.17) | 0.77  (0.60-0.98) | 1.12  (0.89-1.42) | 1.26  (0.98-1.63) | 0.95  (0.75-1.22) | 1.12  (0.86-1.46) | 0.85  (0.65-1.11) | 0.76  (0.57-1.00) |
| **3FL** | 1.10  (0.89-1.36) | 0.99  (0.78-1.27) | 0.90  (0.72-1.14) | 1.09  (0.87-1.38) | 1.14  (0.89-1.46) | 0.81  (0.61-1.07) | 1.04  (0.82-1.33) | 0.74  (0.55-0.99) | 0.71  (0.52-0.96) |
| **LNnT** | 1.03  (0.84-1.27) | 1.05  (0.83-1.33) | 1.02  (0.80-1.30) | 1.10  (0.87-1.38) | 0.96  (0.74-1.23) | 1.03  (0.80-1.32) | 0.87  (0.67-1.13) | 0.94  (0.72-1.22) | 1.07  (0.81-1.42) |
| **3’SL** | 0.99  (0.81-1.21) | 0.79  (0.6-1.04) | 0.80  (0.61-1.04) | 0.89  (0.70-1.12) | 1.00  (0.79-1.26) | 0.86  (0.65-1.12) | 1.13  (0.87-1.45) | 0.96  (0.72-1.29) | 0.85  (0.64-1.15) |
| **DFLac** | 0.97  (0.79-1.20) | 0.72  (0.54-0.96) | 0.74  (0.56-0.98) | 0.94  (0.75-1.18) | 0.94  (0.73-1.21) | 0.91  (0.70-1.19) | 1.00  (0.77-1.29) | 0.97  (0.73-1.28) | 0.97  (0.72-1.31) |
| **6’SL** | 0.85  (0.68-1.06) | 1.09  (0.87-1.36) | 1.29  (1-1.65) | 1.05  (0.83-1.32) | 0.88  (0.67-1.16) | 1.11  (0.88-1.40) | 0.84  (0.64-1.12) | 1.06  (0.82-1.37) | 1.26  (0.94-1.68) |
| **LNT** | 1.01  (0.82-1.24) | 0.99  (0.78-1.25) | 0.98  (0.77-1.24) | 1.08  (0.85-1.37) | 1.08  (0.84-1.38) | 1.22  (0.96-1.55) | 1.00  (0.77-1.29) | 1.13  (0.87-1.47) | 1.13  (0.87-1.48) |
| **LNFP I** | 1.13  (0.92-1.38) | 0.90  (0.71-1.15) | 0.80  (0.63-1.02) | 1.11  (0.87-1.42) | 1.38  (1.08-1.76) | 1.31  (1.01-1.68) | 1.24  (0.96-1.59) | 1.17  (0.9-1.54) | 0.95  (0.73-1.23) |
| **LNFP II** | 0.86  (0.69-1.06) | 1.14  (0.91-1.43) | 1.33  (1.05-1.69) | 0.91  (0.72-1.14) | 0.74  (0.57-0.95) | 0.87  (0.68-1.11) | 0.81  (0.62-1.06) | 0.96  (0.73-1.25) | 1.18  (0.88-1.57) |
| **LNFP III** | 0.53  (0.25-1.14) | 1.86  (0.86-4.02) | **3.53**  **(1.47-8.45)** | 1.33  (0.65-2.74) | 0.51  (0.21-1.23) | 0.77  (0.34-1.74) | 0.39  (0.16-0.95) | 0.58  (0.24-1.38) | 1.50  (0.56-4.02) |
| **LSTb** | 0.87  (0.71-1.07) | 0.83  (0.65-1.07) | 0.96  (0.74-1.24) | 0.86  (0.67-1.1) | 0.99  (0.78-1.26) | 1.20  (0.95-1.52) | 1.15  (0.88-1.51) | 1.40  (1.07-1.84) | 1.22  (0.94-1.57) |
| **LSTc** | 1.19  (0.96-1.47) | 0.97  (0.75-1.25) | 0.82  (0.64-1.04) | 0.99  (0.78-1.25) | 1.03  (0.81-1.31) | 0.93  (0.72-1.22) | 1.04  (0.81-1.34) | 0.94  (0.71-1.25) | 0.91  (0.68-1.21) |
| **DFLNT** | 1.01  (0.82-1.25) | 0.91  (0.72-1.16) | 0.9  (0.71-1.14) | 0.95  (0.75-1.21) | 0.91  (0.71-1.17) | 0.79  (0.62-1.01) | 0.96  (0.74-1.24) | 0.83  (0.64-1.08) | 0.87  (0.66-1.14) |
| **LNH** | 0.97  (0.72-1.3) | 1.21  (0.89-1.65) | 1.25  (0.92-1.7) | 1.18  (0.89-1.57) | 0.76  (0.53-1.09) | 0.78  (0.54-1.13) | 0.64  (0.44-0.93) | 0.66  (0.45-0.97) | 1.03  (0.67-1.58) |
| **DSLNT** | 0.94  (0.76-1.15) | 0.83  (0.65-1.06) | 0.89  (0.70-1.13) | 0.87  (0.69-1.11) | 0.99  (0.77-1.26) | 1.12  (0.88-1.43) | 1.13  (0.87-1.47) | 1.29  (0.99-1.68) | 1.14  (0.87-1.49) |
| **FLNH** | 0.94  (0.76-1.17) | 1.02  (0.81-1.29) | 1.09  (0.86-1.37) | 1.02  (0.82-1.28) | 0.92  (0.71-1.19) | 0.85  (0.65-1.11) | 0.90  (0.69-1.17) | 0.83  (0.63-1.1) | 0.92  (0.68-1.25) |
| **DFLNH** | 1.11  (0.87-1.42) | 1.12  (0.86-1.46) | 1.01  (0.82-1.25) | 1.06  (0.84-1.34) | 1.04  (0.79-1.36) | 0.92  (0.68-1.24) | 0.98  (0.75-1.27) | 0.87  (0.64-1.17) | 0.89  (0.64-1.23) |
| **FDSLNH** | 0.83  (0.66-1.04) | 1.15  (0.92-1.44) | **1.39**  **(1.09-1.78)** | 0.90  (0.72-1.13) | 0.70  (0.52-0.93) | 0.97  (0.76-1.23) | 0.78  (0.58-1.05) | 1.08  (0.83-1.41) | 1.38  (1.01-1.9) |
| **DSLNH** | 0.97  (0.79-1.19) | 0.95  (0.75-1.21) | 0.98  (0.77-1.26) | 0.91  (0.71-1.16) | 0.98  (0.78-1.23) | 0.93  (0.7-1.22) | 1.08  (0.83-1.39) | 1.02  (0.75-1.38) | 0.95  (0.71-1.27) |
| ^a^ To support interpretation of the z-score standardized HMO concentration results, Supplementary 1 Tables 1 and 3 report the median, mean, and standard deviation (SD) values for each HMO. | | | | | | | | | |
| ^b^ Odds ratios (ORs) and corresponding 95% confidence intervals (CIs) are reported per 1 standard deviation increase in z-score standardized HMO concentrations. For maternal secretor status and HMO diversity, ORs (95% CIs) are reported based on unstandardized values. | | | | | | | | | |

**Table 9.** Association of z-score transformed HMO concentrations ^a^ with Shannon diversity and Observed richness in 3-month-old (n=517) and 13-month-old (n=522) infants assessed with covariate-adjusted linear model. Bolded values represent BH-corrected statistical significance.

|  | **Alpha diversity 3-month-old (n=517)** | | **Alpha diversity 13-month-old (n=522)** | |
| --- | --- | --- | --- | --- |
|  | **Shannon diversity**  **b (95 %CI) ^b^** | **Observed richness**  **b (95 %CI)** | **Shannon** **diversity**  **b (95 %CI)** | **Observed richness**  **b (95 %CI)** |
| **Secretor**  **(yes)** | **-0.239 (-0.397,-0.081)** | -2.186 (-7.155,2.783) | -0.111 (-0.248,0.026) | **-11.103(-19.737,**  **-2.469)** |
| **Diversity** | 0.033 (0.000,0.066) | 0.766 (-0.269,1.801) | 0.020 (-0.009,0.048) | 1.144 (-0.659,2.946) |
| **Total HMO concentration** | **-0.074 (-0.122,-0.026)** | -0.606 (-2.124,0.912) | -0.047 (-0.090,-0.005) | **-4.108 (-6.786,-1.429)** |
| **HMO-bound sialic acid** | **0.094 (0.046,0.142)** | **2.688 (1.185,4.192)** | 0.040 (-0.003,0.083) | **4.148 (1.466,6.831)** |
| **HMO-bound fucose** | **-0.068 (-0.117,-0.020)** | -0.606 (-2.123,0.912) | -0.040 (-0.082,0.003) | **-3.573 (-6.258,-0.888)** |
| **2FL** | -**0.086 (-0.134,-0.038)** | -1.286 (-2.801,0.229) | -0.049 (-0.091,-0.006) | **-3.693 (-6.373,-1.012)** |
| **3FL** | -0.002 (-0.051,0.047) | 1.372 (-0.143,2.886) | -0.014 (-0.056,0.029) | -2.749 (-5.436,-0.061) |
| **LNnT** | 0.008 (-0.041,0.057) | -0.376 (-1.908,1.156) | -0.002 (-0.045,0.041) | -1.678 (-4.389,1.033) |
| **3SL** | 0.050 (0.001,0.098) | **2.601 (1.097,4.105)** | -0.002 (-0.045,0.041) | -0.611 (-3.314,2.092) |
| **DFLac** | 0.034 (-0.015,0.082) | **2.475 (0.971,3.979)** | 0.017 (-0.026,0.060) | 0.957 (-1.740,3.655) |
| **6SL** | **0.067 (0.019,0.116)** | 1.503 (-0.011,3.016) | 0.012 (-0.030,0.055) | 2.258 (-0.430,4.947) |
| **LNT** | -0.030 (-0.079,0.018) | -0.896 (-2.412,0.619) | 0.003 (-0.039,0.046) | 0.334 (-2.363,3.031) |
| **LNFP_I** | -0.044 (-0.092,0.005) | -0.613 (-2.129,0.904) | -0.019 (-0.061,0.024) | -1.917 (-4.608,0.774) |
| **LNFP_II** | **0.064 (0.016,0.112)** | 0.358 (-1.162,1.877) | 0.036 (-0.006,0.079) | 2.162 (-0.528,4.853) |
| **LNFP_III** | 0.021 (-0.028,0.070) | 0.210 (-1.315,1.735) | 0.002 (-0.041,0.045) | 0.378 (-2.332,3.087) |
| **LSTb** | **0.101 (0.053,0.149)** | **3.096 (1.600,4.592)** | 0.025 (-0.017,0.068) | 2.196 (-0.494,4.885) |
| **LSTc** | -0.047 (-0.096,0.002) | -0.828 (-2.364,0.709) | -0.030 (-0.073,0.013) | -0.682 (-3.411,2.047) |
| **DFLNT** | -0.015 (-0.064,0.034) | -0.275 (-1.802,1.252) | -0.015 (-0.058,0.028) | -1.624 (-4.340,1.091) |
| **LNH** | -0.016 (-0.065,0.033) | -0.986 (-2.506,0.534) | 0.012 (-0.030,0.055) | 1.616 (-1.084,4.316) |
| **DSLNT** | 0.049 (0.001,0.098) | 1.737 (0.225,3.250) | 0.032 (-0.010,0.075) | 2.620 (-0.075,5.314) |
| **FLNH** | -0.020 (-0.069,0.029) | -0.613 (-2.128,0.902) | -0.006 (-0.048,0.037) | 1.315 (-1.377,4.007) |
| **DFLNH** | -0.026 (-0.075,0.024) | -0.462 (-1.995,1.071) | 0.005 (-0.038,0.048) | -0.989 (-3.709,1.730) |
| **FDSLNH** | 0.027 (-0.021,0.076) | -0.216 (-1.734,1.302) | 0.045 (0.002,0.087) | **3.858 (1.178,6.537)** |
| **DSLNH** | -0.022 (-0.072,0.027) | -0.258 (-1.789,1.273) | -0.008 (-0.052,0.035) | 0.589 (-2.138,3.316) |
| ^a^ To support interpretation of the z-score standardized HMO concentration results, Supplementary 1 Tables 1 and 3 report the median, mean, and standard deviation (SD) values for each HMO. | | | | |
| ^b^ The effect estimates were expressed as (b) and the corresponding 95% confidence interval (CI) per 1 standard deviation increase in z-score transformed HMO concentration. For maternal secreor staus and HMO Diversity the b (95 %CI) were expressed for non-standardized values. | | | | |

**Table 10.** Sensitivity analyses of breastfeeding status. Association of z-score transformed HMO concentrations a with FCTs in exclusively BF 3-month-old (n= 259) and the whole cohort of 13-month-old (n=522) infants assessed with covariate-adjusted multinomial logistic regression models. The covariates used in the models were: birth mode (vaginal or caesarean section delivery), parity (primiparous or multiparous), and maternal pre-pregnancy BMI. In 13-month timepoint also the current HMO exposure and the duration of time that has passed since any HMO exposure was controlled with categorical variable (current breastfeeding, <1 month since cessation of BF, 1–3 months, 3–6 months, >6 months, unknown). An OR greater than 1 signifies increased odds of being classified in the first FCT category relative to the reference category listed second. Star (*) indicates statistical significance and bolded values represent BH-corrected statistical significance (p<0.05).

|  | **FCT types 3-month**  **OR (95 %CI)** ^b^ | | | **FCT types 13-month**  **OR (95 %CI)** | | | | | |
| --- | --- | --- | --- | --- | --- | --- | --- | --- | --- |
| **HMO** | **2 vs 1** | **3 vs 1** | **3 vs 2** | **2 vs 1** | **3 vs 1** | **4 vs 1** | **3 vs 2** | **4 vs 2** | **4 vs 3** |
| **Secretor**  **(yes)** | 1.69(0.52–5.51) | 0.38 (0.14–1.04) | 0.23 (0.07–0.70) * | 1.28 (0.59–2.79) | 1.66 (0.69–4.04) | 0.75 (0.36–1.58) | 1.3 (0.5–3.36) | 0.59 (0.25–1.37) | 0.45 (0.18–1.15) |
| **Diversity** | 0.81(0.66–1.00) * | 0.84 (0.66–1.06) | 1.03 (0.81–1.31) | 0.94 (0.8–1.1) | 0.86 (0.73–1.02) | 0.95 (0.8–1.12) | 0.92 (0.77–1.1) | 1.01 (0.84–1.21) | 1.1 (0.91–1.33) |
| **Total HMO concentration** | 1.24(0.89–1.73) | 0.86 (0.63–1.19) | 0.69 (0.49–0.98) * | 1.12 (0.88–1.42) | 1.27 (0.97–1.67) | 0.95 (0.75–1.2) | 1.14 (0.85–1.52) | 0.84 (0.65–1.1) | 0.74 (0.55–1) * |
| **HMO-bound sialic acid** | 0.70(0.51–0.97) * | 0.99 (0.70–1.39) | 1.41 (0.98–2.02) | 0.86 (0.68–1.1) | 0.8 (0.62–1.04) | 1.04 (0.82–1.32) | 0.93 (0.7–1.23) | 1.2 (0.92–1.58) | 1.3 (0.98–1.72) |
| **HMO-bound fucose** | 1.29(0.92–1.80) | 0.84 (0.61–1.16) | 0.65 (0.46–0.93) * | 1.06 (0.84–1.35) | 1.19 (0.91–1.56) | 0.89 (0.7–1.12) | 1.12 (0.84–1.49) | 0.83 (0.64–1.09) | 0.74 (0.56–0.99) * |
| **2’FL** | 1.33(0.98–1.81) | 0.98 (0.70–1.39) | 0.74 (0.52–1.05) | 1.1 (0.87–1.39) | 1.26 (0.97–1.62) | 0.95 (0.74–1.22) | 1.14 (0.87–1.5) | 0.87 (0.66–1.13) | 0.76 (0.57–1.01) |
| **3FL** | 1.33(0.94–1.87) | 1.26 (0.85–1.88) | 0.95 (0.64–1.41) | 1.09 (0.86–1.37) | 1.14 (0.89–1.46) | 0.81 (0.61–1.07) | 1.05 (0.82–1.34) | 0.74 (0.55–1) * | 0.71 (0.52–0.96) * |
| **LNnT** | 0.83(0.62–1.12) | 0.85 (0.60–1.21) | 1.03 (0.71–1.49) | 1.09 (0.86–1.38) | 0.96 (0.74–1.24) | 1.04 (0.81–1.34) | 0.88 (0.67–1.15) | 0.95 (0.73–1.25) | 1.08 (0.82–1.44) |
| **3’SL** | 0.95(0.65–1.38) | 0.73 (0.44–1.22) | 0.77 (0.45–1.31) | 0.89 (0.7–1.13) | 1 (0.79–1.27) | 0.85 (0.65–1.12) | 1.13 (0.87–1.46) | 0.96 (0.71–1.29) | 0.85 (0.63–1.14) |
| **DFLac** | 1.09(0.72–1.66) | **0.50 (0.28–0.88)** | **0.46 (0.26–0.82)** | 0.94 (0.75–1.19) | 0.95 (0.74–1.22) | 0.91 (0.7–1.18) | 1.01 (0.78–1.32) | 0.96 (0.73–1.28) | 0.95 (0.71–1.28) |
| **6’SL** | 0.87(0.62–1.23) | 1.24 (0.88–1.74) | 1.42 (0.99–2.04) | 1.07 (0.84–1.36) | 0.88 (0.67–1.15) | 1.1 (0.87–1.39) | 0.82 (0.62–1.09) | 1.03 (0.8–1.33) | 1.26 (0.94–1.67) |
| **LNT** | 0.96(0.71–1.29) | 0.90 (0.63–1.29) | 0.95 (0.66–1.35) | 1.08 (0.85–1.38) | 1.06 (0.83–1.37) | 1.2 (0.94–1.53) | 0.98 (0.75–1.28) | 1.11 (0.85–1.45) | 1.13 (0.86–1.48) |
| **LNFP I** | 1.06(0.79–1.43) | 0.83 (0.56–1.21) | 0.78 (0.53–1.14) | 1.11 (0.87–1.42) | 1.36 (1.06–1.74) * | 1.29 (1–1.66) * | 1.22 (0.95–1.58) | 1.16 (0.89–1.53) | 0.95 (0.73–1.24) |
| **LNFP II** | 0.77(0.56–1.06) | 1.26 (0.90–1.75) | **1.64 (1.14–2.35)** | 0.91 (0.72–1.15) | 0.75 (0.58–0.98) * | 0.88 (0.68–1.13) | 0.83 (0.63–1.09) | 0.96 (0.73–1.27) | 1.16 (0.87–1.56) |
| **LNFP III** | 0.32(0.11–0.96) * | 2.39 (0.90–6.32) | **7.45 (2.23–24.87)** | 1.43 (0.68–3.01) | 0.5 (0.2–1.22) | 0.77 (0.34–1.77) | 0.35 (0.14–0.88) * | 0.54 (0.22–1.32) | 1.57 (0.57–4.27) |
| **LSTb** | 0.69(0.49–0.97) * | 0.85 (0.59–1.24) | 1.23 (0.83–1.84) | 0.87 (0.67–1.12) | 0.98 (0.77–1.26) | 1.19 (0.94–1.51) | 1.13 (0.86–1.5) | 1.37 (1.04–1.81) * | 1.21 (0.93–1.57) |
| **LSTc** | 1.39(0.99–1.95) | 0.72 (0.45–1.13) | **0.52 (0.32–0.82)** | 1(0.79–1.26) | 1.04 (0.81–1.32) | 0.94 (0.72–1.23) | 1.04 (0.81–1.34) | 0.95 (0.71–1.25) | 0.91 (0.68–1.21) |
| **DFLNT** | 0.99(0.73–1.35) | **0.65 (0.47–0.92)** | **0.66 (0.46–0.94)** | 0.94 (0.73–1.2) | 0.91 (0.7–1.17) | 0.79 (0.62–1.01) | 0.97 (0.74–1.26) | 0.84 (0.64–1.11) | 0.87 (0.66–1.15) |
| **LNH** | 1.14(0.78–1.67) | 1.15 (0.74–1.79) | 1.01 (0.65–1.57) | 1.17 (0.88–1.56) | 0.76 (0.52–1.1) | 0.81 (0.56–1.17) | 0.65 (0.44–0.94) * | 0.69 (0.47–1.01) | 1.07 (0.69–1.65) |
| **DSLNT** | **0.67(0.48–0.94)** | **0.65 (0.44–0.96)** | 0.96 (0.64–1.44) | 0.87 (0.68–1.11) | 0.98 (0.76–1.25) | 1.1 (0.86–1.41) | 1.13 (0.86–1.48) | 1.27 (0.97–1.67) | 1.13 (0.86–1.48) |
| **FLNH** | 1.00 (0.73–1.37) | 0.91 (0.61–1.34) | 0.91 (0.61–1.35) | 1.04 (0.83–1.31) | 0.91 (0.71–1.18) | 0.86 (0.66–1.12) | 0.88 (0.67–1.15) | 0.83 (0.62–1.09) | 0.94 (0.7–1.27) |
| **DFLNH** | 1.25(0.85–1.84) | 0.93 (0.58–1.47) | 0.74 (0.47–1.16) | 1.06 (0.84–1.33) | 1.03 (0.78–1.35) | 0.93 (0.69–1.25) | 0.97 (0.75–1.26) | 0.88 (0.65–1.18) | 0.9 (0.65–1.25) |
| **FDSLNH** | 0.77(0.56–1.08) | 1.16 (0.86–1.58) | 1.50 (1.07–2.11) * | 0.9 (0.72–1.14) | 0.71 (0.53–0.94) * | 0.99 (0.78–1.26) | 0.78 (0.57–1.06) | 1.1 (0.84–1.44) | 1.41 (1.02–1.94) * |
| **DSLNH** | 1.27(0.86–1.86) | 1.08 (0.69–1.68) | 0.85 (0.55–1.30) | 0.91 (0.71–1.17) | 0.97 (0.78–1.22) | 0.93 (0.71–1.22) | 1.06 (0.82–1.38) | 1.02 (0.76–1.38) | 0.96 (0.72–1.28) |
| ^a^ To support interpretation of the z-score standardized HMO concentration results, Supplementary 1 Tables 1 and 3 report the median, mean, and standard deviation (SD) values for each HMO. | | | | | | | | | |
| ^b^ Odds ratios (ORs) and corresponding 95% confidence intervals (CIs) are reported per 1 standard deviation increase in z-score standardized HMO concentrations. For maternal secretor status and HMO diversity, ORs (95% CIs) are reported based on unstandardized values. | | | | | | | | | |

**Table 11.** Summary table of the associations between individual HMOs and HMO summary measures and gut microbiota beta diversity, alpha diversity (Shannon diversity, observed richness), and FCT types in 3-month-old exclusively BF infants (n=259) and 3-month-old exclusively BF infants receiving milk from secretor mothers (n=234). Beta diversity was studied using stepwise variable selection, followed by covariate adjusted permutational multivariate analysis of variance (PERMANOVA), alpha diversity was studied with covariate-adjusted linear models and FCT analyses was conducted with covariate-adjusted multinomial logistic regression models. The covariates used in the models were: birth mode (vaginal or caesarean section delivery), parity (primiparous or multiparous), and maternal pre-pregnancy BMI. In alpha diversity columns the (+) indicates increasing and (-) decreasing association of the HMO to the diversity metrics. Bolded and underlined values (**X**) represent BH-corrected statistical significance (p<0.05).

|  | 3-month (n=259) | | | | 3-month secretors (n=234) (x) | | | |
| --- | --- | --- | --- | --- | --- | --- | --- | --- |
|  | Beta div | Shannon index | Species richness | FCT types | Beta div | Shannon index | Species richness | FCT types |
| Secretor status |  | **x (-)** |  | x |  |  |  |  |
| Total HMO concentration |  | **x (-)** |  | x |  |  |  | x |
| HMO diversity |  | **x (+)** |  | x |  | x (+) |  |  |
| HMO-bound fucose |  | **x (-)** |  | x |  |  |  |  |
| HMO-bound sialic acid |  | **x (+)** |  | x |  | x (+) |  | **x** |
| 2FL |  | **x (-)** |  |  |  | x (-) |  | x |
| 3FL |  |  |  |  |  |  |  | x |
| LNnT |  | x (+) |  |  |  |  |  |  |
| 3SL |  |  |  |  |  |  |  |  |
| DFLac |  |  |  | **x** |  | x (+) |  |  |
| 6SL |  | **x (+)** |  |  |  |  |  |  |
| LNT |  |  |  |  |  |  |  |  |
| LNFP_I |  |  |  |  |  |  |  |  |
| LNFP_II |  | **x (+)** |  | **x** |  | x (+) |  |  |
| LNFP_III |  |  |  | **x** |  |  |  | x |
| LSTb |  | **x (+)** | x (+) | x |  | x (+) | x (+) | x |
| LSTc |  |  |  | **x** |  |  |  | x |
| DFLNT |  |  |  | **x** |  |  |  |  |
| LNH |  |  |  |  |  |  |  |  |
| DSLNT | x |  |  | **x** | x |  |  | **x** |
| FLNH |  |  |  |  |  |  |  |  |
| DFLNH |  |  |  |  |  |  |  |  |
| FDSLNH |  |  |  | x |  |  |  |  |
| DSLNH |  |  |  |  |  | x (-) |  | x |

**Table 12.** Sensitivity analyses of breastfeeding status. Association of z-score transformed HMO concentrations a with Shannon diversity and Observed richness in exclusively BF 3-month-old (n=259) and in the whole cohort of 13-month-old (n=522) infants assessed with covariate-adjusted linear model. The covariates used in the models were: birth mode (vaginal or caesarean section delivery), parity (primiparous or multiparous), and maternal pre-pregnancy BMI. In 13-month timepoint also the current HMO exposure and the duration of time that has passed since any HMO exposure was controlled with categorical variable (current breastfeeding, <1 month since cessation of BF, 1–3 months, 3–6 months, >6 months, unknown). Bolded values represent BH-corrected statistical significance.

|  | **Alpha diversity exclusively BF 3-month-old (n=259)** | | **Alpha diversity 13-month-old (n=522)** | | |
| --- | --- | --- | --- | --- | --- |
|  | **Shannon diversity**  **b (95 %CI) ^b^** | **Observed richness**  **b (95 %CI)** | **Shannon** **diversity**  **b (95 %CI)** | **Observed richness**  **b (95 %CI)** | |
| **Secretor**  **(yes)** | **-0.290(-0.519;-0.061)** | -1.501(-8.419;5.418) | -0.117(-0.255;0.021) | **-11.324(-19.993;-2.655)** | |
| **Diversity** | **0.070(0.024;0.117)** | 1.129(-0.276;2.534) | 0.020(-0.008;0.049) | 1.187(-0.616;2.990) | |
| **Total HMO concentration** | **-0.101(-0.170;-0.032)** | -0.683(-2.775;1.410) | -0.049(-0.092;-0.006) | **-4.233(-6.916;-1.551)** | |
| **HMO-bound sialic acid** | **0.123(0.053;0.193)** | 1.969(-0.152;4.089) | 0.041(-0.001;0.084) | **4.196(1.513;6.879)** | |
| **HMO-bound fucose** | **-0.103(-0.172;-0.033)** | -0.818(-2.925;1.289) | -0.042(-0.085;0.001) | **-3.696(-6.388;-1.005)** | |
| **2FL** | **-0.129(-0.197;-0.061)** | -1.304(-3.380;0.771) | -0.050(-0.093;-0.008) | **-3.751(-6.431;-1.070)** | |
| **3FL** | -0.057(-0.137;0.024) | 0.388(-2.016;2.792) | -0.016(-0.058;0.027) | -2.945(-5.641;-0.250) | |
| **LNnT** | 0.070(0.002;0.137) | 1.021(-1.014;3.056) | 0.000(-0.043;0.043) | -1.394(-4.114;1.327) | |
| **3SL** | 0.056(-0.037;0.149) | 1.367(-1.415;4.149) | -0.003(-0.046;0.040) | -0.824(-3.543;1.894) | |
| **DFLac** | 0.013(-0.089;0.116) | 1.455(-1.590;4.499) | 0.014(-0.030;0.057) | 0.542(-2.203;3.287) | |
| **6SL** | **0.114(0.041;0.188)** | 2.051(-0.173;4.275) | 0.014(-0.029;0.057) | 2.305(-0.387;4.998) | |
| **LNT** | -0.014(-0.085;0.058) | -1.190(-3.327;0.948) | 0.004(-0.039;0.046) | 0.254(-2.444;2.951) | |
| **LNFP_I** | -0.062(-0.135;0.010) | 0.229(-1.953;2.411) | -0.019(-0.062;0.024) | -2.042(-4.742;0.658) | |
| **LNFP_II** | **0.110(0.040;0.180)** | 0.296(-1.829;2.422) | 0.036(-0.007;0.079) | 2.235(-0.462;4.932) | |
| **LNFP_III** | 0.140(-0.079;0.358) | 1.261(-5.276;7.799) | 0.002(-0.041;0.045) | 0.258(-2.451;2.967) | |
| **LSTb** | **0.130(0.054;0.205)** | 3.056(0.776;5.335) | 0.025(-0.018;0.068) | 1.946(-0.774;4.667) | |
| **LSTc** | -0.065(-0.147;0.018) | 0.179(-2.307;2.664) | -0.031(-0.074;0.013) | -0.756(-3.488;1.975) | |
| **DFLNT** | 0.011(-0.060;0.082) | 0.123(-1.989;2.236) | -0.015(-0.058;0.028) | -1.507(-4.240;1.227) | |
| **LNH** | -0.030(-0.121;0.061) | -0.290(-3.009;2.430) | 0.013(-0.030;0.056) | 1.774(-0.927;4.474) | |
| **DSLNT** | 0.077(0.000;0.153) | 1.384(-0.907;3.674) | 0.032(-0.011;0.075) | 2.441(-0.278;5.160) | |
| **FLNH** | -0.055(-0.131;0.022) | -0.416(-2.714;1.882) | -0.005(-0.048;0.038) | 1.338(-1.355;4.031) | |
| **DFLNH** | -0.079(-0.166;0.009) | 0.043(-2.586;2.671) | 0.007(-0.036;0.050) | -0.772(-3.496;1.953) | |
| **FDSLNH** | 0.042(-0.025;0.109) | -0.055(-2.052;1.943) | 0.049(0.006;0.092) | **4.440(1.722;7.158)** | |
| **DSLNH** | -0.046(-0.135;0.044) | -0.681(-3.354;1.992) | -0.007(-0.051;0.036) | 0.676(-2.047;3.400) | |
| ^a^ To support interpretation of the z-score standardized HMO concentration results, Supplementary 1 Tables 1 and 3 report the median, mean, and standard deviation (SD) values for each HMO. | | | | |  |
| ^b^ The effect estimates were expressed as (b) and the corresponding 95% confidence interval (CI) per 1 standard deviation increase in z-score transformed HMO concentration. For maternal secreor staus and HMO Diversity the b (95 %CI) were expressed for non-standardized values. | | | | |  |


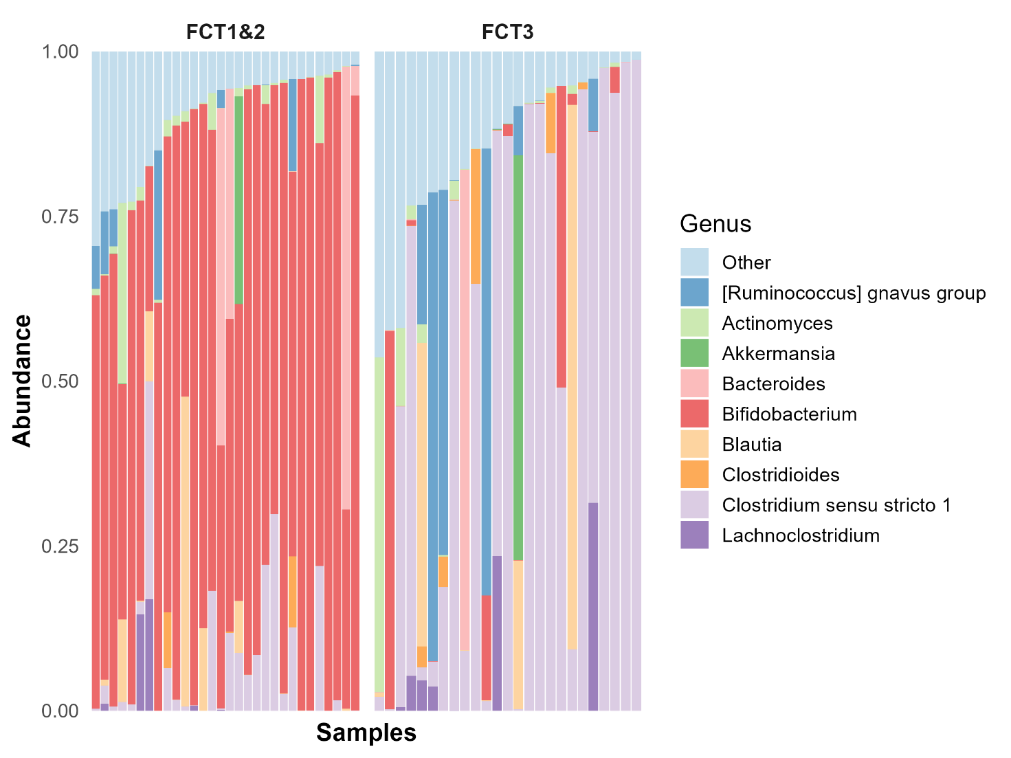


**Figure 6**. Relative abundance of top to 10 bacterial genera of cesarean born infants in FCT1&2 (n=30) and FCT3 (n=25) per sample in 3-month timepoint.

**Table 13**. Differences in descriptive characteristics of the cesarean born infants between binary DMM clusters (FCT1&2 and FCT3) in 3-month-old infants. Sample sizes and percentages are given for categorical variables and means with standard deviations for continuous variables. Continuous variables tested with Wilcoxon test due to non-normal distributions and categorical variables tested with Fisher's exact test. Bolded values represent statistically significant (p<0.05) differences between the FCTs.

| **Variable** | **level** | **Overall** | **3M-FCT1&2** | **3M-FCT3** | **p** |
| --- | --- | --- | --- | --- | --- |
| n |  | 55 | 30 | 25 |  |
| Birth weight (mean (SD)) |  | 3.55 (0.54) | 3.65 (0.48) | 3.42 (0.60) | 0.141 |
| Duration of pregnancy (mean (SD)) |  | 40.02 (1.36) | 39.91 (1.46) | 40.15 (1.24) | 0.482 |
| Mothers age (mean (SD)) |  | 31.87 (4.21) | 31.34 (3.64) | 32.50 (4.80) | 0.597 |
| Infant age (mean (SD)) |  | 2.79 (0.41) | 2.84 (0.48) | 2.73 (0.32) | 0.200 |
| Number of sequencing reads (mean (SD)) |  | 17913 (7932) | 18499 (7023) | 17209 (9001) | 0.574 |
| Pre-pregnency BMI (mean (SD)) |  | 25.07 (5.77) | 25.34 (5.52) | 24.75 (6.15) | 0.434 |
| Sex (%) | 1 | 29 (52.7) | 18 (60.0) | 11 (44.0) | 0.285 |
|  | 2 | 26 (47.3) | 12 (40.0) | 14 (56.0) |  |
| Maternal antibiotics (%) | 0 | 51 (92.7) | 28 (93.3) | 23 (92.0) | 1.000 |
|  | 1 | 4 (7.3) | 2 (6.7) | 2 (8.0) |  |
| Infant antibiotics (%) | 0 | 45 (81.8) | 25 (83.3) | 20 (80.0) | 1.000 |
|  | 1 | 10 (18.2) | 5 (16.7) | 5 (20.0) |  |
| Previous deliveries (%) | >0 | 19 (34.5) | 14 (46.7) | 5 (20.0) | **0.049** |
|  | no previous deliveries | 36 (65.5) | 16 (53.3) | 20 (80.0) |  |
| Secretor (%) | 0 | 10 (18.2) | 2 (6.7) | 8 (32.0) | **0.032** |
|  | 1 | 45 (81.8) | 28 (93.3) | 17 (68.0) |  |
| Breastfeeding status (%) | exclusive | 23 (41.8) | 15 (50.0) | 8 (32.0) | 0.467 |
|  | no | 6 (10.9) | 2 (6.7) | 4 (16.0) |  |
|  | partial | 17 (30.9) | 8 (26.7) | 9 (36.0) |  |
|  | unknown | 9 (16.4) | 5 (16.7) | 4 (16.0) |  |

**Table 14.** The relationship between maternal secretor status and HMO composition and FCTs in a subcohort of cesarean-born infants (n=55), assessed with covariate adjusted logistic regression model. A binary outcome variable was created to compare cesarean born infants assigned to FCT3 (n= 25) versus those in FCT1 or FCT2 combined (n=30). An OR greater than 1 signifies increased odds of being classified in the FCT3 relative to the reference category FCT1 or FCT2 combined.

| **HMO** | **OR** | **CI lower** | **CI upper** |
| --- | --- | --- | --- |
| Secretor status | 0,200 | 0,027 | 0,983 |
| Total HMO concentration | 0,633 | 0,359 | 1,023 |
| HMO-bound sialic acid | 0,806 | 0,469 | 1,312 |
| HMO-bound fucose | 0,656 | 0,368 | 1,092 |
| HMO diversity | 1,159 | 0,782 | 1,748 |
| 2’FL | 0,657 | 0,378 | 1,089 |
| 3FL | 0,974 | 0,645 | 1,404 |
| LNnT | 0,845 | 0,435 | 1,613 |
| 3’SL | 0,746 | 0,363 | 1,255 |
| DFLac | 0,869 | 0,539 | 1,270 |
| 6’SL | 0,921 | 0,461 | 1,745 |
| LNT | 1,186 | 0,693 | 2,107 |
| LNFP I | 0,893 | 0,482 | 1,628 |
| LNFP II | 1,220 | 0,719 | 2,131 |
| LNFP III | 14,500 | 1,363 | 338,381 |
| LSTb | 0,854 | 0,411 | 1,676 |
| LSTc | 1,153 | 0,754 | 1,980 |
| DFLNT | 0,864 | 0,500 | 1,489 |
| LNH | 1,484 | 0,925 | 3,819 |
| DSLNT | 0,903 | 0,555 | 1,392 |
| FLNH | 1,316 | 0,878 | 2,331 |
| DFLNH | 1,128 | 0,857 | 1,622 |
| FDSLNH | 0,974 | 0,635 | 1,507 |
| DSLNH | 0,853 | 0,483 | 1,468 |

**Table 15.** Differences in descriptive characteristics of the cesarean born infants between FCTs in 13-month-old infants. Sample sizes and percentages are given for categorical variables and means with standard deviations for continuous variables. Continuous variables tested with Kruskal-Wallis test due to non-normal distributions and categorical variables tested with Fisher's exact test. Bolded values represent statistically significant (p<0.05) differences between the FCTs.

| **Variable** | **level** | **Overall** | **13M-FCT1** | **13M-FCT2** | **13M-FCT3** | **13M-FCT4** | **p** |
| --- | --- | --- | --- | --- | --- | --- | --- |
| n |  | 51 | 12 | 27 | 6 | 6 |  |
| Birth weight (mean (SD)) |  | 3.53 (0.56) | 3.59 (0.69) | 3.60 (0.47) | 3.51 (0.43) | 3.10 (0.68) | 0.371 |
| Duration of pregnancy (mean (SD)) |  | 39.97 (1.33) | 39.49 (1.28) | 40.51 (1.25) | 39.45 (1.36) | 39.05 (0.88) | **0.032** |
| Mothers age (mean (SD)) |  | 31.86 (4.30) | 30.91 (4.36) | 31.61 (4.22) | 32.09 (4.48) | 34.69 (4.25) | 0.322 |
| Infant age (mean (SD)) |  | 13.58 (0.66) | 13.89 (0.50) | 13.50 (0.75) | 13.42 (0.66) | 13.40 (0.38) | 0.237 |
| Number of sequencing reads (mean (SD)) |  | 25592 (13245) | 26086 (7130) | 24226 (11582) | 20016 (5815) | 36326 (26923) | 0.199 |
| Pre-pregnency BMI (mean (SD)) |  | 24.71 (5.45) | 26.68 (7.97) | 24.34 (3.65) | 22.50 (4.14) | 24.67 (7.41) | 0.508 |
| FCT 3 month (%) | FCT1&2 | 29 (56.9) | 8 (66.7) | 14 (51.9) | 3 (50.0) | 4 (66.7) | 0.786 |
|  | FCT3 | 22 (43.1) | 4 (33.3) | 13 (48.1) | 3 (50.0) | 2 (33.3) |  |
| Sex (%) | 1 | 25 (49.0) | 7 (58.3) | 15 (55.6) | 3 (50.0) | 0 (0.0) | 0.080 |
|  | 2 | 26 (51.0) | 5 (41.7) | 12 (44.4) | 3 (50.0) | 6 (100.0) |  |
| Maternal antibiotics (%) | 0 | 47 (92.2) | 10 (83.3) | 25 (92.6) | 6 (100.0) | 6 (100.0) | 0.657 |
|  | 1 | 4 (7.8) | 2 (16.7) | 2 (7.4) | 0 (0.0) | 0 (0.0) |  |
| Infant antibiotics (%) | 0 | 42 (82.4) | 8 (66.7) | 22 (81.5) | 6 (100.0) | 6 (100.0) | 0.249 |
|  | 1 | 9 (17.6) | 4 (33.3) | 5 (18.5) | 0 (0.0) | 0 (0.0) |  |
| Previous deliveries (%) | >0 | 18 (35.3) | 7 (58.3) | 7 (25.9) | 1 (16.7) | 3 (50.0) | 0.170 |
|  | no previous deliveries | 33 (64.7) | 5 (41.7) | 20 (74.1) | 5 (83.3) | 3 (50.0) |  |
| Secretor (%) | 0 | 9 (17.6) | 2 (16.7) | 4 (14.8) | 2 (33.3) | 1 (16.7) | 0.702 |
|  | 1 | 42 (82.4) | 10 (83.3) | 23 (85.2) | 4 (66.7) | 5 (83.3) |  |
| Breastfeeding status (%) | no | 36 (70.6) | 8 (66.7) | 23 (85.2) | 2 (33.3) | 3 (50.0) | **0.010** |
|  | partial | 8 (15.7) | 2 (16.7) | 1 (3.7) | 4 (66.7) | 1 (16.7) |  |
|  | unknown | 7 (13.7) | 2 (16.7) | 3 (11.1) | 0 (0.0) | 2 (33.3) |  |
